# A systems biology model of junctional localization and downstream signaling of the Ang–Tie signaling pathway

**DOI:** 10.1038/s41540-021-00194-6

**Published:** 2021-08-20

**Authors:** Yu Zhang, Christopher D. Kontos, Brian H. Annex, Aleksander S. Popel

**Affiliations:** 1 Department of Biomedical Engineering, School of Medicine, Johns Hopkins University, Baltimore, MD USA; 2 Department of Medicine, Division of Cardiology, Duke University Medical Center, Durham, NC USA; 3 Department of Medicine and the Vascular Biology Center, Medical College of Georgia at Augusta University, Augusta, GA USA

**Keywords:** Cell biology, Computer modelling

## Abstract

The Ang–Tie signaling pathway is an important vascular signaling pathway regulating vascular growth and stability. Dysregulation in the pathway is associated with vascular dysfunction and numerous diseases that involve abnormal vascular permeability and endothelial cell inflammation. The understanding of the molecular mechanisms of the Ang–Tie pathway has been limited due to the complex reaction network formed by the ligands, receptors, and molecular regulatory mechanisms. In this study, we developed a mechanistic computational model of the Ang–Tie signaling pathway validated against experimental data. The model captures and reproduces the experimentally observed junctional localization and downstream signaling of the Ang–Tie signaling axis, as well as the time-dependent role of receptor Tie1. The model predicts that Tie1 modulates Tie2’s response to the context-dependent agonist Ang2 by junctional interactions. Furthermore, modulation of Tie1’s junctional localization, inhibition of Tie2 extracellular domain cleavage, and inhibition of VE-PTP are identified as potential molecular strategies for potentiating Ang2’s agonistic activity and rescuing Tie2 signaling in inflammatory endothelial cells.

## Introduction

The Angiopoietin (Ang)-Tie signaling pathway is a major endothelial signaling pathway regulating vascular quiescence, permeability, stability, and growth^1,2^. Disrupted Tie2 signaling has been linked to many diseases including cancer^3–5^, peripheral arterial disease^6^, ocular diseases, including diabetic retinopathy and diabetic macular edema^7–9^, systemic inflammation^10,11^, and infectious diseases including COVID-19^12,13^.

The Ang–Tie signaling pathway consists of multiple ligands, receptors, and molecular regulators, and mechanisms that form a complex reaction network. The Ang family of ligands consists of Ang1, Ang2, Ang3, and Ang4. Ang1 is the natural agonist of Tie2, and it is associated with vascular growth and stability^14,15^. Ang2 is primarily considered to be an antagonist of Tie2 and is mostly associated with vascular leakage and inflammation, although it also acts as a context-dependent Tie2 agonist^16–21^. Ang1 exists in highly oligomerized forms, whereas Ang2 exists primarily as lower-order multimers (e.g., dimers), although higher-order oligomeric forms are observed^22^. Multimeric ligands Ang1 and Ang2 are known to induce the multimerization and subsequent activation of Tie2, and the activation of Tie2 requires tetrameric or higher orders of multimerization^22^. Ang3 and Ang4 are mouse and human orthologs, respectively, with poorly defined biological functions^23^.

The Tie receptor family consists of Tie2, which directly binds the angiopoietins, and Tie1, which does not bind to any of the angiopoietins, but can form heterodimers with Tie2 and modulate its signaling^18,24–26^. A recent study has identified a secreted protein from hepatocytes, leukocyte cell-derived chemotaxin 2, as a ligand of Tie1 in liver fibrogenesis^27^. Heparan sulfate glycosaminoglycans have also been shown to bind Tie1 and regulate Ang–Tie signaling^28^. Tie1’s exact mechanisms of action and its effects on Tie2 signaling remain incompletely understood. It has been demonstrated to be able to both inhibit and enhance Tie2 activation^18,29–33^. Tie2 activation is also importantly regulated by vascular endothelial protein tyrosine phosphatase (VE-PTP), which dephosphorylates Tie2 to inhibit its vascular stabilizing effects^34^. The Ang–Tie pathway is also regulated by the extracellular domain cleavage of both receptors. The extracellular domains of both Tie1 and Tie2 are known to be cleaved near the plasma membrane both constitutively and in response to cytokine stimulation, resulting in soluble forms of the receptors (sTie1 and sTie2)^35–37^. The endodomains of cleaved Tie1 and Tie2 undergo further proteolytic processing and are subsequently degraded^36^. sTie2 can act as a ligand trap, further inhibiting the signaling of Tie2, although the physiological role of this process is unclear.

In confluent endothelial cells, Ang1 and Ang2 induce Tie2’s subcellular localization towards endothelial cell junctions^38,39^. Junctional Tie2 activates downstream Akt signaling, promoting endothelial cell stability and survival^38–40^. Activated Tie2 also antagonizes pro-permeability signaling by sequestering Src kinase through RhoA and mDia^41,42^.

Therapeutics targeting the Ang–Tie signaling pathway to promote vascular stability and normalization are being explored extensively due to the essential role of the Ang–Tie signaling pathway in a wide variety of diseases. Among many others, an Ang2-binding Tie2 activating antibody (ABTAA) promotes Ang2-stimulated Tie2 activation by forming a complex with Ang2 and Tie2, simultaneously promoting Tie2 activation and inhibiting Ang2’s antagonistic effect^43^; small molecule inhibitors of VE-PTP (AKB-9778 and AKB-9785) promote the activation of Tie2 and vascular stability^7,44^; Faricimab, a bispecific antibody designed to simultaneously inhibit Ang2 and VEGF-A, promotes vascular stability and quiescence^45,46^; and AXT107, an integrin-binding peptide, promotes Tie2 activation by modulating integrin/Tie interactions^8,20^.

The complexity of the context-dependent interactions and effects of Ang–Tie signaling has created significant challenges to obtaining a quantitative understanding of the molecular control of the Ang–Tie signaling pathway. A systems biology approach to computational modeling of signaling networks allows the complex integration of the molecular mechanisms at a network level. Previously, we have developed computational models of vascular signaling pathways, including the vascular endothelial growth factor (VEGF)^47–49^ and the hepatocyte growth factor pathways^50^. Alawo et al.^51^ also used a computational model to study interactions of Ang1 and Ang2 with the Tie receptors on the endothelial cell surface. The present study aims to extend the computational model for the Ang–Tie interaction at the endothelial cell surface^52^ to include junctional localization, homo- and heteromeric receptor interactions, and downstream Ang–Tie signaling to quantitatively investigate the molecular mechanisms for Tie1’s role, the context-dependence of Ang2’s agonist activity, and their implications for downstream signaling.

In the present study, we developed a mechanistically detailed computational model validated against previously published experimental data to study the molecular control of the Ang–Tie signaling pathway. The model captures and reproduces the ligand–receptor interactions and dynamics, junctional localization, and regulatory mechanisms, including the action of Tie1 and VE-PTP. The model demonstrates that Tie1’s junctional localization and its interaction with Tie2 at the endothelial junctions provide an explanation for its time-dependent effect on activation of Tie2. The model also shows that Tie1’s ability to sustain Tie2 activation is essential for the agonist effects of Ang2. The model provides a mechanistic explanation of how extracellular domain shedding of Tie1 in inflammatory endothelial cells potentiates Ang2’s antagonistic effect on Tie2 activation. The model also shows that inhibiting extracellular domain shedding of Tie2, promoting the junctional localization of Tie1, and inhibiting VE-PTP all have synergistic effects on rescuing Tie2 activation in inflammatory conditions. The model provides a platform to quantitatively study the molecular mechanisms of Ang–Tie signaling and their effects on the reaction network at a systems level and motivates additional experiments to further elucidate the mechanistic aspects of the pathway.

## Results

### A mechanistic computational model of the Ang/Tie pathway, junctional localization, and downstream signaling

**Fig. 1:**
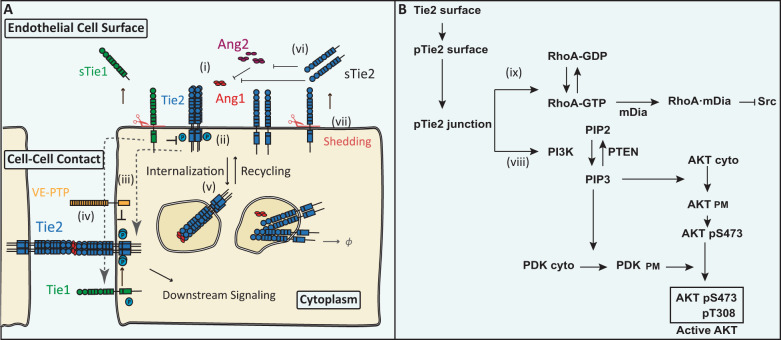
Diagram of the model assumptions. **A***Ligand receptor interactions, receptor dynamics, and molecular regulatory mechanisms*. Tetrameric Ang1 and multimeric Ang2 induce the multimerization, internalization, activation, and junctional localization of Tie2. Tie1 binds and inhibits Tie2 at the luminal surface but binds to Tie2 and sustains its activity at cell–cell junctions. Extracellular domains of Tie1 and Tie2 are cleaved from the cell surface to form soluble receptors. Soluble Tie2 acts as a ligand trap to bind both Ang1 and Ang2. **B** *Downstream signaling of Tie2*. Activated Tie2 sequesters Src kinase through RhoA and mDia and activates Akt via PI3K.

To develop a mechanistically detailed model of the Ang/Tie signaling pathway and its downstream signaling, we included a mass action reaction network of ligand/receptor interactions, regulation by co-receptor Tie1 and phosphatase VE-PTP, receptor trafficking, turnover, subcellular localization to the endothelial cell–cell junctions, as well as downstream signaling. The reaction diagram is shown in Fig. 1. The model is constructed using a rule-based modeling language BioNetGen^53^, and detailed reaction rules and molecular reactions of the model are described in the Methods section. All model equations, values of parameters, reaction rules in BioNetGen Language (BNGL) and Systems Biology Markup Language (SBML) computer code are available in the Supplementary files.

Ang1 molecules are assumed to be in tetrameric form, whereas Ang2 molecules exist in dimeric, trimeric, and tetrameric forms (Fig. 1A). Multimeric ligands can bind to Tie2 and induce its multimerization. Activation of Tie2 requires high order of multimerization, and only tetrameric Ang1 and Ang2 can further induce the partial activation and junctional localization of Tie2. VE-PTP catalyzes the dephosphorylation of activated Tie2 at the endothelial junctions. At the surface of the cell, Tie1 heterodimerizes with any oligomeric form of Tie2, limiting its access to ligands. At the endothelial junctions, Tie1 heterodimerizes with the tetrameric form of Tie2, and the receptors cross-phosphorylate to sustain Tie2 activation. Tie1 binding at the junctions also limits VE-PTP’s access to activated Tie2. Tie2 undergoes internalization upon binding to the ligands, releasing the ligands back to the cell surface. Internalized Tie2 undergoes both recycling and degradation at different rates. The extracellular domains of both Tie1 and Tie2 are cleaved from the surface of the cell constitutively to form soluble Tie1 and soluble Tie2. The endodomains of cleaved Tie1 and Tie2 are assumed to be rapidly degraded. Soluble Tie2, in turn, acts as a ligand trap, binding to both Ang1 and Ang2.

The model reactions also include the anti-permeability and pro-stability downstream signaling pathways of Tie2 (Fig. 1B). Phosphorylated Tie2 at the endothelial junctions activates the GTPase RhoA, turning it into the GTP-bound RhoA. RhoA-GTP then binds and forms a complex with the protein mDia. The RhoA-GTP/mDia complex binds and sequesters Src kinase and inhibits the pro-permeability signaling through Src kinase^41,42^. Phosphorylated Tie2 at the junctions also activates Akt through PI3K/PDK^38,39,48^.

### The computational model incorporates the ligand–receptor interactions and receptor dynamics reported in the literature

**Fig. 2:**
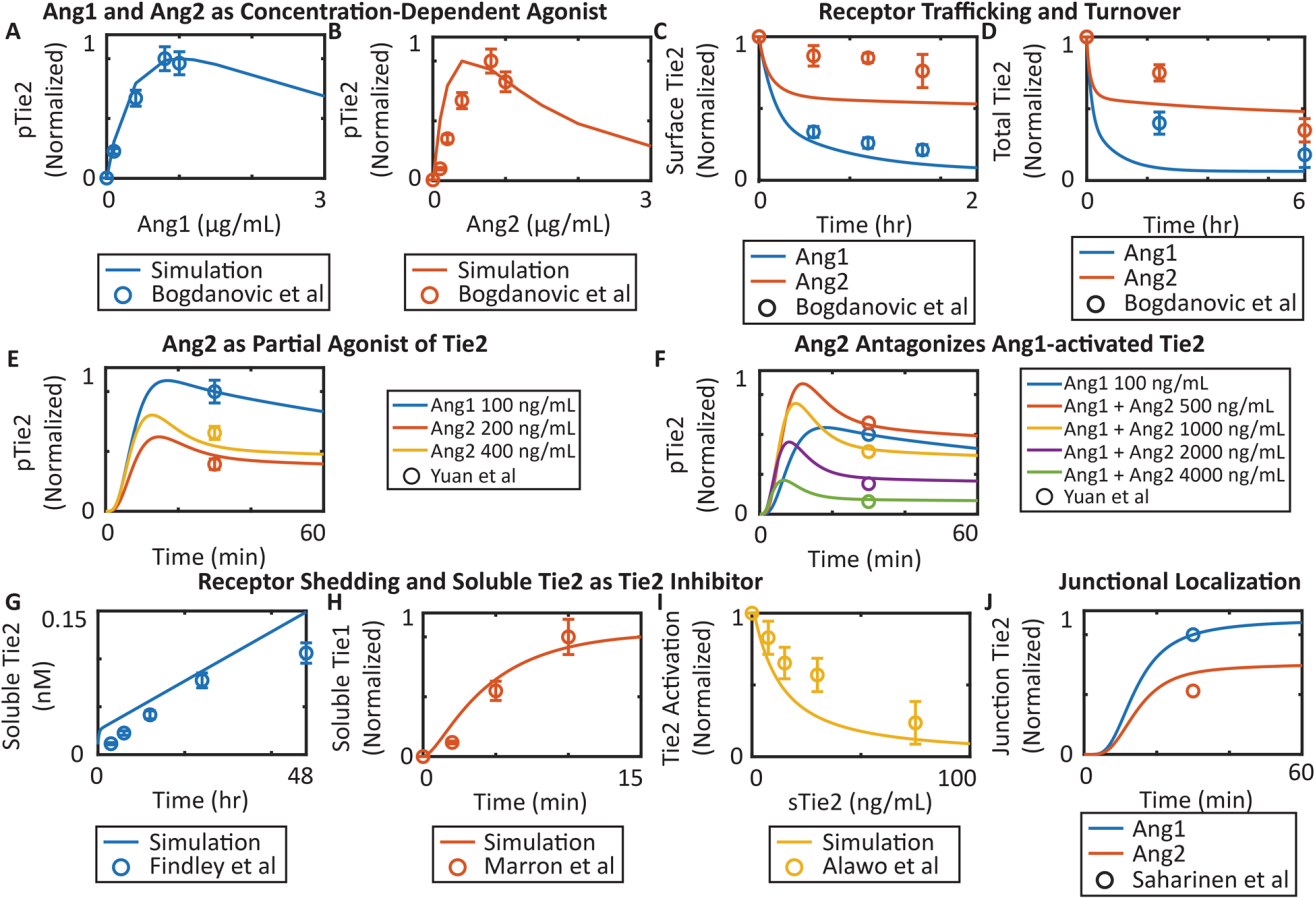
Model calibration of ligand–receptor interactions and receptor dynamics using global optimization. **A**, **B** Ang1 and Ang2 are concentration-dependent agonizts of Tie2 (Bogdanovic et al.^70^). **C**, **D** Ang1 and Ang2 induce receptor internalization and degradation of Tie2 (Bogdanovic et al.^70^). **E** Ang2 is a weaker agonist compared to Ang1 (Yuan et al.^54^). **F** Ang2 antagonizes Ang1-activated Tie2 at high concentrations (Yuan et al.^54^). **G**, **H** Extracellular domains of Tie1 and Tie2 are constitutively cleaved from the surface of the cell (Findley et al.^35^, Marron et al^36^). **I** Soluble Tie2 inhibits Tie2 activation in a concentration-dependent manner (Alawo et al.^51,71^). **J** Ang1 and Ang2 induce junctional localization of Tie2 (Saharinen et al.^39^).

The model shows that both Ang1 and And2 ligands act as concentration-dependent agonizts for Tie2, in agreement with experimental studies (Fig. 2A, B). Both Ang1 and Ang2 induce internalization and degradation of Tie2 at different rates (Fig. 2C, D). Compared to Ang1, Ang2 is a weaker agonist of Tie2^54^ (Fig. 2E). Ang2 at low concentration contributes to Ang1-stimulated activation of Tie2; a high concentration of Ang2, however, antagonizes the agonistic activity of Ang1 on Tie2^54^ (Fig. 2F). The extracellular domains of Tie1 and Tie2 are constitutively cleaved from the surface of the cell, forming the soluble form of the receptors, sTie1 and sTie2^35,36^ (Fig. 2G-H). Soluble Tie2, acting as a ligand trap by binding to Ang1 and Ang2, functions as a concentration-dependent inhibitor of Tie2 (Fig. 2I). Both Ang1 and Ang2 induce junctional localization of Tie2, with Ang1 being a more potent inducer of Tie2’s junctional localization^39^ (Fig. 2J).

**Fig. 3:**
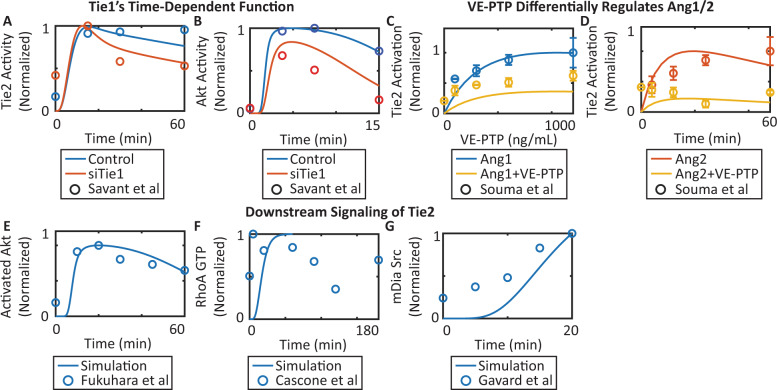
Model calibration of molecular regulatory mechanisms of downstream signaling of the Ang–Tie pathway. **A** Tie1 silencing acutely inhibits but chronically sustains Tie2 activation (Savant et al.^33^). **B** Tie1 silencing inhibits Akt activation (Savant et al.^33^). **C**, **D** VE-PTP differentially regulates Ang1- and Ang2-activated Tie2 (Souma et al.^17^). **E**–**G** Ang1 activates Tie2 and its downstream signaling including activation of Akt, RhoA-GTP, and Src sequestration by mDia (Cascone et al.^42^, Fukuhara et al.^38^, Gavard et al.^41^).

The model also captures and reproduces the molecular regulatory mechanisms and the downstream signaling of the Ang–Tie pathway (Fig. 3). Silencing Tie1 enhances Tie2 activation up to 20 min after stimulation but inhibits Tie2 activation beyond 20 min post-stimulation, suggesting that Tie1 acutely inhibits Tie2 activation but chronically sustains and enhances Tie2 phosphorylation^33^ (Fig. 3A). Silencing Tie1 inhibits the activation of Akt^33^ (Fig. 3B). Ang2-stimulated Tie2 is more sensitive to dephosphorylation by VE-PTP^17^ (Fig. 3C, D). Downstream of activated Tie2, Ang1 induces phosphorylation of Akt^38^ (Fig. 3E). Ang1 also induces RhoA’s conversion to its GTP-bound form and the subsequent sequestration of Src^41,42^ (Fig. 3F, G).

### Tie1 acts as a time-dependent inhibitor/enhancer of Tie2 activation and provides context for Ang2’s agonistic/antagonistic activity

**Fig. 4:**
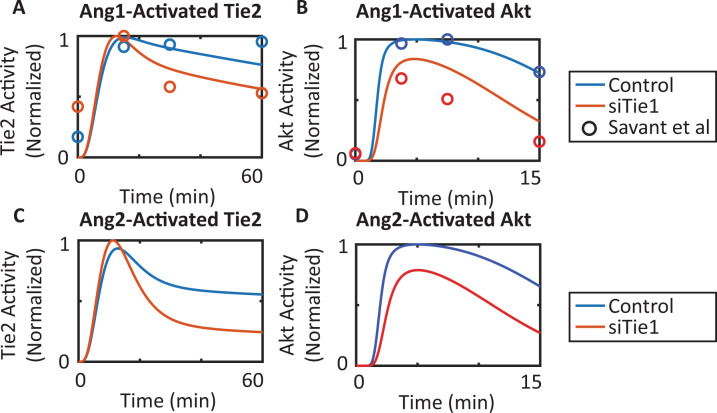
Tie1 sustains the activation of Tie2 and its downstream signaling and is essential for Ang2’s agonistic activity. Tie1 silencing acutely enhances but chronically suppresses Tie2 activation by (**A**) Ang1 and (**C**) Ang2. Tie1 silencing suppresses Akt activation by (**B**) Ang1 and (**D**) Ang2.

The model shows that when Tie2 is stimulated by Ang1, Tie1 acutely inhibits the activation of Tie2 but chronically helps sustain Tie2’s action, in agreement with the experimental observations by Savant et al.^33^ (Fig. 4A). For validation, the model is then used to predict the effect of Tie1 silencing on Ang2 stimulation. When stimulated by Ang2, silencing Tie1 initially slightly enhanced Tie2’s activation. At later time points (after 20 min post-stimulation), silencing Tie1 significantly reduced Ang2-mediated Tie2 activation by up to 50%, suggesting that Tie1’s ability to sustain Tie2 activation is essential for Ang2’s agonist effect (Fig. 4C). This simulation result agrees with the experimental observation that Tie1 is required for Ang2’s agonistic action on Tie2^29^. For both Ang1 and Ang2 stimulation, silencing Tie1 inhibits the activation of Akt downstream of Tie2, suggesting that Tie1 enhances and sustains both the Ang1- and Ang2-stimulated Akt activation (Fig. 4B, D). At the control level of Tie1, low concentrations of Ang2 enhance Ang1-activated Tie2. Tie1 silencing converts low concentration Ang2 into an antagonist of Ang1-activated Tie2 (Supplementary Fig. 1). The results indicate that Tie1’s ability to sustain activated Tie2 at the endothelial junctions is essential for Ang2’s agonistic activity and are consistent with the observation that loss of Tie1 potentiates Ang2’s antagonistic effect.

### Activation of Tie2 and its downstream signaling is modulated by the molecular concentration ratios of both Ang ligands and Tie receptors

**Fig. 5:**
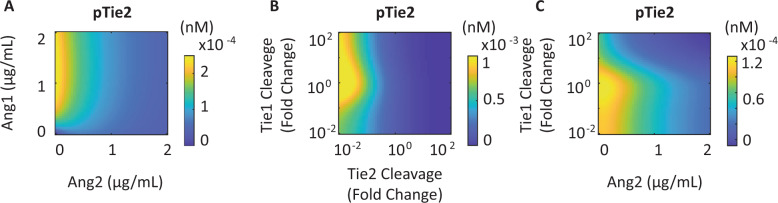
Molecular concentration ratios of ligands and receptors modulate the activity of Tie2. **A** Tie2 activation is modulated by the concentration of Ang1 and Ang2. **B** Extracellular domain cleavage rates of Tie1 and Tie2 affect Tie2 activity by modulating the molecular ratio of Tie1:Tie2. **C** Tie1 cleavage potentiates Ang2’s antagonistic effect, leading to a low level of Tie2 activation.

The model demonstrates that at low concentrations of Ang1 (below 500 ng/mL), Ang2 acts mostly as an agonist, contributing to the activation of Tie2. At a high concentration of Ang1, Ang2 antagonizes the activation of Tie2 by Ang1 (Fig. 5A). The model also makes quantitative predictions about how the combinations of changes in cleavage of the extracellular domains of Tie1 and Tie2 affect the steady-state activation of Tie2 (at 60 min post-stimulation) (Fig. 5B). Tie2 activation is inhibited by an increase in Tie2 cleavage; both an increase and a decrease in the cleavage rate of Tie1 inhibit Tie2’s activation. Simulation results indicate that Tie2’s activation can be modulated by altering the extracellular domain shedding rates of Tie1 and Tie2, consistent with the experimental observations that cytokines regulate Tie2 signaling by inducing receptor cleavage and, therefore, the molecular ratio of Tie1 to Tie2^37^. The model demonstrates that increased Tie1 cleavage combined with elevated Ang2 concentration leads to suppression of Tie2 activation, consistent with the experimental observations that endothelial cells in inflammatory states are often characterized by increased Tie1 cleavage and Ang2 release^36,55^. Although a low concentration of Ang2 is agonistic when Tie1’s cleavage rate is low, increased Tie1 cleavage converts a low concentration of Ang2 from being agonistic to antagonistic, potentiating its antagonistic effect (Fig. 5C).

### Tie2 activation and its downstream signaling are sensitive to parameters for receptor trafficking, turnover, and regulation by Tie1 and VE-PTP

**Fig. 6:**
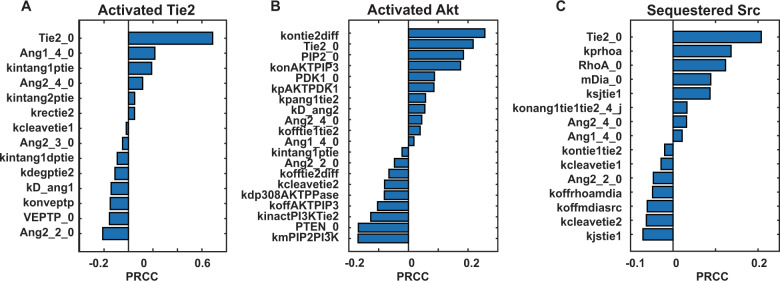
Global sensitivity analysis using LHS/PRCC. Partial rank correlation coefficients for significantly sensitive parameters (having nonzero PRCC values with Bonferroni-corrected *p*-values < 0.05) for **A** activated Tie2, **B** activated Akt, and **C** sequestered Src. Positive PRCCs indicate that an increase in the parameter is correlated with a positive change in the model output; negative PRCCs indicate that an increase in the parameter is correlated with a negative change in the model output.

Global sensitivity analysis was performed to identify the key parameters affecting the model outputs and potential molecular mechanisms as therapeutic targets. Model parameters and initial conditions were varied from one-tenth to ten times the best-fit values using Latin hypercube sampling (LHS) to calculate the partial rank correlation coefficients (PRCCs) for model outputs activated Tie2, activated Akt, and sequestered Src at 30 min post-stimulation (Fig. 6)^56^. Global sensitivity analysis demonstrates that the activity of Tie2 at steady-state (60 min post-stimulation) is sensitive to parameters related to extracellular domain cleavage of Tie1 (kcleavetie1), Tie2 trafficking, and turnover (kintang1ptie, kintang1dptie, krectie2, and kdegptie2), VE-PTP expression (VEPTPT_0, konveptp), and initial levels of Tie2, Ang1, and Ang2 (Tie2_0, Ang1_4_0, Ang2_4_0. Ang2_2_0) (Fig. 6A). Junctional localization of Tie1 affects both the internalization and degradation of Tie2 by sustaining Tie2 at the endothelial junction and protecting Tie2 from dephosphorylation by VE-PTP.

In addition to the sensitive parameters for activated Tie2, activated Akt is also sensitive to initial levels of PTEN, PIP2, PDK1, and the reaction rates of PI3K/Tie2 interaction and PTEN/PIP3 interaction (kmPIP2PI3K, kinactPI3KTie2) (Fig. 6B). Sequestered Src is sensitive to the phosphorylation rate of RhoA (kprhoa), the initial levels of RhoA and Src, and Tie1 synthesis (ksyntie1), cleavage (kcleavetie1), junctional localization (ksjtie1, kjstie1), and binding to Tie2 at junctions (konang1tie1tie2_j) (Fig. 6C). The PRCCs for up to ten positively correlated and ten negatively correlated, statistically significant parameters (Bonferroni-corrected *p*-values < 0.05) are shown in Fig. 6. The PRCCs of all parameters on activated Tie2, internalized Tie2, junctional Tie2, phosphorylated Tie2, activated Akt, and sequestered Src are shown in Supplementary Fig. 2.

### Sustained Tie2 expression, junctional localization of Tie1, and VE-PTP inhibition synergistically rescue Tie2 activation and its downstream signaling in inflammatory endothelial cells

**Fig. 7:**
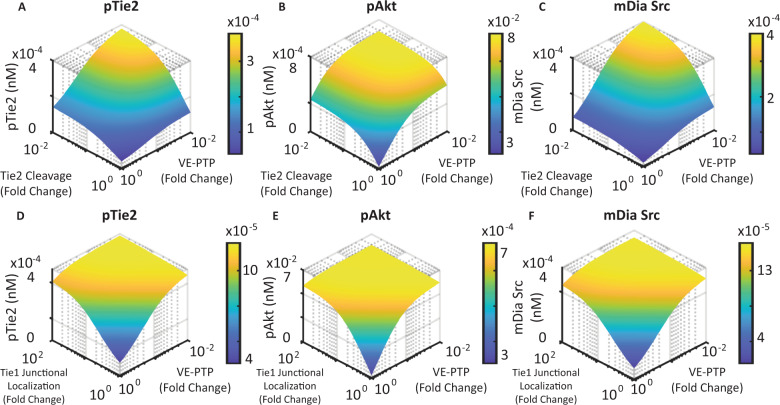
Inhibiting Tie2’s cleavage, promoting Tie1’s localization to the junction, and inhibition of VE-PTP synergistically enhance Tie2 activation and downstream signaling. **A**–**C** Inhibition of Tie2 cleavage and VE-PTP, **D**–**F** promoting Tie1’s junctional localization and inhibition of VE-PTP synergistically enhances Tie2 activation, Akt activation, and Src sequestration.

The model predicts that for endothelial cells in inflammatory states with elevated Tie1 cleavage and high Ang2 concentration, inhibition of Tie2 activity can be rescued by sustaining Tie2 at the cell surface (inhibiting the extracellular domain cleavage of Tie2), promoting Tie1’s junctional localization, and inhibiting VE-PTP. The model also predicts that inhibiting the extracellular domain cleavage of Tie2 and inhibiting VE-PTP synergistically enhance the activation of Tie2 in inflammatory endothelial cells with increased Tie1 cleavage rate and a high level of Ang2. Endothelial cells in the inflammatory state are assumed to have elevated Tie1 cleavage rate and Ang2 concentration. The model predicts that inhibiting Tie2’s cleavage rate and inhibiting VE-PTP by up to 1% alone enhances Tie2 activation by up to threefold. Simultaneous inhibition of Tie2 cleavage and VE-PTP synergistically enhances Tie2 activation by up to 10-fold (Fig. 7A). Inhibition of Tie2 cleavage and VE-PTP also synergistically enhances Tie2’s downstream signaling, including Akt activation and Src sequestration (Fig. 7B, C). The model also predicts that promoting Tie1’s localization rate to the cell junction by up to 100-fold enhances Tie2 activation by up to 2.5-fold, and when combined with VE-PTP inhibition, synergistically enhances Tie2 activation, Akt activation, and Src sequestration (Fig. 7D, F).

## Discussion

Tie1 has been reported as both an inhibitor^31,36,57^ and an enhancer^29,33^ of Tie2’s activation. The exact molecular mechanisms responsible for the dual-action of Tie1 remain incompletely understood. Our model is the first mechanistic computational model with detailed molecular mechanisms of ligand–receptor interactions, junctional localization, and other regulatory mechanisms to quantitatively investigate the seemingly contradictory role of Tie1. The model demonstrates that Tie1 inhibits Tie2 at the cell surface by forming heterodimers with Tie2 but promotes and sustains Tie2’s activation at the endothelial junctions by binding with Tie2 and protecting Tie2 from internalization and dephosphorylation by VE-PTP. The proposed mechanisms capture and reproduce the time-dependent role that Tie1 plays in regulating Tie2’s signaling, consistent with the experimental observation by Savant et al. that Tie1 acutely inhibits but chronically promotes and sustains Tie2’s activation^33^

The model shows that silencing Tie1 chronically inhibits both the Ang1-stimulated and Ang2-stimulated activation of Tie2, and the inhibitory effect of Tie1 silencing is stronger on Ang2-activated Tie2. When simulating Ang2’s activation of Tie2, the model shows that the inhibition of Tie2 activation by Tie1 silencing is stronger compared to Ang1, resulting in a 50% reduction of Tie2 activation, indicating that Tie1’s effect of promoting and sustaining Tie2 activation is essential for Ang2’s activation of Tie2. This observation is consistent with the experimental findings of a recent study by Korhonen et al. demonstrating that Tie1 is required for Ang2’s activation of Tie2^29^. We hypothesize that Tie1’s molecular mechanisms proposed by the model, including its heteromeric formation, co-localization, and cross-activation with Tie2 at cell junctions can explain Ang2’s agonistic function in presence of Tie1 and its antagonistic function in absence of Tie1.

Previously, our computational model has demonstrated that VE-PTP provides context for Ang2’s agonistic activity by differentially regulating Ang1- and Ang2-stimulated Tie2^52^. The present model demonstrates Tie1’s role in modulating Ang2’s activity.

Ang2 alone is a weak agonist of Tie2 and activates Tie2 and its downstream signaling. Tie1 provides context for Ang2’s agonistic effect by sustaining Ang2-activated Tie2 and protecting it from VE-PTP. The model predicts that silencing Tie1 inhibits sustained Ang2 activation of Tie2, essentially converting it into an antagonist (Fig. 4). Simulations show that in the presence of Tie1, a low concentration of Ang2 can enhance Ang1’s activation of Tie2, but in the absence of Tie1, Ang2 antagonizes Tie2’s activity even at low concentration (Fig. S1).

Loss of Tie1 by extracellular domain cleavage has been previously observed to contribute to the loss of Ang2’s agonistic activity and vascular stability, but the exact molecular mechanism is unclear^29^. The model demonstrates that compared to Ang1, Ang2’s agonistic activity is more sensitive to Tie1’s sustaining of activated Tie2 at the endothelial junctions (Fig. 4C), providing an explanation for the observation that loss of Tie1 contributes to Ang2’s antagonistic activity. The model also quantitatively demonstrates how the increase in Tie1 cleavage potentiates Ang2’s antagonistic effect (Fig. 5C). Cytokines such as VEGF and tissue necrosis factor (TNF) induce the extracellular domain cleavage of Tie1^37^. Ang2 has been shown to potentiate inflammation and act in concert with VEGF to promote angiogenesis and vascular remodeling. Thus, increased Tie1 cleavage in these states is consistent with a Tie2-inhibitory, vascular destabilizing effect of Ang2. Inflammatory endothelial cells also release Ang2 from Weibel–Palade bodies^55^, further inhibiting the activation of Tie2. We propose that molecular strategies to sustain Tie1 expression by inhibiting its cleavage or promoting its junctional localization could rescue Tie2’s activity by promoting Ang2’s agonistic effect.

**Fig. 8:**
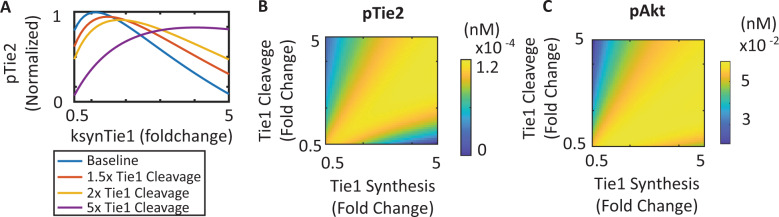
Increase in Tie1 synthesis rescues Tie2 activation in inflammatory endothelial cells with high Tie1 cleavage. **A** Increase in Tie1 synthesis enhances and then inhibits activation of Tie2 when Tie1 cleavage is at baseline, 1.5-fold, or 2-fold of its baseline value; when Tie1 cleavage is at 5-fold its baseline value, an increase in Tie1 synthesis enhances the activation of Tie2. Changes in the cleavage and synthesis rate of Tie1 affect the **B** phosphorylated Tie2 and **C** phosphorylated Akt.

The expression of Tie1 in endothelial cells has also been observed to increase in response to disturbing laminar flow and inflammation^33,58,59^. More recently, the populational difference of Tie1 expression has also been observed to correlate with the presentation of critical limb ischemia, a major manifestation of peripheral arterial disease^60^. Our model has suggested that Tie1 plays an important role in sustaining the signaling of Tie2 in inflammatory endothelial cells. Increased expression of Tie1 to replenish the cleaved Tie1 could therefore potentially enhance and rescue the disrupted Tie2 signaling. To quantitatively investigate the effect of increased Tie1 expression *in silico*, the model was used to simulate the effects of change in the synthesis rate of Tie1 on the activation of Tie2 and Akt. The model demonstrates that at baseline Tie1 cleavage rate, an increase in Tie1 synthesis initially enhances the activation of Tie2, but inhibits Tie2 activation when the synthesis rate is beyond twofold its baseline level. At a high Tie1 cleavage rate corresponding to inflammatory conditions, an increase in Tie1 synthesis up to fivefold its baseline value enhances Tie2’s activation (Fig. 8A). The activation of Tie2 at 30 min post-stimulation obtained by simultaneously varying the synthesis rate and cleavage rate of Tie1 suggests that the increase in Tie1 synthesis can effectively rescue the disrupted Tie2 activation and Akt activation in inflammatory endothelial cells and that an optimal ratio of Tie1 synthesis and cleavage rate produces a Tie1 turnover dynamics for Tie2 and Akt activation (Fig. 8B, C).

Korhonen et al. further demonstrated that the interaction between Tie1 and Tie2 is modulated by the integrin α5β1^29^. AXT107, an integrin-binding peptide, has been demonstrated to be able to enhance Tie2’s activation by binding integrin α5β1 and promoting Tie2’s junctional localization, promoting Ang2’s agonistic effect^8,20^. The model demonstrates that Tie1 and its junctional localization modulate Tie2’s response to Ang2 stimulation, suggesting that AXT107’s ability to convert Ang2 into an agonist can be explained by the integrin mediated Tie1/Tie2 interaction and their junctional localization. We propose that integrin can promote Ang2’s agonistic activity by sustaining Tie1 and enhancing Tie1/Tie2 interaction and junctional localization.

The computational model allows for quantitative predictions of how different concentration ratios of Ang1:Ang2 and ratios of Tie1:Tie2 affect Tie2’s activation. The model demonstrates that although low concentrations of Ang2 may be agonistic, high Ang2 concentrations antagonize Ang1-stimulated Tie2 (Fig. 5A), and that high Ang2:Ang1 ratios correspond to low activity of Tie2. Clinically, Ang2:Ang1 ratio has been evaluated as a prognostic predictor of multiple diseases, including sepsis and acute lung injury^61,62^. The molecular concentration ratio of Tie1:Tie2, controlled by modulating the extracellular domain cleavage rates of Tie1 and Tie2 also regulates the activity of Tie2 (Fig. 5B). Cytokines including VEGF and TNFα have been demonstrated to induce different extracellular domain cleavage rates of Tie1 and Tie2, thereby dynamically regulating Ang/Tie signaling^37^. Consistent with the endothelial cells in inflammatory conditions, high concentrations of Ang2 combined with increased Tie1 cleavage result in low Tie2 activation (Fig. 5C). The model quantitatively demonstrates that targeting Ang2 alone when Tie1 cleavage rate is high cannot effectively rescue Tie2’s activity (Fig. 5C). Rescuing Tie1 cleavage to its baseline rate potentiates a low concentration of Ang2 up to 400 ng/mL to be agonistic in vitro.

Inflammatory endothelial cells with disrupted Tie2 signaling are characterized by increased Tie1 cleavage and elevated Ang2^21,36,37^. The model suggests that Tie1’s cleavage potentiates Ang2’s antagonistic effect and leads to significantly inhibited Tie2 activity when combined with a high concentration of Ang2 (Fig. 5C). We propose that Tie2’s disrupted signaling in inflammatory endothelial cells can be rescued by promoting Ang2’s agonistic activity. The model suggests that VE-PTP inhibition and Tie1’s junctional localization both promote Ang2’s agonistic activity and that simultaneously inhibiting VE-PTP and promoting Tie1’s junctional localization would synergistically enhance Tie2’s activation in inflammatory endothelial cells (Fig. 7D). We also observed that VE-PTP inhibition is more effective and better synergizes with Tie2 sustaining in endothelial cells in inflammatory condition with elevated Tie1 cleavage and Ang2 (Supplementary Fig. 3A, B), suggesting that in inflammatory conditions, Tie1 cleavage causes an increased exposure of Tie2 to dephosphorylation by VE-PTP, causing the system to be sensitive to VE-PTP’s inhibition. We further observed that inhibition of VE-PTP beyond 1% of its baseline value effectively promotes Tie2’s activation (Supplementary Fig. 3C–F), suggesting that strong inhibition of VE-PTP is needed to enhance Tie2 activation. Further experimental studies that isolate the effect of Tie1 cleavage and VE-PTP expression would allow validation of the model and shed light on the molecular mechanisms for the synergistic effects observed by the model.

The Ang–Tie signaling pathway shares downstream signaling pathways and crosstalk with other receptor signaling pathways in various ways. The VEGF signaling pathway, another major vascular signaling pathway regulating angiogenesis and vascular permeability, promotes endothelial cell survival through PI3K/Akt and promotes vascular permeability through Src kinase^48,63^. The model predicts that the Ang–Tie signaling pathway potentially enhances the Akt signaling of VEGF via shared downstream signaling through PI3K/eNOS but inhibits the pro-permeability by sequestering Src kinase required for VEGF-induced permeability. Experimental studies have indeed demonstrated Ang1’s effect in reversing VEGF-induced permeability and contributing to its pro-stability signaling^41^. The VEGF signaling pathway can also regulate Tie2’s signaling by promoting the release of Ang2 from Weibel–Palade bodies and promoting the extracellular domain cleavage of Tie1 and Tie2^35,64^. Integrin αvβ3 has also been shown to affect signaling by both VEGF and Tie2^49,65,66^.

The crosstalk and shared downstream signaling pathways by the Ang–Tie pathway, VEGF pathway, and integrins form a complex reaction network that requires an integrative model to capture the response to different perturbations and conditions at a systems level. We have previously developed computational models for the VEGF signaling pathway and its interaction with integrins^47–49^. Integrative models allow for in silico investigations of simultaneous perturbations on multiple pathways in the reaction network that are not possible with isolated models.

The study of the Ang–Tie signaling pathway has been challenging due to the complexity of the reaction network and the limited molecular tools available for the molecules involved. Most in vivo experiments use genetic knockout of Tie1 or Tie2 as the method of choice and lead to conditions outside of biologic extremes^30,67–69^, posing additional challenges in obtaining a biologically relevant, quantitative understanding of the signaling pathways. The present study uses a rule-based mechanistic computational model validated against experimental data to quantitatively characterize the complex reaction network formed by the Ang–Tie signaling axis, including its ligand-receptor interactions, receptor trafficking and turnover, the context-dependent function of Tie1 and Ang2, other regulatory mechanisms including VE-PTP and cleaved receptors, as well as the downstream signaling. Our model is the first mechanistically detailed model of the Ang/Tie signaling axis and its junctional localization that incorporates and reproduces experimental observations from independent sources. The model incorporates, in mechanistic details, the current understanding of the molecular interactions in the Ang/Tie signaling axis with sufficient experimental data. The model does not, however, include several known mechanistic aspects of the pathway due to computational limitations and insufficient calibration data, including the internalization of Tie1, the effect of trans-association of Tie2 with adjacent endothelial cells, signaling of Tie2 at cell–ECM contact, and the different phosphorylation sites on the intracellular domains of Tie1 and Tie2^2,26,36,39^. New molecular mechanisms could be readily incorporated into the model should additional experimental evidence and data become available.

Our proposed mechanistic model hypothesizes that Tie1 sustains Tie2’s activation by co-localizing with Tie2 at the endothelial junction and inhibiting Tie2’s internalization, degradation, and de-phosphorylation by VE-PTP. The model also provides a mechanistically detailed molecular reaction network that predicts and explains how Tie1, its junction localization, heteromeric formation and activation with Tie2, and VE-PTP provide context for Ang2’s antagonistic/agonistic activity. In addition, the model makes quantitative predictions of how the endothelial cell responds to different concentrations of Ang1 and Ang2 with different receptor concentrations of Tie2 and Tie1. In inflammatory endothelial cells with elevated Tie1 cleavage and Ang2, the model identifies the inhibition of Tie2 cleavage, promoting Tie1’s junctional localization, and inhibiting VE-PTP as a potential target to rescue disrupted Tie2 activation. The model provides a platform for quantitative investigations of the Ang–Tie signaling pathway and its molecular mechanisms. The model also serves to formulate hypotheses on the effects of different perturbations on the signaling pathway to motivate further experimental studies.

## Methods

### Construction of the model using rule-based modeling

The model was constructed using the rule-based modeling language BioNetGen^53^. Molecular species and reactions are described with molecular reaction rules and corresponding reaction rates. The model reaction rules include receptor–ligand binding, ligand-induced multimerization of the receptors, receptor activation, junctional localization, trafficking and turnover, regulation by Tie1, VE-PTP, and receptor extracellular domain cleavage, soluble Tie2 acting as ligand trap, junctional interactions, and downstream signaling through Akt and Src sequestration (see Fig. 1).

The model reaction rules are available in the supplementary file as a BNGL file. The model reaction rules include (Fig. 1A): (i) oligomeric Ang1 (rules 1–4) and Ang2 (rules 5–13) bind and induce the oligomerization of Tie2, and (ii) tetrameric Ang1- and Ang2-bound Tie2 are subsequently activated and phosphorylated (rules 14–15). (iii) Tetrameric Tie2 and Tie1 localize at the endothelial junction, where subcellular localization is modeled as a state of the molecule with first-order localization dynamics (rules 16–18); Tie1 binds and cross-phosphorylates with Tie2 at the endothelial junction (rules 19–22). (iv) VE-PTP binds and catalyzes the dephosphorylation of activated Tie2 that are unbound to Tie1 at the endothelial junction (rules 23–25). (v) Tie1 and Tie2 are constitutively synthesized at constant rates (rules 49–50); Ang1- and Ang2-bound Tie2 are internalized at the cellular surface, releasing Ang1 and Ang2 back to the cell surface (rules 26–46); internalized Tie2 are subsequently degraded (rule 49) or recycled back to the cell surface (rule 47). (vi) Soluble Tie2 can bind and trap all oligomeric forms of Ang1 (rules 51–54) and Ang2 (rules 55–63). At the cellular surface, Tie1 heterodimerizes with Tie2 (rule 64) and limits its oligomerization and activation by binding Ang1 and Ang2 (rules 65–69). (vii) The extracellular domain of Tie1 and Tie2 are cleaved off from the cellular surface, forming soluble forms of the receptors (rules 70–71); the soluble receptors are subsequently degraded at constant rates (rules 72–73). The endodomains of cleaved Tie1 and Tie2 are assumed to be rapidly degraded upon cleavage. The model also includes downstream signaling of activated Tie2 at the endothelial junctions. (viii) Activated Tie2 activates and phosphorylates Akt through Akt/PI3K (rules 74–84), the reaction rules, based on a computational model of the VEGF/VEGFR2 signaling pathway^48^, include: tha activation of PI3K by Tie2 (rules 74–75); generation of PIP2 (rule 76), its phosphorylation by PI3K (rule 77) and dephosphorylation by PTEN (rule 78); binding of PIP3 to Akt and PDK (rules 79,80); activation of Akt at S473 by mTOR (rule 81); activation of Akt at T308 by PDK (rule 82); dephosphorylation of active Akt (rules 83,84). (ix) Activated Tie2 also induces the activation of RhoA (rules 85–86), and its subsequent complex formation of mDia and Src (rules 87–88), preventing Src from phosphorylating VE-Cadherin (rules 89–94) (Fig. 1B). The complete model contains 94 reaction rules, 150 molecular species, 496 reactions, 19 initial condition inputs, and 159 parameters. The model analysis, including global optimization, global sensitivity analysis, and all subsequent simulations are performed in MATLAB (Mathworks, Natick, MA). A table of all parameters, observables, and their descriptions is included in Supplementary Tables 1 and 2. A detailed table of all the molecular species and reactions is available in Supplementary Dataset. The BNGL file with detailed molecular reaction rules and the model in SBML file are included in the Supplementary Dataset.

### Model calibration to experimental data using global optimization

The model parameters were calibrated to experimental data from independent literature sources. Global fitting algorithms, including fmincon and patternsearch in MATLAB’s global optimization toolbox (Mathworks, Natick, MA) are used to simultaneously fit all parameters to fit the experimental data on Ang1 and Ang2-induced Tie2 activation, internalization and turnover^70^, Ang2 as a weaker agonist of Tie2 and antagonizes Ang1-activated Tie2^54^, receptor shedding of both Tie1 and Tie2^35,36^, soluble Tie2 acting as a concentration-dependent inhibitor of Tie2^71^, junctional localization of Tie2 induced by Ang1 and Ang2^39^, Tie1 as a time-dependent regulator of activation of Tie2 and Akt^33^, VE-PTP as a differential regulator of Ang1- and Ang-activated Tie2^17^, and various downstream signaling of Tie2, including Akt^38^, RhoA-GTP^42^, and mDia-Src^41^.

### Performing global sensitivity analysis

Global sensitivity analysis is performed to understand the response of various model outputs to the changes in parameter space based on the methodology described in Marino et al^56^. Parameters are sampled from one-tenth to ten times their baseline best-fit values using LHS to obtain the PRCCs. Statistical significance was evaluated by calculating the *p*-values from the *t* test with Bonferroni correction. Up to the top ten positively correlated and ten negatively correlated, statistically significant sensitive parameters (Bonferroni-corrected *p* < 0.05) for model outputs active Tie2, active Akt, and sequestered Src are reported in Fig. 6 along with their PRCC values. PRCC values of all parameters to model outputs are reported in Supplementary Fig. 2.

### Reporting summary

Further information on research design is available in the Nature Research Reporting Summary linked to this article.

## Supplementary information

Supplementary Data 3

Supplementary Information

Reporting Summary

Supplementary Data 1

Supplementary Data 2

## Data Availability

The simulation data generated during this study as well as the model are included in the article and Supplementary data.

## Code availability

The model is provided in both BNGL and SBML format as Supplementary Data 2 and 3.
