# Supplementary material for: A systems biology model of junctional localization and downstream signaling of the Ang–Tie signaling pathway
Source: NPJ Syst Biol Appl. 2021 Aug 20;7:34. doi: 10.1038/s41540-021-00194-6 (PMC8379279; doi:10.1038/s41540-021-00194-6)
Supplement: Supplementary file 2 — Supplementary Information [file 41540_2021_194_MOESM2_ESM.pdf]

Supplemental Information

Junctional Localization and Downstream Signaling of the Angiopoietin-Tie Signaling Pathway: A Computational Model

Yu Zhang, Christopher D. Kontos, Brian H. Annex, and Aleksander S. Popel

SUPPLEMENTAL FIGURES

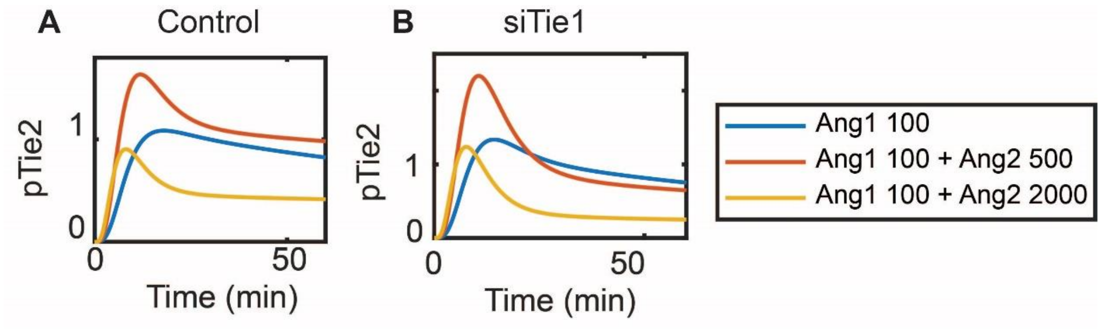

Supplemental Figure S1. See also **Figure 4**. Tie1 provides context for Ang2's agonistic/antagonistic activity. (A) In control condition, low concentration of Ang2 is agonistic and enhances Ang1-activated Tie2. (B) At low level of Tie1, low concentration of Ang2 is antagonistic and suppresses Ang1-activated Tie2.

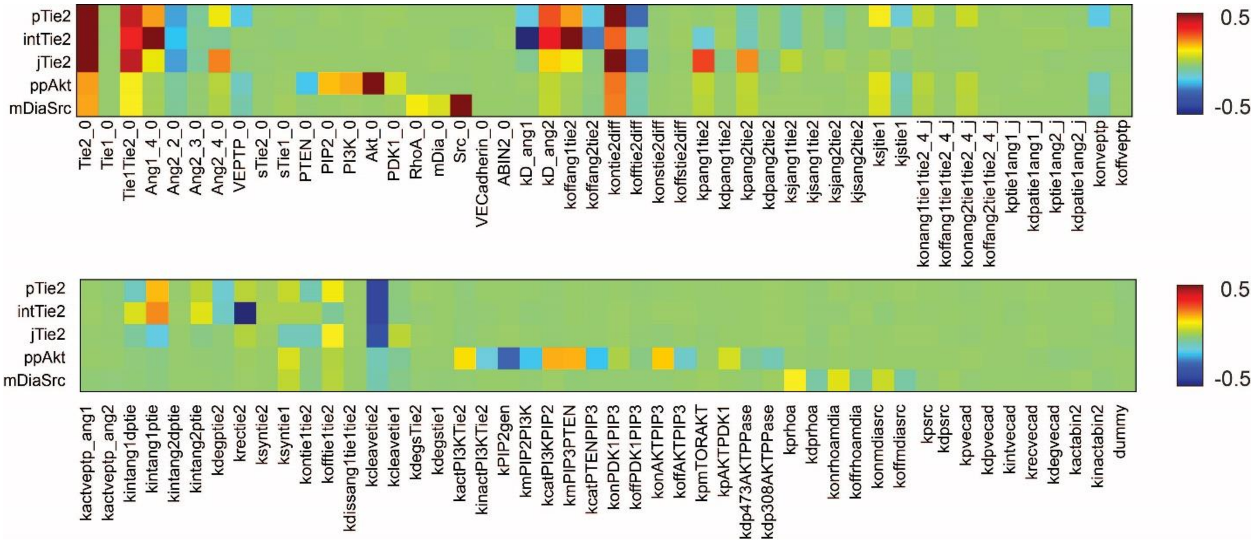

Supplemental Figure S2. See also **Figure 6**. Partial rank correlation coefficients for the sensitivity of phosphorylated Tie2, internalized Tie2, junctional Tie2, activated Akt, and sequestered Src to all parameters of the model.

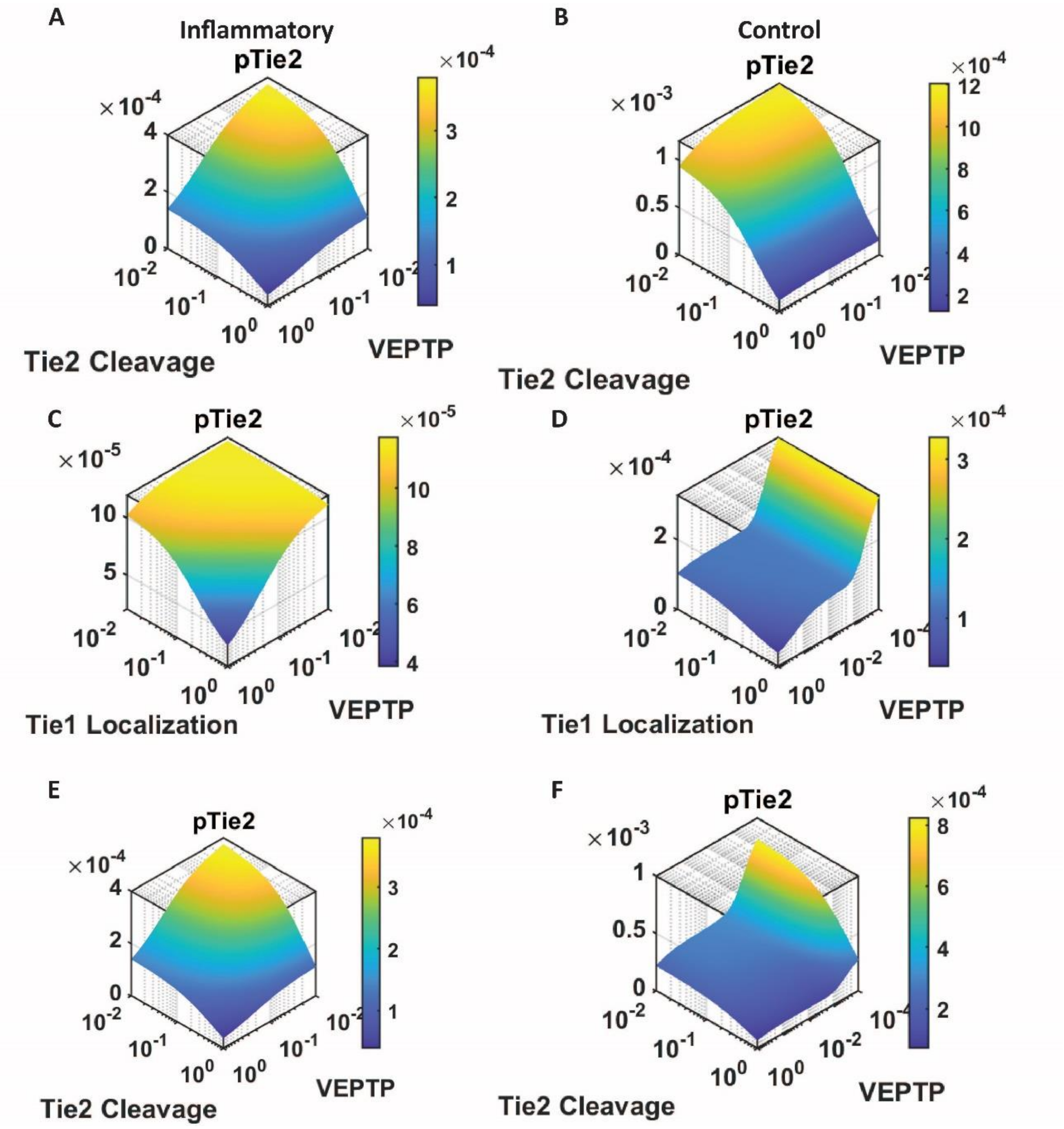

Supplemental Figure S3. See also **Figure 7**. (A-B) VE-PTP inhibition is more effective and better synergizes with Tie2 sustaining in inflammatory endothelial cells. (C-F) VE-PTP inhibition is more effective when inhibition is beyond 1%.

SUPPLEMENTAL TABLES

Supplemental Table S1. Table of all initial conditions and parameters in the model

	Initial Conditions				
**Initial Condition**	**Description**	**Rules**	**Unit**	**Value**	**Reference**
Tie2_0	Initial concentration of Tie2	Initial Condition	nM	1.03E-02	[1, 2]
Tie1_0	Initial concentration of Tie1	Initial Condition	nM	5.00E-03	[1, 2]
Tie1Tie2_0	Initial concentration of Tie1:Tie2 dimer	Initial Condition	nM	5.00E-03	[1, 2]
Ang1_4_0	Initial concentration of tetrameric Ang1	Initial Condition	nM	0.00E+00	-
Ang2_2_0	Initial concentration of dimeric Ang2	Initial Condition	nM	0.00E+00	-
Ang2_3_0	Initial concentration of trimeric Ang2	Initial Condition	nM	0.00E+00	-
Ang2_4_0	Initial concentration of tetrameric Ang2	Initial Condition	nM	0.00E+00	-
VEPTP_0	Initial concentration of VE-PTP	Initial Condition	nM	1.00E+03	[1]
sTie2_0	Initial concentration of soluble Tie2	Initial Condition	nM	1.07E-02	[3, 4]
sTie1_0	Initial concentration of soluble Tie1	Initial Condition	nM	2.13E-03	[5]
PTEN_0	Initial concentration of PTEN	Initial Condition	nM	1.00E-01	[6]
PIP2_0	Initial concentration of PIP2	Initial Condition	nM	1.00E+01	[6]
PI3K_0	Initial concentration of PI3K	Initial Condition	nM	1.00E-01	[6]
Akt_0	Initial concentration of Akt	Initial Condition	nM	1.00E-01	[6]
PDK1_0	Initial concentration of PDK1	Initial Condition	nM	1.00E-01	[6]
RhoA_0	Initial concentration of RhoA	Initial Condition	nM	1.00E-01	Assumed
mDia_0	Initial concentration of mDia	Initial Condition	nM	1.00E-01	Assumed
Src_0	Initial concentration of Src	Initial Condition	nM	1.00E-01	Assumed
VECadherin_0	Initial concentration of VE-Cadherin	Initial Condition	nM	1.00E-01	Assumed
	**Parameters**				
**Parameter**	**Description**	**Rules**	**Unit**	**Value**	**Reference**
kD_ang1	Dissociation rate constant of Ang1-Tie2 binding	1, 51,65	nM	3.70E+00	[7, 8]
kD_ang2	Dissociation rate constant of Ang2-Tie2 binding	5, 7, 10, 55, 57, 60, 67-69	nM	3.70E+00	[7, 8]
koffang1tie2	Dissociation rate of Ang1-Tie2 binding	1, 51, 65	s^{-1}	4.92E-05	Fitting
koffang2tie2	Dissociation rate of Ang2-Tie2 binding	5, 7, 10, 55, 57, 60, 67-69	s^{-1}	1.59E-04	Fitting
kontie2diff	Oligomerization rate of Tie2 by diffusion limit	2-4, 6, 8, 9, 11-13	$nM^{-1} s^{-1}$	3.21E-01	Fitting
kofftie2diff	Dissociation rate of oligomeric Tie2 by diffusion	2-4, 6, 8, 9, 11-13	s^{-1}	2.09E-03	Fitting
konstie2diff	Oligomerization rate of sTie2 by diffusion limit	52-54, 56, 58-59, 61-63	$nM^{-1} s^{-1}$	3.21E-01	Fitting
koffstie2diff	Dissociation rate of oligomeric sTie2 diffusion limit	52-54, 56, 58-59, 61-63	s^{-1}	2.09E-03	Fitting
kpang1tie2	Phosphorylation rate of Ang1-bound Tie2	14	s^{-1}	5.77E-03	Fitting

kdpang1tie2	Dephosphorylation rate of Ang1-bound Tie2	14	s^{-1}	5.83E-04	Fitting
kpang2tie2	Phosphorylation rate of Ang2-bound Tie2	15	s^{-1}	5.77E-03	Fitting
kdpang2tie2	Dephosphorylation rate of Ang2-bound Tie2	15	s^{-1}	5.83E-04	Fitting
ksjang1tie2	Junctional localization rate of Ang1-bound Tie2	16	s^{-1}	3.69E-02	Fitting
kjsang1tie2	Surface localization rate of Ang1-bound Tie2	16	s^{-1}	3.89E-04	Fitting
ksjang2tie2	Junctional localization rate of Ang2-bound Tie2	17	s^{-1}	3.69E-02	Fitting
kjsang2tie2	Surface localization rate of Ang2-bound Tie2	17	s^{-1}	3.89E-04	Fitting
ksjtie1	Junctional localization rate of Tie1	18	s^{-1}	2.25E-01	Fitting
kjstie1	Surface localization rate of Tie1	18	s^{-1}	1.41E-01	Fitting
konang1tie1tie2_4_j	Association rate of Ang1 to junctional Tie1/Tie2	19	$nM^{-1}\ s^{-1}$	6.16E-01	Fitting
koffang1tie1tie2_4_j	Dissociation rate of Ang1 to junctional Tie1/Tie2	19	s^{-1}	3.55E-06	Fitting
konang2tie1tie2_4_j	Association rate of Ang2 to junctional Tie1/Tie2	20	$nM^{-1}\ s^{-1}$	6.16E-01	Fitting
koffang2tie1tie2_4_j	Dissociation rate of Ang2 to junctional Tie1/Tie2	20	s^{-1}	3.55E-06	Fitting
kptie1ang1_j	Phosphorylation rate of junctional Ang1/Tie1	21	s^{-1}	1.63E-01	Fitting
kdpatie1ang1_j	Dephosphorylation rate of junctional Ang1/Tie1	21	s^{-1}	1.12E-07	Fitting
kptie1ang2_j	Phosphorylation rate of junctional Ang2/Tie1	22	s^{-1}	1.63E-01	Fitting
kdpatie1ang2_j	Dephosphorylation rate of junctional Ang2/Tie1	22	s^{-1}	1.12E-07	Fitting
konveptp	Association rate of VE-PTP to Tie2	23	s^{-1}	1.28E-03	Fitting
koffveptp	Dissociation rate of VE-PTP to Tie2	23	$nM^{-1}\ s^{-1}$	8.09E-02	Fitting
kactveptp_ang1	Enzymatic reaction rate of VE-PTP to Ang1-Tie2	24	s^{-1}	4.75E-01	Fitting
kactveptp_ang2	Enzymatic reaction rate of VE-PTP to Ang2-Tie2	25	s^{-1}	4.75E-01	Fitting
kintang1dptie	Internalization rate of Ang1-bound, dephospho-Tie2	26-33	s^{-1}	1.25E-03	Fitting
kintang1ptie	Internalization rate of Ang1-bound, phospho-Tie2	26-33	s^{-1}	1.19E-03	Fitting
kintang2dptie	Internalization rate of Ang2-bound, dephospho-Tie2	34-46	s^{-1}	2.12E-06	Fitting
kintang2ptie	Internalization rate of Ang2-bound, phospho-Tie2	34-46	s^{-1}	1.72E-04	Fitting
kdegptie2	Degradation rate of phosphorylated Tie2	48	s^{-1}	1.30E-04	Fitting
krectie2	Recycling rate of Tie2	47	s^{-1}	1.70E-03	Fitting
ksyntie2	Synthesis rate of Tie2	49	$nM\ s^{-1}$	7.15E-07	Fitting
ksyntie1	Synthesis rate of Tie1	50	$nM\ s^{-1}$	2.62E-02	Fitting

kontie1tie2	Association rate of Tie1:Tie2 binding	64	$nM^{-1} s^{-1}$	6.35E-02	Fitting
kofftie1tie2	Dissociation rate of Tie1:Tie2 binding	64	s^{-1}	7.24E-01	Fitting
kdissang1tie1tie2	Dissociation rate of Tie1:Tie2 when Ang1 is bound	66	s^{-1}	4.82E-01	Fitting
kcleavetie2	Extracellular domain cleavage rate of Tie2	70	s^{-1}	1.46E-03	Fitting
kcleavetie1	Extracellular domain cleavage rate of Tie1	71	s^{-1}	9.97E-03	Fitting
kdegsTie2	Degradation rate of soluble Tie2	72	s^{-1}	1.83E-09	Fitting
kdegstie1	Degradation rate of soluble Tie1	73	s^{-1}	2.25E-02	Fitting
kactPI3KTie2	Activation rate of PI3K Tie2	74	s^{-1}	5.69E+01	Fitting
kinactPI3KTie2	Inactivation rate of PI3K Tie2	75	s^{-1}	5.14E-02	Fitting
kPIP2gen	Generating rate of PIP2	76	s^{-1}	8.62E-04	Fitting
kmPIP2PI3K	Michaelis-Menten reaction rate of PIP2/PI3K	77	nM	3.63E+02	Fitting
kcatPI3KPIP2	Catalytic reaction rate of PIP2/PI3K	77	s^{-1}	6.14E+03	Fitting
kmPIP3PTEN	Michaelis-Menten reaction rate of PTEN/PIP3	78	nM	1.66E+01	Fitting
kcatPTENPIP3	Catalytic reaction rate of PTEN/PIP3	78	s^{-1}	1.17E+04	Fitting
konPDK1PIP3	Association rate of PDK1/PIP3	79	$nM^{-1} s^{-1}$	5.83E+03	Fitting
koffPDK1PIP3	Dissociation rate of PDK1/PIP3	79	s^{-1}	5.68E+00	Fitting
konAKTPIP3	Association rate of Akt/PIP3	80	$nM^{-1} s^{-1}$	2.16E+01	Fitting
koffAKTPIP3	Dissociation rate of Akt/PIP3	80	s^{-1}	1.79E-02	Fitting
kpmTORAKT	Phosphorylation of Akt by mTOR	81	s^{-1}	1.91E+02	Fitting
kpAKTPDK1	Phosphorylation of Akt by PDK1	82	s^{-1}	3.53E+00	Fitting
kdp473AKTPPase	Dephosphorylation of Akt p473 site	83	s^{-1}	1.22E-02	Fitting
kdp308AKTPPase	Dephosphorylation of Akt p308 site	84	s^{-1}	1.25E-02	Fitting
kprhoa	Phosphorylation rate of RhoA	85	s^{-1}	1.00E-01	Fitting
kdprhoa	Dephosphorylation rate of RhoA	86	s^{-1}	6.08E-01	Fitting
konrhoamdia	Association rate of RhoA mDia	87	$nM^{-1} s^{-1}$	4.33E-04	Fitting
koffrhoamdia	Dissociation rate of RhoA mDia	87	s^{-1}	2.74E-02	Fitting
konmdiasrc	Association rate of mDia Src	88	$nM^{-1} s^{-1}$	1.00E+01	Fitting
koffmdiasrc	Dissociation rate of mDia Src	88	s^{-1}	1.00E-01	Fitting
kpsrc	Phosphorylation rate of Src	89	s^{-1}	3.70E-02	Fitting
kdpsrc	Dephosphorylation rate of Src	89	s^{-1}	3.84E-01	Fitting
kpvecad	Phosphorylation rate of VE-Cadherin	90	s^{-1}	1.00E-01	Assumed
kdpvecad	Dephosphorylation rate of VE-Cadherin	90	s^{-1}	1.00E-01	Assumed
kintvecad	Internalization rate of VE-Cadherin	91	s^{-1}	0.1	Assumed
krecvecad	Recycling rate of VE-Cadherin	92	s^{-1}	0.1	Assumed
kdegvecad	Degradation rate of VE-Cadherin	93	s^{-1}	1.00E-01	Assumed
kactabin2	Activation rate of ABIN2	94	s^{-1}	0.1	Assumed
kinactabin2	Inactivation rate of ABIN2	94	s^{-1}	0.1	Assumed

Supplemental Table S2. Table of all observables in the model.

Observable	Description
pTie2	Phosphorylated Tie2
intTie2	Internalized Tie2
jTie2	Tie2 Localized at EC Junctions
ppAkt	Doubly phosphorylated Akt
mDiaSrc	Protein mDia-bound (sequestered) Src
surfTie2	Tie2 at the cell surface
psurfTie2	Phosphorylated Tie2 at cell surface
pintTie2	Internalized phosphorylated Tie2
totalTie2	Total Tie2
sTie2	Soluble Tie2
Tie1Tie2	Tie1:Tie2 heterodimer
surfTie1	Tie1 at the cell surface
sTie1	Soluble Tie1
jTie2	Tie1 at the cell junction
tsurfTie1	Total Tie1 at the cell surface
freepip2	Free PIP2
freepip3	Free PIP3
freeSrc	Free Src
pSrc	Phosphorylated Src
pVECadherin	Phosphorylated VE-Cadherin
aABIN2	Activated ABIN2
totalAkt	Total Akt level
RhoAGTP	Activated (GTP-form) of RhoA
RhoAmDia	mDia-bound RhoA
totalTie1	Total Tie1

Dataset S1. Table of all reactions of the model.

Dataset S2. Systems Biology Markup Language (SBML) of the model reaction network.

Dataset S3. BioNetGen Language (BNGL) file (.bngl) of the model reaction rules.

References

1. Alawo, D.O.A., *Computational modelling of the angiopoietin and tie interactions*, in *Department of Cardiovascular Sciences*. 2017, University of Leicester.
2. Alawo, D.O.A., et al., *Regulation of Angiopoietin Signalling by Soluble Tie2 Ectodomain and Engineered Ligand Trap.* Sci Rep, 2017. **7**(1): p. 3658.
3. Findley, C.M., et al., *VEGF induces Tie2 shedding via a phosphoinositide 3-kinase/Akt dependent pathway to modulate Tie2 signaling.* Arterioscler Thromb Vasc Biol, 2007. **27**(12): p. 2619-26.
4. Findley, C.M., et al., *Plasma levels of soluble Tie2 and vascular endothelial growth factor distinguish critical limb ischemia from intermittent claudication in patients with peripheral arterial disease.* J Am Coll Cardiol, 2008. **52**(5): p. 387-93.
5. Marron, M.B., et al., *Regulated proteolytic processing of Tie1 modulates ligand responsiveness of the receptor-tyrosine kinase Tie2.* J Biol Chem, 2007. **282**(42): p. 30509-17.
6. Bazzazi, H., J.S. Isenberg, and A.S. Popel, *Inhibition of VEGFR2 Activation and Its Downstream Signaling to ERK1/2 and Calcium by Thrombospondin-1 (TSP1): In silico Investigation.* Front Physiol, 2017. **8**: p. 48.
7. Davis, S., et al., *Isolation of angiopoietin-1, a ligand for the TIE2 receptor, by secretion-trap expression cloning.* Cell, 1996. **87**(7): p. 1161-9.
8. Maisonpierre, P.C., et al., *Angiopoietin-2, a natural antagonist for Tie2 that disrupts in vivo angiogenesis.* Science, 1997. **277**(5322): p. 55-60.
