# Supplementary material for: A systems biology model of junctional localization and downstream signaling of the Ang–Tie signaling pathway
Source: NPJ Syst Biol Appl. 2021 Aug 20;7:34. doi: 10.1038/s41540-021-00194-6 (PMC8379279; doi:10.1038/s41540-021-00194-6)
Supplement: Supplementary file 5 — Supplementary Data 2 [file 41540_2021_194_MOESM5_ESM.pdf]

Dataset

Junctional Localization and Downstream Signaling of the Angiopoietin-Tie Signaling Pathway:

A Computational Model

Yu Zhang, Christopher D. Kontos, Brian H. Annex, and Aleksander S. Popel

Dataset S2

Systems Biology Markup Language (SBML) of the model reaction network.

```xml
<?xml version="1.0" encoding="UTF-8"?>
<!-- Created by BioNetGen 2.5.0  -->
<sbml xmlns="http://www.sbml.org/sbml/level2/version3" level="2" version="3">
  <model id="Tie2Model_v200318">
    <listOfCompartments>
      <compartment id="cell" size="1"/>
    </listOfCompartments>
    <listOfSpecies>
      <species id="S1" compartment="cell" initialConcentration="0.0012"
name="Tie2(ang1bs,ang2bs,loc~s,pY~dp,tie1bs,veptpbs)"/>
      <species id="S2" compartment="cell" initialConcentration="1.75"
name="Ang1_4(tie2bs,tie2bs,tie2bs,tie2bs)"/>
      <species id="S3" compartment="cell" initialConcentration="1.3125"
name="Ang2_2(tie2bs,tie2bs)"/>
      <species id="S4" compartment="cell" initialConcentration="1.575"
name="Ang2_3(tie2bs,tie2bs,tie2bs)"/>
      <species id="S5" compartment="cell" initialConcentration="0.28"
name="Ang2_4(tie2bs,tie2bs,tie2bs,tie2bs)"/>
      <species id="S6" compartment="cell" initialConcentration="15"
name="VEPTP(tie2bs)"/>
      <species id="S7" compartment="cell" initialConcentration="1"
name="Tie2(ang1bs,ang2bs,loc~sol,pY~dp,tie1bs,veptpbs)"/>
      <species id="S8" compartment="cell" initialConcentration="1"
name="Tie1(loc~sol,pY~dp,tie2bs)"/>
      <species id="S9" compartment="cell" initialConcentration="0.0006"
name="Tie1(loc~s,pY~dp,tie2bs)"/>
      <species id="S10" compartment="cell" initialConcentration="0.0012"
name="Tie1(loc~s,pY~dp,tie2bs!1).Tie2(ang1bs,ang2bs,loc~s,pY~dp,tie1bs!1,vept
pbs)"/>
      <species id="S11" compartment="cell" initialConcentration="0.1"
name="PTEN(PIP3docking)"/>
      <species id="S12" compartment="cell" initialConcentration="10"
name="PI(PIsite~3P)"/>
      <species id="S13" compartment="cell" initialConcentration="0.1"
name="PI3K(state~inactive)"/>
      <species id="S14" compartment="cell" initialConcentration="0.1"
name="Akt(PHakt,S473~S,T308~S)"/>
      <species id="S15" compartment="cell" initialConcentration="0.1"
name="PDK1(PHpdk1,aktbd)"/>
      <species id="S16" compartment="cell" initialConcentration="0.1"
name="RhoA(G~GDP,mDiabs)"/>
      <species id="S17" compartment="cell" initialConcentration="0.1"
name="mDia(RhoAbs,srcbs)"/>
      <species id="S18" compartment="cell" initialConcentration="0.1"
name="Src(Y1~Y,mDiabs)"/>
      <species id="S19" compartment="cell" initialConcentration="0.1"
name="VECadherin(S665~S,c~j)"/>
      <species id="S20" compartment="cell" initialConcentration="0.1"
name="ABIN2(state~inactive)"/>
      <species id="S21" compartment="cell" initialConcentration="1"
name="I()"/>
      <species id="S22" compartment="cell" initialConcentration="1"
name="Trash()"/>
      <species id="S23" compartment="cell" initialConcentration="0"
name="Ang1_4(tie2bs!1,tie2bs,tie2bs,tie2bs).Tie2(ang1bs!1,ang2bs,loc~s,pY~dp,
tie1bs,veptpbs)"/>
```

```
      <species id="S24" compartment="cell" initialConcentration="0"
name="Ang2_2(tie2bs!1,tie2bs).Tie2(ang1bs,ang2bs!1,loc~s,pY~dp,tie1bs,veptpbs
)"/>
      <species id="S25" compartment="cell" initialConcentration="0"
name="Ang2_3(tie2bs!1,tie2bs,tie2bs).Tie2(ang1bs,ang2bs!1,loc~s,pY~dp,tie1bs,
veptpbs)"/>
      <species id="S26" compartment="cell" initialConcentration="0"
name="Ang2_4(tie2bs!1,tie2bs,tie2bs,tie2bs).Tie2(ang1bs,ang2bs!1,loc~s,pY~dp,
tie1bs,veptpbs)"/>
      <species id="S27" compartment="cell" initialConcentration="0"
name="Tie1(loc~j,pY~dp,tie2bs)"/>
      <species id="S28" compartment="cell" initialConcentration="0"
name="Ang1_4(tie2bs!1,tie2bs,tie2bs,tie2bs).Tie2(ang1bs!1,ang2bs,loc~sol,pY~d
p,tie1bs,veptpbs)"/>
      <species id="S29" compartment="cell" initialConcentration="0"
name="Ang2_2(tie2bs!1,tie2bs).Tie2(ang1bs,ang2bs!1,loc~sol,pY~dp,tie1bs,veptp
bs)"/>
      <species id="S30" compartment="cell" initialConcentration="0"
name="Ang2_3(tie2bs!1,tie2bs,tie2bs).Tie2(ang1bs,ang2bs!1,loc~sol,pY~dp,tie1b
s,veptpbs)"/>
      <species id="S31" compartment="cell" initialConcentration="0"
name="Ang2_4(tie2bs!1,tie2bs,tie2bs,tie2bs).Tie2(ang1bs,ang2bs!1,loc~sol,pY~d
p,tie1bs,veptpbs)"/>
      <species id="S32" compartment="cell" initialConcentration="0"
name="Ang1_4(tie2bs!1,tie2bs,tie2bs,tie2bs).Tie1(loc~s,pY~dp,tie2bs!2).Tie2(a
ng1bs!1,ang2bs,loc~s,pY~dp,tie1bs!2,veptpbs)"/>
      <species id="S33" compartment="cell" initialConcentration="0"
name="Ang2_2(tie2bs!1,tie2bs).Tie1(loc~s,pY~dp,tie2bs!2).Tie2(ang1bs,ang2bs!1
,loc~s,pY~dp,tie1bs!2,veptpbs)"/>
      <species id="S34" compartment="cell" initialConcentration="0"
name="Ang2_3(tie2bs!1,tie2bs,tie2bs).Tie1(loc~s,pY~dp,tie2bs!2).Tie2(ang1bs,a
ng2bs!1,loc~s,pY~dp,tie1bs!2,veptpbs)"/>
      <species id="S35" compartment="cell" initialConcentration="0"
name="Ang2_4(tie2bs!1,tie2bs,tie2bs,tie2bs).Tie1(loc~s,pY~dp,tie2bs!2).Tie2(a
ng1bs,ang2bs!1,loc~s,pY~dp,tie1bs!2,veptpbs)"/>
      <species id="S36" compartment="cell" initialConcentration="0"
name="Src(Y1~pY,mDiabs)"/>
      <species id="S37" compartment="cell" initialConcentration="0"
name="Ang1_4(tie2bs!1,tie2bs!2,tie2bs,tie2bs).Tie2(ang1bs!1,ang2bs,loc~s,pY~d
p,tie1bs,veptpbs).Tie2(ang1bs!2,ang2bs,loc~s,pY~dp,tie1bs,veptpbs)"/>
      <species id="S38" compartment="cell" initialConcentration="0"
name="Ang2_2(tie2bs!1,tie2bs!2).Tie2(ang1bs,ang2bs!1,loc~s,pY~dp,tie1bs,veptp
bs).Tie2(ang1bs,ang2bs!2,loc~s,pY~dp,tie1bs,veptpbs)"/>
      <species id="S39" compartment="cell" initialConcentration="0"
name="Ang2_3(tie2bs!1,tie2bs!2,tie2bs).Tie2(ang1bs,ang2bs!1,loc~s,pY~dp,tie1b
s,veptpbs).Tie2(ang1bs,ang2bs!2,loc~s,pY~dp,tie1bs,veptpbs)"/>
      <species id="S40" compartment="cell" initialConcentration="0"
name="Ang2_4(tie2bs!1,tie2bs!2,tie2bs,tie2bs).Tie2(ang1bs,ang2bs!1,loc~s,pY~d
p,tie1bs,veptpbs).Tie2(ang1bs,ang2bs!2,loc~s,pY~dp,tie1bs,veptpbs)"/>
      <species id="S41" compartment="cell" initialConcentration="0"
name="Tie2(ang1bs,ang2bs,loc~i,pY~dp,tie1bs,veptpbs)"/>
      <species id="S42" compartment="cell" initialConcentration="0"
name="Ang1_4(tie2bs!1,tie2bs!2,tie2bs,tie2bs).Tie2(ang1bs!1,ang2bs,loc~sol,pY
~dp,tie1bs,veptpbs).Tie2(ang1bs!2,ang2bs,loc~sol,pY~dp,tie1bs,veptpbs)"/>
      <species id="S43" compartment="cell" initialConcentration="0"
name="Ang2_2(tie2bs!1,tie2bs!2).Tie2(ang1bs,ang2bs!1,loc~sol,pY~dp,tie1bs,vep
tpbs).Tie2(ang1bs,ang2bs!2,loc~sol,pY~dp,tie1bs,veptpbs)"/>
```

```
      <species id="S44" compartment="cell" initialConcentration="0"
name="Ang2_3(tie2bs!1,tie2bs!2,tie2bs).Tie2(ang1bs,ang2bs!1,loc~sol,pY~dp,tie
1bs,veptpbs).Tie2(ang1bs,ang2bs!2,loc~sol,pY~dp,tie1bs,veptpbs)"/>
      <species id="S45" compartment="cell" initialConcentration="0"
name="Ang2_4(tie2bs!1,tie2bs!2,tie2bs,tie2bs).Tie2(ang1bs,ang2bs!1,loc~sol,pY
~dp,tie1bs,veptpbs).Tie2(ang1bs,ang2bs!2,loc~sol,pY~dp,tie1bs,veptpbs)"/>
      <species id="S46" compartment="cell" initialConcentration="0"
name="VECadherin(S665~pS,c~j)"/>
      <species id="S47" compartment="cell" initialConcentration="0"
name="Ang1_4(tie2bs!1,tie2bs!2,tie2bs!3,tie2bs).Tie2(ang1bs!1,ang2bs,loc~s,pY
~dp,tie1bs,veptpbs).Tie2(ang1bs!2,ang2bs,loc~s,pY~dp,tie1bs,veptpbs).Tie2(ang
1bs!3,ang2bs,loc~s,pY~dp,tie1bs,veptpbs)"/>
      <species id="S48" compartment="cell" initialConcentration="0"
name="Ang2_3(tie2bs!1,tie2bs!2,tie2bs!3).Tie2(ang1bs,ang2bs!1,loc~s,pY~dp,tie
1bs,veptpbs).Tie2(ang1bs,ang2bs!2,loc~s,pY~dp,tie1bs,veptpbs).Tie2(ang1bs,ang
2bs!3,loc~s,pY~dp,tie1bs,veptpbs)"/>
      <species id="S49" compartment="cell" initialConcentration="0"
name="Ang2_4(tie2bs!1,tie2bs!2,tie2bs!3,tie2bs).Tie2(ang1bs,ang2bs!1,loc~s,pY
~dp,tie1bs,veptpbs).Tie2(ang1bs,ang2bs!2,loc~s,pY~dp,tie1bs,veptpbs).Tie2(ang
1bs,ang2bs!3,loc~s,pY~dp,tie1bs,veptpbs)"/>
      <species id="S50" compartment="cell" initialConcentration="0"
name="Ang1_4(tie2bs!1,tie2bs!2,tie2bs!3,tie2bs).Tie2(ang1bs!1,ang2bs,loc~sol,
pY~dp,tie1bs,veptpbs).Tie2(ang1bs!2,ang2bs,loc~sol,pY~dp,tie1bs,veptpbs).Tie2
(ang1bs!3,ang2bs,loc~sol,pY~dp,tie1bs,veptpbs)"/>
      <species id="S51" compartment="cell" initialConcentration="0"
name="Ang2_3(tie2bs!1,tie2bs!2,tie2bs!3).Tie2(ang1bs,ang2bs!1,loc~sol,pY~dp,t
ie1bs,veptpbs).Tie2(ang1bs,ang2bs!2,loc~sol,pY~dp,tie1bs,veptpbs).Tie2(ang1bs
,ang2bs!3,loc~sol,pY~dp,tie1bs,veptpbs)"/>
      <species id="S52" compartment="cell" initialConcentration="0"
name="Ang2_4(tie2bs!1,tie2bs!2,tie2bs!3,tie2bs).Tie2(ang1bs,ang2bs!1,loc~sol,
pY~dp,tie1bs,veptpbs).Tie2(ang1bs,ang2bs!2,loc~sol,pY~dp,tie1bs,veptpbs).Tie2
(ang1bs,ang2bs!3,loc~sol,pY~dp,tie1bs,veptpbs)"/>
      <species id="S53" compartment="cell" initialConcentration="0"
name="VECadherin(S665~pS,c~i)"/>
      <species id="S54" compartment="cell" initialConcentration="0"
name="Ang1_4(tie2bs!1,tie2bs!2,tie2bs!3,tie2bs!4).Tie2(ang1bs!1,ang2bs,loc~s,
pY~dp,tie1bs,veptpbs).Tie2(ang1bs!2,ang2bs,loc~s,pY~dp,tie1bs,veptpbs).Tie2(a
ng1bs!3,ang2bs,loc~s,pY~dp,tie1bs,veptpbs).Tie2(ang1bs!4,ang2bs,loc~s,pY~dp,t
ie1bs,veptpbs)"/>
      <species id="S55" compartment="cell" initialConcentration="0"
name="Ang2_4(tie2bs!1,tie2bs!2,tie2bs!3,tie2bs!4).Tie2(ang1bs,ang2bs!1,loc~s,
pY~dp,tie1bs,veptpbs).Tie2(ang1bs,ang2bs!2,loc~s,pY~dp,tie1bs,veptpbs).Tie2(a
ng1bs,ang2bs!3,loc~s,pY~dp,tie1bs,veptpbs).Tie2(ang1bs,ang2bs!4,loc~s,pY~dp,t
ie1bs,veptpbs)"/>
      <species id="S56" compartment="cell" initialConcentration="0"
name="Ang1_4(tie2bs!1,tie2bs!2,tie2bs!3,tie2bs!4).Tie2(ang1bs!1,ang2bs,loc~so
l,pY~dp,tie1bs,veptpbs).Tie2(ang1bs!2,ang2bs,loc~sol,pY~dp,tie1bs,veptpbs).Ti
e2(ang1bs!3,ang2bs,loc~sol,pY~dp,tie1bs,veptpbs).Tie2(ang1bs!4,ang2bs,loc~sol
,pY~dp,tie1bs,veptpbs)"/>
      <species id="S57" compartment="cell" initialConcentration="0"
name="Ang2_4(tie2bs!1,tie2bs!2,tie2bs!3,tie2bs!4).Tie2(ang1bs,ang2bs!1,loc~so
l,pY~dp,tie1bs,veptpbs).Tie2(ang1bs,ang2bs!2,loc~sol,pY~dp,tie1bs,veptpbs).Ti
e2(ang1bs,ang2bs!3,loc~sol,pY~dp,tie1bs,veptpbs).Tie2(ang1bs,ang2bs!4,loc~sol
,pY~dp,tie1bs,veptpbs)"/>
      <species id="S58" compartment="cell" initialConcentration="0"
name="Ang1_4(tie2bs!1,tie2bs!2,tie2bs!3,tie2bs!4).Tie2(ang1bs!2,ang2bs,loc~s,
pY~dp,tie1bs,veptpbs).Tie2(ang1bs!3,ang2bs,loc~s,pY~dp,tie1bs,veptpbs).Tie2(a
```

```
ng1bs!4,ang2bs,loc~s,pY~dp,tie1bs,veptpbs).Tie2(ang1bs!1,ang2bs,loc~s,pY~p,ti
e1bs,veptpbs)"/>
      <species id="S59" compartment="cell" initialConcentration="0"
name="Ang2_4(tie2bs!1,tie2bs!2,tie2bs!3,tie2bs!4).Tie2(ang1bs,ang2bs!2,loc~s,
pY~dp,tie1bs,veptpbs).Tie2(ang1bs,ang2bs!3,loc~s,pY~dp,tie1bs,veptpbs).Tie2(a
ng1bs,ang2bs!4,loc~s,pY~dp,tie1bs,veptpbs).Tie2(ang1bs,ang2bs!1,loc~s,pY~p,ti
e1bs,veptpbs)"/>
      <species id="S60" compartment="cell" initialConcentration="0"
name="Ang1_4(tie2bs!1,tie2bs!2,tie2bs!3,tie2bs!4).Tie2(ang1bs!3,ang2bs,loc~s,
pY~dp,tie1bs,veptpbs).Tie2(ang1bs!4,ang2bs,loc~s,pY~dp,tie1bs,veptpbs).Tie2(a
ng1bs!2,ang2bs,loc~s,pY~p,tie1bs,veptpbs).Tie2(ang1bs!1,ang2bs,loc~s,pY~p,tie
1bs,veptpbs)"/>
      <species id="S61" compartment="cell" initialConcentration="0"
name="Ang2_4(tie2bs!1,tie2bs!2,tie2bs!3,tie2bs!4).Tie2(ang1bs,ang2bs!3,loc~s,
pY~dp,tie1bs,veptpbs).Tie2(ang1bs,ang2bs!4,loc~s,pY~dp,tie1bs,veptpbs).Tie2(a
ng1bs,ang2bs!2,loc~s,pY~p,tie1bs,veptpbs).Tie2(ang1bs,ang2bs!1,loc~s,pY~p,tie
1bs,veptpbs)"/>
      <species id="S62" compartment="cell" initialConcentration="0"
name="Tie2(ang1bs,ang2bs,loc~i,pY~p,tie1bs,veptpbs)"/>
      <species id="S63" compartment="cell" initialConcentration="0"
name="Ang1_4(tie2bs!1,tie2bs!2,tie2bs!3,tie2bs!4).Tie2(ang1bs!4,ang2bs,loc~s,
pY~dp,tie1bs,veptpbs).Tie2(ang1bs!3,ang2bs,loc~s,pY~p,tie1bs,veptpbs).Tie2(an
g1bs!2,ang2bs,loc~s,pY~p,tie1bs,veptpbs).Tie2(ang1bs!1,ang2bs,loc~s,pY~p,tie1
bs,veptpbs)"/>
      <species id="S64" compartment="cell" initialConcentration="0"
name="Ang2_4(tie2bs!1,tie2bs!2,tie2bs!3,tie2bs!4).Tie2(ang1bs,ang2bs!4,loc~s,
pY~dp,tie1bs,veptpbs).Tie2(ang1bs,ang2bs!3,loc~s,pY~p,tie1bs,veptpbs).Tie2(an
g1bs,ang2bs!2,loc~s,pY~p,tie1bs,veptpbs).Tie2(ang1bs,ang2bs!1,loc~s,pY~p,tie1
bs,veptpbs)"/>
      <species id="S65" compartment="cell" initialConcentration="0"
name="Ang1_4(tie2bs!1,tie2bs!2,tie2bs!3,tie2bs!4).Tie2(ang1bs!4,ang2bs,loc~s,
pY~p,tie1bs,veptpbs).Tie2(ang1bs!3,ang2bs,loc~s,pY~p,tie1bs,veptpbs).Tie2(ang
1bs!2,ang2bs,loc~s,pY~p,tie1bs,veptpbs).Tie2(ang1bs!1,ang2bs,loc~s,pY~p,tie1b
s,veptpbs)"/>
      <species id="S66" compartment="cell" initialConcentration="0"
name="Ang2_4(tie2bs!1,tie2bs!2,tie2bs!3,tie2bs!4).Tie2(ang1bs,ang2bs!4,loc~s,
pY~p,tie1bs,veptpbs).Tie2(ang1bs,ang2bs!3,loc~s,pY~p,tie1bs,veptpbs).Tie2(ang
1bs,ang2bs!2,loc~s,pY~p,tie1bs,veptpbs).Tie2(ang1bs,ang2bs!1,loc~s,pY~p,tie1b
s,veptpbs)"/>
      <species id="S67" compartment="cell" initialConcentration="0"
name="Ang1_4(tie2bs!1,tie2bs!2,tie2bs!3,tie2bs!4).Tie2(ang1bs!4,ang2bs,loc~j,
pY~p,tie1bs,veptpbs).Tie2(ang1bs!3,ang2bs,loc~j,pY~p,tie1bs,veptpbs).Tie2(ang
1bs!2,ang2bs,loc~j,pY~p,tie1bs,veptpbs).Tie2(ang1bs!1,ang2bs,loc~j,pY~p,tie1b
s,veptpbs)"/>
      <species id="S68" compartment="cell" initialConcentration="0"
name="Ang2_4(tie2bs!1,tie2bs!2,tie2bs!3,tie2bs!4).Tie2(ang1bs,ang2bs!4,loc~j,
pY~p,tie1bs,veptpbs).Tie2(ang1bs,ang2bs!3,loc~j,pY~p,tie1bs,veptpbs).Tie2(ang
1bs,ang2bs!2,loc~j,pY~p,tie1bs,veptpbs).Tie2(ang1bs,ang2bs!1,loc~j,pY~p,tie1b
s,veptpbs)"/>
      <species id="S69" compartment="cell" initialConcentration="0"
name="Ang1_4(tie2bs!1,tie2bs!2,tie2bs!3,tie2bs!4).Tie1(loc~j,pY~dp,tie2bs!5).
Tie2(ang1bs!1,ang2bs,loc~j,pY~p,tie1bs!5,veptpbs).Tie2(ang1bs!4,ang2bs,loc~j,
pY~p,tie1bs,veptpbs).Tie2(ang1bs!3,ang2bs,loc~j,pY~p,tie1bs,veptpbs).Tie2(ang
1bs!2,ang2bs,loc~j,pY~p,tie1bs,veptpbs)"/>
      <species id="S70" compartment="cell" initialConcentration="0"
name="Ang2_4(tie2bs!1,tie2bs!2,tie2bs!3,tie2bs!4).Tie1(loc~j,pY~dp,tie2bs!5).
Tie2(ang1bs,ang2bs!1,loc~j,pY~p,tie1bs!5,veptpbs).Tie2(ang1bs,ang2bs!4,loc~j,
```

```
pY~p,tie1bs,veptpbs).Tie2(ang1bs,ang2bs!3,loc~j,pY~p,tie1bs,veptpbs).Tie2(ang
1bs,ang2bs!2,loc~j,pY~p,tie1bs,veptpbs)"/>
      <species id="S71" compartment="cell" initialConcentration="0"
name="Ang1_4(tie2bs!1,tie2bs!2,tie2bs!3,tie2bs!4).Tie2(ang1bs!4,ang2bs,loc~j,
pY~p,tie1bs,veptpbs!5).Tie2(ang1bs!3,ang2bs,loc~j,pY~p,tie1bs,veptpbs).Tie2(a
ng1bs!2,ang2bs,loc~j,pY~p,tie1bs,veptpbs).Tie2(ang1bs!1,ang2bs,loc~j,pY~p,tie
1bs,veptpbs).VEPTP(tie2bs!5)"/>
      <species id="S72" compartment="cell" initialConcentration="0"
name="Ang2_4(tie2bs!1,tie2bs!2,tie2bs!3,tie2bs!4).Tie2(ang1bs,ang2bs!4,loc~j,
pY~p,tie1bs,veptpbs!5).Tie2(ang1bs,ang2bs!3,loc~j,pY~p,tie1bs,veptpbs).Tie2(a
ng1bs,ang2bs!2,loc~j,pY~p,tie1bs,veptpbs).Tie2(ang1bs,ang2bs!1,loc~j,pY~p,tie
1bs,veptpbs).VEPTP(tie2bs!5)"/>
      <species id="S73" compartment="cell" initialConcentration="0"
name="PI3K(state~active)"/>
      <species id="S74" compartment="cell" initialConcentration="0"
name="RhoA(G~GTP,mDiabs)"/>
      <species id="S75" compartment="cell" initialConcentration="0"
name="ABIN2(state~active)"/>
      <species id="S76" compartment="cell" initialConcentration="0"
name="Ang1_4(tie2bs!1,tie2bs!2,tie2bs!3,tie2bs!4).Tie1(loc~j,pY~p,tie2bs!5).T
ie2(ang1bs!1,ang2bs,loc~j,pY~p,tie1bs!5,veptpbs).Tie2(ang1bs!4,ang2bs,loc~j,p
Y~p,tie1bs,veptpbs).Tie2(ang1bs!3,ang2bs,loc~j,pY~p,tie1bs,veptpbs).Tie2(ang1
bs!2,ang2bs,loc~j,pY~p,tie1bs,veptpbs)"/>
      <species id="S77" compartment="cell" initialConcentration="0"
name="Ang2_4(tie2bs!1,tie2bs!2,tie2bs!3,tie2bs!4).Tie1(loc~j,pY~p,tie2bs!5).T
ie2(ang1bs,ang2bs!1,loc~j,pY~p,tie1bs!5,veptpbs).Tie2(ang1bs,ang2bs!4,loc~j,p
Y~p,tie1bs,veptpbs).Tie2(ang1bs,ang2bs!3,loc~j,pY~p,tie1bs,veptpbs).Tie2(ang1
bs,ang2bs!2,loc~j,pY~p,tie1bs,veptpbs)"/>
      <species id="S78" compartment="cell" initialConcentration="0"
name="Ang1_4(tie2bs!1,tie2bs!2,tie2bs!3,tie2bs!4).Tie1(loc~j,pY~dp,tie2bs!5).
Tie2(ang1bs!1,ang2bs,loc~j,pY~p,tie1bs!5,veptpbs).Tie2(ang1bs!4,ang2bs,loc~j,
pY~p,tie1bs,veptpbs!6).Tie2(ang1bs!3,ang2bs,loc~j,pY~p,tie1bs,veptpbs).Tie2(a
ng1bs!2,ang2bs,loc~j,pY~p,tie1bs,veptpbs).VEPTP(tie2bs!6)"/>
      <species id="S79" compartment="cell" initialConcentration="0"
name="Ang2_4(tie2bs!1,tie2bs!2,tie2bs!3,tie2bs!4).Tie1(loc~j,pY~dp,tie2bs!5).
Tie2(ang1bs,ang2bs!1,loc~j,pY~p,tie1bs!5,veptpbs).Tie2(ang1bs,ang2bs!4,loc~j,
pY~p,tie1bs,veptpbs!6).Tie2(ang1bs,ang2bs!3,loc~j,pY~p,tie1bs,veptpbs).Tie2(a
ng1bs,ang2bs!2,loc~j,pY~p,tie1bs,veptpbs).VEPTP(tie2bs!6)"/>
      <species id="S80" compartment="cell" initialConcentration="0"
name="Ang1_4(tie2bs!1,tie2bs!2,tie2bs!3,tie2bs!4).Tie2(ang1bs!4,ang2bs,loc~j,
pY~p,tie1bs,veptpbs!5).Tie2(ang1bs!3,ang2bs,loc~j,pY~p,tie1bs,veptpbs!6).Tie2
(ang1bs!2,ang2bs,loc~j,pY~p,tie1bs,veptpbs).Tie2(ang1bs!1,ang2bs,loc~j,pY~p,t
ie1bs,veptpbs).VEPTP(tie2bs!5).VEPTP(tie2bs!6)"/>
      <species id="S81" compartment="cell" initialConcentration="0"
name="Ang2_4(tie2bs!1,tie2bs!2,tie2bs!3,tie2bs!4).Tie2(ang1bs,ang2bs!4,loc~j,
pY~p,tie1bs,veptpbs!5).Tie2(ang1bs,ang2bs!3,loc~j,pY~p,tie1bs,veptpbs!6).Tie2
(ang1bs,ang2bs!2,loc~j,pY~p,tie1bs,veptpbs).Tie2(ang1bs,ang2bs!1,loc~j,pY~p,t
ie1bs,veptpbs).VEPTP(tie2bs!5).VEPTP(tie2bs!6)"/>
      <species id="S82" compartment="cell" initialConcentration="0"
name="Ang1_4(tie2bs!1,tie2bs!2,tie2bs!3,tie2bs!4).Tie2(ang1bs!4,ang2bs,loc~j,
pY~dp,tie1bs,veptpbs).Tie2(ang1bs!3,ang2bs,loc~j,pY~p,tie1bs,veptpbs).Tie2(an
g1bs!2,ang2bs,loc~j,pY~p,tie1bs,veptpbs).Tie2(ang1bs!1,ang2bs,loc~j,pY~p,tie1
bs,veptpbs)"/>
      <species id="S83" compartment="cell" initialConcentration="0"
name="Ang2_4(tie2bs!1,tie2bs!2,tie2bs!3,tie2bs!4).Tie2(ang1bs,ang2bs!4,loc~j,
pY~dp,tie1bs,veptpbs).Tie2(ang1bs,ang2bs!3,loc~j,pY~p,tie1bs,veptpbs).Tie2(an
```

```
g1bs,ang2bs!2,loc~j,pY~p,tie1bs,veptpbs).Tie2(ang1bs,ang2bs!1,loc~j,pY~p,tie1
bs,veptpbs)"/>
      <species id="S84" compartment="cell" initialConcentration="0"
name="PI(PIsite~4P)"/>
      <species id="S85" compartment="cell" initialConcentration="0"
name="RhoA(G~GTP,mDiabs!1).mDia(RhoAbs!1,srcbs)"/>
      <species id="S86" compartment="cell" initialConcentration="0"
name="Ang1_4(tie2bs!1,tie2bs!2,tie2bs!3,tie2bs!4).Tie1(loc~j,pY~p,tie2bs!5).T
ie2(ang1bs!1,ang2bs,loc~j,pY~p,tie1bs!5,veptpbs).Tie2(ang1bs!4,ang2bs,loc~j,p
Y~p,tie1bs,veptpbs!6).Tie2(ang1bs!3,ang2bs,loc~j,pY~p,tie1bs,veptpbs).Tie2(an
g1bs!2,ang2bs,loc~j,pY~p,tie1bs,veptpbs).VEPTP(tie2bs!6)"/>
      <species id="S87" compartment="cell" initialConcentration="0"
name="Ang2_4(tie2bs!1,tie2bs!2,tie2bs!3,tie2bs!4).Tie1(loc~j,pY~p,tie2bs!5).T
ie2(ang1bs,ang2bs!1,loc~j,pY~p,tie1bs!5,veptpbs).Tie2(ang1bs,ang2bs!4,loc~j,p
Y~p,tie1bs,veptpbs!6).Tie2(ang1bs,ang2bs!3,loc~j,pY~p,tie1bs,veptpbs).Tie2(an
g1bs,ang2bs!2,loc~j,pY~p,tie1bs,veptpbs).VEPTP(tie2bs!6)"/>
      <species id="S88" compartment="cell" initialConcentration="0"
name="Ang1_4(tie2bs!1,tie2bs!2,tie2bs!3,tie2bs!4).Tie1(loc~j,pY~dp,tie2bs!5).
Tie2(ang1bs!1,ang2bs,loc~j,pY~p,tie1bs!5,veptpbs).Tie2(ang1bs!4,ang2bs,loc~j,
pY~p,tie1bs,veptpbs!6).Tie2(ang1bs!3,ang2bs,loc~j,pY~p,tie1bs,veptpbs!7).Tie2
(ang1bs!2,ang2bs,loc~j,pY~p,tie1bs,veptpbs).VEPTP(tie2bs!6).VEPTP(tie2bs!7)"/
>
      <species id="S89" compartment="cell" initialConcentration="0"
name="Ang2_4(tie2bs!1,tie2bs!2,tie2bs!3,tie2bs!4).Tie1(loc~j,pY~dp,tie2bs!5).
Tie2(ang1bs,ang2bs!1,loc~j,pY~p,tie1bs!5,veptpbs).Tie2(ang1bs,ang2bs!4,loc~j,
pY~p,tie1bs,veptpbs!6).Tie2(ang1bs,ang2bs!3,loc~j,pY~p,tie1bs,veptpbs!7).Tie2
(ang1bs,ang2bs!2,loc~j,pY~p,tie1bs,veptpbs).VEPTP(tie2bs!6).VEPTP(tie2bs!7)"/
>
      <species id="S90" compartment="cell" initialConcentration="0"
name="Ang1_4(tie2bs!1,tie2bs!2,tie2bs!3,tie2bs!4).Tie2(ang1bs!4,ang2bs,loc~j,
pY~p,tie1bs,veptpbs!5).Tie2(ang1bs!3,ang2bs,loc~j,pY~p,tie1bs,veptpbs!6).Tie2
(ang1bs!2,ang2bs,loc~j,pY~p,tie1bs,veptpbs!7).Tie2(ang1bs!1,ang2bs,loc~j,pY~p
,tie1bs,veptpbs).VEPTP(tie2bs!5).VEPTP(tie2bs!6).VEPTP(tie2bs!7)"/>
      <species id="S91" compartment="cell" initialConcentration="0"
name="Ang2_4(tie2bs!1,tie2bs!2,tie2bs!3,tie2bs!4).Tie2(ang1bs,ang2bs!4,loc~j,
pY~p,tie1bs,veptpbs!5).Tie2(ang1bs,ang2bs!3,loc~j,pY~p,tie1bs,veptpbs!6).Tie2
(ang1bs,ang2bs!2,loc~j,pY~p,tie1bs,veptpbs!7).Tie2(ang1bs,ang2bs!1,loc~j,pY~p
,tie1bs,veptpbs).VEPTP(tie2bs!5).VEPTP(tie2bs!6).VEPTP(tie2bs!7)"/>
      <species id="S92" compartment="cell" initialConcentration="0"
name="Ang1_4(tie2bs!1,tie2bs!2,tie2bs!3,tie2bs!4).Tie2(ang1bs!4,ang2bs,loc~j,
pY~dp,tie1bs,veptpbs).Tie2(ang1bs!3,ang2bs,loc~j,pY~p,tie1bs,veptpbs!5).Tie2(
ang1bs!2,ang2bs,loc~j,pY~p,tie1bs,veptpbs).Tie2(ang1bs!1,ang2bs,loc~j,pY~p,ti
e1bs,veptpbs).VEPTP(tie2bs!5)"/>
      <species id="S93" compartment="cell" initialConcentration="0"
name="Ang2_4(tie2bs!1,tie2bs!2,tie2bs!3,tie2bs!4).Tie2(ang1bs,ang2bs!4,loc~j,
pY~dp,tie1bs,veptpbs).Tie2(ang1bs,ang2bs!3,loc~j,pY~p,tie1bs,veptpbs!5).Tie2(
ang1bs,ang2bs!2,loc~j,pY~p,tie1bs,veptpbs).Tie2(ang1bs,ang2bs!1,loc~j,pY~p,ti
e1bs,veptpbs).VEPTP(tie2bs!5)"/>
      <species id="S94" compartment="cell" initialConcentration="0"
name="Ang1_4(tie2bs!1,tie2bs!2,tie2bs!3,tie2bs!4).Tie1(loc~j,pY~dp,tie2bs!5).
Tie2(ang1bs!4,ang2bs,loc~j,pY~dp,tie1bs,veptpbs).Tie2(ang1bs!1,ang2bs,loc~j,p
Y~p,tie1bs!5,veptpbs).Tie2(ang1bs!3,ang2bs,loc~j,pY~p,tie1bs,veptpbs).Tie2(an
g1bs!2,ang2bs,loc~j,pY~p,tie1bs,veptpbs)"/>
      <species id="S95" compartment="cell" initialConcentration="0"
name="Ang2_4(tie2bs!1,tie2bs!2,tie2bs!3,tie2bs!4).Tie1(loc~j,pY~dp,tie2bs!5).
Tie2(ang1bs,ang2bs!4,loc~j,pY~dp,tie1bs,veptpbs).Tie2(ang1bs,ang2bs!1,loc~j,p
```

```
Y~p,tie1bs!5,veptpbs).Tie2(ang1bs,ang2bs!3,loc~j,pY~p,tie1bs,veptpbs).Tie2(an
g1bs,ang2bs!2,loc~j,pY~p,tie1bs,veptpbs)"/>
      <species id="S96" compartment="cell" initialConcentration="0"
name="PDK1(PHpdk1!1,aktbd).PI(PIsite~4P!1)"/>
      <species id="S97" compartment="cell" initialConcentration="0"
name="Akt(PHakt!1,S473~S,T308~S).PI(PIsite~4P!1)"/>
      <species id="S98" compartment="cell" initialConcentration="0"
name="RhoA(G~GTP,mDiabs!1).Src(Y1~Y,mDiabs!2).mDia(RhoAbs!1,srcbs!2)"/>
      <species id="S99" compartment="cell" initialConcentration="0"
name="Ang1_4(tie2bs!1,tie2bs!2,tie2bs!3,tie2bs!4).Tie1(loc~j,pY~p,tie2bs!5).T
ie2(ang1bs!1,ang2bs,loc~j,pY~p,tie1bs!5,veptpbs).Tie2(ang1bs!4,ang2bs,loc~j,p
Y~p,tie1bs,veptpbs!6).Tie2(ang1bs!3,ang2bs,loc~j,pY~p,tie1bs,veptpbs!7).Tie2(
ang1bs!2,ang2bs,loc~j,pY~p,tie1bs,veptpbs).VEPTP(tie2bs!6).VEPTP(tie2bs!7)"/>
      <species id="S100" compartment="cell" initialConcentration="0"
name="Ang2_4(tie2bs!1,tie2bs!2,tie2bs!3,tie2bs!4).Tie1(loc~j,pY~p,tie2bs!5).T
ie2(ang1bs,ang2bs!1,loc~j,pY~p,tie1bs!5,veptpbs).Tie2(ang1bs,ang2bs!4,loc~j,p
Y~p,tie1bs,veptpbs!6).Tie2(ang1bs,ang2bs!3,loc~j,pY~p,tie1bs,veptpbs!7).Tie2(
ang1bs,ang2bs!2,loc~j,pY~p,tie1bs,veptpbs).VEPTP(tie2bs!6).VEPTP(tie2bs!7)"/>
      <species id="S101" compartment="cell" initialConcentration="0"
name="Ang1_4(tie2bs!1,tie2bs!2,tie2bs!3,tie2bs!4).Tie1(loc~j,pY~dp,tie2bs!5).
Tie2(ang1bs!1,ang2bs,loc~j,pY~p,tie1bs!5,veptpbs).Tie2(ang1bs!4,ang2bs,loc~j,
pY~p,tie1bs,veptpbs!6).Tie2(ang1bs!3,ang2bs,loc~j,pY~p,tie1bs,veptpbs!7).Tie2
(ang1bs!2,ang2bs,loc~j,pY~p,tie1bs,veptpbs!8).VEPTP(tie2bs!6).VEPTP(tie2bs!7)
.VEPTP(tie2bs!8)"/>
      <species id="S102" compartment="cell" initialConcentration="0"
name="Ang2_4(tie2bs!1,tie2bs!2,tie2bs!3,tie2bs!4).Tie1(loc~j,pY~dp,tie2bs!5).
Tie2(ang1bs,ang2bs!1,loc~j,pY~p,tie1bs!5,veptpbs).Tie2(ang1bs,ang2bs!4,loc~j,
pY~p,tie1bs,veptpbs!6).Tie2(ang1bs,ang2bs!3,loc~j,pY~p,tie1bs,veptpbs!7).Tie2
(ang1bs,ang2bs!2,loc~j,pY~p,tie1bs,veptpbs!8).VEPTP(tie2bs!6).VEPTP(tie2bs!7)
.VEPTP(tie2bs!8)"/>
      <species id="S103" compartment="cell" initialConcentration="0"
name="Ang1_4(tie2bs!1,tie2bs!2,tie2bs!3,tie2bs!4).Tie2(ang1bs!4,ang2bs,loc~j,
pY~p,tie1bs,veptpbs!5).Tie2(ang1bs!3,ang2bs,loc~j,pY~p,tie1bs,veptpbs!6).Tie2
(ang1bs!2,ang2bs,loc~j,pY~p,tie1bs,veptpbs!7).Tie2(ang1bs!1,ang2bs,loc~j,pY~p
,tie1bs,veptpbs!8).VEPTP(tie2bs!5).VEPTP(tie2bs!6).VEPTP(tie2bs!7).VEPTP(tie2
bs!8)"/>
      <species id="S104" compartment="cell" initialConcentration="0"
name="Ang2_4(tie2bs!1,tie2bs!2,tie2bs!3,tie2bs!4).Tie2(ang1bs,ang2bs!4,loc~j,
pY~p,tie1bs,veptpbs!5).Tie2(ang1bs,ang2bs!3,loc~j,pY~p,tie1bs,veptpbs!6).Tie2
(ang1bs,ang2bs!2,loc~j,pY~p,tie1bs,veptpbs!7).Tie2(ang1bs,ang2bs!1,loc~j,pY~p
,tie1bs,veptpbs!8).VEPTP(tie2bs!5).VEPTP(tie2bs!6).VEPTP(tie2bs!7).VEPTP(tie2
bs!8)"/>
      <species id="S105" compartment="cell" initialConcentration="0"
name="Ang1_4(tie2bs!1,tie2bs!2,tie2bs!3,tie2bs!4).Tie2(ang1bs!4,ang2bs,loc~j,
pY~dp,tie1bs,veptpbs).Tie2(ang1bs!3,ang2bs,loc~j,pY~p,tie1bs,veptpbs!5).Tie2(
ang1bs!2,ang2bs,loc~j,pY~p,tie1bs,veptpbs!6).Tie2(ang1bs!1,ang2bs,loc~j,pY~p,
tie1bs,veptpbs).VEPTP(tie2bs!5).VEPTP(tie2bs!6)"/>
      <species id="S106" compartment="cell" initialConcentration="0"
name="Ang2_4(tie2bs!1,tie2bs!2,tie2bs!3,tie2bs!4).Tie2(ang1bs,ang2bs!4,loc~j,
pY~dp,tie1bs,veptpbs).Tie2(ang1bs,ang2bs!3,loc~j,pY~p,tie1bs,veptpbs!5).Tie2(
ang1bs,ang2bs!2,loc~j,pY~p,tie1bs,veptpbs!6).Tie2(ang1bs,ang2bs!1,loc~j,pY~p,
tie1bs,veptpbs).VEPTP(tie2bs!5).VEPTP(tie2bs!6)"/>
      <species id="S107" compartment="cell" initialConcentration="0"
name="Ang1_4(tie2bs!1,tie2bs!2,tie2bs!3,tie2bs!4).Tie1(loc~j,pY~dp,tie2bs!5).
Tie2(ang1bs!4,ang2bs,loc~j,pY~dp,tie1bs,veptpbs).Tie2(ang1bs!1,ang2bs,loc~j,p
Y~p,tie1bs!5,veptpbs).Tie2(ang1bs!3,ang2bs,loc~j,pY~p,tie1bs,veptpbs!6).Tie2(
ang1bs!2,ang2bs,loc~j,pY~p,tie1bs,veptpbs).VEPTP(tie2bs!6)"/>
```

```
      <species id="S108" compartment="cell" initialConcentration="0"
name="Ang2_4(tie2bs!1,tie2bs!2,tie2bs!3,tie2bs!4).Tie1(loc~j,pY~dp,tie2bs!5).
Tie2(ang1bs,ang2bs!4,loc~j,pY~dp,tie1bs,veptpbs).Tie2(ang1bs,ang2bs!1,loc~j,p
Y~p,tie1bs!5,veptpbs).Tie2(ang1bs,ang2bs!3,loc~j,pY~p,tie1bs,veptpbs!6).Tie2(
ang1bs,ang2bs!2,loc~j,pY~p,tie1bs,veptpbs).VEPTP(tie2bs!6)"/>
      <species id="S109" compartment="cell" initialConcentration="0"
name="Ang1_4(tie2bs!1,tie2bs!2,tie2bs!3,tie2bs!4).Tie1(loc~j,pY~p,tie2bs!5).T
ie2(ang1bs!4,ang2bs,loc~j,pY~dp,tie1bs,veptpbs).Tie2(ang1bs!1,ang2bs,loc~j,pY
~p,tie1bs!5,veptpbs).Tie2(ang1bs!3,ang2bs,loc~j,pY~p,tie1bs,veptpbs).Tie2(ang
1bs!2,ang2bs,loc~j,pY~p,tie1bs,veptpbs)"/>
      <species id="S110" compartment="cell" initialConcentration="0"
name="Ang1_4(tie2bs!1,tie2bs!2,tie2bs!3,tie2bs!4).Tie2(ang1bs!4,ang2bs,loc~j,
pY~dp,tie1bs,veptpbs).Tie2(ang1bs!3,ang2bs,loc~j,pY~dp,tie1bs,veptpbs).Tie2(a
ng1bs!2,ang2bs,loc~j,pY~p,tie1bs,veptpbs).Tie2(ang1bs!1,ang2bs,loc~j,pY~p,tie
1bs,veptpbs)"/>
      <species id="S111" compartment="cell" initialConcentration="0"
name="Ang2_4(tie2bs!1,tie2bs!2,tie2bs!3,tie2bs!4).Tie1(loc~j,pY~p,tie2bs!5).T
ie2(ang1bs,ang2bs!4,loc~j,pY~dp,tie1bs,veptpbs).Tie2(ang1bs,ang2bs!1,loc~j,pY
~p,tie1bs!5,veptpbs).Tie2(ang1bs,ang2bs!3,loc~j,pY~p,tie1bs,veptpbs).Tie2(ang
1bs,ang2bs!2,loc~j,pY~p,tie1bs,veptpbs)"/>
      <species id="S112" compartment="cell" initialConcentration="0"
name="Ang2_4(tie2bs!1,tie2bs!2,tie2bs!3,tie2bs!4).Tie2(ang1bs,ang2bs!4,loc~j,
pY~dp,tie1bs,veptpbs).Tie2(ang1bs,ang2bs!3,loc~j,pY~dp,tie1bs,veptpbs).Tie2(a
ng1bs,ang2bs!2,loc~j,pY~p,tie1bs,veptpbs).Tie2(ang1bs,ang2bs!1,loc~j,pY~p,tie
1bs,veptpbs)"/>
      <species id="S113" compartment="cell" initialConcentration="0"
name="Akt(PHakt!1,S473~pS,T308~S).PI(PIsite~4P!1)"/>
      <species id="S114" compartment="cell" initialConcentration="0"
name="Ang1_4(tie2bs!1,tie2bs!2,tie2bs!3,tie2bs!4).Tie1(loc~j,pY~p,tie2bs!5).T
ie2(ang1bs!1,ang2bs,loc~j,pY~p,tie1bs!5,veptpbs).Tie2(ang1bs!4,ang2bs,loc~j,p
Y~p,tie1bs,veptpbs!6).Tie2(ang1bs!3,ang2bs,loc~j,pY~p,tie1bs,veptpbs!7).Tie2(
ang1bs!2,ang2bs,loc~j,pY~p,tie1bs,veptpbs!8).VEPTP(tie2bs!6).VEPTP(tie2bs!7).
VEPTP(tie2bs!8)"/>
      <species id="S115" compartment="cell" initialConcentration="0"
name="Ang2_4(tie2bs!1,tie2bs!2,tie2bs!3,tie2bs!4).Tie1(loc~j,pY~p,tie2bs!5).T
ie2(ang1bs,ang2bs!1,loc~j,pY~p,tie1bs!5,veptpbs).Tie2(ang1bs,ang2bs!4,loc~j,p
Y~p,tie1bs,veptpbs!6).Tie2(ang1bs,ang2bs!3,loc~j,pY~p,tie1bs,veptpbs!7).Tie2(
ang1bs,ang2bs!2,loc~j,pY~p,tie1bs,veptpbs!8).VEPTP(tie2bs!6).VEPTP(tie2bs!7).
VEPTP(tie2bs!8)"/>
      <species id="S116" compartment="cell" initialConcentration="0"
name="Ang1_4(tie2bs!1,tie2bs!2,tie2bs!3,tie2bs!4).Tie2(ang1bs!4,ang2bs,loc~j,
pY~dp,tie1bs,veptpbs).Tie2(ang1bs!3,ang2bs,loc~j,pY~p,tie1bs,veptpbs!5).Tie2(
ang1bs!2,ang2bs,loc~j,pY~p,tie1bs,veptpbs!6).Tie2(ang1bs!1,ang2bs,loc~j,pY~p,
tie1bs,veptpbs!7).VEPTP(tie2bs!5).VEPTP(tie2bs!6).VEPTP(tie2bs!7)"/>
      <species id="S117" compartment="cell" initialConcentration="0"
name="Ang2_4(tie2bs!1,tie2bs!2,tie2bs!3,tie2bs!4).Tie2(ang1bs,ang2bs!4,loc~j,
pY~dp,tie1bs,veptpbs).Tie2(ang1bs,ang2bs!3,loc~j,pY~p,tie1bs,veptpbs!5).Tie2(
ang1bs,ang2bs!2,loc~j,pY~p,tie1bs,veptpbs!6).Tie2(ang1bs,ang2bs!1,loc~j,pY~p,
tie1bs,veptpbs!7).VEPTP(tie2bs!5).VEPTP(tie2bs!6).VEPTP(tie2bs!7)"/>
      <species id="S118" compartment="cell" initialConcentration="0"
name="Ang1_4(tie2bs!1,tie2bs!2,tie2bs!3,tie2bs!4).Tie1(loc~j,pY~dp,tie2bs!5).
Tie2(ang1bs!4,ang2bs,loc~j,pY~dp,tie1bs,veptpbs).Tie2(ang1bs!1,ang2bs,loc~j,p
Y~p,tie1bs!5,veptpbs).Tie2(ang1bs!3,ang2bs,loc~j,pY~p,tie1bs,veptpbs!6).Tie2(
ang1bs!2,ang2bs,loc~j,pY~p,tie1bs,veptpbs!7).VEPTP(tie2bs!6).VEPTP(tie2bs!7)"
/>
      <species id="S119" compartment="cell" initialConcentration="0"
name="Ang2_4(tie2bs!1,tie2bs!2,tie2bs!3,tie2bs!4).Tie1(loc~j,pY~dp,tie2bs!5).
```

```
Tie2(ang1bs,ang2bs!4,loc~j,pY~dp,tie1bs,veptpbs).Tie2(ang1bs,ang2bs!1,loc~j,p
Y~p,tie1bs!5,veptpbs).Tie2(ang1bs,ang2bs!3,loc~j,pY~p,tie1bs,veptpbs!6).Tie2(
ang1bs,ang2bs!2,loc~j,pY~p,tie1bs,veptpbs!7).VEPTP(tie2bs!6).VEPTP(tie2bs!7)"
/>
      <species id="S120" compartment="cell" initialConcentration="0"
name="Ang1_4(tie2bs!1,tie2bs!2,tie2bs!3,tie2bs!4).Tie1(loc~j,pY~p,tie2bs!5).T
ie2(ang1bs!4,ang2bs,loc~j,pY~dp,tie1bs,veptpbs).Tie2(ang1bs!1,ang2bs,loc~j,pY
~p,tie1bs!5,veptpbs).Tie2(ang1bs!3,ang2bs,loc~j,pY~p,tie1bs,veptpbs!6).Tie2(a
ng1bs!2,ang2bs,loc~j,pY~p,tie1bs,veptpbs).VEPTP(tie2bs!6)"/>
      <species id="S121" compartment="cell" initialConcentration="0"
name="Ang1_4(tie2bs!1,tie2bs!2,tie2bs!3,tie2bs!4).Tie2(ang1bs!4,ang2bs,loc~j,
pY~dp,tie1bs,veptpbs).Tie2(ang1bs!3,ang2bs,loc~j,pY~dp,tie1bs,veptpbs).Tie2(a
ng1bs!2,ang2bs,loc~j,pY~p,tie1bs,veptpbs!5).Tie2(ang1bs!1,ang2bs,loc~j,pY~p,t
ie1bs,veptpbs).VEPTP(tie2bs!5)"/>
      <species id="S122" compartment="cell" initialConcentration="0"
name="Ang2_4(tie2bs!1,tie2bs!2,tie2bs!3,tie2bs!4).Tie1(loc~j,pY~p,tie2bs!5).T
ie2(ang1bs,ang2bs!4,loc~j,pY~dp,tie1bs,veptpbs).Tie2(ang1bs,ang2bs!1,loc~j,pY
~p,tie1bs!5,veptpbs).Tie2(ang1bs,ang2bs!3,loc~j,pY~p,tie1bs,veptpbs!6).Tie2(a
ng1bs,ang2bs!2,loc~j,pY~p,tie1bs,veptpbs).VEPTP(tie2bs!6)"/>
      <species id="S123" compartment="cell" initialConcentration="0"
name="Ang2_4(tie2bs!1,tie2bs!2,tie2bs!3,tie2bs!4).Tie2(ang1bs,ang2bs!4,loc~j,
pY~dp,tie1bs,veptpbs).Tie2(ang1bs,ang2bs!3,loc~j,pY~dp,tie1bs,veptpbs).Tie2(a
ng1bs,ang2bs!2,loc~j,pY~p,tie1bs,veptpbs!5).Tie2(ang1bs,ang2bs!1,loc~j,pY~p,t
ie1bs,veptpbs).VEPTP(tie2bs!5)"/>
      <species id="S124" compartment="cell" initialConcentration="0"
name="Ang1_4(tie2bs!1,tie2bs!2,tie2bs!3,tie2bs!4).Tie1(loc~j,pY~dp,tie2bs!5).
Tie2(ang1bs!4,ang2bs,loc~j,pY~dp,tie1bs,veptpbs).Tie2(ang1bs!3,ang2bs,loc~j,p
Y~dp,tie1bs,veptpbs).Tie2(ang1bs!1,ang2bs,loc~j,pY~p,tie1bs!5,veptpbs).Tie2(a
ng1bs!2,ang2bs,loc~j,pY~p,tie1bs,veptpbs)"/>
      <species id="S125" compartment="cell" initialConcentration="0"
name="Ang2_4(tie2bs!1,tie2bs!2,tie2bs!3,tie2bs!4).Tie1(loc~j,pY~dp,tie2bs!5).
Tie2(ang1bs,ang2bs!4,loc~j,pY~dp,tie1bs,veptpbs).Tie2(ang1bs,ang2bs!3,loc~j,p
Y~dp,tie1bs,veptpbs).Tie2(ang1bs,ang2bs!1,loc~j,pY~p,tie1bs!5,veptpbs).Tie2(a
ng1bs,ang2bs!2,loc~j,pY~p,tie1bs,veptpbs)"/>
      <species id="S126" compartment="cell" initialConcentration="0"
name="Akt(PHakt,S473~pS,T308~S)"/>
      <species id="S127" compartment="cell" initialConcentration="0"
name="Akt(PHakt!1,S473~pS,T308~pS).PI(PIsite~4P!1)"/>
      <species id="S128" compartment="cell" initialConcentration="0"
name="Ang1_4(tie2bs!1,tie2bs!2,tie2bs!3,tie2bs!4).Tie1(loc~j,pY~p,tie2bs!5).T
ie2(ang1bs!4,ang2bs,loc~j,pY~dp,tie1bs,veptpbs).Tie2(ang1bs!1,ang2bs,loc~j,pY
~p,tie1bs!5,veptpbs).Tie2(ang1bs!3,ang2bs,loc~j,pY~p,tie1bs,veptpbs!6).Tie2(a
ng1bs!2,ang2bs,loc~j,pY~p,tie1bs,veptpbs!7).VEPTP(tie2bs!6).VEPTP(tie2bs!7)"/
>
      <species id="S129" compartment="cell" initialConcentration="0"
name="Ang1_4(tie2bs!1,tie2bs!2,tie2bs!3,tie2bs!4).Tie2(ang1bs!4,ang2bs,loc~j,
pY~dp,tie1bs,veptpbs).Tie2(ang1bs!3,ang2bs,loc~j,pY~dp,tie1bs,veptpbs).Tie2(a
ng1bs!2,ang2bs,loc~j,pY~p,tie1bs,veptpbs!5).Tie2(ang1bs!1,ang2bs,loc~j,pY~p,t
ie1bs,veptpbs!6).VEPTP(tie2bs!5).VEPTP(tie2bs!6)"/>
      <species id="S130" compartment="cell" initialConcentration="0"
name="Ang2_4(tie2bs!1,tie2bs!2,tie2bs!3,tie2bs!4).Tie1(loc~j,pY~p,tie2bs!5).T
ie2(ang1bs,ang2bs!4,loc~j,pY~dp,tie1bs,veptpbs).Tie2(ang1bs,ang2bs!1,loc~j,pY
~p,tie1bs!5,veptpbs).Tie2(ang1bs,ang2bs!3,loc~j,pY~p,tie1bs,veptpbs!6).Tie2(a
ng1bs,ang2bs!2,loc~j,pY~p,tie1bs,veptpbs!7).VEPTP(tie2bs!6).VEPTP(tie2bs!7)"/
>
      <species id="S131" compartment="cell" initialConcentration="0"
name="Ang2_4(tie2bs!1,tie2bs!2,tie2bs!3,tie2bs!4).Tie2(ang1bs,ang2bs!4,loc~j,
```

```
pY~dp,tie1bs,veptpbs).Tie2(ang1bs,ang2bs!3,loc~j,pY~dp,tie1bs,veptpbs).Tie2(a
ng1bs,ang2bs!2,loc~j,pY~p,tie1bs,veptpbs!5).Tie2(ang1bs,ang2bs!1,loc~j,pY~p,t
ie1bs,veptpbs!6).VEPTP(tie2bs!5).VEPTP(tie2bs!6)"/>
      <species id="S132" compartment="cell" initialConcentration="0"
name="Ang1_4(tie2bs!1,tie2bs!2,tie2bs!3,tie2bs!4).Tie1(loc~j,pY~dp,tie2bs!5).
Tie2(ang1bs!4,ang2bs,loc~j,pY~dp,tie1bs,veptpbs).Tie2(ang1bs!3,ang2bs,loc~j,p
Y~dp,tie1bs,veptpbs).Tie2(ang1bs!1,ang2bs,loc~j,pY~p,tie1bs!5,veptpbs).Tie2(a
ng1bs!2,ang2bs,loc~j,pY~p,tie1bs,veptpbs!6).VEPTP(tie2bs!6)"/>
      <species id="S133" compartment="cell" initialConcentration="0"
name="Ang2_4(tie2bs!1,tie2bs!2,tie2bs!3,tie2bs!4).Tie1(loc~j,pY~dp,tie2bs!5).
Tie2(ang1bs,ang2bs!4,loc~j,pY~dp,tie1bs,veptpbs).Tie2(ang1bs,ang2bs!3,loc~j,p
Y~dp,tie1bs,veptpbs).Tie2(ang1bs,ang2bs!1,loc~j,pY~p,tie1bs!5,veptpbs).Tie2(a
ng1bs,ang2bs!2,loc~j,pY~p,tie1bs,veptpbs!6).VEPTP(tie2bs!6)"/>
      <species id="S134" compartment="cell" initialConcentration="0"
name="Ang1_4(tie2bs!1,tie2bs!2,tie2bs!3,tie2bs!4).Tie1(loc~j,pY~p,tie2bs!5).T
ie2(ang1bs!4,ang2bs,loc~j,pY~dp,tie1bs,veptpbs).Tie2(ang1bs!3,ang2bs,loc~j,pY
~dp,tie1bs,veptpbs).Tie2(ang1bs!1,ang2bs,loc~j,pY~p,tie1bs!5,veptpbs).Tie2(an
g1bs!2,ang2bs,loc~j,pY~p,tie1bs,veptpbs)"/>
      <species id="S135" compartment="cell" initialConcentration="0"
name="Ang1_4(tie2bs!1,tie2bs!2,tie2bs!3,tie2bs!4).Tie2(ang1bs!4,ang2bs,loc~j,
pY~dp,tie1bs,veptpbs).Tie2(ang1bs!3,ang2bs,loc~j,pY~dp,tie1bs,veptpbs).Tie2(a
ng1bs!2,ang2bs,loc~j,pY~dp,tie1bs,veptpbs).Tie2(ang1bs!1,ang2bs,loc~j,pY~p,ti
e1bs,veptpbs)"/>
      <species id="S136" compartment="cell" initialConcentration="0"
name="Ang2_4(tie2bs!1,tie2bs!2,tie2bs!3,tie2bs!4).Tie1(loc~j,pY~p,tie2bs!5).T
ie2(ang1bs,ang2bs!4,loc~j,pY~dp,tie1bs,veptpbs).Tie2(ang1bs,ang2bs!3,loc~j,pY
~dp,tie1bs,veptpbs).Tie2(ang1bs,ang2bs!1,loc~j,pY~p,tie1bs!5,veptpbs).Tie2(an
g1bs,ang2bs!2,loc~j,pY~p,tie1bs,veptpbs)"/>
      <species id="S137" compartment="cell" initialConcentration="0"
name="Ang2_4(tie2bs!1,tie2bs!2,tie2bs!3,tie2bs!4).Tie2(ang1bs,ang2bs!4,loc~j,
pY~dp,tie1bs,veptpbs).Tie2(ang1bs,ang2bs!3,loc~j,pY~dp,tie1bs,veptpbs).Tie2(a
ng1bs,ang2bs!2,loc~j,pY~dp,tie1bs,veptpbs).Tie2(ang1bs,ang2bs!1,loc~j,pY~p,ti
e1bs,veptpbs)"/>
      <species id="S138" compartment="cell" initialConcentration="0"
name="Akt(PHakt,S473~pS,T308~pS)"/>
      <species id="S139" compartment="cell" initialConcentration="0"
name="Akt(PHakt!1,S473~S,T308~pS).PI(PIsite~4P!1)"/>
      <species id="S140" compartment="cell" initialConcentration="0"
name="Ang1_4(tie2bs!1,tie2bs!2,tie2bs!3,tie2bs!4).Tie1(loc~j,pY~p,tie2bs!5).T
ie2(ang1bs!4,ang2bs,loc~j,pY~dp,tie1bs,veptpbs).Tie2(ang1bs!3,ang2bs,loc~j,pY
~dp,tie1bs,veptpbs).Tie2(ang1bs!1,ang2bs,loc~j,pY~p,tie1bs!5,veptpbs).Tie2(an
g1bs!2,ang2bs,loc~j,pY~p,tie1bs,veptpbs!6).VEPTP(tie2bs!6)"/>
      <species id="S141" compartment="cell" initialConcentration="0"
name="Ang1_4(tie2bs!1,tie2bs!2,tie2bs!3,tie2bs!4).Tie2(ang1bs!4,ang2bs,loc~j,
pY~dp,tie1bs,veptpbs).Tie2(ang1bs!3,ang2bs,loc~j,pY~dp,tie1bs,veptpbs).Tie2(a
ng1bs!2,ang2bs,loc~j,pY~dp,tie1bs,veptpbs).Tie2(ang1bs!1,ang2bs,loc~j,pY~p,ti
e1bs,veptpbs!5).VEPTP(tie2bs!5)"/>
      <species id="S142" compartment="cell" initialConcentration="0"
name="Ang2_4(tie2bs!1,tie2bs!2,tie2bs!3,tie2bs!4).Tie1(loc~j,pY~p,tie2bs!5).T
ie2(ang1bs,ang2bs!4,loc~j,pY~dp,tie1bs,veptpbs).Tie2(ang1bs,ang2bs!3,loc~j,pY
~dp,tie1bs,veptpbs).Tie2(ang1bs,ang2bs!1,loc~j,pY~p,tie1bs!5,veptpbs).Tie2(an
g1bs,ang2bs!2,loc~j,pY~p,tie1bs,veptpbs!6).VEPTP(tie2bs!6)"/>
      <species id="S143" compartment="cell" initialConcentration="0"
name="Ang2_4(tie2bs!1,tie2bs!2,tie2bs!3,tie2bs!4).Tie2(ang1bs,ang2bs!4,loc~j,
pY~dp,tie1bs,veptpbs).Tie2(ang1bs,ang2bs!3,loc~j,pY~dp,tie1bs,veptpbs).Tie2(a
ng1bs,ang2bs!2,loc~j,pY~dp,tie1bs,veptpbs).Tie2(ang1bs,ang2bs!1,loc~j,pY~p,ti
e1bs,veptpbs!5).VEPTP(tie2bs!5)"/>
```

```
      <species id="S144" compartment="cell" initialConcentration="0"
name="Ang1_4(tie2bs!1,tie2bs!2,tie2bs!3,tie2bs!4).Tie1(loc~j,pY~dp,tie2bs!5).
Tie2(ang1bs!4,ang2bs,loc~j,pY~dp,tie1bs,veptpbs).Tie2(ang1bs!3,ang2bs,loc~j,p
Y~dp,tie1bs,veptpbs).Tie2(ang1bs!2,ang2bs,loc~j,pY~dp,tie1bs,veptpbs).Tie2(an
g1bs!1,ang2bs,loc~j,pY~p,tie1bs!5,veptpbs)"/>
      <species id="S145" compartment="cell" initialConcentration="0"
name="Ang2_4(tie2bs!1,tie2bs!2,tie2bs!3,tie2bs!4).Tie1(loc~j,pY~dp,tie2bs!5).
Tie2(ang1bs,ang2bs!4,loc~j,pY~dp,tie1bs,veptpbs).Tie2(ang1bs,ang2bs!3,loc~j,p
Y~dp,tie1bs,veptpbs).Tie2(ang1bs,ang2bs!2,loc~j,pY~dp,tie1bs,veptpbs).Tie2(an
g1bs,ang2bs!1,loc~j,pY~p,tie1bs!5,veptpbs)"/>
      <species id="S146" compartment="cell" initialConcentration="0"
name="Akt(PHakt,S473~S,T308~pS)"/>
      <species id="S147" compartment="cell" initialConcentration="0"
name="Ang1_4(tie2bs!1,tie2bs!2,tie2bs!3,tie2bs!4).Tie1(loc~j,pY~p,tie2bs!5).T
ie2(ang1bs!4,ang2bs,loc~j,pY~dp,tie1bs,veptpbs).Tie2(ang1bs!3,ang2bs,loc~j,pY
~dp,tie1bs,veptpbs).Tie2(ang1bs!2,ang2bs,loc~j,pY~dp,tie1bs,veptpbs).Tie2(ang
1bs!1,ang2bs,loc~j,pY~p,tie1bs!5,veptpbs)"/>
      <species id="S148" compartment="cell" initialConcentration="0"
name="Ang1_4(tie2bs!1,tie2bs!2,tie2bs!3,tie2bs!4).Tie2(ang1bs!4,ang2bs,loc~j,
pY~dp,tie1bs,veptpbs).Tie2(ang1bs!3,ang2bs,loc~j,pY~dp,tie1bs,veptpbs).Tie2(a
ng1bs!2,ang2bs,loc~j,pY~dp,tie1bs,veptpbs).Tie2(ang1bs!1,ang2bs,loc~j,pY~dp,t
ie1bs,veptpbs)"/>
      <species id="S149" compartment="cell" initialConcentration="0"
name="Ang2_4(tie2bs!1,tie2bs!2,tie2bs!3,tie2bs!4).Tie1(loc~j,pY~p,tie2bs!5).T
ie2(ang1bs,ang2bs!4,loc~j,pY~dp,tie1bs,veptpbs).Tie2(ang1bs,ang2bs!3,loc~j,pY
~dp,tie1bs,veptpbs).Tie2(ang1bs,ang2bs!2,loc~j,pY~dp,tie1bs,veptpbs).Tie2(ang
1bs,ang2bs!1,loc~j,pY~p,tie1bs!5,veptpbs)"/>
      <species id="S150" compartment="cell" initialConcentration="0"
name="Ang2_4(tie2bs!1,tie2bs!2,tie2bs!3,tie2bs!4).Tie2(ang1bs,ang2bs!4,loc~j,
pY~dp,tie1bs,veptpbs).Tie2(ang1bs,ang2bs!3,loc~j,pY~dp,tie1bs,veptpbs).Tie2(a
ng1bs,ang2bs!2,loc~j,pY~dp,tie1bs,veptpbs).Tie2(ang1bs,ang2bs!1,loc~j,pY~dp,t
ie1bs,veptpbs)"/>
    </listOfSpecies>
    <listOfParameters>
      <!-- Independent variables -->
      <parameter id="Tie2_0" value="0.0012"/>
      <parameter id="Tie1_0" value="0.0006"/>
      <parameter id="Tie1Tie2_0" value="0.0012"/>
      <parameter id="Ang1_4_0" value="1.75"/>
      <parameter id="Ang2_2_0" value="1.3125"/>
      <parameter id="Ang2_3_0" value="1.575"/>
      <parameter id="Ang2_4_0" value="0.28"/>
      <parameter id="VEPTP_0" value="15"/>
      <parameter id="sTie2_0" value="1"/>
      <parameter id="sTie1_0" value="1"/>
      <parameter id="PTEN_0" value="0.1"/>
      <parameter id="PIP2_0" value="10"/>
      <parameter id="PI3K_0" value="0.1"/>
      <parameter id="Akt_0" value="0.1"/>
      <parameter id="PDK1_0" value="0.1"/>
      <parameter id="RhoA_0" value="0.1"/>
      <parameter id="mDia_0" value="0.1"/>
      <parameter id="Src_0" value="0.1"/>
      <parameter id="VECadherin_0" value="0.1"/>
      <parameter id="ABIN2_0" value="0.1"/>
      <parameter id="kD_ang1" value="3.7"/>
      <parameter id="kD_ang2" value="3.7"/>
```

```
<parameter id="koffang1tie2" value="0.5"/>
<parameter id="koffang2tie2" value="0.5"/>
<parameter id="kontie2diff" value="0.5"/>
<parameter id="kofftie2diff" value="0.1"/>
<parameter id="konstie2diff" value="0.5"/>
<parameter id="koffstie2diff" value="0.1"/>
<parameter id="kpang1tie2" value="1"/>
<parameter id="kdpang1tie2" value="0.01"/>
<parameter id="kpang2tie2" value="1"/>
<parameter id="kdpang2tie2" value="0.01"/>
<parameter id="ksjang1tie2" value="1"/>
<parameter id="kjsang1tie2" value="1"/>
<parameter id="ksjang2tie2" value="1"/>
<parameter id="kjsang2tie2" value="1"/>
<parameter id="ksjtie1" value="1"/>
<parameter id="kjstie1" value="1"/>
<parameter id="konang1tie1tie2_4_j" value="1"/>
<parameter id="koffang1tie1tie2_4_j" value="0.01"/>
<parameter id="konang2tie1tie2_4_j" value="1"/>
<parameter id="koffang2tie1tie2_4_j" value="0.01"/>
<parameter id="kptie1ang1_j" value="1"/>
<parameter id="kdpatie1ang1_j" value="0.01"/>
<parameter id="kptie1ang2_j" value="1"/>
<parameter id="kdpatie1ang2_j" value="0.01"/>
<parameter id="konveptp" value="0.01"/>
<parameter id="koffveptp" value="0.001"/>
<parameter id="kactveptp_ang1" value="30"/>
<parameter id="kactveptp_ang2" value="300"/>
<parameter id="kintang1dptie" value="0.0015"/>
<parameter id="kintang1ptie" value="0.01"/>
<parameter id="kintang2dptie" value="0.0015"/>
<parameter id="kintang2ptie" value="0.01"/>
<parameter id="kdegptie2" value="0.0001"/>
<parameter id="krectie2" value="0.00032"/>
<parameter id="ksyntie2" value="0.001"/>
<parameter id="ksyntie1" value="1e-06"/>
<parameter id="kontie1tie2" value="1.71e-12"/>
<parameter id="kofftie1tie2" value="0.1"/>
<parameter id="kdissang1tie1tie2" value="0.1"/>
<parameter id="kcleavetie2" value="0.0001"/>
<parameter id="kcleavetie1" value="0.0001"/>
<parameter id="kdegsTie2" value="0.0001"/>
<parameter id="kdegstie1" value="0.0001"/>
<parameter id="kactPI3KTie2" value="55.91689"/>
<parameter id="kinactPI3KTie2" value="0.6"/>
<parameter id="kPIP2gen" value="4.8e-05"/>
<parameter id="kmPIP2PI3K" value="309.9"/>
<parameter id="kcatPI3KPIP2" value="1764.48"/>
<parameter id="kmPIP3PTEN" value="6.27"/>
<parameter id="kcatPTENPIP3" value="4767.44"/>
<parameter id="konPDK1PIP3" value="5828.07"/>
<parameter id="koffPDK1PIP3" value="0.64"/>
<parameter id="konAKTPIP3" value="12.48"/>
<parameter id="koffAKTPIP3" value="0.032"/>
<parameter id="kpmTORAKT" value="2"/>
<parameter id="kpAKTPDK1" value="2"/>
<parameter id="kdp473AKTPPase" value="0.1"/>
```

```xml
<parameter id="kdp308AKTPPase" value="0.038"/>
<parameter id="kprhoa" value="0.1"/>
<parameter id="kdprhoa" value="0.1"/>
<parameter id="konrhoamdia" value="0.1"/>
<parameter id="koffrhoamdia" value="0.1"/>
<parameter id="konmdiasrc" value="0.1"/>
<parameter id="koffmdiasrc" value="0.1"/>
<parameter id="kpsrc" value="0.1"/>
<parameter id="kdpsrc" value="0.1"/>
<parameter id="kpvecad" value="0.1"/>
<parameter id="kdpvecad" value="0.1"/>
<parameter id="kintvecad" value="0.1"/>
<parameter id="krecvecad" value="0.1"/>
<parameter id="kdegvecad" value="0.1"/>
<parameter id="kactabin2" value="0.1"/>
<parameter id="kinactabin2" value="0.1"/>
<!-- Dependent variables -->
<parameter id="konang1tie2" constant="true"/>
<parameter id="konang2tie2" constant="true"/>
<parameter id="konang1tie2_1" constant="true"/>
<parameter id="koffang1tie2_1" constant="true"/>
<parameter id="konang1tie2_2" constant="true"/>
<parameter id="koffang1tie2_2" constant="true"/>
<parameter id="konang1tie2_3" constant="true"/>
<parameter id="koffang1tie2_3" constant="true"/>
<parameter id="konang1tie2_4" constant="true"/>
<parameter id="koffang1tie2_4" constant="true"/>
<parameter id="konang2_2tie2_1" constant="true"/>
<parameter id="koffang2_2tie2_1" constant="true"/>
<parameter id="konang2_2tie2_2" constant="true"/>
<parameter id="koffang2_2tie2_2" constant="true"/>
<parameter id="konang2_3tie2_1" constant="true"/>
<parameter id="koffang2_3tie2_1" constant="true"/>
<parameter id="konang2_3tie2_2" constant="true"/>
<parameter id="koffang2_3tie2_2" constant="true"/>
<parameter id="konang2_3tie2_3" constant="true"/>
<parameter id="koffang2_3tie2_3" constant="true"/>
<parameter id="konang2_4tie2_1" constant="true"/>
<parameter id="koffang2_4tie2_1" constant="true"/>
<parameter id="konang2_4tie2_2" constant="true"/>
<parameter id="koffang2_4tie2_2" constant="true"/>
<parameter id="konang2_4tie2_3" constant="true"/>
<parameter id="koffang2_4tie2_3" constant="true"/>
<parameter id="konang2_4tie2_4" constant="true"/>
<parameter id="koffang2_4tie2_4" constant="true"/>
<parameter id="kintang1tie2_1" constant="true"/>
<parameter id="kintang1tie2_2" constant="true"/>
<parameter id="kintang1tie2_3" constant="true"/>
<parameter id="kintang1tie2_4" constant="true"/>
<parameter id="kintang1p1tie2_4" constant="true"/>
<parameter id="kintang1p2tie2_4" constant="true"/>
<parameter id="kintang1p3tie2_4" constant="true"/>
<parameter id="kintang1p4tie2_4" constant="true"/>
<parameter id="kintang2_2tie2_1" constant="true"/>
<parameter id="kintang2_2tie2_2" constant="true"/>
<parameter id="kintang2_3tie2_1" constant="true"/>
<parameter id="kintang2_3tie2_2" constant="true"/>
```

```
<parameter id="kintang2_3tie2_3" constant="true"/>
<parameter id="kintang2_4tie2_1" constant="true"/>
<parameter id="kintang2_4tie2_2" constant="true"/>
<parameter id="kintang2_4tie2_3" constant="true"/>
<parameter id="kintang2_4tie2_4" constant="true"/>
<parameter id="kintang2_4p1tie2_4" constant="true"/>
<parameter id="kintang2_4p2tie2_4" constant="true"/>
<parameter id="kintang2_4p3tie2_4" constant="true"/>
<parameter id="kintang2_4p4tie2_4" constant="true"/>
<parameter id="konang1stie2_1" constant="true"/>
<parameter id="koffang1stie2_1" constant="true"/>
<parameter id="konang1stie2_2" constant="true"/>
<parameter id="koffang1stie2_2" constant="true"/>
<parameter id="konang1stie2_3" constant="true"/>
<parameter id="koffang1stie2_3" constant="true"/>
<parameter id="konang1stie2_4" constant="true"/>
<parameter id="koffang1stie2_4" constant="true"/>
<parameter id="konang2_2stie2_1" constant="true"/>
<parameter id="koffang2_2stie2_1" constant="true"/>
<parameter id="konang2_2stie2_2" constant="true"/>
<parameter id="koffang2_2stie2_2" constant="true"/>
<parameter id="konang2_3stie2_1" constant="true"/>
<parameter id="koffang2_3stie2_1" constant="true"/>
<parameter id="konang2_3stie2_2" constant="true"/>
<parameter id="koffang2_3stie2_2" constant="true"/>
<parameter id="konang2_3stie2_3" constant="true"/>
<parameter id="koffang2_3stie2_3" constant="true"/>
<parameter id="konang2_4stie2_1" constant="true"/>
<parameter id="koffang2_4stie2_1" constant="true"/>
<parameter id="konang2_4stie2_2" constant="true"/>
<parameter id="koffang2_4stie2_2" constant="true"/>
<parameter id="konang2_4stie2_3" constant="true"/>
<parameter id="koffang2_4stie2_3" constant="true"/>
<parameter id="konang2_4stie2_4" constant="true"/>
<parameter id="koffang2_4stie2_4" constant="true"/>
<parameter id="konang1_4tie1tie2" constant="true"/>
<parameter id="koffang1_4tie1tie2" constant="true"/>
<parameter id="koffang2_2tie1tie2" constant="true"/>
<parameter id="konang2_2tie1tie2" constant="true"/>
<parameter id="koffang2_3tie1tie2" constant="true"/>
<parameter id="konang2_3tie1tie2" constant="true"/>
<parameter id="koffang2_4tie1tie2" constant="true"/>
<parameter id="konang2_4tie1tie2" constant="true"/>
<!-- Observables -->
<parameter id="pTie2" constant="false"/>
<parameter id="intTie2" constant="false"/>
<parameter id="surfTie2" constant="false"/>
<parameter id="psurfTie2" constant="false"/>
<parameter id="pintTie2" constant="false"/>
<parameter id="totalTie2" constant="false"/>
<parameter id="sTie2" constant="false"/>
<parameter id="Tie1Tie2" constant="false"/>
<parameter id="surfTie1" constant="false"/>
<parameter id="sTie1" constant="false"/>
<parameter id="jTie2" constant="false"/>
<parameter id="tsurfTie1" constant="false"/>
<parameter id="jTie1" constant="false"/>
```

```
    <parameter id="freepip2" constant="false"/>
    <parameter id="freepip3" constant="false"/>
    <parameter id="ppAkt" constant="false"/>
    <parameter id="freeSrc" constant="false"/>
    <parameter id="pSrc" constant="false"/>
    <parameter id="pVECadherin" constant="false"/>
    <parameter id="aABIN2" constant="false"/>
    <parameter id="totalAkt" constant="false"/>
    <parameter id="RhoAGTP" constant="false"/>
    <parameter id="RhoAmDia" constant="false"/>
    <parameter id="mDiaSrc" constant="false"/>
    <!-- Global functions -->
    <parameter id="_rateLaw1" constant="false"/>
    <parameter id="_rateLaw2" constant="false"/>
  </listOfParameters>
  <listOfInitialAssignments>
    <!-- Dependent variables -->
    <initialAssignment symbol="konang1tie2">
      <math xmlns="http://www.w3.org/1998/Math/MathML">
        <apply>
          <divide/>
          <ci> koffang1tie2 </ci>
          <ci> kD_ang1 </ci>
        </apply>
      </math>
    </initialAssignment>
    <initialAssignment symbol="konang2tie2">
      <math xmlns="http://www.w3.org/1998/Math/MathML">
        <apply>
          <divide/>
          <ci> koffang2tie2 </ci>
          <ci> kD_ang2 </ci>
        </apply>
      </math>
    </initialAssignment>
    <initialAssignment symbol="konang1tie2_1">
      <math xmlns="http://www.w3.org/1998/Math/MathML">
        <ci> konang1tie2 </ci>
      </math>
    </initialAssignment>
    <initialAssignment symbol="koffang1tie2_1">
      <math xmlns="http://www.w3.org/1998/Math/MathML">
        <ci> koffang1tie2 </ci>
      </math>
    </initialAssignment>
    <initialAssignment symbol="konang1tie2_2">
      <math xmlns="http://www.w3.org/1998/Math/MathML">
        <ci> kontie2diff </ci>
      </math>
    </initialAssignment>
    <initialAssignment symbol="koffang1tie2_2">
      <math xmlns="http://www.w3.org/1998/Math/MathML">
        <ci> kofftie2diff </ci>
      </math>
    </initialAssignment>
    <initialAssignment symbol="konang1tie2_3">
      <math xmlns="http://www.w3.org/1998/Math/MathML">
```

```
    <ci> kontie2diff </ci>
  </math>
</initialAssignment>
<initialAssignment symbol="koffang1tie2_3">
  <math xmlns="http://www.w3.org/1998/Math/MathML">
    <ci> kofftie2diff </ci>
  </math>
</initialAssignment>
<initialAssignment symbol="konang1tie2_4">
  <math xmlns="http://www.w3.org/1998/Math/MathML">
    <ci> kontie2diff </ci>
  </math>
</initialAssignment>
<initialAssignment symbol="koffang1tie2_4">
  <math xmlns="http://www.w3.org/1998/Math/MathML">
    <ci> kofftie2diff </ci>
  </math>
</initialAssignment>
<initialAssignment symbol="konang2_2tie2_1">
  <math xmlns="http://www.w3.org/1998/Math/MathML">
    <ci> konang2tie2 </ci>
  </math>
</initialAssignment>
<initialAssignment symbol="koffang2_2tie2_1">
  <math xmlns="http://www.w3.org/1998/Math/MathML">
    <ci> koffang2tie2 </ci>
  </math>
</initialAssignment>
<initialAssignment symbol="konang2_2tie2_2">
  <math xmlns="http://www.w3.org/1998/Math/MathML">
    <ci> kontie2diff </ci>
  </math>
</initialAssignment>
<initialAssignment symbol="koffang2_2tie2_2">
  <math xmlns="http://www.w3.org/1998/Math/MathML">
    <ci> kofftie2diff </ci>
  </math>
</initialAssignment>
<initialAssignment symbol="konang2_3tie2_1">
  <math xmlns="http://www.w3.org/1998/Math/MathML">
    <ci> konang2tie2 </ci>
  </math>
</initialAssignment>
<initialAssignment symbol="koffang2_3tie2_1">
  <math xmlns="http://www.w3.org/1998/Math/MathML">
    <ci> koffang2tie2 </ci>
  </math>
</initialAssignment>
<initialAssignment symbol="konang2_3tie2_2">
  <math xmlns="http://www.w3.org/1998/Math/MathML">
    <ci> kontie2diff </ci>
  </math>
</initialAssignment>
<initialAssignment symbol="koffang2_3tie2_2">
  <math xmlns="http://www.w3.org/1998/Math/MathML">
    <ci> kofftie2diff </ci>
  </math>
```

```
&lt;/initialAssignment&gt;
&lt;initialAssignment symbol="konang2_3tie2_3"&gt;
  &lt;math xmlns="http://www.w3.org/1998/Math/MathML"&gt;
    &lt;ci&gt; kontie2diff &lt;/ci&gt;
  &lt;/math&gt;
&lt;/initialAssignment&gt;
&lt;initialAssignment symbol="koffang2_3tie2_3"&gt;
  &lt;math xmlns="http://www.w3.org/1998/Math/MathML"&gt;
    &lt;ci&gt; kofftie2diff &lt;/ci&gt;
  &lt;/math&gt;
&lt;/initialAssignment&gt;
&lt;initialAssignment symbol="konang2_4tie2_1"&gt;
  &lt;math xmlns="http://www.w3.org/1998/Math/MathML"&gt;
    &lt;ci&gt; konang2tie2 &lt;/ci&gt;
  &lt;/math&gt;
&lt;/initialAssignment&gt;
&lt;initialAssignment symbol="koffang2_4tie2_1"&gt;
  &lt;math xmlns="http://www.w3.org/1998/Math/MathML"&gt;
    &lt;ci&gt; koffang2tie2 &lt;/ci&gt;
  &lt;/math&gt;
&lt;/initialAssignment&gt;
&lt;initialAssignment symbol="konang2_4tie2_2"&gt;
  &lt;math xmlns="http://www.w3.org/1998/Math/MathML"&gt;
    &lt;ci&gt; kontie2diff &lt;/ci&gt;
  &lt;/math&gt;
&lt;/initialAssignment&gt;
&lt;initialAssignment symbol="koffang2_4tie2_2"&gt;
  &lt;math xmlns="http://www.w3.org/1998/Math/MathML"&gt;
    &lt;ci&gt; kofftie2diff &lt;/ci&gt;
  &lt;/math&gt;
&lt;/initialAssignment&gt;
&lt;initialAssignment symbol="konang2_4tie2_3"&gt;
  &lt;math xmlns="http://www.w3.org/1998/Math/MathML"&gt;
    &lt;ci&gt; kontie2diff &lt;/ci&gt;
  &lt;/math&gt;
&lt;/initialAssignment&gt;
&lt;initialAssignment symbol="koffang2_4tie2_3"&gt;
  &lt;math xmlns="http://www.w3.org/1998/Math/MathML"&gt;
    &lt;ci&gt; kofftie2diff &lt;/ci&gt;
  &lt;/math&gt;
&lt;/initialAssignment&gt;
&lt;initialAssignment symbol="konang2_4tie2_4"&gt;
  &lt;math xmlns="http://www.w3.org/1998/Math/MathML"&gt;
    &lt;ci&gt; kontie2diff &lt;/ci&gt;
  &lt;/math&gt;
&lt;/initialAssignment&gt;
&lt;initialAssignment symbol="koffang2_4tie2_4"&gt;
  &lt;math xmlns="http://www.w3.org/1998/Math/MathML"&gt;
    &lt;ci&gt; kofftie2diff &lt;/ci&gt;
  &lt;/math&gt;
&lt;/initialAssignment&gt;
&lt;initialAssignment symbol="kintang1tie2_1"&gt;
  &lt;math xmlns="http://www.w3.org/1998/Math/MathML"&gt;
    &lt;ci&gt; kintang1dptie &lt;/ci&gt;
  &lt;/math&gt;
&lt;/initialAssignment&gt;
&lt;initialAssignment symbol="kintang1tie2_2"&gt;
```

```
    <math xmlns="http://www.w3.org/1998/Math/MathML">
      <ci> kintang1dptie </ci>
    </math>
  </initialAssignment>
  <initialAssignment symbol="kintang1tie2_3">
    <math xmlns="http://www.w3.org/1998/Math/MathML">
      <ci> kintang1dptie </ci>
    </math>
  </initialAssignment>
  <initialAssignment symbol="kintang1tie2_4">
    <math xmlns="http://www.w3.org/1998/Math/MathML">
      <ci> kintang1dptie </ci>
    </math>
  </initialAssignment>
  <initialAssignment symbol="kintang1p1tie2_4">
    <math xmlns="http://www.w3.org/1998/Math/MathML">
      <ci> kintang1ptie </ci>
    </math>
  </initialAssignment>
  <initialAssignment symbol="kintang1p2tie2_4">
    <math xmlns="http://www.w3.org/1998/Math/MathML">
      <ci> kintang1ptie </ci>
    </math>
  </initialAssignment>
  <initialAssignment symbol="kintang1p3tie2_4">
    <math xmlns="http://www.w3.org/1998/Math/MathML">
      <ci> kintang1ptie </ci>
    </math>
  </initialAssignment>
  <initialAssignment symbol="kintang1p4tie2_4">
    <math xmlns="http://www.w3.org/1998/Math/MathML">
      <ci> kintang1ptie </ci>
    </math>
  </initialAssignment>
  <initialAssignment symbol="kintang2_2tie2_1">
    <math xmlns="http://www.w3.org/1998/Math/MathML">
      <ci> kintang2dptie </ci>
    </math>
  </initialAssignment>
  <initialAssignment symbol="kintang2_2tie2_2">
    <math xmlns="http://www.w3.org/1998/Math/MathML">
      <ci> kintang2dptie </ci>
    </math>
  </initialAssignment>
  <initialAssignment symbol="kintang2_3tie2_1">
    <math xmlns="http://www.w3.org/1998/Math/MathML">
      <ci> kintang2dptie </ci>
    </math>
  </initialAssignment>
  <initialAssignment symbol="kintang2_3tie2_2">
    <math xmlns="http://www.w3.org/1998/Math/MathML">
      <ci> kintang2dptie </ci>
    </math>
  </initialAssignment>
  <initialAssignment symbol="kintang2_3tie2_3">
    <math xmlns="http://www.w3.org/1998/Math/MathML">
      <ci> kintang2dptie </ci>
```

```xml
    </math>
  </initialAssignment>
  <initialAssignment symbol="kintang2_4tie2_1">
    <math xmlns="http://www.w3.org/1998/Math/MathML">
      <ci> kintang2dptie </ci>
    </math>
  </initialAssignment>
  <initialAssignment symbol="kintang2_4tie2_2">
    <math xmlns="http://www.w3.org/1998/Math/MathML">
      <ci> kintang2dptie </ci>
    </math>
  </initialAssignment>
  <initialAssignment symbol="kintang2_4tie2_3">
    <math xmlns="http://www.w3.org/1998/Math/MathML">
      <ci> kintang2dptie </ci>
    </math>
  </initialAssignment>
  <initialAssignment symbol="kintang2_4tie2_4">
    <math xmlns="http://www.w3.org/1998/Math/MathML">
      <ci> kintang2dptie </ci>
    </math>
  </initialAssignment>
  <initialAssignment symbol="kintang2_4p1tie2_4">
    <math xmlns="http://www.w3.org/1998/Math/MathML">
      <ci> kintang2ptie </ci>
    </math>
  </initialAssignment>
  <initialAssignment symbol="kintang2_4p2tie2_4">
    <math xmlns="http://www.w3.org/1998/Math/MathML">
      <ci> kintang2ptie </ci>
    </math>
  </initialAssignment>
  <initialAssignment symbol="kintang2_4p3tie2_4">
    <math xmlns="http://www.w3.org/1998/Math/MathML">
      <ci> kintang2ptie </ci>
    </math>
  </initialAssignment>
  <initialAssignment symbol="kintang2_4p4tie2_4">
    <math xmlns="http://www.w3.org/1998/Math/MathML">
      <ci> kintang2ptie </ci>
    </math>
  </initialAssignment>
  <initialAssignment symbol="konang1stie2_1">
    <math xmlns="http://www.w3.org/1998/Math/MathML">
      <ci> konang1tie2 </ci>
    </math>
  </initialAssignment>
  <initialAssignment symbol="koffang1stie2_1">
    <math xmlns="http://www.w3.org/1998/Math/MathML">
      <ci> koffang1tie2 </ci>
    </math>
  </initialAssignment>
  <initialAssignment symbol="konang1stie2_2">
    <math xmlns="http://www.w3.org/1998/Math/MathML">
      <ci> konstie2diff </ci>
    </math>
  </initialAssignment>
```

```
&lt;initialAssignment symbol="koffang1stie2_2"&gt;
  &lt;math xmlns="http://www.w3.org/1998/Math/MathML"&gt;
    &lt;ci&gt; koffstie2diff &lt;/ci&gt;
  &lt;/math&gt;
&lt;/initialAssignment&gt;
&lt;initialAssignment symbol="konang1stie2_3"&gt;
  &lt;math xmlns="http://www.w3.org/1998/Math/MathML"&gt;
    &lt;ci&gt; konstie2diff &lt;/ci&gt;
  &lt;/math&gt;
&lt;/initialAssignment&gt;
&lt;initialAssignment symbol="koffang1stie2_3"&gt;
  &lt;math xmlns="http://www.w3.org/1998/Math/MathML"&gt;
    &lt;ci&gt; koffstie2diff &lt;/ci&gt;
  &lt;/math&gt;
&lt;/initialAssignment&gt;
&lt;initialAssignment symbol="konang1stie2_4"&gt;
  &lt;math xmlns="http://www.w3.org/1998/Math/MathML"&gt;
    &lt;ci&gt; konstie2diff &lt;/ci&gt;
  &lt;/math&gt;
&lt;/initialAssignment&gt;
&lt;initialAssignment symbol="koffang1stie2_4"&gt;
  &lt;math xmlns="http://www.w3.org/1998/Math/MathML"&gt;
    &lt;ci&gt; koffstie2diff &lt;/ci&gt;
  &lt;/math&gt;
&lt;/initialAssignment&gt;
&lt;initialAssignment symbol="konang2_2stie2_1"&gt;
  &lt;math xmlns="http://www.w3.org/1998/Math/MathML"&gt;
    &lt;ci&gt; konang2tie2 &lt;/ci&gt;
  &lt;/math&gt;
&lt;/initialAssignment&gt;
&lt;initialAssignment symbol="koffang2_2stie2_1"&gt;
  &lt;math xmlns="http://www.w3.org/1998/Math/MathML"&gt;
    &lt;ci&gt; koffang2tie2 &lt;/ci&gt;
  &lt;/math&gt;
&lt;/initialAssignment&gt;
&lt;initialAssignment symbol="konang2_2stie2_2"&gt;
  &lt;math xmlns="http://www.w3.org/1998/Math/MathML"&gt;
    &lt;ci&gt; konstie2diff &lt;/ci&gt;
  &lt;/math&gt;
&lt;/initialAssignment&gt;
&lt;initialAssignment symbol="koffang2_2stie2_2"&gt;
  &lt;math xmlns="http://www.w3.org/1998/Math/MathML"&gt;
    &lt;ci&gt; koffstie2diff &lt;/ci&gt;
  &lt;/math&gt;
&lt;/initialAssignment&gt;
&lt;initialAssignment symbol="konang2_3stie2_1"&gt;
  &lt;math xmlns="http://www.w3.org/1998/Math/MathML"&gt;
    &lt;ci&gt; konang2tie2 &lt;/ci&gt;
  &lt;/math&gt;
&lt;/initialAssignment&gt;
&lt;initialAssignment symbol="koffang2_3stie2_1"&gt;
  &lt;math xmlns="http://www.w3.org/1998/Math/MathML"&gt;
    &lt;ci&gt; koffang2tie2 &lt;/ci&gt;
  &lt;/math&gt;
&lt;/initialAssignment&gt;
&lt;initialAssignment symbol="konang2_3stie2_2"&gt;
  &lt;math xmlns="http://www.w3.org/1998/Math/MathML"&gt;
```

```
    &lt;ci&gt; konstie2diff &lt;/ci&gt;
  &lt;/math&gt;
&lt;/initialAssignment&gt;
&lt;initialAssignment symbol="koffang2_3stie2_2"&gt;
  &lt;math xmlns="http://www.w3.org/1998/Math/MathML"&gt;
    &lt;ci&gt; koffstie2diff &lt;/ci&gt;
  &lt;/math&gt;
&lt;/initialAssignment&gt;
&lt;initialAssignment symbol="konang2_3stie2_3"&gt;
  &lt;math xmlns="http://www.w3.org/1998/Math/MathML"&gt;
    &lt;ci&gt; konstie2diff &lt;/ci&gt;
  &lt;/math&gt;
&lt;/initialAssignment&gt;
&lt;initialAssignment symbol="koffang2_3stie2_3"&gt;
  &lt;math xmlns="http://www.w3.org/1998/Math/MathML"&gt;
    &lt;ci&gt; koffstie2diff &lt;/ci&gt;
  &lt;/math&gt;
&lt;/initialAssignment&gt;
&lt;initialAssignment symbol="konang2_4stie2_1"&gt;
  &lt;math xmlns="http://www.w3.org/1998/Math/MathML"&gt;
    &lt;ci&gt; konang2tie2 &lt;/ci&gt;
  &lt;/math&gt;
&lt;/initialAssignment&gt;
&lt;initialAssignment symbol="koffang2_4stie2_1"&gt;
  &lt;math xmlns="http://www.w3.org/1998/Math/MathML"&gt;
    &lt;ci&gt; koffang2tie2 &lt;/ci&gt;
  &lt;/math&gt;
&lt;/initialAssignment&gt;
&lt;initialAssignment symbol="konang2_4stie2_2"&gt;
  &lt;math xmlns="http://www.w3.org/1998/Math/MathML"&gt;
    &lt;ci&gt; konstie2diff &lt;/ci&gt;
  &lt;/math&gt;
&lt;/initialAssignment&gt;
&lt;initialAssignment symbol="koffang2_4stie2_2"&gt;
  &lt;math xmlns="http://www.w3.org/1998/Math/MathML"&gt;
    &lt;ci&gt; koffstie2diff &lt;/ci&gt;
  &lt;/math&gt;
&lt;/initialAssignment&gt;
&lt;initialAssignment symbol="konang2_4stie2_3"&gt;
  &lt;math xmlns="http://www.w3.org/1998/Math/MathML"&gt;
    &lt;ci&gt; konstie2diff &lt;/ci&gt;
  &lt;/math&gt;
&lt;/initialAssignment&gt;
&lt;initialAssignment symbol="koffang2_4stie2_3"&gt;
  &lt;math xmlns="http://www.w3.org/1998/Math/MathML"&gt;
    &lt;ci&gt; koffstie2diff &lt;/ci&gt;
  &lt;/math&gt;
&lt;/initialAssignment&gt;
&lt;initialAssignment symbol="konang2_4stie2_4"&gt;
  &lt;math xmlns="http://www.w3.org/1998/Math/MathML"&gt;
    &lt;ci&gt; konstie2diff &lt;/ci&gt;
  &lt;/math&gt;
&lt;/initialAssignment&gt;
&lt;initialAssignment symbol="koffang2_4stie2_4"&gt;
  &lt;math xmlns="http://www.w3.org/1998/Math/MathML"&gt;
    &lt;ci&gt; koffstie2diff &lt;/ci&gt;
  &lt;/math&gt;
```

```
</initialAssignment>
<initialAssignment symbol="konang1_4tie1tie2">
  <math xmlns="http://www.w3.org/1998/Math/MathML">
    <apply>
      <divide/>
      <ci> koffang1tie2 </ci>
      <ci> kD_ang1 </ci>
    </apply>
  </math>
</initialAssignment>
<initialAssignment symbol="koffang1_4tie1tie2">
  <math xmlns="http://www.w3.org/1998/Math/MathML">
    <ci> koffang1tie2 </ci>
  </math>
</initialAssignment>
<initialAssignment symbol="koffang2_2tie1tie2">
  <math xmlns="http://www.w3.org/1998/Math/MathML">
    <ci> koffang2tie2 </ci>
  </math>
</initialAssignment>
<initialAssignment symbol="konang2_2tie1tie2">
  <math xmlns="http://www.w3.org/1998/Math/MathML">
    <apply>
      <divide/>
      <ci> koffang2tie2 </ci>
      <ci> kD_ang2 </ci>
    </apply>
  </math>
</initialAssignment>
<initialAssignment symbol="koffang2_3tie1tie2">
  <math xmlns="http://www.w3.org/1998/Math/MathML">
    <ci> koffang2tie2 </ci>
  </math>
</initialAssignment>
<initialAssignment symbol="konang2_3tie1tie2">
  <math xmlns="http://www.w3.org/1998/Math/MathML">
    <apply>
      <divide/>
      <ci> koffang2tie2 </ci>
      <ci> kD_ang2 </ci>
    </apply>
  </math>
</initialAssignment>
<initialAssignment symbol="koffang2_4tie1tie2">
  <math xmlns="http://www.w3.org/1998/Math/MathML">
    <ci> koffang2tie2 </ci>
  </math>
</initialAssignment>
<initialAssignment symbol="konang2_4tie1tie2">
  <math xmlns="http://www.w3.org/1998/Math/MathML">
    <apply>
      <divide/>
      <ci> koffang2tie2 </ci>
      <ci> kD_ang2 </ci>
    </apply>
  </math>
</initialAssignment>
```

```
</listOfInitialAssignments>
<listOfRules>
  <!-- Observables -->
  <assignmentRule variable="pTie2">
      <math xmlns="http://www.w3.org/1998/Math/MathML">
        <apply>
          <plus/>
          <ci> S58 </ci>
          <ci> S59 </ci>
          <apply>
            <times/>
            <cn> 2 </cn>
            <ci> S60 </ci>
          </apply>
          <apply>
            <times/>
            <cn> 2 </cn>
            <ci> S61 </ci>
          </apply>
          <ci> S62 </ci>
          <apply>
            <times/>
            <cn> 3 </cn>
            <ci> S63 </ci>
          </apply>
          <apply>
            <times/>
            <cn> 3 </cn>
            <ci> S64 </ci>
          </apply>
          <apply>
            <times/>
            <cn> 4 </cn>
            <ci> S65 </ci>
          </apply>
          <apply>
            <times/>
            <cn> 4 </cn>
            <ci> S66 </ci>
          </apply>
          <apply>
            <times/>
            <cn> 4 </cn>
            <ci> S67 </ci>
          </apply>
          <apply>
            <times/>
            <cn> 4 </cn>
            <ci> S68 </ci>
          </apply>
          <apply>
            <times/>
            <cn> 4 </cn>
            <ci> S69 </ci>
          </apply>
          <apply>
            <times/>
```

```
    &lt;cn&gt; 4 &lt;/cn&gt;
    &lt;ci&gt; S70 &lt;/ci&gt;
  &lt;/apply&gt;
  &lt;apply&gt;
    &lt;times/&gt;
    &lt;cn&gt; 4 &lt;/cn&gt;
    &lt;ci&gt; S71 &lt;/ci&gt;
  &lt;/apply&gt;
  &lt;apply&gt;
    &lt;times/&gt;
    &lt;cn&gt; 4 &lt;/cn&gt;
    &lt;ci&gt; S72 &lt;/ci&gt;
  &lt;/apply&gt;
  &lt;apply&gt;
    &lt;times/&gt;
    &lt;cn&gt; 4 &lt;/cn&gt;
    &lt;ci&gt; S76 &lt;/ci&gt;
  &lt;/apply&gt;
  &lt;apply&gt;
    &lt;times/&gt;
    &lt;cn&gt; 4 &lt;/cn&gt;
    &lt;ci&gt; S77 &lt;/ci&gt;
  &lt;/apply&gt;
  &lt;apply&gt;
    &lt;times/&gt;
    &lt;cn&gt; 4 &lt;/cn&gt;
    &lt;ci&gt; S78 &lt;/ci&gt;
  &lt;/apply&gt;
  &lt;apply&gt;
    &lt;times/&gt;
    &lt;cn&gt; 4 &lt;/cn&gt;
    &lt;ci&gt; S79 &lt;/ci&gt;
  &lt;/apply&gt;
  &lt;apply&gt;
    &lt;times/&gt;
    &lt;cn&gt; 4 &lt;/cn&gt;
    &lt;ci&gt; S80 &lt;/ci&gt;
  &lt;/apply&gt;
  &lt;apply&gt;
    &lt;times/&gt;
    &lt;cn&gt; 4 &lt;/cn&gt;
    &lt;ci&gt; S81 &lt;/ci&gt;
  &lt;/apply&gt;
  &lt;apply&gt;
    &lt;times/&gt;
    &lt;cn&gt; 3 &lt;/cn&gt;
    &lt;ci&gt; S82 &lt;/ci&gt;
  &lt;/apply&gt;
  &lt;apply&gt;
    &lt;times/&gt;
    &lt;cn&gt; 3 &lt;/cn&gt;
    &lt;ci&gt; S83 &lt;/ci&gt;
  &lt;/apply&gt;
  &lt;apply&gt;
    &lt;times/&gt;
    &lt;cn&gt; 4 &lt;/cn&gt;
    &lt;ci&gt; S86 &lt;/ci&gt;
```

```
</apply>
<apply>
  <times/>
  <cn> 4 </cn>
  <ci> S87 </ci>
</apply>
<apply>
  <times/>
  <cn> 4 </cn>
  <ci> S88 </ci>
</apply>
<apply>
  <times/>
  <cn> 4 </cn>
  <ci> S89 </ci>
</apply>
<apply>
  <times/>
  <cn> 4 </cn>
  <ci> S90 </ci>
</apply>
<apply>
  <times/>
  <cn> 4 </cn>
  <ci> S91 </ci>
</apply>
<apply>
  <times/>
  <cn> 3 </cn>
  <ci> S92 </ci>
</apply>
<apply>
  <times/>
  <cn> 3 </cn>
  <ci> S93 </ci>
</apply>
<apply>
  <times/>
  <cn> 3 </cn>
  <ci> S94 </ci>
</apply>
<apply>
  <times/>
  <cn> 3 </cn>
  <ci> S95 </ci>
</apply>
<apply>
  <times/>
  <cn> 4 </cn>
  <ci> S99 </ci>
</apply>
<apply>
  <times/>
  <cn> 4 </cn>
  <ci> S100 </ci>
</apply>
<apply>
```

```
  <times/>
  <cn> 4 </cn>
  <ci> S101 </ci>
</apply>
<apply>
  <times/>
  <cn> 4 </cn>
  <ci> S102 </ci>
</apply>
<apply>
  <times/>
  <cn> 4 </cn>
  <ci> S103 </ci>
</apply>
<apply>
  <times/>
  <cn> 4 </cn>
  <ci> S104 </ci>
</apply>
<apply>
  <times/>
  <cn> 3 </cn>
  <ci> S105 </ci>
</apply>
<apply>
  <times/>
  <cn> 3 </cn>
  <ci> S106 </ci>
</apply>
<apply>
  <times/>
  <cn> 3 </cn>
  <ci> S107 </ci>
</apply>
<apply>
  <times/>
  <cn> 3 </cn>
  <ci> S108 </ci>
</apply>
<apply>
  <times/>
  <cn> 3 </cn>
  <ci> S109 </ci>
</apply>
<apply>
  <times/>
  <cn> 2 </cn>
  <ci> S110 </ci>
</apply>
<apply>
  <times/>
  <cn> 3 </cn>
  <ci> S111 </ci>
</apply>
<apply>
  <times/>
  <cn> 2 </cn>
```

```
  <ci> S112 </ci>
</apply>
<apply>
  <times/>
  <cn> 4 </cn>
  <ci> S114 </ci>
</apply>
<apply>
  <times/>
  <cn> 4 </cn>
  <ci> S115 </ci>
</apply>
<apply>
  <times/>
  <cn> 3 </cn>
  <ci> S116 </ci>
</apply>
<apply>
  <times/>
  <cn> 3 </cn>
  <ci> S117 </ci>
</apply>
<apply>
  <times/>
  <cn> 3 </cn>
  <ci> S118 </ci>
</apply>
<apply>
  <times/>
  <cn> 3 </cn>
  <ci> S119 </ci>
</apply>
<apply>
  <times/>
  <cn> 3 </cn>
  <ci> S120 </ci>
</apply>
<apply>
  <times/>
  <cn> 2 </cn>
  <ci> S121 </ci>
</apply>
<apply>
  <times/>
  <cn> 3 </cn>
  <ci> S122 </ci>
</apply>
<apply>
  <times/>
  <cn> 2 </cn>
  <ci> S123 </ci>
</apply>
<apply>
  <times/>
  <cn> 2 </cn>
  <ci> S124 </ci>
</apply>
```

```xml
<apply>
  <times/>
  <cn> 2 </cn>
  <ci> S125 </ci>
</apply>
<apply>
  <times/>
  <cn> 3 </cn>
  <ci> S128 </ci>
</apply>
<apply>
  <times/>
  <cn> 2 </cn>
  <ci> S129 </ci>
</apply>
<apply>
  <times/>
  <cn> 3 </cn>
  <ci> S130 </ci>
</apply>
<apply>
  <times/>
  <cn> 2 </cn>
  <ci> S131 </ci>
</apply>
<apply>
  <times/>
  <cn> 2 </cn>
  <ci> S132 </ci>
</apply>
<apply>
  <times/>
  <cn> 2 </cn>
  <ci> S133 </ci>
</apply>
<apply>
  <times/>
  <cn> 2 </cn>
  <ci> S134 </ci>
</apply>
<ci> S135 </ci>
<apply>
  <times/>
  <cn> 2 </cn>
  <ci> S136 </ci>
</apply>
<ci> S137 </ci>
<apply>
  <times/>
  <cn> 2 </cn>
  <ci> S140 </ci>
</apply>
<ci> S141 </ci>
<apply>
  <times/>
  <cn> 2 </cn>
  <ci> S142 </ci>
```

```
        &lt;/apply&gt;
        &lt;ci&gt; S143 &lt;/ci&gt;
        &lt;ci&gt; S144 &lt;/ci&gt;
        &lt;ci&gt; S145 &lt;/ci&gt;
        &lt;ci&gt; S147 &lt;/ci&gt;
        &lt;ci&gt; S149 &lt;/ci&gt;
      &lt;/apply&gt;
    &lt;/math&gt;
&lt;/assignmentRule&gt;
&lt;assignmentRule variable="intTie2"&gt;
    &lt;math xmlns="http://www.w3.org/1998/Math/MathML"&gt;
      &lt;apply&gt;
        &lt;plus/&gt;
        &lt;ci&gt; S41 &lt;/ci&gt;
        &lt;ci&gt; S62 &lt;/ci&gt;
      &lt;/apply&gt;
    &lt;/math&gt;
&lt;/assignmentRule&gt;
&lt;assignmentRule variable="surfTie2"&gt;
    &lt;math xmlns="http://www.w3.org/1998/Math/MathML"&gt;
      &lt;apply&gt;
        &lt;plus/&gt;
        &lt;ci&gt; S1 &lt;/ci&gt;
        &lt;ci&gt; S10 &lt;/ci&gt;
        &lt;ci&gt; S23 &lt;/ci&gt;
        &lt;ci&gt; S24 &lt;/ci&gt;
        &lt;ci&gt; S25 &lt;/ci&gt;
        &lt;ci&gt; S26 &lt;/ci&gt;
        &lt;ci&gt; S32 &lt;/ci&gt;
        &lt;ci&gt; S33 &lt;/ci&gt;
        &lt;ci&gt; S34 &lt;/ci&gt;
        &lt;ci&gt; S35 &lt;/ci&gt;
        &lt;apply&gt;
          &lt;times/&gt;
          &lt;cn&gt; 2 &lt;/cn&gt;
          &lt;ci&gt; S37 &lt;/ci&gt;
        &lt;/apply&gt;
        &lt;apply&gt;
          &lt;times/&gt;
          &lt;cn&gt; 2 &lt;/cn&gt;
          &lt;ci&gt; S38 &lt;/ci&gt;
        &lt;/apply&gt;
        &lt;apply&gt;
          &lt;times/&gt;
          &lt;cn&gt; 2 &lt;/cn&gt;
          &lt;ci&gt; S39 &lt;/ci&gt;
        &lt;/apply&gt;
        &lt;apply&gt;
          &lt;times/&gt;
          &lt;cn&gt; 2 &lt;/cn&gt;
          &lt;ci&gt; S40 &lt;/ci&gt;
        &lt;/apply&gt;
        &lt;apply&gt;
          &lt;times/&gt;
          &lt;cn&gt; 3 &lt;/cn&gt;
          &lt;ci&gt; S47 &lt;/ci&gt;
        &lt;/apply&gt;
```

```
<apply>
  <times/>
  <cn> 3 </cn>
  <ci> S48 </ci>
</apply>
<apply>
  <times/>
  <cn> 3 </cn>
  <ci> S49 </ci>
</apply>
<apply>
  <times/>
  <cn> 4 </cn>
  <ci> S54 </ci>
</apply>
<apply>
  <times/>
  <cn> 4 </cn>
  <ci> S55 </ci>
</apply>
<apply>
  <times/>
  <cn> 4 </cn>
  <ci> S58 </ci>
</apply>
<apply>
  <times/>
  <cn> 4 </cn>
  <ci> S59 </ci>
</apply>
<apply>
  <times/>
  <cn> 4 </cn>
  <ci> S60 </ci>
</apply>
<apply>
  <times/>
  <cn> 4 </cn>
  <ci> S61 </ci>
</apply>
<apply>
  <times/>
  <cn> 4 </cn>
  <ci> S63 </ci>
</apply>
<apply>
  <times/>
  <cn> 4 </cn>
  <ci> S64 </ci>
</apply>
<apply>
  <times/>
  <cn> 4 </cn>
  <ci> S65 </ci>
</apply>
<apply>
  <times/>
```

```
          <cn> 4 </cn>
          <ci> S66 </ci>
        </apply>
      </apply>
    </math>
</assignmentRule>
<assignmentRule variable="psurfTie2">
    <math xmlns="http://www.w3.org/1998/Math/MathML">
      <apply>
        <plus/>
        <ci> S58 </ci>
        <ci> S59 </ci>
        <apply>
          <times/>
          <cn> 2 </cn>
          <ci> S60 </ci>
        </apply>
        <apply>
          <times/>
          <cn> 2 </cn>
          <ci> S61 </ci>
        </apply>
        <apply>
          <times/>
          <cn> 3 </cn>
          <ci> S63 </ci>
        </apply>
        <apply>
          <times/>
          <cn> 3 </cn>
          <ci> S64 </ci>
        </apply>
        <apply>
          <times/>
          <cn> 4 </cn>
          <ci> S65 </ci>
        </apply>
        <apply>
          <times/>
          <cn> 4 </cn>
          <ci> S66 </ci>
        </apply>
      </apply>
    </math>
</assignmentRule>
<assignmentRule variable="pintTie2">
    <math xmlns="http://www.w3.org/1998/Math/MathML">
      <apply>
        <plus/>
        <cn> 0 </cn>
        <ci> S62 </ci>
      </apply>
    </math>
</assignmentRule>
<assignmentRule variable="totalTie2">
    <math xmlns="http://www.w3.org/1998/Math/MathML">
      <apply>
```

```
<plus/>
<ci> S1 </ci>
<ci> S7 </ci>
<ci> S10 </ci>
<ci> S23 </ci>
<ci> S24 </ci>
<ci> S25 </ci>
<ci> S26 </ci>
<ci> S28 </ci>
<ci> S29 </ci>
<ci> S30 </ci>
<ci> S31 </ci>
<ci> S32 </ci>
<ci> S33 </ci>
<ci> S34 </ci>
<ci> S35 </ci>
<apply>
  <times/>
  <cn> 2 </cn>
  <ci> S37 </ci>
</apply>
<apply>
  <times/>
  <cn> 2 </cn>
  <ci> S38 </ci>
</apply>
<apply>
  <times/>
  <cn> 2 </cn>
  <ci> S39 </ci>
</apply>
<apply>
  <times/>
  <cn> 2 </cn>
  <ci> S40 </ci>
</apply>
<ci> S41 </ci>
<apply>
  <times/>
  <cn> 2 </cn>
  <ci> S42 </ci>
</apply>
<apply>
  <times/>
  <cn> 2 </cn>
  <ci> S43 </ci>
</apply>
<apply>
  <times/>
  <cn> 2 </cn>
  <ci> S44 </ci>
</apply>
<apply>
  <times/>
  <cn> 2 </cn>
  <ci> S45 </ci>
</apply>
```

```
&lt;apply&gt;
  &lt;times/&gt;
  &lt;cn&gt; 3 &lt;/cn&gt;
  &lt;ci&gt; S47 &lt;/ci&gt;
&lt;/apply&gt;
&lt;apply&gt;
  &lt;times/&gt;
  &lt;cn&gt; 3 &lt;/cn&gt;
  &lt;ci&gt; S48 &lt;/ci&gt;
&lt;/apply&gt;
&lt;apply&gt;
  &lt;times/&gt;
  &lt;cn&gt; 3 &lt;/cn&gt;
  &lt;ci&gt; S49 &lt;/ci&gt;
&lt;/apply&gt;
&lt;apply&gt;
  &lt;times/&gt;
  &lt;cn&gt; 3 &lt;/cn&gt;
  &lt;ci&gt; S50 &lt;/ci&gt;
&lt;/apply&gt;
&lt;apply&gt;
  &lt;times/&gt;
  &lt;cn&gt; 3 &lt;/cn&gt;
  &lt;ci&gt; S51 &lt;/ci&gt;
&lt;/apply&gt;
&lt;apply&gt;
  &lt;times/&gt;
  &lt;cn&gt; 3 &lt;/cn&gt;
  &lt;ci&gt; S52 &lt;/ci&gt;
&lt;/apply&gt;
&lt;apply&gt;
  &lt;times/&gt;
  &lt;cn&gt; 4 &lt;/cn&gt;
  &lt;ci&gt; S54 &lt;/ci&gt;
&lt;/apply&gt;
&lt;apply&gt;
  &lt;times/&gt;
  &lt;cn&gt; 4 &lt;/cn&gt;
  &lt;ci&gt; S55 &lt;/ci&gt;
&lt;/apply&gt;
&lt;apply&gt;
  &lt;times/&gt;
  &lt;cn&gt; 4 &lt;/cn&gt;
  &lt;ci&gt; S56 &lt;/ci&gt;
&lt;/apply&gt;
&lt;apply&gt;
  &lt;times/&gt;
  &lt;cn&gt; 4 &lt;/cn&gt;
  &lt;ci&gt; S57 &lt;/ci&gt;
&lt;/apply&gt;
&lt;apply&gt;
  &lt;times/&gt;
  &lt;cn&gt; 4 &lt;/cn&gt;
  &lt;ci&gt; S58 &lt;/ci&gt;
&lt;/apply&gt;
&lt;apply&gt;
  &lt;times/&gt;
```

```
    <cn> 4 </cn>
    <ci> S59 </ci>
  </apply>
  <apply>
    <times/>
    <cn> 4 </cn>
    <ci> S60 </ci>
  </apply>
  <apply>
    <times/>
    <cn> 4 </cn>
    <ci> S61 </ci>
  </apply>
  <ci> S62 </ci>
  <apply>
    <times/>
    <cn> 4 </cn>
    <ci> S63 </ci>
  </apply>
  <apply>
    <times/>
    <cn> 4 </cn>
    <ci> S64 </ci>
  </apply>
  <apply>
    <times/>
    <cn> 4 </cn>
    <ci> S65 </ci>
  </apply>
  <apply>
    <times/>
    <cn> 4 </cn>
    <ci> S66 </ci>
  </apply>
  <apply>
    <times/>
    <cn> 4 </cn>
    <ci> S67 </ci>
  </apply>
  <apply>
    <times/>
    <cn> 4 </cn>
    <ci> S68 </ci>
  </apply>
  <apply>
    <times/>
    <cn> 4 </cn>
    <ci> S69 </ci>
  </apply>
  <apply>
    <times/>
    <cn> 4 </cn>
    <ci> S70 </ci>
  </apply>
  <apply>
    <times/>
    <cn> 4 </cn>
```

```
    <ci> S71 </ci>
  </apply>
  <apply>
    <times/>
    <cn> 4 </cn>
    <ci> S72 </ci>
  </apply>
  <apply>
    <times/>
    <cn> 4 </cn>
    <ci> S76 </ci>
  </apply>
  <apply>
    <times/>
    <cn> 4 </cn>
    <ci> S77 </ci>
  </apply>
  <apply>
    <times/>
    <cn> 4 </cn>
    <ci> S78 </ci>
  </apply>
  <apply>
    <times/>
    <cn> 4 </cn>
    <ci> S79 </ci>
  </apply>
  <apply>
    <times/>
    <cn> 4 </cn>
    <ci> S80 </ci>
  </apply>
  <apply>
    <times/>
    <cn> 4 </cn>
    <ci> S81 </ci>
  </apply>
  <apply>
    <times/>
    <cn> 4 </cn>
    <ci> S82 </ci>
  </apply>
  <apply>
    <times/>
    <cn> 4 </cn>
    <ci> S83 </ci>
  </apply>
  <apply>
    <times/>
    <cn> 4 </cn>
    <ci> S86 </ci>
  </apply>
  <apply>
    <times/>
    <cn> 4 </cn>
    <ci> S87 </ci>
  </apply>
```

```
<apply>
  <times/>
  <cn> 4 </cn>
  <ci> S88 </ci>
</apply>
<apply>
  <times/>
  <cn> 4 </cn>
  <ci> S89 </ci>
</apply>
<apply>
  <times/>
  <cn> 4 </cn>
  <ci> S90 </ci>
</apply>
<apply>
  <times/>
  <cn> 4 </cn>
  <ci> S91 </ci>
</apply>
<apply>
  <times/>
  <cn> 4 </cn>
  <ci> S92 </ci>
</apply>
<apply>
  <times/>
  <cn> 4 </cn>
  <ci> S93 </ci>
</apply>
<apply>
  <times/>
  <cn> 4 </cn>
  <ci> S94 </ci>
</apply>
<apply>
  <times/>
  <cn> 4 </cn>
  <ci> S95 </ci>
</apply>
<apply>
  <times/>
  <cn> 4 </cn>
  <ci> S99 </ci>
</apply>
<apply>
  <times/>
  <cn> 4 </cn>
  <ci> S100 </ci>
</apply>
<apply>
  <times/>
  <cn> 4 </cn>
  <ci> S101 </ci>
</apply>
<apply>
  <times/>
```

```
  <cn> 4 </cn>
  <ci> S102 </ci>
</apply>
<apply>
  <times/>
  <cn> 4 </cn>
  <ci> S103 </ci>
</apply>
<apply>
  <times/>
  <cn> 4 </cn>
  <ci> S104 </ci>
</apply>
<apply>
  <times/>
  <cn> 4 </cn>
  <ci> S105 </ci>
</apply>
<apply>
  <times/>
  <cn> 4 </cn>
  <ci> S106 </ci>
</apply>
<apply>
  <times/>
  <cn> 4 </cn>
  <ci> S107 </ci>
</apply>
<apply>
  <times/>
  <cn> 4 </cn>
  <ci> S108 </ci>
</apply>
<apply>
  <times/>
  <cn> 4 </cn>
  <ci> S109 </ci>
</apply>
<apply>
  <times/>
  <cn> 4 </cn>
  <ci> S110 </ci>
</apply>
<apply>
  <times/>
  <cn> 4 </cn>
  <ci> S111 </ci>
</apply>
<apply>
  <times/>
  <cn> 4 </cn>
  <ci> S112 </ci>
</apply>
<apply>
  <times/>
  <cn> 4 </cn>
  <ci> S114 </ci>
```

```
</apply>
<apply>
  <times/>
  <cn> 4 </cn>
  <ci> S115 </ci>
</apply>
<apply>
  <times/>
  <cn> 4 </cn>
  <ci> S116 </ci>
</apply>
<apply>
  <times/>
  <cn> 4 </cn>
  <ci> S117 </ci>
</apply>
<apply>
  <times/>
  <cn> 4 </cn>
  <ci> S118 </ci>
</apply>
<apply>
  <times/>
  <cn> 4 </cn>
  <ci> S119 </ci>
</apply>
<apply>
  <times/>
  <cn> 4 </cn>
  <ci> S120 </ci>
</apply>
<apply>
  <times/>
  <cn> 4 </cn>
  <ci> S121 </ci>
</apply>
<apply>
  <times/>
  <cn> 4 </cn>
  <ci> S122 </ci>
</apply>
<apply>
  <times/>
  <cn> 4 </cn>
  <ci> S123 </ci>
</apply>
<apply>
  <times/>
  <cn> 4 </cn>
  <ci> S124 </ci>
</apply>
<apply>
  <times/>
  <cn> 4 </cn>
  <ci> S125 </ci>
</apply>
<apply>
```

```
    <times/>
    <cn> 4 </cn>
    <ci> S128 </ci>
  </apply>
  <apply>
    <times/>
    <cn> 4 </cn>
    <ci> S129 </ci>
  </apply>
  <apply>
    <times/>
    <cn> 4 </cn>
    <ci> S130 </ci>
  </apply>
  <apply>
    <times/>
    <cn> 4 </cn>
    <ci> S131 </ci>
  </apply>
  <apply>
    <times/>
    <cn> 4 </cn>
    <ci> S132 </ci>
  </apply>
  <apply>
    <times/>
    <cn> 4 </cn>
    <ci> S133 </ci>
  </apply>
  <apply>
    <times/>
    <cn> 4 </cn>
    <ci> S134 </ci>
  </apply>
  <apply>
    <times/>
    <cn> 4 </cn>
    <ci> S135 </ci>
  </apply>
  <apply>
    <times/>
    <cn> 4 </cn>
    <ci> S136 </ci>
  </apply>
  <apply>
    <times/>
    <cn> 4 </cn>
    <ci> S137 </ci>
  </apply>
  <apply>
    <times/>
    <cn> 4 </cn>
    <ci> S140 </ci>
  </apply>
  <apply>
    <times/>
    <cn> 4 </cn>
```

```
          <ci> S141 </ci>
        </apply>
        <apply>
          <times/>
          <cn> 4 </cn>
          <ci> S142 </ci>
        </apply>
        <apply>
          <times/>
          <cn> 4 </cn>
          <ci> S143 </ci>
        </apply>
        <apply>
          <times/>
          <cn> 4 </cn>
          <ci> S144 </ci>
        </apply>
        <apply>
          <times/>
          <cn> 4 </cn>
          <ci> S145 </ci>
        </apply>
        <apply>
          <times/>
          <cn> 4 </cn>
          <ci> S147 </ci>
        </apply>
        <apply>
          <times/>
          <cn> 4 </cn>
          <ci> S148 </ci>
        </apply>
        <apply>
          <times/>
          <cn> 4 </cn>
          <ci> S149 </ci>
        </apply>
        <apply>
          <times/>
          <cn> 4 </cn>
          <ci> S150 </ci>
        </apply>
      </apply>
    </math>
</assignmentRule>
<assignmentRule variable="sTie2">
    <math xmlns="http://www.w3.org/1998/Math/MathML">
      <apply>
        <plus/>
        <ci> S7 </ci>
        <ci> S28 </ci>
        <ci> S29 </ci>
        <ci> S30 </ci>
        <ci> S31 </ci>
        <apply>
          <times/>
          <cn> 2 </cn>
```

```
          <ci> S42 </ci>
        </apply>
        <apply>
          <times/>
          <cn> 2 </cn>
          <ci> S43 </ci>
        </apply>
        <apply>
          <times/>
          <cn> 2 </cn>
          <ci> S44 </ci>
        </apply>
        <apply>
          <times/>
          <cn> 2 </cn>
          <ci> S45 </ci>
        </apply>
        <apply>
          <times/>
          <cn> 3 </cn>
          <ci> S50 </ci>
        </apply>
        <apply>
          <times/>
          <cn> 3 </cn>
          <ci> S51 </ci>
        </apply>
        <apply>
          <times/>
          <cn> 3 </cn>
          <ci> S52 </ci>
        </apply>
        <apply>
          <times/>
          <cn> 4 </cn>
          <ci> S56 </ci>
        </apply>
        <apply>
          <times/>
          <cn> 4 </cn>
          <ci> S57 </ci>
        </apply>
      </apply>
    </math>
</assignmentRule>
<assignmentRule variable="Tie1Tie2">
    <math xmlns="http://www.w3.org/1998/Math/MathML">
      <apply>
        <plus/>
        <ci> S10 </ci>
        <ci> S32 </ci>
        <ci> S33 </ci>
        <ci> S34 </ci>
        <ci> S35 </ci>
      </apply>
    </math>
</assignmentRule>
```

```
&lt;assignmentRule variable="surfTie1"&gt;
    &lt;math xmlns="http://www.w3.org/1998/Math/MathML"&gt;
      &lt;apply&gt;
        &lt;plus/&gt;
        &lt;cn&gt; 0 &lt;/cn&gt;
        &lt;ci&gt; S9 &lt;/ci&gt;
      &lt;/apply&gt;
    &lt;/math&gt;
&lt;/assignmentRule&gt;
&lt;assignmentRule variable="sTie1"&gt;
    &lt;math xmlns="http://www.w3.org/1998/Math/MathML"&gt;
      &lt;apply&gt;
        &lt;plus/&gt;
        &lt;cn&gt; 0 &lt;/cn&gt;
        &lt;ci&gt; S8 &lt;/ci&gt;
      &lt;/apply&gt;
    &lt;/math&gt;
&lt;/assignmentRule&gt;
&lt;assignmentRule variable="jTie2"&gt;
    &lt;math xmlns="http://www.w3.org/1998/Math/MathML"&gt;
      &lt;apply&gt;
        &lt;plus/&gt;
        &lt;apply&gt;
          &lt;times/&gt;
          &lt;cn&gt; 4 &lt;/cn&gt;
          &lt;ci&gt; S67 &lt;/ci&gt;
        &lt;/apply&gt;
        &lt;apply&gt;
          &lt;times/&gt;
          &lt;cn&gt; 4 &lt;/cn&gt;
          &lt;ci&gt; S68 &lt;/ci&gt;
        &lt;/apply&gt;
        &lt;apply&gt;
          &lt;times/&gt;
          &lt;cn&gt; 4 &lt;/cn&gt;
          &lt;ci&gt; S69 &lt;/ci&gt;
        &lt;/apply&gt;
        &lt;apply&gt;
          &lt;times/&gt;
          &lt;cn&gt; 4 &lt;/cn&gt;
          &lt;ci&gt; S70 &lt;/ci&gt;
        &lt;/apply&gt;
        &lt;apply&gt;
          &lt;times/&gt;
          &lt;cn&gt; 4 &lt;/cn&gt;
          &lt;ci&gt; S71 &lt;/ci&gt;
        &lt;/apply&gt;
        &lt;apply&gt;
          &lt;times/&gt;
          &lt;cn&gt; 4 &lt;/cn&gt;
          &lt;ci&gt; S72 &lt;/ci&gt;
        &lt;/apply&gt;
        &lt;apply&gt;
          &lt;times/&gt;
          &lt;cn&gt; 4 &lt;/cn&gt;
          &lt;ci&gt; S76 &lt;/ci&gt;
        &lt;/apply&gt;
```

```
<apply>
  <times/>
  <cn> 4 </cn>
  <ci> S77 </ci>
</apply>
<apply>
  <times/>
  <cn> 4 </cn>
  <ci> S78 </ci>
</apply>
<apply>
  <times/>
  <cn> 4 </cn>
  <ci> S79 </ci>
</apply>
<apply>
  <times/>
  <cn> 4 </cn>
  <ci> S80 </ci>
</apply>
<apply>
  <times/>
  <cn> 4 </cn>
  <ci> S81 </ci>
</apply>
<apply>
  <times/>
  <cn> 4 </cn>
  <ci> S82 </ci>
</apply>
<apply>
  <times/>
  <cn> 4 </cn>
  <ci> S83 </ci>
</apply>
<apply>
  <times/>
  <cn> 4 </cn>
  <ci> S86 </ci>
</apply>
<apply>
  <times/>
  <cn> 4 </cn>
  <ci> S87 </ci>
</apply>
<apply>
  <times/>
  <cn> 4 </cn>
  <ci> S88 </ci>
</apply>
<apply>
  <times/>
  <cn> 4 </cn>
  <ci> S89 </ci>
</apply>
<apply>
  <times/>
```

```
  <cn> 4 </cn>
  <ci> S90 </ci>
</apply>
<apply>
  <times/>
  <cn> 4 </cn>
  <ci> S91 </ci>
</apply>
<apply>
  <times/>
  <cn> 4 </cn>
  <ci> S92 </ci>
</apply>
<apply>
  <times/>
  <cn> 4 </cn>
  <ci> S93 </ci>
</apply>
<apply>
  <times/>
  <cn> 4 </cn>
  <ci> S94 </ci>
</apply>
<apply>
  <times/>
  <cn> 4 </cn>
  <ci> S95 </ci>
</apply>
<apply>
  <times/>
  <cn> 4 </cn>
  <ci> S99 </ci>
</apply>
<apply>
  <times/>
  <cn> 4 </cn>
  <ci> S100 </ci>
</apply>
<apply>
  <times/>
  <cn> 4 </cn>
  <ci> S101 </ci>
</apply>
<apply>
  <times/>
  <cn> 4 </cn>
  <ci> S102 </ci>
</apply>
<apply>
  <times/>
  <cn> 4 </cn>
  <ci> S103 </ci>
</apply>
<apply>
  <times/>
  <cn> 4 </cn>
  <ci> S104 </ci>
```

```
&lt;/apply&gt;
&lt;apply&gt;
  &lt;times/&gt;
  &lt;cn&gt; 4 &lt;/cn&gt;
  &lt;ci&gt; S105 &lt;/ci&gt;
&lt;/apply&gt;
&lt;apply&gt;
  &lt;times/&gt;
  &lt;cn&gt; 4 &lt;/cn&gt;
  &lt;ci&gt; S106 &lt;/ci&gt;
&lt;/apply&gt;
&lt;apply&gt;
  &lt;times/&gt;
  &lt;cn&gt; 4 &lt;/cn&gt;
  &lt;ci&gt; S107 &lt;/ci&gt;
&lt;/apply&gt;
&lt;apply&gt;
  &lt;times/&gt;
  &lt;cn&gt; 4 &lt;/cn&gt;
  &lt;ci&gt; S108 &lt;/ci&gt;
&lt;/apply&gt;
&lt;apply&gt;
  &lt;times/&gt;
  &lt;cn&gt; 4 &lt;/cn&gt;
  &lt;ci&gt; S109 &lt;/ci&gt;
&lt;/apply&gt;
&lt;apply&gt;
  &lt;times/&gt;
  &lt;cn&gt; 4 &lt;/cn&gt;
  &lt;ci&gt; S110 &lt;/ci&gt;
&lt;/apply&gt;
&lt;apply&gt;
  &lt;times/&gt;
  &lt;cn&gt; 4 &lt;/cn&gt;
  &lt;ci&gt; S111 &lt;/ci&gt;
&lt;/apply&gt;
&lt;apply&gt;
  &lt;times/&gt;
  &lt;cn&gt; 4 &lt;/cn&gt;
  &lt;ci&gt; S112 &lt;/ci&gt;
&lt;/apply&gt;
&lt;apply&gt;
  &lt;times/&gt;
  &lt;cn&gt; 4 &lt;/cn&gt;
  &lt;ci&gt; S114 &lt;/ci&gt;
&lt;/apply&gt;
&lt;apply&gt;
  &lt;times/&gt;
  &lt;cn&gt; 4 &lt;/cn&gt;
  &lt;ci&gt; S115 &lt;/ci&gt;
&lt;/apply&gt;
&lt;apply&gt;
  &lt;times/&gt;
  &lt;cn&gt; 4 &lt;/cn&gt;
  &lt;ci&gt; S116 &lt;/ci&gt;
&lt;/apply&gt;
&lt;apply&gt;
```

```
  <times/>
  <cn> 4 </cn>
  <ci> S117 </ci>
</apply>
<apply>
  <times/>
  <cn> 4 </cn>
  <ci> S118 </ci>
</apply>
<apply>
  <times/>
  <cn> 4 </cn>
  <ci> S119 </ci>
</apply>
<apply>
  <times/>
  <cn> 4 </cn>
  <ci> S120 </ci>
</apply>
<apply>
  <times/>
  <cn> 4 </cn>
  <ci> S121 </ci>
</apply>
<apply>
  <times/>
  <cn> 4 </cn>
  <ci> S122 </ci>
</apply>
<apply>
  <times/>
  <cn> 4 </cn>
  <ci> S123 </ci>
</apply>
<apply>
  <times/>
  <cn> 4 </cn>
  <ci> S124 </ci>
</apply>
<apply>
  <times/>
  <cn> 4 </cn>
  <ci> S125 </ci>
</apply>
<apply>
  <times/>
  <cn> 4 </cn>
  <ci> S128 </ci>
</apply>
<apply>
  <times/>
  <cn> 4 </cn>
  <ci> S129 </ci>
</apply>
<apply>
  <times/>
  <cn> 4 </cn>
```

```
  <ci> S130 </ci>
</apply>
<apply>
  <times/>
  <cn> 4 </cn>
  <ci> S131 </ci>
</apply>
<apply>
  <times/>
  <cn> 4 </cn>
  <ci> S132 </ci>
</apply>
<apply>
  <times/>
  <cn> 4 </cn>
  <ci> S133 </ci>
</apply>
<apply>
  <times/>
  <cn> 4 </cn>
  <ci> S134 </ci>
</apply>
<apply>
  <times/>
  <cn> 4 </cn>
  <ci> S135 </ci>
</apply>
<apply>
  <times/>
  <cn> 4 </cn>
  <ci> S136 </ci>
</apply>
<apply>
  <times/>
  <cn> 4 </cn>
  <ci> S137 </ci>
</apply>
<apply>
  <times/>
  <cn> 4 </cn>
  <ci> S140 </ci>
</apply>
<apply>
  <times/>
  <cn> 4 </cn>
  <ci> S141 </ci>
</apply>
<apply>
  <times/>
  <cn> 4 </cn>
  <ci> S142 </ci>
</apply>
<apply>
  <times/>
  <cn> 4 </cn>
  <ci> S143 </ci>
</apply>
```

```
          <apply>
            <times/>
            <cn> 4 </cn>
            <ci> S144 </ci>
          </apply>
          <apply>
            <times/>
            <cn> 4 </cn>
            <ci> S145 </ci>
          </apply>
          <apply>
            <times/>
            <cn> 4 </cn>
            <ci> S147 </ci>
          </apply>
          <apply>
            <times/>
            <cn> 4 </cn>
            <ci> S148 </ci>
          </apply>
          <apply>
            <times/>
            <cn> 4 </cn>
            <ci> S149 </ci>
          </apply>
          <apply>
            <times/>
            <cn> 4 </cn>
            <ci> S150 </ci>
          </apply>
        </apply>
      </math>
  </assignmentRule>
  <assignmentRule variable="tsurfTie1">
      <math xmlns="http://www.w3.org/1998/Math/MathML">
        <apply>
          <plus/>
          <ci> S9 </ci>
          <ci> S10 </ci>
          <ci> S32 </ci>
          <ci> S33 </ci>
          <ci> S34 </ci>
          <ci> S35 </ci>
        </apply>
      </math>
  </assignmentRule>
  <assignmentRule variable="jTie1">
      <math xmlns="http://www.w3.org/1998/Math/MathML">
        <apply>
          <plus/>
          <ci> S27 </ci>
          <ci> S69 </ci>
          <ci> S70 </ci>
          <ci> S76 </ci>
          <ci> S77 </ci>
          <ci> S78 </ci>
          <ci> S79 </ci>
```

```
        <ci> S86 </ci>
        <ci> S87 </ci>
        <ci> S88 </ci>
        <ci> S89 </ci>
        <ci> S94 </ci>
        <ci> S95 </ci>
        <ci> S99 </ci>
        <ci> S100 </ci>
        <ci> S101 </ci>
        <ci> S102 </ci>
        <ci> S107 </ci>
        <ci> S108 </ci>
        <ci> S109 </ci>
        <ci> S111 </ci>
        <ci> S114 </ci>
        <ci> S115 </ci>
        <ci> S118 </ci>
        <ci> S119 </ci>
        <ci> S120 </ci>
        <ci> S122 </ci>
        <ci> S124 </ci>
        <ci> S125 </ci>
        <ci> S128 </ci>
        <ci> S130 </ci>
        <ci> S132 </ci>
        <ci> S133 </ci>
        <ci> S134 </ci>
        <ci> S136 </ci>
        <ci> S140 </ci>
        <ci> S142 </ci>
        <ci> S144 </ci>
        <ci> S145 </ci>
        <ci> S147 </ci>
        <ci> S149 </ci>
      </apply>
    </math>
</assignmentRule>
<assignmentRule variable="freepip2">
    <math xmlns="http://www.w3.org/1998/Math/MathML">
      <apply>
        <plus/>
        <cn> 0 </cn>
        <ci> S12 </ci>
      </apply>
    </math>
</assignmentRule>
<assignmentRule variable="freepip3">
    <math xmlns="http://www.w3.org/1998/Math/MathML">
      <apply>
        <plus/>
        <cn> 0 </cn>
        <ci> S84 </ci>
      </apply>
    </math>
</assignmentRule>
<assignmentRule variable="ppAkt">
    <math xmlns="http://www.w3.org/1998/Math/MathML">
```

```
        <apply>
          <plus/>
          <ci> S127 </ci>
          <ci> S138 </ci>
        </apply>
      </math>
  </assignmentRule>
  <assignmentRule variable="freeSrc">
      <math xmlns="http://www.w3.org/1998/Math/MathML">
        <apply>
          <plus/>
          <ci> S18 </ci>
          <ci> S36 </ci>
        </apply>
      </math>
  </assignmentRule>
  <assignmentRule variable="pSrc">
      <math xmlns="http://www.w3.org/1998/Math/MathML">
        <apply>
          <plus/>
          <cn> 0 </cn>
          <ci> S36 </ci>
        </apply>
      </math>
  </assignmentRule>
  <assignmentRule variable="pVECadherin">
      <math xmlns="http://www.w3.org/1998/Math/MathML">
        <apply>
          <plus/>
          <ci> S46 </ci>
          <ci> S53 </ci>
        </apply>
      </math>
  </assignmentRule>
  <assignmentRule variable="aABIN2">
      <math xmlns="http://www.w3.org/1998/Math/MathML">
        <apply>
          <plus/>
          <cn> 0 </cn>
          <ci> S75 </ci>
        </apply>
      </math>
  </assignmentRule>
  <assignmentRule variable="totalAkt">
      <math xmlns="http://www.w3.org/1998/Math/MathML">
        <apply>
          <plus/>
          <ci> S14 </ci>
          <ci> S97 </ci>
          <ci> S113 </ci>
          <ci> S126 </ci>
          <ci> S127 </ci>
          <ci> S138 </ci>
          <ci> S139 </ci>
          <ci> S146 </ci>
        </apply>
      </math>
```

```xml
  </assignmentRule>
  <assignmentRule variable="RhoAGTP">
      <math xmlns="http://www.w3.org/1998/Math/MathML">
        <apply>
          <plus/>
          <ci> S74 </ci>
          <ci> S85 </ci>
          <ci> S98 </ci>
        </apply>
      </math>
  </assignmentRule>
  <assignmentRule variable="RhoAmDia">
      <math xmlns="http://www.w3.org/1998/Math/MathML">
        <apply>
          <plus/>
          <ci> S85 </ci>
          <ci> S98 </ci>
        </apply>
      </math>
  </assignmentRule>
  <assignmentRule variable="mDiaSrc">
      <math xmlns="http://www.w3.org/1998/Math/MathML">
        <apply>
          <plus/>
          <cn> 0 </cn>
          <ci> S98 </ci>
        </apply>
      </math>
  </assignmentRule>
  <!-- Global functions -->
  <assignmentRule variable="_rateLaw1">
    <math xmlns="http://www.w3.org/1998/Math/MathML">
      <apply>
        <divide/>
        <ci> kcatPI3KPIP2 </ci>
        <apply>
          <plus/>
          <ci> kmPIP2PI3K </ci>
          <ci> freepip2 </ci>
        </apply>
      </apply>
    </math>
  </assignmentRule>
  <assignmentRule variable="_rateLaw2">
    <math xmlns="http://www.w3.org/1998/Math/MathML">
      <apply>
        <divide/>
        <ci> kcatPTENPIP3 </ci>
        <apply>
          <plus/>
          <ci> kmPIP3PTEN </ci>
          <ci> freepip3 </ci>
        </apply>
      </apply>
    </math>
  </assignmentRule>
</listOfRules>
```

```xml
<listOfReactions>
  <reaction id="R1" reversible="false">
    <listOfReactants>
      <speciesReference species="S1"/>
      <speciesReference species="S2"/>
    </listOfReactants>
    <listOfProducts>
      <speciesReference species="S23"/>
    </listOfProducts>
    <kineticLaw>
      <math xmlns="http://www.w3.org/1998/Math/MathML">
        <apply>
          <times/>
          <cn> 4 </cn>
          <ci> konang1tie2_1 </ci>
          <ci> S1 </ci>
          <ci> S2 </ci>
        </apply>
      </math>
    </kineticLaw>
  </reaction>
  <reaction id="R2" reversible="false">
    <listOfReactants>
      <speciesReference species="S1"/>
      <speciesReference species="S3"/>
    </listOfReactants>
    <listOfProducts>
      <speciesReference species="S24"/>
    </listOfProducts>
    <kineticLaw>
      <math xmlns="http://www.w3.org/1998/Math/MathML">
        <apply>
          <times/>
          <cn> 2 </cn>
          <ci> konang2_2tie2_1 </ci>
          <ci> S1 </ci>
          <ci> S3 </ci>
        </apply>
      </math>
    </kineticLaw>
  </reaction>
  <reaction id="R3" reversible="false">
    <listOfReactants>
      <speciesReference species="S1"/>
      <speciesReference species="S4"/>
    </listOfReactants>
    <listOfProducts>
      <speciesReference species="S25"/>
    </listOfProducts>
    <kineticLaw>
      <math xmlns="http://www.w3.org/1998/Math/MathML">
        <apply>
          <times/>
          <cn> 3 </cn>
          <ci> konang2_3tie2_1 </ci>
          <ci> S1 </ci>
          <ci> S4 </ci>
```

```
      </apply>
    </math>
  </kineticLaw>
</reaction>
<reaction id="R4" reversible="false">
  <listOfReactants>
    <speciesReference species="S1"/>
    <speciesReference species="S5"/>
  </listOfReactants>
  <listOfProducts>
    <speciesReference species="S26"/>
  </listOfProducts>
  <kineticLaw>
    <math xmlns="http://www.w3.org/1998/Math/MathML">
      <apply>
        <times/>
        <cn> 4 </cn>
        <ci> konang2_4tie2_1 </ci>
        <ci> S1 </ci>
        <ci> S5 </ci>
      </apply>
    </math>
  </kineticLaw>
</reaction>
<reaction id="R5" reversible="false">
  <listOfReactants>
    <speciesReference species="S9"/>
  </listOfReactants>
  <listOfProducts>
    <speciesReference species="S27"/>
  </listOfProducts>
  <kineticLaw>
    <math xmlns="http://www.w3.org/1998/Math/MathML">
      <apply>
        <times/>
        <ci> ksjtie1 </ci>
        <ci> S9 </ci>
      </apply>
    </math>
  </kineticLaw>
</reaction>
<reaction id="R6" reversible="false">
  <listOfReactants>
    <speciesReference species="S21"/>
  </listOfReactants>
  <listOfProducts>
    <speciesReference species="S1"/>
    <speciesReference species="S21"/>
  </listOfProducts>
  <kineticLaw>
    <math xmlns="http://www.w3.org/1998/Math/MathML">
      <apply>
        <times/>
        <ci> ksyntie2 </ci>
        <ci> S21 </ci>
      </apply>
    </math>
```

```
      </kineticLaw>
    </reaction>
    <reaction id="R7" reversible="false">
      <listOfReactants>
        <speciesReference species="S21"/>
      </listOfReactants>
      <listOfProducts>
        <speciesReference species="S9"/>
        <speciesReference species="S21"/>
      </listOfProducts>
      <kineticLaw>
        <math xmlns="http://www.w3.org/1998/Math/MathML">
          <apply>
            <times/>
            <ci> ksyntie1 </ci>
            <ci> S21 </ci>
          </apply>
        </math>
      </kineticLaw>
    </reaction>
    <reaction id="R8" reversible="false">
      <listOfReactants>
        <speciesReference species="S2"/>
        <speciesReference species="S7"/>
      </listOfReactants>
      <listOfProducts>
        <speciesReference species="S28"/>
      </listOfProducts>
      <kineticLaw>
        <math xmlns="http://www.w3.org/1998/Math/MathML">
          <apply>
            <times/>
            <cn> 4 </cn>
            <ci> konang1tie2_1 </ci>
            <ci> S2 </ci>
            <ci> S7 </ci>
          </apply>
        </math>
      </kineticLaw>
    </reaction>
    <reaction id="R9" reversible="false">
      <listOfReactants>
        <speciesReference species="S3"/>
        <speciesReference species="S7"/>
      </listOfReactants>
      <listOfProducts>
        <speciesReference species="S29"/>
      </listOfProducts>
      <kineticLaw>
        <math xmlns="http://www.w3.org/1998/Math/MathML">
          <apply>
            <times/>
            <cn> 2 </cn>
            <ci> konang2_2tie2_1 </ci>
            <ci> S3 </ci>
            <ci> S7 </ci>
          </apply>
```

```
    &lt;/math&gt;
  &lt;/kineticLaw&gt;
&lt;/reaction&gt;
&lt;reaction id="R10" reversible="false"&gt;
  &lt;listOfReactants&gt;
    &lt;speciesReference species="S4"/&gt;
    &lt;speciesReference species="S7"/&gt;
  &lt;/listOfReactants&gt;
  &lt;listOfProducts&gt;
    &lt;speciesReference species="S30"/&gt;
  &lt;/listOfProducts&gt;
  &lt;kineticLaw&gt;
    &lt;math xmlns="http://www.w3.org/1998/Math/MathML"&gt;
      &lt;apply&gt;
        &lt;times/&gt;
        &lt;cn&gt; 3 &lt;/cn&gt;
        &lt;ci&gt; konang2_3tie2_1 &lt;/ci&gt;
        &lt;ci&gt; S4 &lt;/ci&gt;
        &lt;ci&gt; S7 &lt;/ci&gt;
      &lt;/apply&gt;
    &lt;/math&gt;
  &lt;/kineticLaw&gt;
&lt;/reaction&gt;
&lt;reaction id="R11" reversible="false"&gt;
  &lt;listOfReactants&gt;
    &lt;speciesReference species="S5"/&gt;
    &lt;speciesReference species="S7"/&gt;
  &lt;/listOfReactants&gt;
  &lt;listOfProducts&gt;
    &lt;speciesReference species="S31"/&gt;
  &lt;/listOfProducts&gt;
  &lt;kineticLaw&gt;
    &lt;math xmlns="http://www.w3.org/1998/Math/MathML"&gt;
      &lt;apply&gt;
        &lt;times/&gt;
        &lt;cn&gt; 4 &lt;/cn&gt;
        &lt;ci&gt; konang2_4tie2_1 &lt;/ci&gt;
        &lt;ci&gt; S5 &lt;/ci&gt;
        &lt;ci&gt; S7 &lt;/ci&gt;
      &lt;/apply&gt;
    &lt;/math&gt;
  &lt;/kineticLaw&gt;
&lt;/reaction&gt;
&lt;reaction id="R12" reversible="false"&gt;
  &lt;listOfReactants&gt;
    &lt;speciesReference species="S1"/&gt;
    &lt;speciesReference species="S9"/&gt;
  &lt;/listOfReactants&gt;
  &lt;listOfProducts&gt;
    &lt;speciesReference species="S10"/&gt;
  &lt;/listOfProducts&gt;
  &lt;kineticLaw&gt;
    &lt;math xmlns="http://www.w3.org/1998/Math/MathML"&gt;
      &lt;apply&gt;
        &lt;times/&gt;
        &lt;ci&gt; kontie1tie2 &lt;/ci&gt;
        &lt;ci&gt; S1 &lt;/ci&gt;
```

```
        &lt;ci&gt; S9 &lt;/ci&gt;
      &lt;/apply&gt;
    &lt;/math&gt;
  &lt;/kineticLaw&gt;
&lt;/reaction&gt;
&lt;reaction id="R13" reversible="false"&gt;
  &lt;listOfReactants&gt;
    &lt;speciesReference species="S10"/&gt;
  &lt;/listOfReactants&gt;
  &lt;listOfProducts&gt;
    &lt;speciesReference species="S1"/&gt;
    &lt;speciesReference species="S9"/&gt;
  &lt;/listOfProducts&gt;
  &lt;kineticLaw&gt;
    &lt;math xmlns="http://www.w3.org/1998/Math/MathML"&gt;
      &lt;apply&gt;
        &lt;times/&gt;
        &lt;ci&gt; kofftie1tie2 &lt;/ci&gt;
        &lt;ci&gt; S10 &lt;/ci&gt;
      &lt;/apply&gt;
    &lt;/math&gt;
  &lt;/kineticLaw&gt;
&lt;/reaction&gt;
&lt;reaction id="R14" reversible="false"&gt;
  &lt;listOfReactants&gt;
    &lt;speciesReference species="S2"/&gt;
    &lt;speciesReference species="S10"/&gt;
  &lt;/listOfReactants&gt;
  &lt;listOfProducts&gt;
    &lt;speciesReference species="S32"/&gt;
  &lt;/listOfProducts&gt;
  &lt;kineticLaw&gt;
    &lt;math xmlns="http://www.w3.org/1998/Math/MathML"&gt;
      &lt;apply&gt;
        &lt;times/&gt;
        &lt;cn&gt; 4 &lt;/cn&gt;
        &lt;ci&gt; konang1_4tie1tie2 &lt;/ci&gt;
        &lt;ci&gt; S2 &lt;/ci&gt;
        &lt;ci&gt; S10 &lt;/ci&gt;
      &lt;/apply&gt;
    &lt;/math&gt;
  &lt;/kineticLaw&gt;
&lt;/reaction&gt;
&lt;reaction id="R15" reversible="false"&gt;
  &lt;listOfReactants&gt;
    &lt;speciesReference species="S3"/&gt;
    &lt;speciesReference species="S10"/&gt;
  &lt;/listOfReactants&gt;
  &lt;listOfProducts&gt;
    &lt;speciesReference species="S33"/&gt;
  &lt;/listOfProducts&gt;
  &lt;kineticLaw&gt;
    &lt;math xmlns="http://www.w3.org/1998/Math/MathML"&gt;
      &lt;apply&gt;
        &lt;times/&gt;
        &lt;cn&gt; 2 &lt;/cn&gt;
        &lt;ci&gt; konang2_2tie1tie2 &lt;/ci&gt;
```

```
        &lt;ci&gt; S3 &lt;/ci&gt;
        &lt;ci&gt; S10 &lt;/ci&gt;
      &lt;/apply&gt;
    &lt;/math&gt;
  &lt;/kineticLaw&gt;
&lt;/reaction&gt;
&lt;reaction id="R16" reversible="false"&gt;
  &lt;listOfReactants&gt;
    &lt;speciesReference species="S4"/&gt;
    &lt;speciesReference species="S10"/&gt;
  &lt;/listOfReactants&gt;
  &lt;listOfProducts&gt;
    &lt;speciesReference species="S34"/&gt;
  &lt;/listOfProducts&gt;
  &lt;kineticLaw&gt;
    &lt;math xmlns="http://www.w3.org/1998/Math/MathML"&gt;
      &lt;apply&gt;
        &lt;times/&gt;
        &lt;cn&gt; 3 &lt;/cn&gt;
        &lt;ci&gt; konang2_3tie1tie2 &lt;/ci&gt;
        &lt;ci&gt; S4 &lt;/ci&gt;
        &lt;ci&gt; S10 &lt;/ci&gt;
      &lt;/apply&gt;
    &lt;/math&gt;
  &lt;/kineticLaw&gt;
&lt;/reaction&gt;
&lt;reaction id="R17" reversible="false"&gt;
  &lt;listOfReactants&gt;
    &lt;speciesReference species="S5"/&gt;
    &lt;speciesReference species="S10"/&gt;
  &lt;/listOfReactants&gt;
  &lt;listOfProducts&gt;
    &lt;speciesReference species="S35"/&gt;
  &lt;/listOfProducts&gt;
  &lt;kineticLaw&gt;
    &lt;math xmlns="http://www.w3.org/1998/Math/MathML"&gt;
      &lt;apply&gt;
        &lt;times/&gt;
        &lt;cn&gt; 4 &lt;/cn&gt;
        &lt;ci&gt; konang2_4tie1tie2 &lt;/ci&gt;
        &lt;ci&gt; S5 &lt;/ci&gt;
        &lt;ci&gt; S10 &lt;/ci&gt;
      &lt;/apply&gt;
    &lt;/math&gt;
  &lt;/kineticLaw&gt;
&lt;/reaction&gt;
&lt;reaction id="R18" reversible="false"&gt;
  &lt;listOfReactants&gt;
    &lt;speciesReference species="S1"/&gt;
  &lt;/listOfReactants&gt;
  &lt;listOfProducts&gt;
    &lt;speciesReference species="S7"/&gt;
  &lt;/listOfProducts&gt;
  &lt;kineticLaw&gt;
    &lt;math xmlns="http://www.w3.org/1998/Math/MathML"&gt;
      &lt;apply&gt;
        &lt;times/&gt;
```

```xml
        <ci> kcleavetie2 </ci>
        <ci> S1 </ci>
      </apply>
    </math>
  </kineticLaw>
</reaction>
<reaction id="R19" reversible="false">
  <listOfReactants>
    <speciesReference species="S9"/>
  </listOfReactants>
  <listOfProducts>
    <speciesReference species="S8"/>
  </listOfProducts>
  <kineticLaw>
    <math xmlns="http://www.w3.org/1998/Math/MathML">
      <apply>
        <times/>
        <ci> kcleavetie1 </ci>
        <ci> S9 </ci>
      </apply>
    </math>
  </kineticLaw>
</reaction>
<reaction id="R20" reversible="false">
  <listOfReactants>
    <speciesReference species="S7"/>
  </listOfReactants>
  <listOfProducts>
    <speciesReference species="S22"/>
  </listOfProducts>
  <kineticLaw>
    <math xmlns="http://www.w3.org/1998/Math/MathML">
      <apply>
        <times/>
        <ci> kdegsTie2 </ci>
        <ci> S7 </ci>
      </apply>
    </math>
  </kineticLaw>
</reaction>
<reaction id="R21" reversible="false">
  <listOfReactants>
    <speciesReference species="S8"/>
  </listOfReactants>
  <listOfProducts>
    <speciesReference species="S22"/>
  </listOfProducts>
  <kineticLaw>
    <math xmlns="http://www.w3.org/1998/Math/MathML">
      <apply>
        <times/>
        <ci> kdegstie1 </ci>
        <ci> S8 </ci>
      </apply>
    </math>
  </kineticLaw>
</reaction>
```

```xml
<reaction id="R22" reversible="false">
  <listOfReactants>
    <speciesReference species="S12"/>
  </listOfReactants>
  <listOfProducts>
    <speciesReference species="S22"/>
  </listOfProducts>
  <kineticLaw>
    <math xmlns="http://www.w3.org/1998/Math/MathML">
      <apply>
        <times/>
        <ci> kPIP2gen </ci>
        <ci> S12 </ci>
      </apply>
    </math>
  </kineticLaw>
</reaction>
<reaction id="R23" reversible="false">
  <listOfReactants>
    <speciesReference species="S18"/>
  </listOfReactants>
  <listOfProducts>
    <speciesReference species="S36"/>
  </listOfProducts>
  <kineticLaw>
    <math xmlns="http://www.w3.org/1998/Math/MathML">
      <apply>
        <times/>
        <ci> kpsrc </ci>
        <ci> S18 </ci>
      </apply>
    </math>
  </kineticLaw>
</reaction>
<reaction id="R24" reversible="false">
  <listOfReactants>
    <speciesReference species="S23"/>
  </listOfReactants>
  <listOfProducts>
    <speciesReference species="S1"/>
    <speciesReference species="S2"/>
  </listOfProducts>
  <kineticLaw>
    <math xmlns="http://www.w3.org/1998/Math/MathML">
      <apply>
        <times/>
        <ci> koffang1tie2_1 </ci>
        <ci> S23 </ci>
      </apply>
    </math>
  </kineticLaw>
</reaction>
<reaction id="R25" reversible="false">
  <listOfReactants>
    <speciesReference species="S1"/>
    <speciesReference species="S23"/>
  </listOfReactants>
```

```
    <listOfProducts>
      <speciesReference species="S37"/>
    </listOfProducts>
    <kineticLaw>
      <math xmlns="http://www.w3.org/1998/Math/MathML">
        <apply>
          <times/>
          <cn> 3 </cn>
          <ci> konang1tie2_2 </ci>
          <ci> S1 </ci>
          <ci> S23 </ci>
        </apply>
      </math>
    </kineticLaw>
  </reaction>
  <reaction id="R26" reversible="false">
    <listOfReactants>
      <speciesReference species="S24"/>
    </listOfReactants>
    <listOfProducts>
      <speciesReference species="S1"/>
      <speciesReference species="S3"/>
    </listOfProducts>
    <kineticLaw>
      <math xmlns="http://www.w3.org/1998/Math/MathML">
        <apply>
          <times/>
          <ci> koffang2_2tie2_1 </ci>
          <ci> S24 </ci>
        </apply>
      </math>
    </kineticLaw>
  </reaction>
  <reaction id="R27" reversible="false">
    <listOfReactants>
      <speciesReference species="S1"/>
      <speciesReference species="S24"/>
    </listOfReactants>
    <listOfProducts>
      <speciesReference species="S38"/>
    </listOfProducts>
    <kineticLaw>
      <math xmlns="http://www.w3.org/1998/Math/MathML">
        <apply>
          <times/>
          <ci> konang2_2tie2_2 </ci>
          <ci> S1 </ci>
          <ci> S24 </ci>
        </apply>
      </math>
    </kineticLaw>
  </reaction>
  <reaction id="R28" reversible="false">
    <listOfReactants>
      <speciesReference species="S25"/>
    </listOfReactants>
    <listOfProducts>
```

```
      <speciesReference species="S1"/>
      <speciesReference species="S4"/>
    </listOfProducts>
    <kineticLaw>
      <math xmlns="http://www.w3.org/1998/Math/MathML">
        <apply>
          <times/>
          <ci> koffang2_3tie2_1 </ci>
          <ci> S25 </ci>
        </apply>
      </math>
    </kineticLaw>
  </reaction>
  <reaction id="R29" reversible="false">
    <listOfReactants>
      <speciesReference species="S1"/>
      <speciesReference species="S25"/>
    </listOfReactants>
    <listOfProducts>
      <speciesReference species="S39"/>
    </listOfProducts>
    <kineticLaw>
      <math xmlns="http://www.w3.org/1998/Math/MathML">
        <apply>
          <times/>
          <cn> 2 </cn>
          <ci> konang2_3tie2_2 </ci>
          <ci> S1 </ci>
          <ci> S25 </ci>
        </apply>
      </math>
    </kineticLaw>
  </reaction>
  <reaction id="R30" reversible="false">
    <listOfReactants>
      <speciesReference species="S26"/>
    </listOfReactants>
    <listOfProducts>
      <speciesReference species="S1"/>
      <speciesReference species="S5"/>
    </listOfProducts>
    <kineticLaw>
      <math xmlns="http://www.w3.org/1998/Math/MathML">
        <apply>
          <times/>
          <ci> koffang2_4tie2_1 </ci>
          <ci> S26 </ci>
        </apply>
      </math>
    </kineticLaw>
  </reaction>
  <reaction id="R31" reversible="false">
    <listOfReactants>
      <speciesReference species="S1"/>
      <speciesReference species="S26"/>
    </listOfReactants>
    <listOfProducts>
```

```
      <speciesReference species="S40"/>
    </listOfProducts>
    <kineticLaw>
      <math xmlns="http://www.w3.org/1998/Math/MathML">
        <apply>
          <times/>
          <cn> 3 </cn>
          <ci> konang2_4tie2_2 </ci>
          <ci> S1 </ci>
          <ci> S26 </ci>
        </apply>
      </math>
    </kineticLaw>
  </reaction>
  <reaction id="R32" reversible="false">
    <listOfReactants>
      <speciesReference species="S27"/>
    </listOfReactants>
    <listOfProducts>
      <speciesReference species="S9"/>
    </listOfProducts>
    <kineticLaw>
      <math xmlns="http://www.w3.org/1998/Math/MathML">
        <apply>
          <times/>
          <ci> kjstie1 </ci>
          <ci> S27 </ci>
        </apply>
      </math>
    </kineticLaw>
  </reaction>
  <reaction id="R33" reversible="false">
    <listOfReactants>
      <speciesReference species="S23"/>
    </listOfReactants>
    <listOfProducts>
      <speciesReference species="S2"/>
      <speciesReference species="S41"/>
    </listOfProducts>
    <kineticLaw>
      <math xmlns="http://www.w3.org/1998/Math/MathML">
        <apply>
          <times/>
          <ci> kintang1tie2_1 </ci>
          <ci> S23 </ci>
        </apply>
      </math>
    </kineticLaw>
  </reaction>
  <reaction id="R34" reversible="false">
    <listOfReactants>
      <speciesReference species="S24"/>
    </listOfReactants>
    <listOfProducts>
      <speciesReference species="S3"/>
      <speciesReference species="S41"/>
    </listOfProducts>
```

```
    <kineticLaw>
      <math xmlns="http://www.w3.org/1998/Math/MathML">
        <apply>
          <times/>
          <ci> kintang2_2tie2_1 </ci>
          <ci> S24 </ci>
        </apply>
      </math>
    </kineticLaw>
  </reaction>
  <reaction id="R35" reversible="false">
    <listOfReactants>
      <speciesReference species="S25"/>
    </listOfReactants>
    <listOfProducts>
      <speciesReference species="S4"/>
      <speciesReference species="S41"/>
    </listOfProducts>
    <kineticLaw>
      <math xmlns="http://www.w3.org/1998/Math/MathML">
        <apply>
          <times/>
          <ci> kintang2_3tie2_1 </ci>
          <ci> S25 </ci>
        </apply>
      </math>
    </kineticLaw>
  </reaction>
  <reaction id="R36" reversible="false">
    <listOfReactants>
      <speciesReference species="S26"/>
    </listOfReactants>
    <listOfProducts>
      <speciesReference species="S5"/>
      <speciesReference species="S41"/>
    </listOfProducts>
    <kineticLaw>
      <math xmlns="http://www.w3.org/1998/Math/MathML">
        <apply>
          <times/>
          <ci> kintang2_4tie2_1 </ci>
          <ci> S26 </ci>
        </apply>
      </math>
    </kineticLaw>
  </reaction>
  <reaction id="R37" reversible="false">
    <listOfReactants>
      <speciesReference species="S28"/>
    </listOfReactants>
    <listOfProducts>
      <speciesReference species="S2"/>
      <speciesReference species="S7"/>
    </listOfProducts>
    <kineticLaw>
      <math xmlns="http://www.w3.org/1998/Math/MathML">
        <apply>
```

```
        <times/>
        <ci> koffang1tie2_1 </ci>
        <ci> S28 </ci>
      </apply>
    </math>
  </kineticLaw>
</reaction>
<reaction id="R38" reversible="false">
  <listOfReactants>
    <speciesReference species="S7"/>
    <speciesReference species="S28"/>
  </listOfReactants>
  <listOfProducts>
    <speciesReference species="S42"/>
  </listOfProducts>
  <kineticLaw>
    <math xmlns="http://www.w3.org/1998/Math/MathML">
      <apply>
        <times/>
        <cn> 3 </cn>
        <ci> konang1tie2_2 </ci>
        <ci> S7 </ci>
        <ci> S28 </ci>
      </apply>
    </math>
  </kineticLaw>
</reaction>
<reaction id="R39" reversible="false">
  <listOfReactants>
    <speciesReference species="S29"/>
  </listOfReactants>
  <listOfProducts>
    <speciesReference species="S3"/>
    <speciesReference species="S7"/>
  </listOfProducts>
  <kineticLaw>
    <math xmlns="http://www.w3.org/1998/Math/MathML">
      <apply>
        <times/>
        <ci> koffang2_2tie2_1 </ci>
        <ci> S29 </ci>
      </apply>
    </math>
  </kineticLaw>
</reaction>
<reaction id="R40" reversible="false">
  <listOfReactants>
    <speciesReference species="S7"/>
    <speciesReference species="S29"/>
  </listOfReactants>
  <listOfProducts>
    <speciesReference species="S43"/>
  </listOfProducts>
  <kineticLaw>
    <math xmlns="http://www.w3.org/1998/Math/MathML">
      <apply>
        <times/>
```

```
        <ci> konang2_2tie2_2 </ci>
        <ci> S7 </ci>
        <ci> S29 </ci>
      </apply>
    </math>
  </kineticLaw>
</reaction>
<reaction id="R41" reversible="false">
  <listOfReactants>
    <speciesReference species="S30"/>
  </listOfReactants>
  <listOfProducts>
    <speciesReference species="S4"/>
    <speciesReference species="S7"/>
  </listOfProducts>
  <kineticLaw>
    <math xmlns="http://www.w3.org/1998/Math/MathML">
      <apply>
        <times/>
        <ci> koffang2_3tie2_1 </ci>
        <ci> S30 </ci>
      </apply>
    </math>
  </kineticLaw>
</reaction>
<reaction id="R42" reversible="false">
  <listOfReactants>
    <speciesReference species="S7"/>
    <speciesReference species="S30"/>
  </listOfReactants>
  <listOfProducts>
    <speciesReference species="S44"/>
  </listOfProducts>
  <kineticLaw>
    <math xmlns="http://www.w3.org/1998/Math/MathML">
      <apply>
        <times/>
        <cn> 2 </cn>
        <ci> konang2_3tie2_2 </ci>
        <ci> S7 </ci>
        <ci> S30 </ci>
      </apply>
    </math>
  </kineticLaw>
</reaction>
<reaction id="R43" reversible="false">
  <listOfReactants>
    <speciesReference species="S31"/>
  </listOfReactants>
  <listOfProducts>
    <speciesReference species="S5"/>
    <speciesReference species="S7"/>
  </listOfProducts>
  <kineticLaw>
    <math xmlns="http://www.w3.org/1998/Math/MathML">
      <apply>
        <times/>
```

```
          <ci> koffang2_4tie2_1 </ci>
          <ci> S31 </ci>
        </apply>
      </math>
    </kineticLaw>
  </reaction>
  <reaction id="R44" reversible="false">
    <listOfReactants>
      <speciesReference species="S7"/>
      <speciesReference species="S31"/>
    </listOfReactants>
    <listOfProducts>
      <speciesReference species="S45"/>
    </listOfProducts>
    <kineticLaw>
      <math xmlns="http://www.w3.org/1998/Math/MathML">
        <apply>
          <times/>
          <cn> 3 </cn>
          <ci> konang2_4tie2_2 </ci>
          <ci> S7 </ci>
          <ci> S31 </ci>
        </apply>
      </math>
    </kineticLaw>
  </reaction>
  <reaction id="R45" reversible="false">
    <listOfReactants>
      <speciesReference species="S32"/>
    </listOfReactants>
    <listOfProducts>
      <speciesReference species="S2"/>
      <speciesReference species="S10"/>
    </listOfProducts>
    <kineticLaw>
      <math xmlns="http://www.w3.org/1998/Math/MathML">
        <apply>
          <times/>
          <ci> koffang1_4tie1tie2 </ci>
          <ci> S32 </ci>
        </apply>
      </math>
    </kineticLaw>
  </reaction>
  <reaction id="R46" reversible="false">
    <listOfReactants>
      <speciesReference species="S32"/>
    </listOfReactants>
    <listOfProducts>
      <speciesReference species="S9"/>
      <speciesReference species="S23"/>
    </listOfProducts>
    <kineticLaw>
      <math xmlns="http://www.w3.org/1998/Math/MathML">
        <apply>
          <times/>
          <ci> kdissang1tie1tie2 </ci>
```

```
          <ci> S32 </ci>
        </apply>
      </math>
    </kineticLaw>
  </reaction>
  <reaction id="R47" reversible="false">
    <listOfReactants>
      <speciesReference species="S33"/>
    </listOfReactants>
    <listOfProducts>
      <speciesReference species="S3"/>
      <speciesReference species="S10"/>
    </listOfProducts>
    <kineticLaw>
      <math xmlns="http://www.w3.org/1998/Math/MathML">
        <apply>
          <times/>
          <ci> koffang2_2tie1tie2 </ci>
          <ci> S33 </ci>
        </apply>
      </math>
    </kineticLaw>
  </reaction>
  <reaction id="R48" reversible="false">
    <listOfReactants>
      <speciesReference species="S34"/>
    </listOfReactants>
    <listOfProducts>
      <speciesReference species="S4"/>
      <speciesReference species="S10"/>
    </listOfProducts>
    <kineticLaw>
      <math xmlns="http://www.w3.org/1998/Math/MathML">
        <apply>
          <times/>
          <ci> koffang2_3tie1tie2 </ci>
          <ci> S34 </ci>
        </apply>
      </math>
    </kineticLaw>
  </reaction>
  <reaction id="R49" reversible="false">
    <listOfReactants>
      <speciesReference species="S35"/>
    </listOfReactants>
    <listOfProducts>
      <speciesReference species="S5"/>
      <speciesReference species="S10"/>
    </listOfProducts>
    <kineticLaw>
      <math xmlns="http://www.w3.org/1998/Math/MathML">
        <apply>
          <times/>
          <ci> koffang2_4tie1tie2 </ci>
          <ci> S35 </ci>
        </apply>
      </math>
```

```
    </kineticLaw>
  </reaction>
  <reaction id="R50" reversible="false">
    <listOfReactants>
      <speciesReference species="S36"/>
    </listOfReactants>
    <listOfProducts>
      <speciesReference species="S18"/>
    </listOfProducts>
    <kineticLaw>
      <math xmlns="http://www.w3.org/1998/Math/MathML">
        <apply>
          <times/>
          <ci> kdpsrc </ci>
          <ci> S36 </ci>
        </apply>
      </math>
    </kineticLaw>
  </reaction>
  <reaction id="R51" reversible="false">
    <listOfReactants>
      <speciesReference species="S19"/>
      <speciesReference species="S36"/>
    </listOfReactants>
    <listOfProducts>
      <speciesReference species="S36"/>
      <speciesReference species="S46"/>
    </listOfProducts>
    <kineticLaw>
      <math xmlns="http://www.w3.org/1998/Math/MathML">
        <apply>
          <times/>
          <ci> kpvecad </ci>
          <ci> S19 </ci>
          <ci> S36 </ci>
        </apply>
      </math>
    </kineticLaw>
  </reaction>
  <reaction id="R52" reversible="false">
    <listOfReactants>
      <speciesReference species="S37"/>
    </listOfReactants>
    <listOfProducts>
      <speciesReference species="S1"/>
      <speciesReference species="S23"/>
    </listOfProducts>
    <kineticLaw>
      <math xmlns="http://www.w3.org/1998/Math/MathML">
        <apply>
          <times/>
          <cn> 2 </cn>
          <ci> koffang1tie2_2 </ci>
          <ci> S37 </ci>
        </apply>
      </math>
    </kineticLaw>
```

```
</reaction>
<reaction id="R53" reversible="false">
  <listOfReactants>
    <speciesReference species="S1"/>
    <speciesReference species="S37"/>
  </listOfReactants>
  <listOfProducts>
    <speciesReference species="S47"/>
  </listOfProducts>
  <kineticLaw>
    <math xmlns="http://www.w3.org/1998/Math/MathML">
      <apply>
        <times/>
        <cn> 2 </cn>
        <ci> konang1tie2_3 </ci>
        <ci> S1 </ci>
        <ci> S37 </ci>
      </apply>
    </math>
  </kineticLaw>
</reaction>
<reaction id="R54" reversible="false">
  <listOfReactants>
    <speciesReference species="S38"/>
  </listOfReactants>
  <listOfProducts>
    <speciesReference species="S1"/>
    <speciesReference species="S24"/>
  </listOfProducts>
  <kineticLaw>
    <math xmlns="http://www.w3.org/1998/Math/MathML">
      <apply>
        <times/>
        <cn> 2 </cn>
        <ci> koffang2_2tie2_2 </ci>
        <ci> S38 </ci>
      </apply>
    </math>
  </kineticLaw>
</reaction>
<reaction id="R55" reversible="false">
  <listOfReactants>
    <speciesReference species="S39"/>
  </listOfReactants>
  <listOfProducts>
    <speciesReference species="S1"/>
    <speciesReference species="S25"/>
  </listOfProducts>
  <kineticLaw>
    <math xmlns="http://www.w3.org/1998/Math/MathML">
      <apply>
        <times/>
        <cn> 2 </cn>
        <ci> koffang2_3tie2_2 </ci>
        <ci> S39 </ci>
      </apply>
    </math>
```

```
    </kineticLaw>
  </reaction>
  <reaction id="R56" reversible="false">
    <listOfReactants>
      <speciesReference species="S1"/>
      <speciesReference species="S39"/>
    </listOfReactants>
    <listOfProducts>
      <speciesReference species="S48"/>
    </listOfProducts>
    <kineticLaw>
      <math xmlns="http://www.w3.org/1998/Math/MathML">
        <apply>
          <times/>
          <ci> konang2_3tie2_3 </ci>
          <ci> S1 </ci>
          <ci> S39 </ci>
        </apply>
      </math>
    </kineticLaw>
  </reaction>
  <reaction id="R57" reversible="false">
    <listOfReactants>
      <speciesReference species="S40"/>
    </listOfReactants>
    <listOfProducts>
      <speciesReference species="S1"/>
      <speciesReference species="S26"/>
    </listOfProducts>
    <kineticLaw>
      <math xmlns="http://www.w3.org/1998/Math/MathML">
        <apply>
          <times/>
          <cn> 2 </cn>
          <ci> koffang2_4tie2_2 </ci>
          <ci> S40 </ci>
        </apply>
      </math>
    </kineticLaw>
  </reaction>
  <reaction id="R58" reversible="false">
    <listOfReactants>
      <speciesReference species="S1"/>
      <speciesReference species="S40"/>
    </listOfReactants>
    <listOfProducts>
      <speciesReference species="S49"/>
    </listOfProducts>
    <kineticLaw>
      <math xmlns="http://www.w3.org/1998/Math/MathML">
        <apply>
          <times/>
          <cn> 2 </cn>
          <ci> konang2_4tie2_3 </ci>
          <ci> S1 </ci>
          <ci> S40 </ci>
        </apply>
```

```
    </math>
  </kineticLaw>
</reaction>
<reaction id="R59" reversible="false">
  <listOfReactants>
    <speciesReference species="S37"/>
  </listOfReactants>
  <listOfProducts>
    <speciesReference species="S2"/>
    <speciesReference species="S41"/>
    <speciesReference species="S41"/>
  </listOfProducts>
  <kineticLaw>
    <math xmlns="http://www.w3.org/1998/Math/MathML">
      <apply>
        <times/>
        <ci> kintang1tie2_2 </ci>
        <ci> S37 </ci>
      </apply>
    </math>
  </kineticLaw>
</reaction>
<reaction id="R60" reversible="false">
  <listOfReactants>
    <speciesReference species="S39"/>
  </listOfReactants>
  <listOfProducts>
    <speciesReference species="S3"/>
    <speciesReference species="S41"/>
    <speciesReference species="S41"/>
  </listOfProducts>
  <kineticLaw>
    <math xmlns="http://www.w3.org/1998/Math/MathML">
      <apply>
        <times/>
        <ci> kintang2_2tie2_2 </ci>
        <ci> S39 </ci>
      </apply>
    </math>
  </kineticLaw>
</reaction>
<reaction id="R61" reversible="false">
  <listOfReactants>
    <speciesReference species="S39"/>
  </listOfReactants>
  <listOfProducts>
    <speciesReference species="S4"/>
    <speciesReference species="S41"/>
    <speciesReference species="S41"/>
  </listOfProducts>
  <kineticLaw>
    <math xmlns="http://www.w3.org/1998/Math/MathML">
      <apply>
        <times/>
        <ci> kintang2_3tie2_2 </ci>
        <ci> S39 </ci>
      </apply>
```

```
      </math>
    </kineticLaw>
  </reaction>
  <reaction id="R62" reversible="false">
    <listOfReactants>
      <speciesReference species="S40"/>
    </listOfReactants>
    <listOfProducts>
      <speciesReference species="S5"/>
      <speciesReference species="S41"/>
      <speciesReference species="S41"/>
    </listOfProducts>
    <kineticLaw>
      <math xmlns="http://www.w3.org/1998/Math/MathML">
        <apply>
          <times/>
          <ci> kintang2_4tie2_2 </ci>
          <ci> S40 </ci>
        </apply>
      </math>
    </kineticLaw>
  </reaction>
  <reaction id="R63" reversible="false">
    <listOfReactants>
      <speciesReference species="S41"/>
    </listOfReactants>
    <listOfProducts>
      <speciesReference species="S1"/>
    </listOfProducts>
    <kineticLaw>
      <math xmlns="http://www.w3.org/1998/Math/MathML">
        <apply>
          <times/>
          <ci> krectie2 </ci>
          <ci> S41 </ci>
        </apply>
      </math>
    </kineticLaw>
  </reaction>
  <reaction id="R64" reversible="false">
    <listOfReactants>
      <speciesReference species="S42"/>
    </listOfReactants>
    <listOfProducts>
      <speciesReference species="S7"/>
      <speciesReference species="S28"/>
    </listOfProducts>
    <kineticLaw>
      <math xmlns="http://www.w3.org/1998/Math/MathML">
        <apply>
          <times/>
          <cn> 2 </cn>
          <ci> koffang1tie2_2 </ci>
          <ci> S42 </ci>
        </apply>
      </math>
    </kineticLaw>
```

```xml
</reaction>
<reaction id="R65" reversible="false">
  <listOfReactants>
    <speciesReference species="S7"/>
    <speciesReference species="S42"/>
  </listOfReactants>
  <listOfProducts>
    <speciesReference species="S50"/>
  </listOfProducts>
  <kineticLaw>
    <math xmlns="http://www.w3.org/1998/Math/MathML">
      <apply>
        <times/>
        <cn> 2 </cn>
        <ci> konang1tie2_3 </ci>
        <ci> S7 </ci>
        <ci> S42 </ci>
      </apply>
    </math>
  </kineticLaw>
</reaction>
<reaction id="R66" reversible="false">
  <listOfReactants>
    <speciesReference species="S43"/>
  </listOfReactants>
  <listOfProducts>
    <speciesReference species="S7"/>
    <speciesReference species="S29"/>
  </listOfProducts>
  <kineticLaw>
    <math xmlns="http://www.w3.org/1998/Math/MathML">
      <apply>
        <times/>
        <cn> 2 </cn>
        <ci> koffang2_2tie2_2 </ci>
        <ci> S43 </ci>
      </apply>
    </math>
  </kineticLaw>
</reaction>
<reaction id="R67" reversible="false">
  <listOfReactants>
    <speciesReference species="S44"/>
  </listOfReactants>
  <listOfProducts>
    <speciesReference species="S7"/>
    <speciesReference species="S30"/>
  </listOfProducts>
  <kineticLaw>
    <math xmlns="http://www.w3.org/1998/Math/MathML">
      <apply>
        <times/>
        <cn> 2 </cn>
        <ci> koffang2_3tie2_2 </ci>
        <ci> S44 </ci>
      </apply>
    </math>
```

```
    &lt;/kineticLaw&gt;
  &lt;/reaction&gt;
  &lt;reaction id="R68" reversible="false"&gt;
    &lt;listOfReactants&gt;
      &lt;speciesReference species="S7"/&gt;
      &lt;speciesReference species="S44"/&gt;
    &lt;/listOfReactants&gt;
    &lt;listOfProducts&gt;
      &lt;speciesReference species="S51"/&gt;
    &lt;/listOfProducts&gt;
    &lt;kineticLaw&gt;
      &lt;math xmlns="http://www.w3.org/1998/Math/MathML"&gt;
        &lt;apply&gt;
          &lt;times/&gt;
          &lt;ci&gt; konang2_3tie2_3 &lt;/ci&gt;
          &lt;ci&gt; S7 &lt;/ci&gt;
          &lt;ci&gt; S44 &lt;/ci&gt;
        &lt;/apply&gt;
      &lt;/math&gt;
    &lt;/kineticLaw&gt;
  &lt;/reaction&gt;
  &lt;reaction id="R69" reversible="false"&gt;
    &lt;listOfReactants&gt;
      &lt;speciesReference species="S45"/&gt;
    &lt;/listOfReactants&gt;
    &lt;listOfProducts&gt;
      &lt;speciesReference species="S7"/&gt;
      &lt;speciesReference species="S31"/&gt;
    &lt;/listOfProducts&gt;
    &lt;kineticLaw&gt;
      &lt;math xmlns="http://www.w3.org/1998/Math/MathML"&gt;
        &lt;apply&gt;
          &lt;times/&gt;
          &lt;cn&gt; 2 &lt;/cn&gt;
          &lt;ci&gt; koffang2_4tie2_2 &lt;/ci&gt;
          &lt;ci&gt; S45 &lt;/ci&gt;
        &lt;/apply&gt;
      &lt;/math&gt;
    &lt;/kineticLaw&gt;
  &lt;/reaction&gt;
  &lt;reaction id="R70" reversible="false"&gt;
    &lt;listOfReactants&gt;
      &lt;speciesReference species="S7"/&gt;
      &lt;speciesReference species="S45"/&gt;
    &lt;/listOfReactants&gt;
    &lt;listOfProducts&gt;
      &lt;speciesReference species="S52"/&gt;
    &lt;/listOfProducts&gt;
    &lt;kineticLaw&gt;
      &lt;math xmlns="http://www.w3.org/1998/Math/MathML"&gt;
        &lt;apply&gt;
          &lt;times/&gt;
          &lt;cn&gt; 2 &lt;/cn&gt;
          &lt;ci&gt; konang2_4tie2_3 &lt;/ci&gt;
          &lt;ci&gt; S7 &lt;/ci&gt;
          &lt;ci&gt; S45 &lt;/ci&gt;
        &lt;/apply&gt;
```

```
    </math>
  </kineticLaw>
</reaction>
<reaction id="R71" reversible="false">
  <listOfReactants>
    <speciesReference species="S36"/>
    <speciesReference species="S46"/>
  </listOfReactants>
  <listOfProducts>
    <speciesReference species="S19"/>
    <speciesReference species="S36"/>
  </listOfProducts>
  <kineticLaw>
    <math xmlns="http://www.w3.org/1998/Math/MathML">
      <apply>
        <times/>
        <ci> kdpvecad </ci>
        <ci> S36 </ci>
        <ci> S46 </ci>
      </apply>
    </math>
  </kineticLaw>
</reaction>
<reaction id="R72" reversible="false">
  <listOfReactants>
    <speciesReference species="S46"/>
  </listOfReactants>
  <listOfProducts>
    <speciesReference species="S53"/>
  </listOfProducts>
  <kineticLaw>
    <math xmlns="http://www.w3.org/1998/Math/MathML">
      <apply>
        <times/>
        <ci> kintvecad </ci>
        <ci> S46 </ci>
      </apply>
    </math>
  </kineticLaw>
</reaction>
<reaction id="R73" reversible="false">
  <listOfReactants>
    <speciesReference species="S47"/>
  </listOfReactants>
  <listOfProducts>
    <speciesReference species="S1"/>
    <speciesReference species="S37"/>
  </listOfProducts>
  <kineticLaw>
    <math xmlns="http://www.w3.org/1998/Math/MathML">
      <apply>
        <times/>
        <cn> 3 </cn>
        <ci> koffang1tie2_3 </ci>
        <ci> S47 </ci>
      </apply>
    </math>
```

```xml
    </kineticLaw>
  </reaction>
  <reaction id="R74" reversible="false">
    <listOfReactants>
      <speciesReference species="S1"/>
      <speciesReference species="S47"/>
    </listOfReactants>
    <listOfProducts>
      <speciesReference species="S54"/>
    </listOfProducts>
    <kineticLaw>
      <math xmlns="http://www.w3.org/1998/Math/MathML">
        <apply>
          <times/>
          <ci> konang1tie2_4 </ci>
          <ci> S1 </ci>
          <ci> S47 </ci>
        </apply>
      </math>
    </kineticLaw>
  </reaction>
  <reaction id="R75" reversible="false">
    <listOfReactants>
      <speciesReference species="S48"/>
    </listOfReactants>
    <listOfProducts>
      <speciesReference species="S1"/>
      <speciesReference species="S39"/>
    </listOfProducts>
    <kineticLaw>
      <math xmlns="http://www.w3.org/1998/Math/MathML">
        <apply>
          <times/>
          <cn> 3 </cn>
          <ci> koffang2_3tie2_3 </ci>
          <ci> S48 </ci>
        </apply>
      </math>
    </kineticLaw>
  </reaction>
  <reaction id="R76" reversible="false">
    <listOfReactants>
      <speciesReference species="S49"/>
    </listOfReactants>
    <listOfProducts>
      <speciesReference species="S1"/>
      <speciesReference species="S40"/>
    </listOfProducts>
    <kineticLaw>
      <math xmlns="http://www.w3.org/1998/Math/MathML">
        <apply>
          <times/>
          <cn> 3 </cn>
          <ci> koffang2_4tie2_3 </ci>
          <ci> S49 </ci>
        </apply>
      </math>
```

```
  </kineticLaw>
</reaction>
<reaction id="R77" reversible="false">
  <listOfReactants>
    <speciesReference species="S1"/>
    <speciesReference species="S49"/>
  </listOfReactants>
  <listOfProducts>
    <speciesReference species="S55"/>
  </listOfProducts>
  <kineticLaw>
    <math xmlns="http://www.w3.org/1998/Math/MathML">
      <apply>
        <times/>
        <ci> konang2_4tie2_4 </ci>
        <ci> S1 </ci>
        <ci> S49 </ci>
      </apply>
    </math>
  </kineticLaw>
</reaction>
<reaction id="R78" reversible="false">
  <listOfReactants>
    <speciesReference species="S47"/>
  </listOfReactants>
  <listOfProducts>
    <speciesReference species="S2"/>
    <speciesReference species="S41"/>
    <speciesReference species="S41"/>
    <speciesReference species="S41"/>
  </listOfProducts>
  <kineticLaw>
    <math xmlns="http://www.w3.org/1998/Math/MathML">
      <apply>
        <times/>
        <cn> 1 </cn>
        <ci> kintang1tie2_3 </ci>
        <ci> S47 </ci>
      </apply>
    </math>
  </kineticLaw>
</reaction>
<reaction id="R79" reversible="false">
  <listOfReactants>
    <speciesReference species="S48"/>
  </listOfReactants>
  <listOfProducts>
    <speciesReference species="S4"/>
    <speciesReference species="S41"/>
    <speciesReference species="S41"/>
    <speciesReference species="S41"/>
  </listOfProducts>
  <kineticLaw>
    <math xmlns="http://www.w3.org/1998/Math/MathML">
      <apply>
        <times/>
        <cn> 1 </cn>
```

```
        &lt;ci&gt; kintang2_3tie2_3 &lt;/ci&gt;
        &lt;ci&gt; S48 &lt;/ci&gt;
      &lt;/apply&gt;
    &lt;/math&gt;
  &lt;/kineticLaw&gt;
&lt;/reaction&gt;
&lt;reaction id="R80" reversible="false"&gt;
  &lt;listOfReactants&gt;
    &lt;speciesReference species="S49"/&gt;
  &lt;/listOfReactants&gt;
  &lt;listOfProducts&gt;
    &lt;speciesReference species="S5"/&gt;
    &lt;speciesReference species="S41"/&gt;
    &lt;speciesReference species="S41"/&gt;
    &lt;speciesReference species="S41"/&gt;
  &lt;/listOfProducts&gt;
  &lt;kineticLaw&gt;
    &lt;math xmlns="http://www.w3.org/1998/Math/MathML"&gt;
      &lt;apply&gt;
        &lt;times/&gt;
        &lt;cn&gt; 1 &lt;/cn&gt;
        &lt;ci&gt; kintang2_4tie2_3 &lt;/ci&gt;
        &lt;ci&gt; S49 &lt;/ci&gt;
      &lt;/apply&gt;
    &lt;/math&gt;
  &lt;/kineticLaw&gt;
&lt;/reaction&gt;
&lt;reaction id="R81" reversible="false"&gt;
  &lt;listOfReactants&gt;
    &lt;speciesReference species="S50"/&gt;
  &lt;/listOfReactants&gt;
  &lt;listOfProducts&gt;
    &lt;speciesReference species="S7"/&gt;
    &lt;speciesReference species="S42"/&gt;
  &lt;/listOfProducts&gt;
  &lt;kineticLaw&gt;
    &lt;math xmlns="http://www.w3.org/1998/Math/MathML"&gt;
      &lt;apply&gt;
        &lt;times/&gt;
        &lt;cn&gt; 3 &lt;/cn&gt;
        &lt;ci&gt; koffang1tie2_3 &lt;/ci&gt;
        &lt;ci&gt; S50 &lt;/ci&gt;
      &lt;/apply&gt;
    &lt;/math&gt;
  &lt;/kineticLaw&gt;
&lt;/reaction&gt;
&lt;reaction id="R82" reversible="false"&gt;
  &lt;listOfReactants&gt;
    &lt;speciesReference species="S7"/&gt;
    &lt;speciesReference species="S50"/&gt;
  &lt;/listOfReactants&gt;
  &lt;listOfProducts&gt;
    &lt;speciesReference species="S56"/&gt;
  &lt;/listOfProducts&gt;
  &lt;kineticLaw&gt;
    &lt;math xmlns="http://www.w3.org/1998/Math/MathML"&gt;
      &lt;apply&gt;
```

```xml
          &lt;times/&gt;
          &lt;ci&gt; konang1tie2_4 &lt;/ci&gt;
          &lt;ci&gt; S7 &lt;/ci&gt;
          &lt;ci&gt; S50 &lt;/ci&gt;
        &lt;/apply&gt;
      &lt;/math&gt;
    &lt;/kineticLaw&gt;
  &lt;/reaction&gt;
  &lt;reaction id="R83" reversible="false"&gt;
    &lt;listOfReactants&gt;
      &lt;speciesReference species="S51"/&gt;
    &lt;/listOfReactants&gt;
    &lt;listOfProducts&gt;
      &lt;speciesReference species="S7"/&gt;
      &lt;speciesReference species="S44"/&gt;
    &lt;/listOfProducts&gt;
    &lt;kineticLaw&gt;
      &lt;math xmlns="http://www.w3.org/1998/Math/MathML"&gt;
        &lt;apply&gt;
          &lt;times/&gt;
          &lt;cn&gt; 3 &lt;/cn&gt;
          &lt;ci&gt; koffang2_3tie2_3 &lt;/ci&gt;
          &lt;ci&gt; S51 &lt;/ci&gt;
        &lt;/apply&gt;
      &lt;/math&gt;
    &lt;/kineticLaw&gt;
  &lt;/reaction&gt;
  &lt;reaction id="R84" reversible="false"&gt;
    &lt;listOfReactants&gt;
      &lt;speciesReference species="S52"/&gt;
    &lt;/listOfReactants&gt;
    &lt;listOfProducts&gt;
      &lt;speciesReference species="S7"/&gt;
      &lt;speciesReference species="S45"/&gt;
    &lt;/listOfProducts&gt;
    &lt;kineticLaw&gt;
      &lt;math xmlns="http://www.w3.org/1998/Math/MathML"&gt;
        &lt;apply&gt;
          &lt;times/&gt;
          &lt;cn&gt; 3 &lt;/cn&gt;
          &lt;ci&gt; koffang2_4tie2_3 &lt;/ci&gt;
          &lt;ci&gt; S52 &lt;/ci&gt;
        &lt;/apply&gt;
      &lt;/math&gt;
    &lt;/kineticLaw&gt;
  &lt;/reaction&gt;
  &lt;reaction id="R85" reversible="false"&gt;
    &lt;listOfReactants&gt;
      &lt;speciesReference species="S7"/&gt;
      &lt;speciesReference species="S52"/&gt;
    &lt;/listOfReactants&gt;
    &lt;listOfProducts&gt;
      &lt;speciesReference species="S57"/&gt;
    &lt;/listOfProducts&gt;
    &lt;kineticLaw&gt;
      &lt;math xmlns="http://www.w3.org/1998/Math/MathML"&gt;
        &lt;apply&gt;
```

```
          <times/>
          <ci> konang2_4tie2_4 </ci>
          <ci> S7 </ci>
          <ci> S52 </ci>
        </apply>
      </math>
    </kineticLaw>
  </reaction>
  <reaction id="R86" reversible="false">
    <listOfReactants>
      <speciesReference species="S53"/>
    </listOfReactants>
    <listOfProducts>
      <speciesReference species="S46"/>
    </listOfProducts>
    <kineticLaw>
      <math xmlns="http://www.w3.org/1998/Math/MathML">
        <apply>
          <times/>
          <ci> krecvecad </ci>
          <ci> S53 </ci>
        </apply>
      </math>
    </kineticLaw>
  </reaction>
  <reaction id="R87" reversible="false">
    <listOfReactants>
      <speciesReference species="S53"/>
    </listOfReactants>
    <listOfProducts>
      <speciesReference species="S22"/>
    </listOfProducts>
    <kineticLaw>
      <math xmlns="http://www.w3.org/1998/Math/MathML">
        <apply>
          <times/>
          <ci> kdegvecad </ci>
          <ci> S53 </ci>
        </apply>
      </math>
    </kineticLaw>
  </reaction>
  <reaction id="R88" reversible="false">
    <listOfReactants>
      <speciesReference species="S54"/>
    </listOfReactants>
    <listOfProducts>
      <speciesReference species="S1"/>
      <speciesReference species="S47"/>
    </listOfProducts>
    <kineticLaw>
      <math xmlns="http://www.w3.org/1998/Math/MathML">
        <apply>
          <times/>
          <cn> 4 </cn>
          <ci> koffang1tie2_4 </ci>
          <ci> S54 </ci>
```

```
      </apply>
    </math>
  </kineticLaw>
</reaction>
<reaction id="R89" reversible="false">
  <listOfReactants>
    <speciesReference species="S55"/>
  </listOfReactants>
  <listOfProducts>
    <speciesReference species="S1"/>
    <speciesReference species="S49"/>
  </listOfProducts>
  <kineticLaw>
    <math xmlns="http://www.w3.org/1998/Math/MathML">
      <apply>
        <times/>
        <cn> 4 </cn>
        <ci> koffang2_4tie2_4 </ci>
        <ci> S55 </ci>
      </apply>
    </math>
  </kineticLaw>
</reaction>
<reaction id="R90" reversible="false">
  <listOfReactants>
    <speciesReference species="S54"/>
  </listOfReactants>
  <listOfProducts>
    <speciesReference species="S58"/>
  </listOfProducts>
  <kineticLaw>
    <math xmlns="http://www.w3.org/1998/Math/MathML">
      <apply>
        <times/>
        <cn> 4 </cn>
        <ci> kpang1tie2 </ci>
        <ci> S54 </ci>
      </apply>
    </math>
  </kineticLaw>
</reaction>
<reaction id="R91" reversible="false">
  <listOfReactants>
    <speciesReference species="S55"/>
  </listOfReactants>
  <listOfProducts>
    <speciesReference species="S59"/>
  </listOfProducts>
  <kineticLaw>
    <math xmlns="http://www.w3.org/1998/Math/MathML">
      <apply>
        <times/>
        <cn> 4 </cn>
        <ci> kpang2tie2 </ci>
        <ci> S55 </ci>
      </apply>
    </math>
```

```xml
      &lt;/kineticLaw&gt;
    &lt;/reaction&gt;
    &lt;reaction id="R92" reversible="false"&gt;
      &lt;listOfReactants&gt;
        &lt;speciesReference species="S54"/&gt;
      &lt;/listOfReactants&gt;
      &lt;listOfProducts&gt;
        &lt;speciesReference species="S2"/&gt;
        &lt;speciesReference species="S41"/&gt;
        &lt;speciesReference species="S41"/&gt;
        &lt;speciesReference species="S41"/&gt;
        &lt;speciesReference species="S41"/&gt;
      &lt;/listOfProducts&gt;
      &lt;kineticLaw&gt;
        &lt;math xmlns="http://www.w3.org/1998/Math/MathML"&gt;
          &lt;apply&gt;
            &lt;times/&gt;
            &lt;cn&gt; 1 &lt;/cn&gt;
            &lt;ci&gt; kintang1tie2_4 &lt;/ci&gt;
            &lt;ci&gt; S54 &lt;/ci&gt;
          &lt;/apply&gt;
        &lt;/math&gt;
      &lt;/kineticLaw&gt;
    &lt;/reaction&gt;
    &lt;reaction id="R93" reversible="false"&gt;
      &lt;listOfReactants&gt;
        &lt;speciesReference species="S55"/&gt;
      &lt;/listOfReactants&gt;
      &lt;listOfProducts&gt;
        &lt;speciesReference species="S5"/&gt;
        &lt;speciesReference species="S41"/&gt;
        &lt;speciesReference species="S41"/&gt;
        &lt;speciesReference species="S41"/&gt;
        &lt;speciesReference species="S41"/&gt;
      &lt;/listOfProducts&gt;
      &lt;kineticLaw&gt;
        &lt;math xmlns="http://www.w3.org/1998/Math/MathML"&gt;
          &lt;apply&gt;
            &lt;times/&gt;
            &lt;cn&gt; 1 &lt;/cn&gt;
            &lt;ci&gt; kintang2_4tie2_4 &lt;/ci&gt;
            &lt;ci&gt; S55 &lt;/ci&gt;
          &lt;/apply&gt;
        &lt;/math&gt;
      &lt;/kineticLaw&gt;
    &lt;/reaction&gt;
    &lt;reaction id="R94" reversible="false"&gt;
      &lt;listOfReactants&gt;
        &lt;speciesReference species="S56"/&gt;
      &lt;/listOfReactants&gt;
      &lt;listOfProducts&gt;
        &lt;speciesReference species="S7"/&gt;
        &lt;speciesReference species="S50"/&gt;
      &lt;/listOfProducts&gt;
      &lt;kineticLaw&gt;
        &lt;math xmlns="http://www.w3.org/1998/Math/MathML"&gt;
          &lt;apply&gt;
```

```
        <times/>
        <cn> 4 </cn>
        <ci> koffang1tie2_4 </ci>
        <ci> S56 </ci>
      </apply>
    </math>
  </kineticLaw>
</reaction>
<reaction id="R95" reversible="false">
  <listOfReactants>
    <speciesReference species="S57"/>
  </listOfReactants>
  <listOfProducts>
    <speciesReference species="S7"/>
    <speciesReference species="S52"/>
  </listOfProducts>
  <kineticLaw>
    <math xmlns="http://www.w3.org/1998/Math/MathML">
      <apply>
        <times/>
        <cn> 4 </cn>
        <ci> koffang2_4tie2_4 </ci>
        <ci> S57 </ci>
      </apply>
    </math>
  </kineticLaw>
</reaction>
<reaction id="R96" reversible="false">
  <listOfReactants>
    <speciesReference species="S58"/>
  </listOfReactants>
  <listOfProducts>
    <speciesReference species="S60"/>
  </listOfProducts>
  <kineticLaw>
    <math xmlns="http://www.w3.org/1998/Math/MathML">
      <apply>
        <times/>
        <cn> 3 </cn>
        <ci> kpang1tie2 </ci>
        <ci> S58 </ci>
      </apply>
    </math>
  </kineticLaw>
</reaction>
<reaction id="R97" reversible="false">
  <listOfReactants>
    <speciesReference species="S58"/>
  </listOfReactants>
  <listOfProducts>
    <speciesReference species="S54"/>
  </listOfProducts>
  <kineticLaw>
    <math xmlns="http://www.w3.org/1998/Math/MathML">
      <apply>
        <times/>
        <ci> kdpang1tie2 </ci>
```

```
        <ci> S58 </ci>
      </apply>
    </math>
  </kineticLaw>
</reaction>
<reaction id="R98" reversible="false">
  <listOfReactants>
    <speciesReference species="S59"/>
  </listOfReactants>
  <listOfProducts>
    <speciesReference species="S61"/>
  </listOfProducts>
  <kineticLaw>
    <math xmlns="http://www.w3.org/1998/Math/MathML">
      <apply>
        <times/>
        <cn> 3 </cn>
        <ci> kpang2tie2 </ci>
        <ci> S59 </ci>
      </apply>
    </math>
  </kineticLaw>
</reaction>
<reaction id="R99" reversible="false">
  <listOfReactants>
    <speciesReference species="S59"/>
  </listOfReactants>
  <listOfProducts>
    <speciesReference species="S55"/>
  </listOfProducts>
  <kineticLaw>
    <math xmlns="http://www.w3.org/1998/Math/MathML">
      <apply>
        <times/>
        <ci> kdpang2tie2 </ci>
        <ci> S59 </ci>
      </apply>
    </math>
  </kineticLaw>
</reaction>
<reaction id="R100" reversible="false">
  <listOfReactants>
    <speciesReference species="S58"/>
  </listOfReactants>
  <listOfProducts>
    <speciesReference species="S2"/>
    <speciesReference species="S41"/>
    <speciesReference species="S41"/>
    <speciesReference species="S41"/>
    <speciesReference species="S62"/>
  </listOfProducts>
  <kineticLaw>
    <math xmlns="http://www.w3.org/1998/Math/MathML">
      <apply>
        <times/>
        <cn> 1 </cn>
        <ci> kintang1p1tie2_4 </ci>
```

```
        <ci> S58 </ci>
      </apply>
    </math>
  </kineticLaw>
</reaction>
<reaction id="R101" reversible="false">
  <listOfReactants>
    <speciesReference species="S59"/>
  </listOfReactants>
  <listOfProducts>
    <speciesReference species="S5"/>
    <speciesReference species="S41"/>
    <speciesReference species="S41"/>
    <speciesReference species="S41"/>
    <speciesReference species="S62"/>
  </listOfProducts>
  <kineticLaw>
    <math xmlns="http://www.w3.org/1998/Math/MathML">
      <apply>
        <times/>
        <cn> 1 </cn>
        <ci> kintang2_4p1tie2_4 </ci>
        <ci> S59 </ci>
      </apply>
    </math>
  </kineticLaw>
</reaction>
<reaction id="R102" reversible="false">
  <listOfReactants>
    <speciesReference species="S60"/>
  </listOfReactants>
  <listOfProducts>
    <speciesReference species="S63"/>
  </listOfProducts>
  <kineticLaw>
    <math xmlns="http://www.w3.org/1998/Math/MathML">
      <apply>
        <times/>
        <cn> 2 </cn>
        <ci> kpang1tie2 </ci>
        <ci> S60 </ci>
      </apply>
    </math>
  </kineticLaw>
</reaction>
<reaction id="R103" reversible="false">
  <listOfReactants>
    <speciesReference species="S60"/>
  </listOfReactants>
  <listOfProducts>
    <speciesReference species="S58"/>
  </listOfProducts>
  <kineticLaw>
    <math xmlns="http://www.w3.org/1998/Math/MathML">
      <apply>
        <times/>
        <cn> 2 </cn>
```

```
        &lt;ci&gt; kdpang1tie2 &lt;/ci&gt;
        &lt;ci&gt; S60 &lt;/ci&gt;
      &lt;/apply&gt;
    &lt;/math&gt;
  &lt;/kineticLaw&gt;
&lt;/reaction&gt;
&lt;reaction id="R104" reversible="false"&gt;
  &lt;listOfReactants&gt;
    &lt;speciesReference species="S61"/&gt;
  &lt;/listOfReactants&gt;
  &lt;listOfProducts&gt;
    &lt;speciesReference species="S64"/&gt;
  &lt;/listOfProducts&gt;
  &lt;kineticLaw&gt;
    &lt;math xmlns="http://www.w3.org/1998/Math/MathML"&gt;
      &lt;apply&gt;
        &lt;times/&gt;
        &lt;cn&gt; 2 &lt;/cn&gt;
        &lt;ci&gt; kpang2tie2 &lt;/ci&gt;
        &lt;ci&gt; S61 &lt;/ci&gt;
      &lt;/apply&gt;
    &lt;/math&gt;
  &lt;/kineticLaw&gt;
&lt;/reaction&gt;
&lt;reaction id="R105" reversible="false"&gt;
  &lt;listOfReactants&gt;
    &lt;speciesReference species="S61"/&gt;
  &lt;/listOfReactants&gt;
  &lt;listOfProducts&gt;
    &lt;speciesReference species="S59"/&gt;
  &lt;/listOfProducts&gt;
  &lt;kineticLaw&gt;
    &lt;math xmlns="http://www.w3.org/1998/Math/MathML"&gt;
      &lt;apply&gt;
        &lt;times/&gt;
        &lt;cn&gt; 2 &lt;/cn&gt;
        &lt;ci&gt; kdpang2tie2 &lt;/ci&gt;
        &lt;ci&gt; S61 &lt;/ci&gt;
      &lt;/apply&gt;
    &lt;/math&gt;
  &lt;/kineticLaw&gt;
&lt;/reaction&gt;
&lt;reaction id="R106" reversible="false"&gt;
  &lt;listOfReactants&gt;
    &lt;speciesReference species="S60"/&gt;
  &lt;/listOfReactants&gt;
  &lt;listOfProducts&gt;
    &lt;speciesReference species="S2"/&gt;
    &lt;speciesReference species="S41"/&gt;
    &lt;speciesReference species="S41"/&gt;
    &lt;speciesReference species="S62"/&gt;
    &lt;speciesReference species="S62"/&gt;
  &lt;/listOfProducts&gt;
  &lt;kineticLaw&gt;
    &lt;math xmlns="http://www.w3.org/1998/Math/MathML"&gt;
      &lt;apply&gt;
        &lt;times/&gt;
```

```
        <ci> kintang1p2tie2_4 </ci>
        <ci> S60 </ci>
      </apply>
    </math>
  </kineticLaw>
</reaction>
<reaction id="R107" reversible="false">
  <listOfReactants>
    <speciesReference species="S61"/>
  </listOfReactants>
  <listOfProducts>
    <speciesReference species="S5"/>
    <speciesReference species="S41"/>
    <speciesReference species="S41"/>
    <speciesReference species="S62"/>
    <speciesReference species="S62"/>
  </listOfProducts>
  <kineticLaw>
    <math xmlns="http://www.w3.org/1998/Math/MathML">
      <apply>
        <times/>
        <ci> kintang2_4p2tie2_4 </ci>
        <ci> S61 </ci>
      </apply>
    </math>
  </kineticLaw>
</reaction>
<reaction id="R108" reversible="false">
  <listOfReactants>
    <speciesReference species="S62"/>
  </listOfReactants>
  <listOfProducts>
    <speciesReference species="S22"/>
  </listOfProducts>
  <kineticLaw>
    <math xmlns="http://www.w3.org/1998/Math/MathML">
      <apply>
        <times/>
        <ci> kdegptie2 </ci>
        <ci> S62 </ci>
      </apply>
    </math>
  </kineticLaw>
</reaction>
<reaction id="R109" reversible="false">
  <listOfReactants>
    <speciesReference species="S63"/>
  </listOfReactants>
  <listOfProducts>
    <speciesReference species="S65"/>
  </listOfProducts>
  <kineticLaw>
    <math xmlns="http://www.w3.org/1998/Math/MathML">
      <apply>
        <times/>
        <ci> kpang1tie2 </ci>
        <ci> S63 </ci>
```

```
      </apply>
    </math>
  </kineticLaw>
</reaction>
<reaction id="R110" reversible="false">
  <listOfReactants>
    <speciesReference species="S63"/>
  </listOfReactants>
  <listOfProducts>
    <speciesReference species="S60"/>
  </listOfProducts>
  <kineticLaw>
    <math xmlns="http://www.w3.org/1998/Math/MathML">
      <apply>
        <times/>
        <cn> 3 </cn>
        <ci> kdpang1tie2 </ci>
        <ci> S63 </ci>
      </apply>
    </math>
  </kineticLaw>
</reaction>
<reaction id="R111" reversible="false">
  <listOfReactants>
    <speciesReference species="S64"/>
  </listOfReactants>
  <listOfProducts>
    <speciesReference species="S66"/>
  </listOfProducts>
  <kineticLaw>
    <math xmlns="http://www.w3.org/1998/Math/MathML">
      <apply>
        <times/>
        <ci> kpang2tie2 </ci>
        <ci> S64 </ci>
      </apply>
    </math>
  </kineticLaw>
</reaction>
<reaction id="R112" reversible="false">
  <listOfReactants>
    <speciesReference species="S64"/>
  </listOfReactants>
  <listOfProducts>
    <speciesReference species="S61"/>
  </listOfProducts>
  <kineticLaw>
    <math xmlns="http://www.w3.org/1998/Math/MathML">
      <apply>
        <times/>
        <cn> 3 </cn>
        <ci> kdpang2tie2 </ci>
        <ci> S64 </ci>
      </apply>
    </math>
  </kineticLaw>
</reaction>
```

```
<reaction id="R113" reversible="false">
  <listOfReactants>
    <speciesReference species="S63"/>
  </listOfReactants>
  <listOfProducts>
    <speciesReference species="S2"/>
    <speciesReference species="S41"/>
    <speciesReference species="S62"/>
    <speciesReference species="S62"/>
    <speciesReference species="S62"/>
  </listOfProducts>
  <kineticLaw>
    <math xmlns="http://www.w3.org/1998/Math/MathML">
      <apply>
        <times/>
        <cn> 1 </cn>
        <ci> kintang1p3tie2_4 </ci>
        <ci> S63 </ci>
      </apply>
    </math>
  </kineticLaw>
</reaction>
<reaction id="R114" reversible="false">
  <listOfReactants>
    <speciesReference species="S64"/>
  </listOfReactants>
  <listOfProducts>
    <speciesReference species="S5"/>
    <speciesReference species="S41"/>
    <speciesReference species="S62"/>
    <speciesReference species="S62"/>
    <speciesReference species="S62"/>
  </listOfProducts>
  <kineticLaw>
    <math xmlns="http://www.w3.org/1998/Math/MathML">
      <apply>
        <times/>
        <cn> 1 </cn>
        <ci> kintang2_4p3tie2_4 </ci>
        <ci> S64 </ci>
      </apply>
    </math>
  </kineticLaw>
</reaction>
<reaction id="R115" reversible="false">
  <listOfReactants>
    <speciesReference species="S65"/>
  </listOfReactants>
  <listOfProducts>
    <speciesReference species="S63"/>
  </listOfProducts>
  <kineticLaw>
    <math xmlns="http://www.w3.org/1998/Math/MathML">
      <apply>
        <times/>
        <cn> 4 </cn>
        <ci> kdpang1tie2 </ci>
```

```
        <ci> S65 </ci>
      </apply>
    </math>
  </kineticLaw>
</reaction>
<reaction id="R116" reversible="false">
  <listOfReactants>
    <speciesReference species="S66"/>
  </listOfReactants>
  <listOfProducts>
    <speciesReference species="S64"/>
  </listOfProducts>
  <kineticLaw>
    <math xmlns="http://www.w3.org/1998/Math/MathML">
      <apply>
        <times/>
        <cn> 4 </cn>
        <ci> kdpang2tie2 </ci>
        <ci> S66 </ci>
      </apply>
    </math>
  </kineticLaw>
</reaction>
<reaction id="R117" reversible="false">
  <listOfReactants>
    <speciesReference species="S65"/>
  </listOfReactants>
  <listOfProducts>
    <speciesReference species="S67"/>
  </listOfProducts>
  <kineticLaw>
    <math xmlns="http://www.w3.org/1998/Math/MathML">
      <apply>
        <times/>
        <cn> 1 </cn>
        <ci> ksjang1tie2 </ci>
        <ci> S65 </ci>
      </apply>
    </math>
  </kineticLaw>
</reaction>
<reaction id="R118" reversible="false">
  <listOfReactants>
    <speciesReference species="S66"/>
  </listOfReactants>
  <listOfProducts>
    <speciesReference species="S68"/>
  </listOfProducts>
  <kineticLaw>
    <math xmlns="http://www.w3.org/1998/Math/MathML">
      <apply>
        <times/>
        <cn> 1 </cn>
        <ci> ksjang2tie2 </ci>
        <ci> S66 </ci>
      </apply>
    </math>
```

```xml
    </kineticLaw>
  </reaction>
  <reaction id="R119" reversible="false">
    <listOfReactants>
      <speciesReference species="S65"/>
    </listOfReactants>
    <listOfProducts>
      <speciesReference species="S2"/>
      <speciesReference species="S62"/>
      <speciesReference species="S62"/>
      <speciesReference species="S62"/>
      <speciesReference species="S62"/>
    </listOfProducts>
    <kineticLaw>
      <math xmlns="http://www.w3.org/1998/Math/MathML">
        <apply>
          <times/>
          <cn> 1 </cn>
          <ci> kintang1p4tie2_4 </ci>
          <ci> S65 </ci>
        </apply>
      </math>
    </kineticLaw>
  </reaction>
  <reaction id="R120" reversible="false">
    <listOfReactants>
      <speciesReference species="S66"/>
    </listOfReactants>
    <listOfProducts>
      <speciesReference species="S5"/>
      <speciesReference species="S62"/>
      <speciesReference species="S62"/>
      <speciesReference species="S62"/>
      <speciesReference species="S62"/>
    </listOfProducts>
    <kineticLaw>
      <math xmlns="http://www.w3.org/1998/Math/MathML">
        <apply>
          <times/>
          <cn> 1 </cn>
          <ci> kintang2_4p4tie2_4 </ci>
          <ci> S66 </ci>
        </apply>
      </math>
    </kineticLaw>
  </reaction>
  <reaction id="R121" reversible="false">
    <listOfReactants>
      <speciesReference species="S67"/>
    </listOfReactants>
    <listOfProducts>
      <speciesReference species="S65"/>
    </listOfProducts>
    <kineticLaw>
      <math xmlns="http://www.w3.org/1998/Math/MathML">
        <apply>
          <times/>
```

```xml
        <cn> 1 </cn>
        <ci> kjsang1tie2 </ci>
        <ci> S67 </ci>
      </apply>
    </math>
  </kineticLaw>
</reaction>
<reaction id="R122" reversible="false">
  <listOfReactants>
    <speciesReference species="S68"/>
  </listOfReactants>
  <listOfProducts>
    <speciesReference species="S66"/>
  </listOfProducts>
  <kineticLaw>
    <math xmlns="http://www.w3.org/1998/Math/MathML">
      <apply>
        <times/>
        <cn> 1 </cn>
        <ci> kjsang2tie2 </ci>
        <ci> S68 </ci>
      </apply>
    </math>
  </kineticLaw>
</reaction>
<reaction id="R123" reversible="false">
  <listOfReactants>
    <speciesReference species="S27"/>
    <speciesReference species="S67"/>
  </listOfReactants>
  <listOfProducts>
    <speciesReference species="S69"/>
  </listOfProducts>
  <kineticLaw>
    <math xmlns="http://www.w3.org/1998/Math/MathML">
      <apply>
        <times/>
        <cn> 4 </cn>
        <ci> konang1tie1tie2_4_j </ci>
        <ci> S27 </ci>
        <ci> S67 </ci>
      </apply>
    </math>
  </kineticLaw>
</reaction>
<reaction id="R124" reversible="false">
  <listOfReactants>
    <speciesReference species="S27"/>
    <speciesReference species="S68"/>
  </listOfReactants>
  <listOfProducts>
    <speciesReference species="S70"/>
  </listOfProducts>
  <kineticLaw>
    <math xmlns="http://www.w3.org/1998/Math/MathML">
      <apply>
        <times/>
```

```
          <cn> 4 </cn>
          <ci> konang2tie1tie2_4_j </ci>
          <ci> S27 </ci>
          <ci> S68 </ci>
        </apply>
      </math>
    </kineticLaw>
  </reaction>
  <reaction id="R125" reversible="false">
    <listOfReactants>
      <speciesReference species="S6"/>
      <speciesReference species="S67"/>
    </listOfReactants>
    <listOfProducts>
      <speciesReference species="S71"/>
    </listOfProducts>
    <kineticLaw>
      <math xmlns="http://www.w3.org/1998/Math/MathML">
        <apply>
          <times/>
          <cn> 4 </cn>
          <ci> konveptp </ci>
          <ci> S6 </ci>
          <ci> S67 </ci>
        </apply>
      </math>
    </kineticLaw>
  </reaction>
  <reaction id="R126" reversible="false">
    <listOfReactants>
      <speciesReference species="S6"/>
      <speciesReference species="S68"/>
    </listOfReactants>
    <listOfProducts>
      <speciesReference species="S72"/>
    </listOfProducts>
    <kineticLaw>
      <math xmlns="http://www.w3.org/1998/Math/MathML">
        <apply>
          <times/>
          <cn> 4 </cn>
          <ci> konveptp </ci>
          <ci> S6 </ci>
          <ci> S68 </ci>
        </apply>
      </math>
    </kineticLaw>
  </reaction>
  <reaction id="R127" reversible="false">
    <listOfReactants>
      <speciesReference species="S13"/>
      <speciesReference species="S67"/>
    </listOfReactants>
    <listOfProducts>
      <speciesReference species="S67"/>
      <speciesReference species="S73"/>
    </listOfProducts>
```

```
      <kineticLaw>
        <math xmlns="http://www.w3.org/1998/Math/MathML">
          <apply>
            <times/>
            <ci> kactPI3KTie2 </ci>
            <ci> S13 </ci>
            <ci> S67 </ci>
          </apply>
        </math>
      </kineticLaw>
    </reaction>
    <reaction id="R128" reversible="false">
      <listOfReactants>
        <speciesReference species="S13"/>
        <speciesReference species="S68"/>
      </listOfReactants>
      <listOfProducts>
        <speciesReference species="S68"/>
        <speciesReference species="S73"/>
      </listOfProducts>
      <kineticLaw>
        <math xmlns="http://www.w3.org/1998/Math/MathML">
          <apply>
            <times/>
            <ci> kactPI3KTie2 </ci>
            <ci> S13 </ci>
            <ci> S68 </ci>
          </apply>
        </math>
      </kineticLaw>
    </reaction>
    <reaction id="R129" reversible="false">
      <listOfReactants>
        <speciesReference species="S16"/>
        <speciesReference species="S67"/>
      </listOfReactants>
      <listOfProducts>
        <speciesReference species="S67"/>
        <speciesReference species="S74"/>
      </listOfProducts>
      <kineticLaw>
        <math xmlns="http://www.w3.org/1998/Math/MathML">
          <apply>
            <times/>
            <ci> kprhoa </ci>
            <ci> S16 </ci>
            <ci> S67 </ci>
          </apply>
        </math>
      </kineticLaw>
    </reaction>
    <reaction id="R130" reversible="false">
      <listOfReactants>
        <speciesReference species="S16"/>
        <speciesReference species="S68"/>
      </listOfReactants>
      <listOfProducts>
```

```
        <speciesReference species="S68"/>
        <speciesReference species="S74"/>
      </listOfProducts>
      <kineticLaw>
        <math xmlns="http://www.w3.org/1998/Math/MathML">
          <apply>
            <times/>
            <ci> kprhoa </ci>
            <ci> S16 </ci>
            <ci> S68 </ci>
          </apply>
        </math>
      </kineticLaw>
    </reaction>
    <reaction id="R131" reversible="false">
      <listOfReactants>
        <speciesReference species="S20"/>
        <speciesReference species="S67"/>
      </listOfReactants>
      <listOfProducts>
        <speciesReference species="S67"/>
        <speciesReference species="S75"/>
      </listOfProducts>
      <kineticLaw>
        <math xmlns="http://www.w3.org/1998/Math/MathML">
          <apply>
            <times/>
            <ci> kactabin2 </ci>
            <ci> S20 </ci>
            <ci> S67 </ci>
          </apply>
        </math>
      </kineticLaw>
    </reaction>
    <reaction id="R132" reversible="false">
      <listOfReactants>
        <speciesReference species="S20"/>
        <speciesReference species="S68"/>
      </listOfReactants>
      <listOfProducts>
        <speciesReference species="S68"/>
        <speciesReference species="S75"/>
      </listOfProducts>
      <kineticLaw>
        <math xmlns="http://www.w3.org/1998/Math/MathML">
          <apply>
            <times/>
            <ci> kactabin2 </ci>
            <ci> S20 </ci>
            <ci> S68 </ci>
          </apply>
        </math>
      </kineticLaw>
    </reaction>
    <reaction id="R133" reversible="false">
      <listOfReactants>
        <speciesReference species="S69"/>
```

```
    </listOfReactants>
    <listOfProducts>
      <speciesReference species="S27"/>
      <speciesReference species="S67"/>
    </listOfProducts>
    <kineticLaw>
      <math xmlns="http://www.w3.org/1998/Math/MathML">
        <apply>
          <times/>
          <ci> koffang1tie1tie2_4_j </ci>
          <ci> S69 </ci>
        </apply>
      </math>
    </kineticLaw>
  </reaction>
  <reaction id="R134" reversible="false">
    <listOfReactants>
      <speciesReference species="S70"/>
    </listOfReactants>
    <listOfProducts>
      <speciesReference species="S27"/>
      <speciesReference species="S68"/>
    </listOfProducts>
    <kineticLaw>
      <math xmlns="http://www.w3.org/1998/Math/MathML">
        <apply>
          <times/>
          <ci> koffang2tie1tie2_4_j </ci>
          <ci> S70 </ci>
        </apply>
      </math>
    </kineticLaw>
  </reaction>
  <reaction id="R135" reversible="false">
    <listOfReactants>
      <speciesReference species="S69"/>
    </listOfReactants>
    <listOfProducts>
      <speciesReference species="S76"/>
    </listOfProducts>
    <kineticLaw>
      <math xmlns="http://www.w3.org/1998/Math/MathML">
        <apply>
          <times/>
          <ci> kptie1ang1_j </ci>
          <ci> S69 </ci>
        </apply>
      </math>
    </kineticLaw>
  </reaction>
  <reaction id="R136" reversible="false">
    <listOfReactants>
      <speciesReference species="S70"/>
    </listOfReactants>
    <listOfProducts>
      <speciesReference species="S77"/>
    </listOfProducts>
```

```
    <kineticLaw>
      <math xmlns="http://www.w3.org/1998/Math/MathML">
        <apply>
          <times/>
          <ci> kptie1ang2_j </ci>
          <ci> S70 </ci>
        </apply>
      </math>
    </kineticLaw>
  </reaction>
  <reaction id="R137" reversible="false">
    <listOfReactants>
      <speciesReference species="S6"/>
      <speciesReference species="S69"/>
    </listOfReactants>
    <listOfProducts>
      <speciesReference species="S78"/>
    </listOfProducts>
    <kineticLaw>
      <math xmlns="http://www.w3.org/1998/Math/MathML">
        <apply>
          <times/>
          <cn> 3 </cn>
          <ci> konveptp </ci>
          <ci> S6 </ci>
          <ci> S69 </ci>
        </apply>
      </math>
    </kineticLaw>
  </reaction>
  <reaction id="R138" reversible="false">
    <listOfReactants>
      <speciesReference species="S6"/>
      <speciesReference species="S70"/>
    </listOfReactants>
    <listOfProducts>
      <speciesReference species="S79"/>
    </listOfProducts>
    <kineticLaw>
      <math xmlns="http://www.w3.org/1998/Math/MathML">
        <apply>
          <times/>
          <cn> 3 </cn>
          <ci> konveptp </ci>
          <ci> S6 </ci>
          <ci> S70 </ci>
        </apply>
      </math>
    </kineticLaw>
  </reaction>
  <reaction id="R139" reversible="false">
    <listOfReactants>
      <speciesReference species="S6"/>
      <speciesReference species="S71"/>
    </listOfReactants>
    <listOfProducts>
      <speciesReference species="S80"/>
```

```
      </listOfProducts>
      <kineticLaw>
        <math xmlns="http://www.w3.org/1998/Math/MathML">
          <apply>
            <times/>
            <cn> 3 </cn>
            <ci> konveptp </ci>
            <ci> S6 </ci>
            <ci> S71 </ci>
          </apply>
        </math>
      </kineticLaw>
    </reaction>
    <reaction id="R140" reversible="false">
      <listOfReactants>
        <speciesReference species="S6"/>
        <speciesReference species="S72"/>
      </listOfReactants>
      <listOfProducts>
        <speciesReference species="S81"/>
      </listOfProducts>
      <kineticLaw>
        <math xmlns="http://www.w3.org/1998/Math/MathML">
          <apply>
            <times/>
            <cn> 3 </cn>
            <ci> konveptp </ci>
            <ci> S6 </ci>
            <ci> S72 </ci>
          </apply>
        </math>
      </kineticLaw>
    </reaction>
    <reaction id="R141" reversible="false">
      <listOfReactants>
        <speciesReference species="S71"/>
      </listOfReactants>
      <listOfProducts>
        <speciesReference species="S6"/>
        <speciesReference species="S67"/>
      </listOfProducts>
      <kineticLaw>
        <math xmlns="http://www.w3.org/1998/Math/MathML">
          <apply>
            <times/>
            <ci> koffveptp </ci>
            <ci> S71 </ci>
          </apply>
        </math>
      </kineticLaw>
    </reaction>
    <reaction id="R142" reversible="false">
      <listOfReactants>
        <speciesReference species="S72"/>
      </listOfReactants>
      <listOfProducts>
        <speciesReference species="S6"/>
```

```
      &lt;speciesReference species="S68"/&gt;
    &lt;/listOfProducts&gt;
    &lt;kineticLaw&gt;
      &lt;math xmlns="http://www.w3.org/1998/Math/MathML"&gt;
        &lt;apply&gt;
          &lt;times/&gt;
          &lt;ci&gt; koffveptp &lt;/ci&gt;
          &lt;ci&gt; S72 &lt;/ci&gt;
        &lt;/apply&gt;
      &lt;/math&gt;
    &lt;/kineticLaw&gt;
  &lt;/reaction&gt;
  &lt;reaction id="R143" reversible="false"&gt;
    &lt;listOfReactants&gt;
      &lt;speciesReference species="S71"/&gt;
    &lt;/listOfReactants&gt;
    &lt;listOfProducts&gt;
      &lt;speciesReference species="S6"/&gt;
      &lt;speciesReference species="S82"/&gt;
    &lt;/listOfProducts&gt;
    &lt;kineticLaw&gt;
      &lt;math xmlns="http://www.w3.org/1998/Math/MathML"&gt;
        &lt;apply&gt;
          &lt;times/&gt;
          &lt;ci&gt; kactveptp_ang1 &lt;/ci&gt;
          &lt;ci&gt; S71 &lt;/ci&gt;
        &lt;/apply&gt;
      &lt;/math&gt;
    &lt;/kineticLaw&gt;
  &lt;/reaction&gt;
  &lt;reaction id="R144" reversible="false"&gt;
    &lt;listOfReactants&gt;
      &lt;speciesReference species="S72"/&gt;
    &lt;/listOfReactants&gt;
    &lt;listOfProducts&gt;
      &lt;speciesReference species="S6"/&gt;
      &lt;speciesReference species="S83"/&gt;
    &lt;/listOfProducts&gt;
    &lt;kineticLaw&gt;
      &lt;math xmlns="http://www.w3.org/1998/Math/MathML"&gt;
        &lt;apply&gt;
          &lt;times/&gt;
          &lt;ci&gt; kactveptp_ang2 &lt;/ci&gt;
          &lt;ci&gt; S72 &lt;/ci&gt;
        &lt;/apply&gt;
      &lt;/math&gt;
    &lt;/kineticLaw&gt;
  &lt;/reaction&gt;
  &lt;reaction id="R145" reversible="false"&gt;
    &lt;listOfReactants&gt;
      &lt;speciesReference species="S13"/&gt;
      &lt;speciesReference species="S69"/&gt;
    &lt;/listOfReactants&gt;
    &lt;listOfProducts&gt;
      &lt;speciesReference species="S69"/&gt;
      &lt;speciesReference species="S73"/&gt;
    &lt;/listOfProducts&gt;
```

```
    <kineticLaw>
      <math xmlns="http://www.w3.org/1998/Math/MathML">
        <apply>
          <times/>
          <ci> kactPI3KTie2 </ci>
          <ci> S13 </ci>
          <ci> S69 </ci>
        </apply>
      </math>
    </kineticLaw>
  </reaction>
  <reaction id="R146" reversible="false">
    <listOfReactants>
      <speciesReference species="S13"/>
      <speciesReference species="S70"/>
    </listOfReactants>
    <listOfProducts>
      <speciesReference species="S70"/>
      <speciesReference species="S73"/>
    </listOfProducts>
    <kineticLaw>
      <math xmlns="http://www.w3.org/1998/Math/MathML">
        <apply>
          <times/>
          <ci> kactPI3KTie2 </ci>
          <ci> S13 </ci>
          <ci> S70 </ci>
        </apply>
      </math>
    </kineticLaw>
  </reaction>
  <reaction id="R147" reversible="false">
    <listOfReactants>
      <speciesReference species="S13"/>
      <speciesReference species="S71"/>
    </listOfReactants>
    <listOfProducts>
      <speciesReference species="S71"/>
      <speciesReference species="S73"/>
    </listOfProducts>
    <kineticLaw>
      <math xmlns="http://www.w3.org/1998/Math/MathML">
        <apply>
          <times/>
          <ci> kactPI3KTie2 </ci>
          <ci> S13 </ci>
          <ci> S71 </ci>
        </apply>
      </math>
    </kineticLaw>
  </reaction>
  <reaction id="R148" reversible="false">
    <listOfReactants>
      <speciesReference species="S13"/>
      <speciesReference species="S72"/>
    </listOfReactants>
    <listOfProducts>
```

```xml
    <speciesReference species="S72"/>
    <speciesReference species="S73"/>
  </listOfProducts>
  <kineticLaw>
    <math xmlns="http://www.w3.org/1998/Math/MathML">
      <apply>
        <times/>
        <ci> kactPI3KTie2 </ci>
        <ci> S13 </ci>
        <ci> S72 </ci>
      </apply>
    </math>
  </kineticLaw>
</reaction>
<reaction id="R149" reversible="false">
  <listOfReactants>
    <speciesReference species="S73"/>
  </listOfReactants>
  <listOfProducts>
    <speciesReference species="S13"/>
  </listOfProducts>
  <kineticLaw>
    <math xmlns="http://www.w3.org/1998/Math/MathML">
      <apply>
        <times/>
        <ci> kinactPI3KTie2 </ci>
        <ci> S73 </ci>
      </apply>
    </math>
  </kineticLaw>
</reaction>
<reaction id="R150" reversible="false">
  <listOfReactants>
    <speciesReference species="S12"/>
    <speciesReference species="S73"/>
  </listOfReactants>
  <listOfProducts>
    <speciesReference species="S73"/>
    <speciesReference species="S84"/>
  </listOfProducts>
  <kineticLaw>
    <math xmlns="http://www.w3.org/1998/Math/MathML">
      <apply>
        <times/>
        <ci> _rateLaw1 </ci>
        <ci> S12 </ci>
        <ci> S73 </ci>
      </apply>
    </math>
  </kineticLaw>
</reaction>
<reaction id="R151" reversible="false">
  <listOfReactants>
    <speciesReference species="S16"/>
    <speciesReference species="S69"/>
  </listOfReactants>
  <listOfProducts>
```

```
    <speciesReference species="S69"/>
    <speciesReference species="S74"/>
  </listOfProducts>
  <kineticLaw>
    <math xmlns="http://www.w3.org/1998/Math/MathML">
      <apply>
        <times/>
        <ci> kprhoa </ci>
        <ci> S16 </ci>
        <ci> S69 </ci>
      </apply>
    </math>
  </kineticLaw>
</reaction>
<reaction id="R152" reversible="false">
  <listOfReactants>
    <speciesReference species="S16"/>
    <speciesReference species="S70"/>
  </listOfReactants>
  <listOfProducts>
    <speciesReference species="S70"/>
    <speciesReference species="S74"/>
  </listOfProducts>
  <kineticLaw>
    <math xmlns="http://www.w3.org/1998/Math/MathML">
      <apply>
        <times/>
        <ci> kprhoa </ci>
        <ci> S16 </ci>
        <ci> S70 </ci>
      </apply>
    </math>
  </kineticLaw>
</reaction>
<reaction id="R153" reversible="false">
  <listOfReactants>
    <speciesReference species="S16"/>
    <speciesReference species="S71"/>
  </listOfReactants>
  <listOfProducts>
    <speciesReference species="S71"/>
    <speciesReference species="S74"/>
  </listOfProducts>
  <kineticLaw>
    <math xmlns="http://www.w3.org/1998/Math/MathML">
      <apply>
        <times/>
        <ci> kprhoa </ci>
        <ci> S16 </ci>
        <ci> S71 </ci>
      </apply>
    </math>
  </kineticLaw>
</reaction>
<reaction id="R154" reversible="false">
  <listOfReactants>
    <speciesReference species="S16"/>
```

```
      <speciesReference species="S72"/>
    </listOfReactants>
    <listOfProducts>
      <speciesReference species="S72"/>
      <speciesReference species="S74"/>
    </listOfProducts>
    <kineticLaw>
      <math xmlns="http://www.w3.org/1998/Math/MathML">
        <apply>
          <times/>
          <ci> kprhoa </ci>
          <ci> S16 </ci>
          <ci> S72 </ci>
        </apply>
      </math>
    </kineticLaw>
  </reaction>
  <reaction id="R155" reversible="false">
    <listOfReactants>
      <speciesReference species="S74"/>
    </listOfReactants>
    <listOfProducts>
      <speciesReference species="S16"/>
    </listOfProducts>
    <kineticLaw>
      <math xmlns="http://www.w3.org/1998/Math/MathML">
        <apply>
          <times/>
          <ci> kdprhoa </ci>
          <ci> S74 </ci>
        </apply>
      </math>
    </kineticLaw>
  </reaction>
  <reaction id="R156" reversible="false">
    <listOfReactants>
      <speciesReference species="S17"/>
      <speciesReference species="S74"/>
    </listOfReactants>
    <listOfProducts>
      <speciesReference species="S85"/>
    </listOfProducts>
    <kineticLaw>
      <math xmlns="http://www.w3.org/1998/Math/MathML">
        <apply>
          <times/>
          <ci> konrhoamdia </ci>
          <ci> S17 </ci>
          <ci> S74 </ci>
        </apply>
      </math>
    </kineticLaw>
  </reaction>
  <reaction id="R157" reversible="false">
    <listOfReactants>
      <speciesReference species="S20"/>
      <speciesReference species="S69"/>
```

```
    </listOfReactants>
    <listOfProducts>
      <speciesReference species="S69"/>
      <speciesReference species="S75"/>
    </listOfProducts>
    <kineticLaw>
      <math xmlns="http://www.w3.org/1998/Math/MathML">
        <apply>
          <times/>
          <ci> kactabin2 </ci>
          <ci> S20 </ci>
          <ci> S69 </ci>
        </apply>
      </math>
    </kineticLaw>
  </reaction>
  <reaction id="R158" reversible="false">
    <listOfReactants>
      <speciesReference species="S20"/>
      <speciesReference species="S70"/>
    </listOfReactants>
    <listOfProducts>
      <speciesReference species="S70"/>
      <speciesReference species="S75"/>
    </listOfProducts>
    <kineticLaw>
      <math xmlns="http://www.w3.org/1998/Math/MathML">
        <apply>
          <times/>
          <ci> kactabin2 </ci>
          <ci> S20 </ci>
          <ci> S70 </ci>
        </apply>
      </math>
    </kineticLaw>
  </reaction>
  <reaction id="R159" reversible="false">
    <listOfReactants>
      <speciesReference species="S20"/>
      <speciesReference species="S71"/>
    </listOfReactants>
    <listOfProducts>
      <speciesReference species="S71"/>
      <speciesReference species="S75"/>
    </listOfProducts>
    <kineticLaw>
      <math xmlns="http://www.w3.org/1998/Math/MathML">
        <apply>
          <times/>
          <ci> kactabin2 </ci>
          <ci> S20 </ci>
          <ci> S71 </ci>
        </apply>
      </math>
    </kineticLaw>
  </reaction>
  <reaction id="R160" reversible="false">
```

```
    <listOfReactants>
      <speciesReference species="S20"/>
      <speciesReference species="S72"/>
    </listOfReactants>
    <listOfProducts>
      <speciesReference species="S72"/>
      <speciesReference species="S75"/>
    </listOfProducts>
    <kineticLaw>
      <math xmlns="http://www.w3.org/1998/Math/MathML">
        <apply>
          <times/>
          <ci> kactabin2 </ci>
          <ci> S20 </ci>
          <ci> S72 </ci>
        </apply>
      </math>
    </kineticLaw>
  </reaction>
  <reaction id="R161" reversible="false">
    <listOfReactants>
      <speciesReference species="S75"/>
    </listOfReactants>
    <listOfProducts>
      <speciesReference species="S20"/>
    </listOfProducts>
    <kineticLaw>
      <math xmlns="http://www.w3.org/1998/Math/MathML">
        <apply>
          <times/>
          <ci> kinactabin2 </ci>
          <ci> S75 </ci>
        </apply>
      </math>
    </kineticLaw>
  </reaction>
  <reaction id="R162" reversible="false">
    <listOfReactants>
      <speciesReference species="S76"/>
    </listOfReactants>
    <listOfProducts>
      <speciesReference species="S69"/>
    </listOfProducts>
    <kineticLaw>
      <math xmlns="http://www.w3.org/1998/Math/MathML">
        <apply>
          <times/>
          <ci> kdpatie1ang1_j </ci>
          <ci> S76 </ci>
        </apply>
      </math>
    </kineticLaw>
  </reaction>
  <reaction id="R163" reversible="false">
    <listOfReactants>
      <speciesReference species="S77"/>
    </listOfReactants>
```

```xml
    &lt;listOfProducts&gt;
      &lt;speciesReference species="S70"/&gt;
    &lt;/listOfProducts&gt;
    &lt;kineticLaw&gt;
      &lt;math xmlns="http://www.w3.org/1998/Math/MathML"&gt;
        &lt;apply&gt;
          &lt;times/&gt;
          &lt;ci&gt; kdpatie1ang2_j &lt;/ci&gt;
          &lt;ci&gt; S77 &lt;/ci&gt;
        &lt;/apply&gt;
      &lt;/math&gt;
    &lt;/kineticLaw&gt;
  &lt;/reaction&gt;
  &lt;reaction id="R164" reversible="false"&gt;
    &lt;listOfReactants&gt;
      &lt;speciesReference species="S6"/&gt;
      &lt;speciesReference species="S76"/&gt;
    &lt;/listOfReactants&gt;
    &lt;listOfProducts&gt;
      &lt;speciesReference species="S86"/&gt;
    &lt;/listOfProducts&gt;
    &lt;kineticLaw&gt;
      &lt;math xmlns="http://www.w3.org/1998/Math/MathML"&gt;
        &lt;apply&gt;
          &lt;times/&gt;
          &lt;cn&gt; 3 &lt;/cn&gt;
          &lt;ci&gt; konveptp &lt;/ci&gt;
          &lt;ci&gt; S6 &lt;/ci&gt;
          &lt;ci&gt; S76 &lt;/ci&gt;
        &lt;/apply&gt;
      &lt;/math&gt;
    &lt;/kineticLaw&gt;
  &lt;/reaction&gt;
  &lt;reaction id="R165" reversible="false"&gt;
    &lt;listOfReactants&gt;
      &lt;speciesReference species="S6"/&gt;
      &lt;speciesReference species="S77"/&gt;
    &lt;/listOfReactants&gt;
    &lt;listOfProducts&gt;
      &lt;speciesReference species="S87"/&gt;
    &lt;/listOfProducts&gt;
    &lt;kineticLaw&gt;
      &lt;math xmlns="http://www.w3.org/1998/Math/MathML"&gt;
        &lt;apply&gt;
          &lt;times/&gt;
          &lt;cn&gt; 3 &lt;/cn&gt;
          &lt;ci&gt; konveptp &lt;/ci&gt;
          &lt;ci&gt; S6 &lt;/ci&gt;
          &lt;ci&gt; S77 &lt;/ci&gt;
        &lt;/apply&gt;
      &lt;/math&gt;
    &lt;/kineticLaw&gt;
  &lt;/reaction&gt;
  &lt;reaction id="R166" reversible="false"&gt;
    &lt;listOfReactants&gt;
      &lt;speciesReference species="S6"/&gt;
      &lt;speciesReference species="S78"/&gt;
```

```xml
    </listOfReactants>
    <listOfProducts>
      <speciesReference species="S88"/>
    </listOfProducts>
    <kineticLaw>
      <math xmlns="http://www.w3.org/1998/Math/MathML">
        <apply>
          <times/>
          <cn> 2 </cn>
          <ci> konveptp </ci>
          <ci> S6 </ci>
          <ci> S78 </ci>
        </apply>
      </math>
    </kineticLaw>
  </reaction>
  <reaction id="R167" reversible="false">
    <listOfReactants>
      <speciesReference species="S6"/>
      <speciesReference species="S79"/>
    </listOfReactants>
    <listOfProducts>
      <speciesReference species="S89"/>
    </listOfProducts>
    <kineticLaw>
      <math xmlns="http://www.w3.org/1998/Math/MathML">
        <apply>
          <times/>
          <cn> 2 </cn>
          <ci> konveptp </ci>
          <ci> S6 </ci>
          <ci> S79 </ci>
        </apply>
      </math>
    </kineticLaw>
  </reaction>
  <reaction id="R168" reversible="false">
    <listOfReactants>
      <speciesReference species="S6"/>
      <speciesReference species="S80"/>
    </listOfReactants>
    <listOfProducts>
      <speciesReference species="S90"/>
    </listOfProducts>
    <kineticLaw>
      <math xmlns="http://www.w3.org/1998/Math/MathML">
        <apply>
          <times/>
          <cn> 2 </cn>
          <ci> konveptp </ci>
          <ci> S6 </ci>
          <ci> S80 </ci>
        </apply>
      </math>
    </kineticLaw>
  </reaction>
  <reaction id="R169" reversible="false">
```

```
      <listOfReactants>
        <speciesReference species="S6"/>
        <speciesReference species="S81"/>
      </listOfReactants>
      <listOfProducts>
        <speciesReference species="S91"/>
      </listOfProducts>
      <kineticLaw>
        <math xmlns="http://www.w3.org/1998/Math/MathML">
          <apply>
            <times/>
            <cn> 2 </cn>
            <ci> konveptp </ci>
            <ci> S6 </ci>
            <ci> S81 </ci>
          </apply>
        </math>
      </kineticLaw>
    </reaction>
    <reaction id="R170" reversible="false">
      <listOfReactants>
        <speciesReference species="S6"/>
        <speciesReference species="S82"/>
      </listOfReactants>
      <listOfProducts>
        <speciesReference species="S92"/>
      </listOfProducts>
      <kineticLaw>
        <math xmlns="http://www.w3.org/1998/Math/MathML">
          <apply>
            <times/>
            <cn> 3 </cn>
            <ci> konveptp </ci>
            <ci> S6 </ci>
            <ci> S82 </ci>
          </apply>
        </math>
      </kineticLaw>
    </reaction>
    <reaction id="R171" reversible="false">
      <listOfReactants>
        <speciesReference species="S6"/>
        <speciesReference species="S83"/>
      </listOfReactants>
      <listOfProducts>
        <speciesReference species="S93"/>
      </listOfProducts>
      <kineticLaw>
        <math xmlns="http://www.w3.org/1998/Math/MathML">
          <apply>
            <times/>
            <cn> 3 </cn>
            <ci> konveptp </ci>
            <ci> S6 </ci>
            <ci> S83 </ci>
          </apply>
        </math>
```

```xml
    </kineticLaw>
  </reaction>
  <reaction id="R172" reversible="false">
    <listOfReactants>
      <speciesReference species="S78"/>
    </listOfReactants>
    <listOfProducts>
      <speciesReference species="S6"/>
      <speciesReference species="S69"/>
    </listOfProducts>
    <kineticLaw>
      <math xmlns="http://www.w3.org/1998/Math/MathML">
        <apply>
          <times/>
          <ci> koffveptp </ci>
          <ci> S78 </ci>
        </apply>
      </math>
    </kineticLaw>
  </reaction>
  <reaction id="R173" reversible="false">
    <listOfReactants>
      <speciesReference species="S79"/>
    </listOfReactants>
    <listOfProducts>
      <speciesReference species="S6"/>
      <speciesReference species="S70"/>
    </listOfProducts>
    <kineticLaw>
      <math xmlns="http://www.w3.org/1998/Math/MathML">
        <apply>
          <times/>
          <ci> koffveptp </ci>
          <ci> S79 </ci>
        </apply>
      </math>
    </kineticLaw>
  </reaction>
  <reaction id="R174" reversible="false">
    <listOfReactants>
      <speciesReference species="S80"/>
    </listOfReactants>
    <listOfProducts>
      <speciesReference species="S6"/>
      <speciesReference species="S71"/>
    </listOfProducts>
    <kineticLaw>
      <math xmlns="http://www.w3.org/1998/Math/MathML">
        <apply>
          <times/>
          <cn> 2 </cn>
          <ci> koffveptp </ci>
          <ci> S80 </ci>
        </apply>
      </math>
    </kineticLaw>
  </reaction>
```

```
<reaction id="R175" reversible="false">
  <listOfReactants>
    <speciesReference species="S81"/>
  </listOfReactants>
  <listOfProducts>
    <speciesReference species="S6"/>
    <speciesReference species="S72"/>
  </listOfProducts>
  <kineticLaw>
    <math xmlns="http://www.w3.org/1998/Math/MathML">
      <apply>
        <times/>
        <cn> 2 </cn>
        <ci> koffveptp </ci>
        <ci> S81 </ci>
      </apply>
    </math>
  </kineticLaw>
</reaction>
<reaction id="R176" reversible="false">
  <listOfReactants>
    <speciesReference species="S78"/>
  </listOfReactants>
  <listOfProducts>
    <speciesReference species="S6"/>
    <speciesReference species="S94"/>
  </listOfProducts>
  <kineticLaw>
    <math xmlns="http://www.w3.org/1998/Math/MathML">
      <apply>
        <times/>
        <ci> kactveptp_ang1 </ci>
        <ci> S78 </ci>
      </apply>
    </math>
  </kineticLaw>
</reaction>
<reaction id="R177" reversible="false">
  <listOfReactants>
    <speciesReference species="S80"/>
  </listOfReactants>
  <listOfProducts>
    <speciesReference species="S6"/>
    <speciesReference species="S92"/>
  </listOfProducts>
  <kineticLaw>
    <math xmlns="http://www.w3.org/1998/Math/MathML">
      <apply>
        <times/>
        <cn> 2 </cn>
        <ci> kactveptp_ang1 </ci>
        <ci> S80 </ci>
      </apply>
    </math>
  </kineticLaw>
</reaction>
<reaction id="R178" reversible="false">
```

```
    <listOfReactants>
      <speciesReference species="S79"/>
    </listOfReactants>
    <listOfProducts>
      <speciesReference species="S6"/>
      <speciesReference species="S95"/>
    </listOfProducts>
    <kineticLaw>
      <math xmlns="http://www.w3.org/1998/Math/MathML">
        <apply>
          <times/>
          <ci> kactveptp_ang2 </ci>
          <ci> S79 </ci>
        </apply>
      </math>
    </kineticLaw>
  </reaction>
  <reaction id="R179" reversible="false">
    <listOfReactants>
      <speciesReference species="S81"/>
    </listOfReactants>
    <listOfProducts>
      <speciesReference species="S6"/>
      <speciesReference species="S93"/>
    </listOfProducts>
    <kineticLaw>
      <math xmlns="http://www.w3.org/1998/Math/MathML">
        <apply>
          <times/>
          <cn> 2 </cn>
          <ci> kactveptp_ang2 </ci>
          <ci> S81 </ci>
        </apply>
      </math>
    </kineticLaw>
  </reaction>
  <reaction id="R180" reversible="false">
    <listOfReactants>
      <speciesReference species="S13"/>
      <speciesReference species="S76"/>
    </listOfReactants>
    <listOfProducts>
      <speciesReference species="S73"/>
      <speciesReference species="S76"/>
    </listOfProducts>
    <kineticLaw>
      <math xmlns="http://www.w3.org/1998/Math/MathML">
        <apply>
          <times/>
          <ci> kactPI3KTie2 </ci>
          <ci> S13 </ci>
          <ci> S76 </ci>
        </apply>
      </math>
    </kineticLaw>
  </reaction>
  <reaction id="R181" reversible="false">
```

```
    <listOfReactants>
      <speciesReference species="S13"/>
      <speciesReference species="S77"/>
    </listOfReactants>
    <listOfProducts>
      <speciesReference species="S73"/>
      <speciesReference species="S77"/>
    </listOfProducts>
    <kineticLaw>
      <math xmlns="http://www.w3.org/1998/Math/MathML">
        <apply>
          <times/>
          <ci> kactPI3KTie2 </ci>
          <ci> S13 </ci>
          <ci> S77 </ci>
        </apply>
      </math>
    </kineticLaw>
  </reaction>
  <reaction id="R182" reversible="false">
    <listOfReactants>
      <speciesReference species="S13"/>
      <speciesReference species="S78"/>
    </listOfReactants>
    <listOfProducts>
      <speciesReference species="S73"/>
      <speciesReference species="S78"/>
    </listOfProducts>
    <kineticLaw>
      <math xmlns="http://www.w3.org/1998/Math/MathML">
        <apply>
          <times/>
          <ci> kactPI3KTie2 </ci>
          <ci> S13 </ci>
          <ci> S78 </ci>
        </apply>
      </math>
    </kineticLaw>
  </reaction>
  <reaction id="R183" reversible="false">
    <listOfReactants>
      <speciesReference species="S13"/>
      <speciesReference species="S79"/>
    </listOfReactants>
    <listOfProducts>
      <speciesReference species="S73"/>
      <speciesReference species="S79"/>
    </listOfProducts>
    <kineticLaw>
      <math xmlns="http://www.w3.org/1998/Math/MathML">
        <apply>
          <times/>
          <ci> kactPI3KTie2 </ci>
          <ci> S13 </ci>
          <ci> S79 </ci>
        </apply>
      </math>
```

```
    </kineticLaw>
  </reaction>
  <reaction id="R184" reversible="false">
    <listOfReactants>
      <speciesReference species="S13"/>
      <speciesReference species="S80"/>
    </listOfReactants>
    <listOfProducts>
      <speciesReference species="S73"/>
      <speciesReference species="S80"/>
    </listOfProducts>
    <kineticLaw>
      <math xmlns="http://www.w3.org/1998/Math/MathML">
        <apply>
          <times/>
          <ci> kactPI3KTie2 </ci>
          <ci> S13 </ci>
          <ci> S80 </ci>
        </apply>
      </math>
    </kineticLaw>
  </reaction>
  <reaction id="R185" reversible="false">
    <listOfReactants>
      <speciesReference species="S13"/>
      <speciesReference species="S81"/>
    </listOfReactants>
    <listOfProducts>
      <speciesReference species="S73"/>
      <speciesReference species="S81"/>
    </listOfProducts>
    <kineticLaw>
      <math xmlns="http://www.w3.org/1998/Math/MathML">
        <apply>
          <times/>
          <ci> kactPI3KTie2 </ci>
          <ci> S13 </ci>
          <ci> S81 </ci>
        </apply>
      </math>
    </kineticLaw>
  </reaction>
  <reaction id="R186" reversible="false">
    <listOfReactants>
      <speciesReference species="S13"/>
      <speciesReference species="S82"/>
    </listOfReactants>
    <listOfProducts>
      <speciesReference species="S73"/>
      <speciesReference species="S82"/>
    </listOfProducts>
    <kineticLaw>
      <math xmlns="http://www.w3.org/1998/Math/MathML">
        <apply>
          <times/>
          <ci> kactPI3KTie2 </ci>
          <ci> S13 </ci>
```

```
          <ci> S82 </ci>
        </apply>
      </math>
    </kineticLaw>
  </reaction>
  <reaction id="R187" reversible="false">
    <listOfReactants>
      <speciesReference species="S13"/>
      <speciesReference species="S83"/>
    </listOfReactants>
    <listOfProducts>
      <speciesReference species="S73"/>
      <speciesReference species="S83"/>
    </listOfProducts>
    <kineticLaw>
      <math xmlns="http://www.w3.org/1998/Math/MathML">
        <apply>
          <times/>
          <ci> kactPI3KTie2 </ci>
          <ci> S13 </ci>
          <ci> S83 </ci>
        </apply>
      </math>
    </kineticLaw>
  </reaction>
  <reaction id="R188" reversible="false">
    <listOfReactants>
      <speciesReference species="S11"/>
      <speciesReference species="S84"/>
    </listOfReactants>
    <listOfProducts>
      <speciesReference species="S11"/>
      <speciesReference species="S12"/>
    </listOfProducts>
    <kineticLaw>
      <math xmlns="http://www.w3.org/1998/Math/MathML">
        <apply>
          <times/>
          <ci> _rateLaw2 </ci>
          <ci> S11 </ci>
          <ci> S84 </ci>
        </apply>
      </math>
    </kineticLaw>
  </reaction>
  <reaction id="R189" reversible="false">
    <listOfReactants>
      <speciesReference species="S15"/>
      <speciesReference species="S84"/>
    </listOfReactants>
    <listOfProducts>
      <speciesReference species="S96"/>
    </listOfProducts>
    <kineticLaw>
      <math xmlns="http://www.w3.org/1998/Math/MathML">
        <apply>
          <times/>
```

```
        <ci> konPDK1PIP3 </ci>
        <ci> S15 </ci>
        <ci> S84 </ci>
      </apply>
    </math>
  </kineticLaw>
</reaction>
<reaction id="R190" reversible="false">
  <listOfReactants>
    <speciesReference species="S14"/>
    <speciesReference species="S84"/>
  </listOfReactants>
  <listOfProducts>
    <speciesReference species="S97"/>
  </listOfProducts>
  <kineticLaw>
    <math xmlns="http://www.w3.org/1998/Math/MathML">
      <apply>
        <times/>
        <ci> konAKTPIP3 </ci>
        <ci> S14 </ci>
        <ci> S84 </ci>
      </apply>
    </math>
  </kineticLaw>
</reaction>
<reaction id="R191" reversible="false">
  <listOfReactants>
    <speciesReference species="S16"/>
    <speciesReference species="S76"/>
  </listOfReactants>
  <listOfProducts>
    <speciesReference species="S74"/>
    <speciesReference species="S76"/>
  </listOfProducts>
  <kineticLaw>
    <math xmlns="http://www.w3.org/1998/Math/MathML">
      <apply>
        <times/>
        <ci> kprhoa </ci>
        <ci> S16 </ci>
        <ci> S76 </ci>
      </apply>
    </math>
  </kineticLaw>
</reaction>
<reaction id="R192" reversible="false">
  <listOfReactants>
    <speciesReference species="S16"/>
    <speciesReference species="S77"/>
  </listOfReactants>
  <listOfProducts>
    <speciesReference species="S74"/>
    <speciesReference species="S77"/>
  </listOfProducts>
  <kineticLaw>
    <math xmlns="http://www.w3.org/1998/Math/MathML">
```

```
        <apply>
          <times/>
          <ci> kprhoa </ci>
          <ci> S16 </ci>
          <ci> S77 </ci>
        </apply>
      </math>
    </kineticLaw>
  </reaction>
  <reaction id="R193" reversible="false">
    <listOfReactants>
      <speciesReference species="S16"/>
      <speciesReference species="S78"/>
    </listOfReactants>
    <listOfProducts>
      <speciesReference species="S74"/>
      <speciesReference species="S78"/>
    </listOfProducts>
    <kineticLaw>
      <math xmlns="http://www.w3.org/1998/Math/MathML">
        <apply>
          <times/>
          <ci> kprhoa </ci>
          <ci> S16 </ci>
          <ci> S78 </ci>
        </apply>
      </math>
    </kineticLaw>
  </reaction>
  <reaction id="R194" reversible="false">
    <listOfReactants>
      <speciesReference species="S16"/>
      <speciesReference species="S79"/>
    </listOfReactants>
    <listOfProducts>
      <speciesReference species="S74"/>
      <speciesReference species="S79"/>
    </listOfProducts>
    <kineticLaw>
      <math xmlns="http://www.w3.org/1998/Math/MathML">
        <apply>
          <times/>
          <ci> kprhoa </ci>
          <ci> S16 </ci>
          <ci> S79 </ci>
        </apply>
      </math>
    </kineticLaw>
  </reaction>
  <reaction id="R195" reversible="false">
    <listOfReactants>
      <speciesReference species="S16"/>
      <speciesReference species="S80"/>
    </listOfReactants>
    <listOfProducts>
      <speciesReference species="S74"/>
      <speciesReference species="S80"/>
```

```xml
    </listOfProducts>
    <kineticLaw>
      <math xmlns="http://www.w3.org/1998/Math/MathML">
        <apply>
          <times/>
          <ci> kprhoa </ci>
          <ci> S16 </ci>
          <ci> S80 </ci>
        </apply>
      </math>
    </kineticLaw>
  </reaction>
  <reaction id="R196" reversible="false">
    <listOfReactants>
      <speciesReference species="S16"/>
      <speciesReference species="S81"/>
    </listOfReactants>
    <listOfProducts>
      <speciesReference species="S74"/>
      <speciesReference species="S81"/>
    </listOfProducts>
    <kineticLaw>
      <math xmlns="http://www.w3.org/1998/Math/MathML">
        <apply>
          <times/>
          <ci> kprhoa </ci>
          <ci> S16 </ci>
          <ci> S81 </ci>
        </apply>
      </math>
    </kineticLaw>
  </reaction>
  <reaction id="R197" reversible="false">
    <listOfReactants>
      <speciesReference species="S16"/>
      <speciesReference species="S82"/>
    </listOfReactants>
    <listOfProducts>
      <speciesReference species="S74"/>
      <speciesReference species="S82"/>
    </listOfProducts>
    <kineticLaw>
      <math xmlns="http://www.w3.org/1998/Math/MathML">
        <apply>
          <times/>
          <ci> kprhoa </ci>
          <ci> S16 </ci>
          <ci> S82 </ci>
        </apply>
      </math>
    </kineticLaw>
  </reaction>
  <reaction id="R198" reversible="false">
    <listOfReactants>
      <speciesReference species="S16"/>
      <speciesReference species="S83"/>
    </listOfReactants>
```

```
    <listOfProducts>
      <speciesReference species="S74"/>
      <speciesReference species="S83"/>
    </listOfProducts>
    <kineticLaw>
      <math xmlns="http://www.w3.org/1998/Math/MathML">
        <apply>
          <times/>
          <ci> kprhoa </ci>
          <ci> S16 </ci>
          <ci> S83 </ci>
        </apply>
      </math>
    </kineticLaw>
  </reaction>
  <reaction id="R199" reversible="false">
    <listOfReactants>
      <speciesReference species="S85"/>
    </listOfReactants>
    <listOfProducts>
      <speciesReference species="S17"/>
      <speciesReference species="S74"/>
    </listOfProducts>
    <kineticLaw>
      <math xmlns="http://www.w3.org/1998/Math/MathML">
        <apply>
          <times/>
          <ci> koffrhoamdia </ci>
          <ci> S85 </ci>
        </apply>
      </math>
    </kineticLaw>
  </reaction>
  <reaction id="R200" reversible="false">
    <listOfReactants>
      <speciesReference species="S18"/>
      <speciesReference species="S85"/>
    </listOfReactants>
    <listOfProducts>
      <speciesReference species="S98"/>
    </listOfProducts>
    <kineticLaw>
      <math xmlns="http://www.w3.org/1998/Math/MathML">
        <apply>
          <times/>
          <ci> konmdiasrc </ci>
          <ci> S18 </ci>
          <ci> S85 </ci>
        </apply>
      </math>
    </kineticLaw>
  </reaction>
  <reaction id="R201" reversible="false">
    <listOfReactants>
      <speciesReference species="S20"/>
      <speciesReference species="S76"/>
    </listOfReactants>
```

```
    <listOfProducts>
      <speciesReference species="S75"/>
      <speciesReference species="S76"/>
    </listOfProducts>
    <kineticLaw>
      <math xmlns="http://www.w3.org/1998/Math/MathML">
        <apply>
          <times/>
          <ci> kactabin2 </ci>
          <ci> S20 </ci>
          <ci> S76 </ci>
        </apply>
      </math>
    </kineticLaw>
  </reaction>
  <reaction id="R202" reversible="false">
    <listOfReactants>
      <speciesReference species="S20"/>
      <speciesReference species="S77"/>
    </listOfReactants>
    <listOfProducts>
      <speciesReference species="S75"/>
      <speciesReference species="S77"/>
    </listOfProducts>
    <kineticLaw>
      <math xmlns="http://www.w3.org/1998/Math/MathML">
        <apply>
          <times/>
          <ci> kactabin2 </ci>
          <ci> S20 </ci>
          <ci> S77 </ci>
        </apply>
      </math>
    </kineticLaw>
  </reaction>
  <reaction id="R203" reversible="false">
    <listOfReactants>
      <speciesReference species="S20"/>
      <speciesReference species="S78"/>
    </listOfReactants>
    <listOfProducts>
      <speciesReference species="S75"/>
      <speciesReference species="S78"/>
    </listOfProducts>
    <kineticLaw>
      <math xmlns="http://www.w3.org/1998/Math/MathML">
        <apply>
          <times/>
          <ci> kactabin2 </ci>
          <ci> S20 </ci>
          <ci> S78 </ci>
        </apply>
      </math>
    </kineticLaw>
  </reaction>
  <reaction id="R204" reversible="false">
    <listOfReactants>
```

```
      <speciesReference species="S20"/>
      <speciesReference species="S79"/>
    </listOfReactants>
    <listOfProducts>
      <speciesReference species="S75"/>
      <speciesReference species="S79"/>
    </listOfProducts>
    <kineticLaw>
      <math xmlns="http://www.w3.org/1998/Math/MathML">
        <apply>
          <times/>
          <ci> kactabin2 </ci>
          <ci> S20 </ci>
          <ci> S79 </ci>
        </apply>
      </math>
    </kineticLaw>
  </reaction>
  <reaction id="R205" reversible="false">
    <listOfReactants>
      <speciesReference species="S20"/>
      <speciesReference species="S80"/>
    </listOfReactants>
    <listOfProducts>
      <speciesReference species="S75"/>
      <speciesReference species="S80"/>
    </listOfProducts>
    <kineticLaw>
      <math xmlns="http://www.w3.org/1998/Math/MathML">
        <apply>
          <times/>
          <ci> kactabin2 </ci>
          <ci> S20 </ci>
          <ci> S80 </ci>
        </apply>
      </math>
    </kineticLaw>
  </reaction>
  <reaction id="R206" reversible="false">
    <listOfReactants>
      <speciesReference species="S20"/>
      <speciesReference species="S81"/>
    </listOfReactants>
    <listOfProducts>
      <speciesReference species="S75"/>
      <speciesReference species="S81"/>
    </listOfProducts>
    <kineticLaw>
      <math xmlns="http://www.w3.org/1998/Math/MathML">
        <apply>
          <times/>
          <ci> kactabin2 </ci>
          <ci> S20 </ci>
          <ci> S81 </ci>
        </apply>
      </math>
    </kineticLaw>
```

```
&lt;/reaction&gt;
&lt;reaction id="R207" reversible="false"&gt;
  &lt;listOfReactants&gt;
    &lt;speciesReference species="S20"/&gt;
    &lt;speciesReference species="S82"/&gt;
  &lt;/listOfReactants&gt;
  &lt;listOfProducts&gt;
    &lt;speciesReference species="S75"/&gt;
    &lt;speciesReference species="S82"/&gt;
  &lt;/listOfProducts&gt;
  &lt;kineticLaw&gt;
    &lt;math xmlns="http://www.w3.org/1998/Math/MathML"&gt;
      &lt;apply&gt;
        &lt;times/&gt;
        &lt;ci&gt; kactabin2 &lt;/ci&gt;
        &lt;ci&gt; S20 &lt;/ci&gt;
        &lt;ci&gt; S82 &lt;/ci&gt;
      &lt;/apply&gt;
    &lt;/math&gt;
  &lt;/kineticLaw&gt;
&lt;/reaction&gt;
&lt;reaction id="R208" reversible="false"&gt;
  &lt;listOfReactants&gt;
    &lt;speciesReference species="S20"/&gt;
    &lt;speciesReference species="S83"/&gt;
  &lt;/listOfReactants&gt;
  &lt;listOfProducts&gt;
    &lt;speciesReference species="S75"/&gt;
    &lt;speciesReference species="S83"/&gt;
  &lt;/listOfProducts&gt;
  &lt;kineticLaw&gt;
    &lt;math xmlns="http://www.w3.org/1998/Math/MathML"&gt;
      &lt;apply&gt;
        &lt;times/&gt;
        &lt;ci&gt; kactabin2 &lt;/ci&gt;
        &lt;ci&gt; S20 &lt;/ci&gt;
        &lt;ci&gt; S83 &lt;/ci&gt;
      &lt;/apply&gt;
    &lt;/math&gt;
  &lt;/kineticLaw&gt;
&lt;/reaction&gt;
&lt;reaction id="R209" reversible="false"&gt;
  &lt;listOfReactants&gt;
    &lt;speciesReference species="S6"/&gt;
    &lt;speciesReference species="S86"/&gt;
  &lt;/listOfReactants&gt;
  &lt;listOfProducts&gt;
    &lt;speciesReference species="S99"/&gt;
  &lt;/listOfProducts&gt;
  &lt;kineticLaw&gt;
    &lt;math xmlns="http://www.w3.org/1998/Math/MathML"&gt;
      &lt;apply&gt;
        &lt;times/&gt;
        &lt;cn&gt; 2 &lt;/cn&gt;
        &lt;ci&gt; konveptp &lt;/ci&gt;
        &lt;ci&gt; S6 &lt;/ci&gt;
        &lt;ci&gt; S86 &lt;/ci&gt;
```

```
      </apply>
    </math>
  </kineticLaw>
</reaction>
<reaction id="R210" reversible="false">
  <listOfReactants>
    <speciesReference species="S6"/>
    <speciesReference species="S87"/>
  </listOfReactants>
  <listOfProducts>
    <speciesReference species="S100"/>
  </listOfProducts>
  <kineticLaw>
    <math xmlns="http://www.w3.org/1998/Math/MathML">
      <apply>
        <times/>
        <cn> 2 </cn>
        <ci> konveptp </ci>
        <ci> S6 </ci>
        <ci> S87 </ci>
      </apply>
    </math>
  </kineticLaw>
</reaction>
<reaction id="R211" reversible="false">
  <listOfReactants>
    <speciesReference species="S6"/>
    <speciesReference species="S88"/>
  </listOfReactants>
  <listOfProducts>
    <speciesReference species="S101"/>
  </listOfProducts>
  <kineticLaw>
    <math xmlns="http://www.w3.org/1998/Math/MathML">
      <apply>
        <times/>
        <ci> konveptp </ci>
        <ci> S6 </ci>
        <ci> S88 </ci>
      </apply>
    </math>
  </kineticLaw>
</reaction>
<reaction id="R212" reversible="false">
  <listOfReactants>
    <speciesReference species="S6"/>
    <speciesReference species="S89"/>
  </listOfReactants>
  <listOfProducts>
    <speciesReference species="S102"/>
  </listOfProducts>
  <kineticLaw>
    <math xmlns="http://www.w3.org/1998/Math/MathML">
      <apply>
        <times/>
        <ci> konveptp </ci>
        <ci> S6 </ci>
```

```
        <ci> S89 </ci>
      </apply>
    </math>
  </kineticLaw>
</reaction>
<reaction id="R213" reversible="false">
  <listOfReactants>
    <speciesReference species="S6"/>
    <speciesReference species="S90"/>
  </listOfReactants>
  <listOfProducts>
    <speciesReference species="S103"/>
  </listOfProducts>
  <kineticLaw>
    <math xmlns="http://www.w3.org/1998/Math/MathML">
      <apply>
        <times/>
        <ci> konveptp </ci>
        <ci> S6 </ci>
        <ci> S90 </ci>
      </apply>
    </math>
  </kineticLaw>
</reaction>
<reaction id="R214" reversible="false">
  <listOfReactants>
    <speciesReference species="S6"/>
    <speciesReference species="S91"/>
  </listOfReactants>
  <listOfProducts>
    <speciesReference species="S104"/>
  </listOfProducts>
  <kineticLaw>
    <math xmlns="http://www.w3.org/1998/Math/MathML">
      <apply>
        <times/>
        <ci> konveptp </ci>
        <ci> S6 </ci>
        <ci> S91 </ci>
      </apply>
    </math>
  </kineticLaw>
</reaction>
<reaction id="R215" reversible="false">
  <listOfReactants>
    <speciesReference species="S6"/>
    <speciesReference species="S92"/>
  </listOfReactants>
  <listOfProducts>
    <speciesReference species="S105"/>
  </listOfProducts>
  <kineticLaw>
    <math xmlns="http://www.w3.org/1998/Math/MathML">
      <apply>
        <times/>
        <cn> 2 </cn>
        <ci> konveptp </ci>
```

```
        <ci> S6 </ci>
        <ci> S92 </ci>
      </apply>
    </math>
  </kineticLaw>
</reaction>
<reaction id="R216" reversible="false">
  <listOfReactants>
    <speciesReference species="S6"/>
    <speciesReference species="S93"/>
  </listOfReactants>
  <listOfProducts>
    <speciesReference species="S106"/>
  </listOfProducts>
  <kineticLaw>
    <math xmlns="http://www.w3.org/1998/Math/MathML">
      <apply>
        <times/>
        <cn> 2 </cn>
        <ci> konveptp </ci>
        <ci> S6 </ci>
        <ci> S93 </ci>
      </apply>
    </math>
  </kineticLaw>
</reaction>
<reaction id="R217" reversible="false">
  <listOfReactants>
    <speciesReference species="S6"/>
    <speciesReference species="S94"/>
  </listOfReactants>
  <listOfProducts>
    <speciesReference species="S107"/>
  </listOfProducts>
  <kineticLaw>
    <math xmlns="http://www.w3.org/1998/Math/MathML">
      <apply>
        <times/>
        <cn> 2 </cn>
        <ci> konveptp </ci>
        <ci> S6 </ci>
        <ci> S94 </ci>
      </apply>
    </math>
  </kineticLaw>
</reaction>
<reaction id="R218" reversible="false">
  <listOfReactants>
    <speciesReference species="S6"/>
    <speciesReference species="S95"/>
  </listOfReactants>
  <listOfProducts>
    <speciesReference species="S108"/>
  </listOfProducts>
  <kineticLaw>
    <math xmlns="http://www.w3.org/1998/Math/MathML">
      <apply>
```

```
        <times/>
        <cn> 2 </cn>
        <ci> konveptp </ci>
        <ci> S6 </ci>
        <ci> S95 </ci>
      </apply>
    </math>
  </kineticLaw>
</reaction>
<reaction id="R219" reversible="false">
  <listOfReactants>
    <speciesReference species="S86"/>
  </listOfReactants>
  <listOfProducts>
    <speciesReference species="S6"/>
    <speciesReference species="S76"/>
  </listOfProducts>
  <kineticLaw>
    <math xmlns="http://www.w3.org/1998/Math/MathML">
      <apply>
        <times/>
        <ci> koffveptp </ci>
        <ci> S86 </ci>
      </apply>
    </math>
  </kineticLaw>
</reaction>
<reaction id="R220" reversible="false">
  <listOfReactants>
    <speciesReference species="S87"/>
  </listOfReactants>
  <listOfProducts>
    <speciesReference species="S6"/>
    <speciesReference species="S77"/>
  </listOfProducts>
  <kineticLaw>
    <math xmlns="http://www.w3.org/1998/Math/MathML">
      <apply>
        <times/>
        <ci> koffveptp </ci>
        <ci> S87 </ci>
      </apply>
    </math>
  </kineticLaw>
</reaction>
<reaction id="R221" reversible="false">
  <listOfReactants>
    <speciesReference species="S88"/>
  </listOfReactants>
  <listOfProducts>
    <speciesReference species="S6"/>
    <speciesReference species="S78"/>
  </listOfProducts>
  <kineticLaw>
    <math xmlns="http://www.w3.org/1998/Math/MathML">
      <apply>
        <times/>
```

```
        <cn> 2 </cn>
        <ci> koffveptp </ci>
        <ci> S88 </ci>
      </apply>
    </math>
  </kineticLaw>
</reaction>
<reaction id="R222" reversible="false">
  <listOfReactants>
    <speciesReference species="S89"/>
  </listOfReactants>
  <listOfProducts>
    <speciesReference species="S6"/>
    <speciesReference species="S79"/>
  </listOfProducts>
  <kineticLaw>
    <math xmlns="http://www.w3.org/1998/Math/MathML">
      <apply>
        <times/>
        <cn> 2 </cn>
        <ci> koffveptp </ci>
        <ci> S89 </ci>
      </apply>
    </math>
  </kineticLaw>
</reaction>
<reaction id="R223" reversible="false">
  <listOfReactants>
    <speciesReference species="S90"/>
  </listOfReactants>
  <listOfProducts>
    <speciesReference species="S6"/>
    <speciesReference species="S80"/>
  </listOfProducts>
  <kineticLaw>
    <math xmlns="http://www.w3.org/1998/Math/MathML">
      <apply>
        <times/>
        <cn> 3 </cn>
        <ci> koffveptp </ci>
        <ci> S90 </ci>
      </apply>
    </math>
  </kineticLaw>
</reaction>
<reaction id="R224" reversible="false">
  <listOfReactants>
    <speciesReference species="S91"/>
  </listOfReactants>
  <listOfProducts>
    <speciesReference species="S6"/>
    <speciesReference species="S81"/>
  </listOfProducts>
  <kineticLaw>
    <math xmlns="http://www.w3.org/1998/Math/MathML">
      <apply>
        <times/>
```

```xml
          <cn> 3 </cn>
          <ci> koffveptp </ci>
          <ci> S91 </ci>
        </apply>
      </math>
    </kineticLaw>
  </reaction>
  <reaction id="R225" reversible="false">
    <listOfReactants>
      <speciesReference species="S92"/>
    </listOfReactants>
    <listOfProducts>
      <speciesReference species="S6"/>
      <speciesReference species="S82"/>
    </listOfProducts>
    <kineticLaw>
      <math xmlns="http://www.w3.org/1998/Math/MathML">
        <apply>
          <times/>
          <ci> koffveptp </ci>
          <ci> S92 </ci>
        </apply>
      </math>
    </kineticLaw>
  </reaction>
  <reaction id="R226" reversible="false">
    <listOfReactants>
      <speciesReference species="S93"/>
    </listOfReactants>
    <listOfProducts>
      <speciesReference species="S6"/>
      <speciesReference species="S83"/>
    </listOfProducts>
    <kineticLaw>
      <math xmlns="http://www.w3.org/1998/Math/MathML">
        <apply>
          <times/>
          <ci> koffveptp </ci>
          <ci> S93 </ci>
        </apply>
      </math>
    </kineticLaw>
  </reaction>
  <reaction id="R227" reversible="false">
    <listOfReactants>
      <speciesReference species="S86"/>
    </listOfReactants>
    <listOfProducts>
      <speciesReference species="S6"/>
      <speciesReference species="S109"/>
    </listOfProducts>
    <kineticLaw>
      <math xmlns="http://www.w3.org/1998/Math/MathML">
        <apply>
          <times/>
          <ci> kactveptp_ang1 </ci>
          <ci> S86 </ci>
```

```xml
        </apply>
      </math>
    </kineticLaw>
  </reaction>
  <reaction id="R228" reversible="false">
    <listOfReactants>
      <speciesReference species="S88"/>
    </listOfReactants>
    <listOfProducts>
      <speciesReference species="S6"/>
      <speciesReference species="S107"/>
    </listOfProducts>
    <kineticLaw>
      <math xmlns="http://www.w3.org/1998/Math/MathML">
        <apply>
          <times/>
          <cn> 2 </cn>
          <ci> kactveptp_ang1 </ci>
          <ci> S88 </ci>
        </apply>
      </math>
    </kineticLaw>
  </reaction>
  <reaction id="R229" reversible="false">
    <listOfReactants>
      <speciesReference species="S90"/>
    </listOfReactants>
    <listOfProducts>
      <speciesReference species="S6"/>
      <speciesReference species="S105"/>
    </listOfProducts>
    <kineticLaw>
      <math xmlns="http://www.w3.org/1998/Math/MathML">
        <apply>
          <times/>
          <cn> 3 </cn>
          <ci> kactveptp_ang1 </ci>
          <ci> S90 </ci>
        </apply>
      </math>
    </kineticLaw>
  </reaction>
  <reaction id="R230" reversible="false">
    <listOfReactants>
      <speciesReference species="S92"/>
    </listOfReactants>
    <listOfProducts>
      <speciesReference species="S6"/>
      <speciesReference species="S110"/>
    </listOfProducts>
    <kineticLaw>
      <math xmlns="http://www.w3.org/1998/Math/MathML">
        <apply>
          <times/>
          <ci> kactveptp_ang1 </ci>
          <ci> S92 </ci>
        </apply>
```

```
    </math>
  </kineticLaw>
</reaction>
<reaction id="R231" reversible="false">
  <listOfReactants>
    <speciesReference species="S87"/>
  </listOfReactants>
  <listOfProducts>
    <speciesReference species="S6"/>
    <speciesReference species="S111"/>
  </listOfProducts>
  <kineticLaw>
    <math xmlns="http://www.w3.org/1998/Math/MathML">
      <apply>
        <times/>
        <ci> kactveptp_ang2 </ci>
        <ci> S87 </ci>
      </apply>
    </math>
  </kineticLaw>
</reaction>
<reaction id="R232" reversible="false">
  <listOfReactants>
    <speciesReference species="S89"/>
  </listOfReactants>
  <listOfProducts>
    <speciesReference species="S6"/>
    <speciesReference species="S108"/>
  </listOfProducts>
  <kineticLaw>
    <math xmlns="http://www.w3.org/1998/Math/MathML">
      <apply>
        <times/>
        <cn> 2 </cn>
        <ci> kactveptp_ang2 </ci>
        <ci> S89 </ci>
      </apply>
    </math>
  </kineticLaw>
</reaction>
<reaction id="R233" reversible="false">
  <listOfReactants>
    <speciesReference species="S91"/>
  </listOfReactants>
  <listOfProducts>
    <speciesReference species="S6"/>
    <speciesReference species="S106"/>
  </listOfProducts>
  <kineticLaw>
    <math xmlns="http://www.w3.org/1998/Math/MathML">
      <apply>
        <times/>
        <cn> 3 </cn>
        <ci> kactveptp_ang2 </ci>
        <ci> S91 </ci>
      </apply>
    </math>
```

```
  </kineticLaw>
</reaction>
<reaction id="R234" reversible="false">
  <listOfReactants>
    <speciesReference species="S93"/>
  </listOfReactants>
  <listOfProducts>
    <speciesReference species="S6"/>
    <speciesReference species="S112"/>
  </listOfProducts>
  <kineticLaw>
    <math xmlns="http://www.w3.org/1998/Math/MathML">
      <apply>
        <times/>
        <ci> kactveptp_ang2 </ci>
        <ci> S93 </ci>
      </apply>
    </math>
  </kineticLaw>
</reaction>
<reaction id="R235" reversible="false">
  <listOfReactants>
    <speciesReference species="S13"/>
    <speciesReference species="S86"/>
  </listOfReactants>
  <listOfProducts>
    <speciesReference species="S73"/>
    <speciesReference species="S86"/>
  </listOfProducts>
  <kineticLaw>
    <math xmlns="http://www.w3.org/1998/Math/MathML">
      <apply>
        <times/>
        <ci> kactPI3KTie2 </ci>
        <ci> S13 </ci>
        <ci> S86 </ci>
      </apply>
    </math>
  </kineticLaw>
</reaction>
<reaction id="R236" reversible="false">
  <listOfReactants>
    <speciesReference species="S13"/>
    <speciesReference species="S87"/>
  </listOfReactants>
  <listOfProducts>
    <speciesReference species="S73"/>
    <speciesReference species="S87"/>
  </listOfProducts>
  <kineticLaw>
    <math xmlns="http://www.w3.org/1998/Math/MathML">
      <apply>
        <times/>
        <ci> kactPI3KTie2 </ci>
        <ci> S13 </ci>
        <ci> S87 </ci>
      </apply>
```

```
      </math>
    </kineticLaw>
  </reaction>
  <reaction id="R237" reversible="false">
    <listOfReactants>
      <speciesReference species="S13"/>
      <speciesReference species="S88"/>
    </listOfReactants>
    <listOfProducts>
      <speciesReference species="S73"/>
      <speciesReference species="S88"/>
    </listOfProducts>
    <kineticLaw>
      <math xmlns="http://www.w3.org/1998/Math/MathML">
        <apply>
          <times/>
          <ci> kactPI3KTie2 </ci>
          <ci> S13 </ci>
          <ci> S88 </ci>
        </apply>
      </math>
    </kineticLaw>
  </reaction>
  <reaction id="R238" reversible="false">
    <listOfReactants>
      <speciesReference species="S13"/>
      <speciesReference species="S89"/>
    </listOfReactants>
    <listOfProducts>
      <speciesReference species="S73"/>
      <speciesReference species="S89"/>
    </listOfProducts>
    <kineticLaw>
      <math xmlns="http://www.w3.org/1998/Math/MathML">
        <apply>
          <times/>
          <ci> kactPI3KTie2 </ci>
          <ci> S13 </ci>
          <ci> S89 </ci>
        </apply>
      </math>
    </kineticLaw>
  </reaction>
  <reaction id="R239" reversible="false">
    <listOfReactants>
      <speciesReference species="S13"/>
      <speciesReference species="S90"/>
    </listOfReactants>
    <listOfProducts>
      <speciesReference species="S73"/>
      <speciesReference species="S90"/>
    </listOfProducts>
    <kineticLaw>
      <math xmlns="http://www.w3.org/1998/Math/MathML">
        <apply>
          <times/>
          <ci> kactPI3KTie2 </ci>
```

```
          <ci> S13 </ci>
          <ci> S90 </ci>
        </apply>
      </math>
    </kineticLaw>
  </reaction>
  <reaction id="R240" reversible="false">
    <listOfReactants>
      <speciesReference species="S13"/>
      <speciesReference species="S91"/>
    </listOfReactants>
    <listOfProducts>
      <speciesReference species="S73"/>
      <speciesReference species="S91"/>
    </listOfProducts>
    <kineticLaw>
      <math xmlns="http://www.w3.org/1998/Math/MathML">
        <apply>
          <times/>
          <ci> kactPI3KTie2 </ci>
          <ci> S13 </ci>
          <ci> S91 </ci>
        </apply>
      </math>
    </kineticLaw>
  </reaction>
  <reaction id="R241" reversible="false">
    <listOfReactants>
      <speciesReference species="S13"/>
      <speciesReference species="S92"/>
    </listOfReactants>
    <listOfProducts>
      <speciesReference species="S73"/>
      <speciesReference species="S92"/>
    </listOfProducts>
    <kineticLaw>
      <math xmlns="http://www.w3.org/1998/Math/MathML">
        <apply>
          <times/>
          <ci> kactPI3KTie2 </ci>
          <ci> S13 </ci>
          <ci> S92 </ci>
        </apply>
      </math>
    </kineticLaw>
  </reaction>
  <reaction id="R242" reversible="false">
    <listOfReactants>
      <speciesReference species="S13"/>
      <speciesReference species="S93"/>
    </listOfReactants>
    <listOfProducts>
      <speciesReference species="S73"/>
      <speciesReference species="S93"/>
    </listOfProducts>
    <kineticLaw>
      <math xmlns="http://www.w3.org/1998/Math/MathML">
```

```
        <apply>
          <times/>
          <ci> kactPI3KTie2 </ci>
          <ci> S13 </ci>
          <ci> S93 </ci>
        </apply>
      </math>
    </kineticLaw>
  </reaction>
  <reaction id="R243" reversible="false">
    <listOfReactants>
      <speciesReference species="S13"/>
      <speciesReference species="S94"/>
    </listOfReactants>
    <listOfProducts>
      <speciesReference species="S73"/>
      <speciesReference species="S94"/>
    </listOfProducts>
    <kineticLaw>
      <math xmlns="http://www.w3.org/1998/Math/MathML">
        <apply>
          <times/>
          <ci> kactPI3KTie2 </ci>
          <ci> S13 </ci>
          <ci> S94 </ci>
        </apply>
      </math>
    </kineticLaw>
  </reaction>
  <reaction id="R244" reversible="false">
    <listOfReactants>
      <speciesReference species="S13"/>
      <speciesReference species="S95"/>
    </listOfReactants>
    <listOfProducts>
      <speciesReference species="S73"/>
      <speciesReference species="S95"/>
    </listOfProducts>
    <kineticLaw>
      <math xmlns="http://www.w3.org/1998/Math/MathML">
        <apply>
          <times/>
          <ci> kactPI3KTie2 </ci>
          <ci> S13 </ci>
          <ci> S95 </ci>
        </apply>
      </math>
    </kineticLaw>
  </reaction>
  <reaction id="R245" reversible="false">
    <listOfReactants>
      <speciesReference species="S96"/>
    </listOfReactants>
    <listOfProducts>
      <speciesReference species="S15"/>
      <speciesReference species="S84"/>
    </listOfProducts>
```

```
    <kineticLaw>
      <math xmlns="http://www.w3.org/1998/Math/MathML">
        <apply>
          <times/>
          <ci> koffPDK1PIP3 </ci>
          <ci> S96 </ci>
        </apply>
      </math>
    </kineticLaw>
  </reaction>
  <reaction id="R246" reversible="false">
    <listOfReactants>
      <speciesReference species="S97"/>
    </listOfReactants>
    <listOfProducts>
      <speciesReference species="S14"/>
      <speciesReference species="S84"/>
    </listOfProducts>
    <kineticLaw>
      <math xmlns="http://www.w3.org/1998/Math/MathML">
        <apply>
          <times/>
          <ci> koffAKTPIP3 </ci>
          <ci> S97 </ci>
        </apply>
      </math>
    </kineticLaw>
  </reaction>
  <reaction id="R247" reversible="false">
    <listOfReactants>
      <speciesReference species="S97"/>
    </listOfReactants>
    <listOfProducts>
      <speciesReference species="S113"/>
    </listOfProducts>
    <kineticLaw>
      <math xmlns="http://www.w3.org/1998/Math/MathML">
        <apply>
          <times/>
          <ci> kpmTORAKT </ci>
          <ci> S97 </ci>
        </apply>
      </math>
    </kineticLaw>
  </reaction>
  <reaction id="R248" reversible="false">
    <listOfReactants>
      <speciesReference species="S16"/>
      <speciesReference species="S86"/>
    </listOfReactants>
    <listOfProducts>
      <speciesReference species="S74"/>
      <speciesReference species="S86"/>
    </listOfProducts>
    <kineticLaw>
      <math xmlns="http://www.w3.org/1998/Math/MathML">
        <apply>
```

```
        <times/>
        <ci> kprhoa </ci>
        <ci> S16 </ci>
        <ci> S86 </ci>
      </apply>
    </math>
  </kineticLaw>
</reaction>
<reaction id="R249" reversible="false">
  <listOfReactants>
    <speciesReference species="S16"/>
    <speciesReference species="S87"/>
  </listOfReactants>
  <listOfProducts>
    <speciesReference species="S74"/>
    <speciesReference species="S87"/>
  </listOfProducts>
  <kineticLaw>
    <math xmlns="http://www.w3.org/1998/Math/MathML">
      <apply>
        <times/>
        <ci> kprhoa </ci>
        <ci> S16 </ci>
        <ci> S87 </ci>
      </apply>
    </math>
  </kineticLaw>
</reaction>
<reaction id="R250" reversible="false">
  <listOfReactants>
    <speciesReference species="S16"/>
    <speciesReference species="S88"/>
  </listOfReactants>
  <listOfProducts>
    <speciesReference species="S74"/>
    <speciesReference species="S88"/>
  </listOfProducts>
  <kineticLaw>
    <math xmlns="http://www.w3.org/1998/Math/MathML">
      <apply>
        <times/>
        <ci> kprhoa </ci>
        <ci> S16 </ci>
        <ci> S88 </ci>
      </apply>
    </math>
  </kineticLaw>
</reaction>
<reaction id="R251" reversible="false">
  <listOfReactants>
    <speciesReference species="S16"/>
    <speciesReference species="S89"/>
  </listOfReactants>
  <listOfProducts>
    <speciesReference species="S74"/>
    <speciesReference species="S89"/>
  </listOfProducts>
```

```
    <kineticLaw>
      <math xmlns="http://www.w3.org/1998/Math/MathML">
        <apply>
          <times/>
          <ci> kprhoa </ci>
          <ci> S16 </ci>
          <ci> S89 </ci>
        </apply>
      </math>
    </kineticLaw>
  </reaction>
  <reaction id="R252" reversible="false">
    <listOfReactants>
      <speciesReference species="S16"/>
      <speciesReference species="S90"/>
    </listOfReactants>
    <listOfProducts>
      <speciesReference species="S74"/>
      <speciesReference species="S90"/>
    </listOfProducts>
    <kineticLaw>
      <math xmlns="http://www.w3.org/1998/Math/MathML">
        <apply>
          <times/>
          <ci> kprhoa </ci>
          <ci> S16 </ci>
          <ci> S90 </ci>
        </apply>
      </math>
    </kineticLaw>
  </reaction>
  <reaction id="R253" reversible="false">
    <listOfReactants>
      <speciesReference species="S16"/>
      <speciesReference species="S91"/>
    </listOfReactants>
    <listOfProducts>
      <speciesReference species="S74"/>
      <speciesReference species="S91"/>
    </listOfProducts>
    <kineticLaw>
      <math xmlns="http://www.w3.org/1998/Math/MathML">
        <apply>
          <times/>
          <ci> kprhoa </ci>
          <ci> S16 </ci>
          <ci> S91 </ci>
        </apply>
      </math>
    </kineticLaw>
  </reaction>
  <reaction id="R254" reversible="false">
    <listOfReactants>
      <speciesReference species="S16"/>
      <speciesReference species="S92"/>
    </listOfReactants>
    <listOfProducts>
```

```
      <speciesReference species="S74"/>
      <speciesReference species="S92"/>
    </listOfProducts>
    <kineticLaw>
      <math xmlns="http://www.w3.org/1998/Math/MathML">
        <apply>
          <times/>
          <ci> kprhoa </ci>
          <ci> S16 </ci>
          <ci> S92 </ci>
        </apply>
      </math>
    </kineticLaw>
  </reaction>
  <reaction id="R255" reversible="false">
    <listOfReactants>
      <speciesReference species="S16"/>
      <speciesReference species="S93"/>
    </listOfReactants>
    <listOfProducts>
      <speciesReference species="S74"/>
      <speciesReference species="S93"/>
    </listOfProducts>
    <kineticLaw>
      <math xmlns="http://www.w3.org/1998/Math/MathML">
        <apply>
          <times/>
          <ci> kprhoa </ci>
          <ci> S16 </ci>
          <ci> S93 </ci>
        </apply>
      </math>
    </kineticLaw>
  </reaction>
  <reaction id="R256" reversible="false">
    <listOfReactants>
      <speciesReference species="S16"/>
      <speciesReference species="S94"/>
    </listOfReactants>
    <listOfProducts>
      <speciesReference species="S74"/>
      <speciesReference species="S94"/>
    </listOfProducts>
    <kineticLaw>
      <math xmlns="http://www.w3.org/1998/Math/MathML">
        <apply>
          <times/>
          <ci> kprhoa </ci>
          <ci> S16 </ci>
          <ci> S94 </ci>
        </apply>
      </math>
    </kineticLaw>
  </reaction>
  <reaction id="R257" reversible="false">
    <listOfReactants>
      <speciesReference species="S16"/>
```

```
      <speciesReference species="S95"/>
    </listOfReactants>
    <listOfProducts>
      <speciesReference species="S74"/>
      <speciesReference species="S95"/>
    </listOfProducts>
    <kineticLaw>
      <math xmlns="http://www.w3.org/1998/Math/MathML">
        <apply>
          <times/>
          <ci> kprhoa </ci>
          <ci> S16 </ci>
          <ci> S95 </ci>
        </apply>
      </math>
    </kineticLaw>
  </reaction>
  <reaction id="R258" reversible="false">
    <listOfReactants>
      <speciesReference species="S98"/>
    </listOfReactants>
    <listOfProducts>
      <speciesReference species="S18"/>
      <speciesReference species="S85"/>
    </listOfProducts>
    <kineticLaw>
      <math xmlns="http://www.w3.org/1998/Math/MathML">
        <apply>
          <times/>
          <ci> koffmdiasrc </ci>
          <ci> S98 </ci>
        </apply>
      </math>
    </kineticLaw>
  </reaction>
  <reaction id="R259" reversible="false">
    <listOfReactants>
      <speciesReference species="S20"/>
      <speciesReference species="S86"/>
    </listOfReactants>
    <listOfProducts>
      <speciesReference species="S75"/>
      <speciesReference species="S86"/>
    </listOfProducts>
    <kineticLaw>
      <math xmlns="http://www.w3.org/1998/Math/MathML">
        <apply>
          <times/>
          <ci> kactabin2 </ci>
          <ci> S20 </ci>
          <ci> S86 </ci>
        </apply>
      </math>
    </kineticLaw>
  </reaction>
  <reaction id="R260" reversible="false">
    <listOfReactants>
```

```
      <speciesReference species="S20"/>
      <speciesReference species="S87"/>
    </listOfReactants>
    <listOfProducts>
      <speciesReference species="S75"/>
      <speciesReference species="S87"/>
    </listOfProducts>
    <kineticLaw>
      <math xmlns="http://www.w3.org/1998/Math/MathML">
        <apply>
          <times/>
          <ci> kactabin2 </ci>
          <ci> S20 </ci>
          <ci> S87 </ci>
        </apply>
      </math>
    </kineticLaw>
  </reaction>
  <reaction id="R261" reversible="false">
    <listOfReactants>
      <speciesReference species="S20"/>
      <speciesReference species="S88"/>
    </listOfReactants>
    <listOfProducts>
      <speciesReference species="S75"/>
      <speciesReference species="S88"/>
    </listOfProducts>
    <kineticLaw>
      <math xmlns="http://www.w3.org/1998/Math/MathML">
        <apply>
          <times/>
          <ci> kactabin2 </ci>
          <ci> S20 </ci>
          <ci> S88 </ci>
        </apply>
      </math>
    </kineticLaw>
  </reaction>
  <reaction id="R262" reversible="false">
    <listOfReactants>
      <speciesReference species="S20"/>
      <speciesReference species="S89"/>
    </listOfReactants>
    <listOfProducts>
      <speciesReference species="S75"/>
      <speciesReference species="S89"/>
    </listOfProducts>
    <kineticLaw>
      <math xmlns="http://www.w3.org/1998/Math/MathML">
        <apply>
          <times/>
          <ci> kactabin2 </ci>
          <ci> S20 </ci>
          <ci> S89 </ci>
        </apply>
      </math>
    </kineticLaw>
```

```
&lt;/reaction&gt;
&lt;reaction id="R263" reversible="false"&gt;
  &lt;listOfReactants&gt;
    &lt;speciesReference species="S20"/&gt;
    &lt;speciesReference species="S90"/&gt;
  &lt;/listOfReactants&gt;
  &lt;listOfProducts&gt;
    &lt;speciesReference species="S75"/&gt;
    &lt;speciesReference species="S90"/&gt;
  &lt;/listOfProducts&gt;
  &lt;kineticLaw&gt;
    &lt;math xmlns="http://www.w3.org/1998/Math/MathML"&gt;
      &lt;apply&gt;
        &lt;times/&gt;
        &lt;ci&gt; kactabin2 &lt;/ci&gt;
        &lt;ci&gt; S20 &lt;/ci&gt;
        &lt;ci&gt; S90 &lt;/ci&gt;
      &lt;/apply&gt;
    &lt;/math&gt;
  &lt;/kineticLaw&gt;
&lt;/reaction&gt;
&lt;reaction id="R264" reversible="false"&gt;
  &lt;listOfReactants&gt;
    &lt;speciesReference species="S20"/&gt;
    &lt;speciesReference species="S91"/&gt;
  &lt;/listOfReactants&gt;
  &lt;listOfProducts&gt;
    &lt;speciesReference species="S75"/&gt;
    &lt;speciesReference species="S91"/&gt;
  &lt;/listOfProducts&gt;
  &lt;kineticLaw&gt;
    &lt;math xmlns="http://www.w3.org/1998/Math/MathML"&gt;
      &lt;apply&gt;
        &lt;times/&gt;
        &lt;ci&gt; kactabin2 &lt;/ci&gt;
        &lt;ci&gt; S20 &lt;/ci&gt;
        &lt;ci&gt; S91 &lt;/ci&gt;
      &lt;/apply&gt;
    &lt;/math&gt;
  &lt;/kineticLaw&gt;
&lt;/reaction&gt;
&lt;reaction id="R265" reversible="false"&gt;
  &lt;listOfReactants&gt;
    &lt;speciesReference species="S20"/&gt;
    &lt;speciesReference species="S92"/&gt;
  &lt;/listOfReactants&gt;
  &lt;listOfProducts&gt;
    &lt;speciesReference species="S75"/&gt;
    &lt;speciesReference species="S92"/&gt;
  &lt;/listOfProducts&gt;
  &lt;kineticLaw&gt;
    &lt;math xmlns="http://www.w3.org/1998/Math/MathML"&gt;
      &lt;apply&gt;
        &lt;times/&gt;
        &lt;ci&gt; kactabin2 &lt;/ci&gt;
        &lt;ci&gt; S20 &lt;/ci&gt;
        &lt;ci&gt; S92 &lt;/ci&gt;
```

```
      &lt;/apply&gt;
    &lt;/math&gt;
  &lt;/kineticLaw&gt;
&lt;/reaction&gt;
&lt;reaction id="R266" reversible="false"&gt;
  &lt;listOfReactants&gt;
    &lt;speciesReference species="S20"/&gt;
    &lt;speciesReference species="S93"/&gt;
  &lt;/listOfReactants&gt;
  &lt;listOfProducts&gt;
    &lt;speciesReference species="S75"/&gt;
    &lt;speciesReference species="S93"/&gt;
  &lt;/listOfProducts&gt;
  &lt;kineticLaw&gt;
    &lt;math xmlns="http://www.w3.org/1998/Math/MathML"&gt;
      &lt;apply&gt;
        &lt;times/&gt;
        &lt;ci&gt; kactabin2 &lt;/ci&gt;
        &lt;ci&gt; S20 &lt;/ci&gt;
        &lt;ci&gt; S93 &lt;/ci&gt;
      &lt;/apply&gt;
    &lt;/math&gt;
  &lt;/kineticLaw&gt;
&lt;/reaction&gt;
&lt;reaction id="R267" reversible="false"&gt;
  &lt;listOfReactants&gt;
    &lt;speciesReference species="S20"/&gt;
    &lt;speciesReference species="S94"/&gt;
  &lt;/listOfReactants&gt;
  &lt;listOfProducts&gt;
    &lt;speciesReference species="S75"/&gt;
    &lt;speciesReference species="S94"/&gt;
  &lt;/listOfProducts&gt;
  &lt;kineticLaw&gt;
    &lt;math xmlns="http://www.w3.org/1998/Math/MathML"&gt;
      &lt;apply&gt;
        &lt;times/&gt;
        &lt;ci&gt; kactabin2 &lt;/ci&gt;
        &lt;ci&gt; S20 &lt;/ci&gt;
        &lt;ci&gt; S94 &lt;/ci&gt;
      &lt;/apply&gt;
    &lt;/math&gt;
  &lt;/kineticLaw&gt;
&lt;/reaction&gt;
&lt;reaction id="R268" reversible="false"&gt;
  &lt;listOfReactants&gt;
    &lt;speciesReference species="S20"/&gt;
    &lt;speciesReference species="S95"/&gt;
  &lt;/listOfReactants&gt;
  &lt;listOfProducts&gt;
    &lt;speciesReference species="S75"/&gt;
    &lt;speciesReference species="S95"/&gt;
  &lt;/listOfProducts&gt;
  &lt;kineticLaw&gt;
    &lt;math xmlns="http://www.w3.org/1998/Math/MathML"&gt;
      &lt;apply&gt;
        &lt;times/&gt;
```

```
        <ci> kactabin2 </ci>
        <ci> S20 </ci>
        <ci> S95 </ci>
      </apply>
    </math>
  </kineticLaw>
</reaction>
<reaction id="R269" reversible="false">
  <listOfReactants>
    <speciesReference species="S6"/>
    <speciesReference species="S99"/>
  </listOfReactants>
  <listOfProducts>
    <speciesReference species="S114"/>
  </listOfProducts>
  <kineticLaw>
    <math xmlns="http://www.w3.org/1998/Math/MathML">
      <apply>
        <times/>
        <ci> konveptp </ci>
        <ci> S6 </ci>
        <ci> S99 </ci>
      </apply>
    </math>
  </kineticLaw>
</reaction>
<reaction id="R270" reversible="false">
  <listOfReactants>
    <speciesReference species="S6"/>
    <speciesReference species="S100"/>
  </listOfReactants>
  <listOfProducts>
    <speciesReference species="S115"/>
  </listOfProducts>
  <kineticLaw>
    <math xmlns="http://www.w3.org/1998/Math/MathML">
      <apply>
        <times/>
        <ci> konveptp </ci>
        <ci> S6 </ci>
        <ci> S100 </ci>
      </apply>
    </math>
  </kineticLaw>
</reaction>
<reaction id="R271" reversible="false">
  <listOfReactants>
    <speciesReference species="S6"/>
    <speciesReference species="S105"/>
  </listOfReactants>
  <listOfProducts>
    <speciesReference species="S116"/>
  </listOfProducts>
  <kineticLaw>
    <math xmlns="http://www.w3.org/1998/Math/MathML">
      <apply>
        <times/>
```

```xml
          <ci> konveptp </ci>
          <ci> S6 </ci>
          <ci> S105 </ci>
        </apply>
      </math>
    </kineticLaw>
  </reaction>
  <reaction id="R272" reversible="false">
    <listOfReactants>
      <speciesReference species="S6"/>
      <speciesReference species="S106"/>
    </listOfReactants>
    <listOfProducts>
      <speciesReference species="S117"/>
    </listOfProducts>
    <kineticLaw>
      <math xmlns="http://www.w3.org/1998/Math/MathML">
        <apply>
          <times/>
          <ci> konveptp </ci>
          <ci> S6 </ci>
          <ci> S106 </ci>
        </apply>
      </math>
    </kineticLaw>
  </reaction>
  <reaction id="R273" reversible="false">
    <listOfReactants>
      <speciesReference species="S6"/>
      <speciesReference species="S107"/>
    </listOfReactants>
    <listOfProducts>
      <speciesReference species="S118"/>
    </listOfProducts>
    <kineticLaw>
      <math xmlns="http://www.w3.org/1998/Math/MathML">
        <apply>
          <times/>
          <ci> konveptp </ci>
          <ci> S6 </ci>
          <ci> S107 </ci>
        </apply>
      </math>
    </kineticLaw>
  </reaction>
  <reaction id="R274" reversible="false">
    <listOfReactants>
      <speciesReference species="S6"/>
      <speciesReference species="S108"/>
    </listOfReactants>
    <listOfProducts>
      <speciesReference species="S119"/>
    </listOfProducts>
    <kineticLaw>
      <math xmlns="http://www.w3.org/1998/Math/MathML">
        <apply>
          <times/>
```

```
        <ci> konveptp </ci>
        <ci> S6 </ci>
        <ci> S108 </ci>
      </apply>
    </math>
  </kineticLaw>
</reaction>
<reaction id="R275" reversible="false">
  <listOfReactants>
    <speciesReference species="S6"/>
    <speciesReference species="S109"/>
  </listOfReactants>
  <listOfProducts>
    <speciesReference species="S120"/>
  </listOfProducts>
  <kineticLaw>
    <math xmlns="http://www.w3.org/1998/Math/MathML">
      <apply>
        <times/>
        <cn> 2 </cn>
        <ci> konveptp </ci>
        <ci> S6 </ci>
        <ci> S109 </ci>
      </apply>
    </math>
  </kineticLaw>
</reaction>
<reaction id="R276" reversible="false">
  <listOfReactants>
    <speciesReference species="S6"/>
    <speciesReference species="S110"/>
  </listOfReactants>
  <listOfProducts>
    <speciesReference species="S121"/>
  </listOfProducts>
  <kineticLaw>
    <math xmlns="http://www.w3.org/1998/Math/MathML">
      <apply>
        <times/>
        <cn> 2 </cn>
        <ci> konveptp </ci>
        <ci> S6 </ci>
        <ci> S110 </ci>
      </apply>
    </math>
  </kineticLaw>
</reaction>
<reaction id="R277" reversible="false">
  <listOfReactants>
    <speciesReference species="S6"/>
    <speciesReference species="S111"/>
  </listOfReactants>
  <listOfProducts>
    <speciesReference species="S122"/>
  </listOfProducts>
  <kineticLaw>
    <math xmlns="http://www.w3.org/1998/Math/MathML">
```

```
      <apply>
        <times/>
        <cn> 2 </cn>
        <ci> konveptp </ci>
        <ci> S6 </ci>
        <ci> S111 </ci>
      </apply>
    </math>
  </kineticLaw>
</reaction>
<reaction id="R278" reversible="false">
  <listOfReactants>
    <speciesReference species="S6"/>
    <speciesReference species="S112"/>
  </listOfReactants>
  <listOfProducts>
    <speciesReference species="S123"/>
  </listOfProducts>
  <kineticLaw>
    <math xmlns="http://www.w3.org/1998/Math/MathML">
      <apply>
        <times/>
        <cn> 2 </cn>
        <ci> konveptp </ci>
        <ci> S6 </ci>
        <ci> S112 </ci>
      </apply>
    </math>
  </kineticLaw>
</reaction>
<reaction id="R279" reversible="false">
  <listOfReactants>
    <speciesReference species="S99"/>
  </listOfReactants>
  <listOfProducts>
    <speciesReference species="S6"/>
    <speciesReference species="S86"/>
  </listOfProducts>
  <kineticLaw>
    <math xmlns="http://www.w3.org/1998/Math/MathML">
      <apply>
        <times/>
        <cn> 2 </cn>
        <ci> koffveptp </ci>
        <ci> S99 </ci>
      </apply>
    </math>
  </kineticLaw>
</reaction>
<reaction id="R280" reversible="false">
  <listOfReactants>
    <speciesReference species="S100"/>
  </listOfReactants>
  <listOfProducts>
    <speciesReference species="S6"/>
    <speciesReference species="S87"/>
  </listOfProducts>
```

```
      <kineticLaw>
        <math xmlns="http://www.w3.org/1998/Math/MathML">
          <apply>
            <times/>
            <cn> 2 </cn>
            <ci> koffveptp </ci>
            <ci> S100 </ci>
          </apply>
        </math>
      </kineticLaw>
    </reaction>
    <reaction id="R281" reversible="false">
      <listOfReactants>
        <speciesReference species="S101"/>
      </listOfReactants>
      <listOfProducts>
        <speciesReference species="S6"/>
        <speciesReference species="S88"/>
      </listOfProducts>
      <kineticLaw>
        <math xmlns="http://www.w3.org/1998/Math/MathML">
          <apply>
            <times/>
            <cn> 3 </cn>
            <ci> koffveptp </ci>
            <ci> S101 </ci>
          </apply>
        </math>
      </kineticLaw>
    </reaction>
    <reaction id="R282" reversible="false">
      <listOfReactants>
        <speciesReference species="S102"/>
      </listOfReactants>
      <listOfProducts>
        <speciesReference species="S6"/>
        <speciesReference species="S89"/>
      </listOfProducts>
      <kineticLaw>
        <math xmlns="http://www.w3.org/1998/Math/MathML">
          <apply>
            <times/>
            <cn> 3 </cn>
            <ci> koffveptp </ci>
            <ci> S102 </ci>
          </apply>
        </math>
      </kineticLaw>
    </reaction>
    <reaction id="R283" reversible="false">
      <listOfReactants>
        <speciesReference species="S103"/>
      </listOfReactants>
      <listOfProducts>
        <speciesReference species="S6"/>
        <speciesReference species="S90"/>
      </listOfProducts>
```

```
    <kineticLaw>
      <math xmlns="http://www.w3.org/1998/Math/MathML">
        <apply>
          <times/>
          <cn> 4 </cn>
          <ci> koffveptp </ci>
          <ci> S103 </ci>
        </apply>
      </math>
    </kineticLaw>
  </reaction>
  <reaction id="R284" reversible="false">
    <listOfReactants>
      <speciesReference species="S104"/>
    </listOfReactants>
    <listOfProducts>
      <speciesReference species="S6"/>
      <speciesReference species="S91"/>
    </listOfProducts>
    <kineticLaw>
      <math xmlns="http://www.w3.org/1998/Math/MathML">
        <apply>
          <times/>
          <cn> 4 </cn>
          <ci> koffveptp </ci>
          <ci> S104 </ci>
        </apply>
      </math>
    </kineticLaw>
  </reaction>
  <reaction id="R285" reversible="false">
    <listOfReactants>
      <speciesReference species="S105"/>
    </listOfReactants>
    <listOfProducts>
      <speciesReference species="S6"/>
      <speciesReference species="S92"/>
    </listOfProducts>
    <kineticLaw>
      <math xmlns="http://www.w3.org/1998/Math/MathML">
        <apply>
          <times/>
          <cn> 2 </cn>
          <ci> koffveptp </ci>
          <ci> S105 </ci>
        </apply>
      </math>
    </kineticLaw>
  </reaction>
  <reaction id="R286" reversible="false">
    <listOfReactants>
      <speciesReference species="S106"/>
    </listOfReactants>
    <listOfProducts>
      <speciesReference species="S6"/>
      <speciesReference species="S93"/>
    </listOfProducts>
```

```
      <kineticLaw>
        <math xmlns="http://www.w3.org/1998/Math/MathML">
          <apply>
            <times/>
            <cn> 2 </cn>
            <ci> koffveptp </ci>
            <ci> S106 </ci>
          </apply>
        </math>
      </kineticLaw>
    </reaction>
    <reaction id="R287" reversible="false">
      <listOfReactants>
        <speciesReference species="S107"/>
      </listOfReactants>
      <listOfProducts>
        <speciesReference species="S6"/>
        <speciesReference species="S94"/>
      </listOfProducts>
      <kineticLaw>
        <math xmlns="http://www.w3.org/1998/Math/MathML">
          <apply>
            <times/>
            <ci> koffveptp </ci>
            <ci> S107 </ci>
          </apply>
        </math>
      </kineticLaw>
    </reaction>
    <reaction id="R288" reversible="false">
      <listOfReactants>
        <speciesReference species="S108"/>
      </listOfReactants>
      <listOfProducts>
        <speciesReference species="S6"/>
        <speciesReference species="S95"/>
      </listOfProducts>
      <kineticLaw>
        <math xmlns="http://www.w3.org/1998/Math/MathML">
          <apply>
            <times/>
            <ci> koffveptp </ci>
            <ci> S108 </ci>
          </apply>
        </math>
      </kineticLaw>
    </reaction>
    <reaction id="R289" reversible="false">
      <listOfReactants>
        <speciesReference species="S99"/>
      </listOfReactants>
      <listOfProducts>
        <speciesReference species="S6"/>
        <speciesReference species="S120"/>
      </listOfProducts>
      <kineticLaw>
        <math xmlns="http://www.w3.org/1998/Math/MathML">
```

```
        &lt;apply&gt;
          &lt;times/&gt;
          &lt;cn&gt; 2 &lt;/cn&gt;
          &lt;ci&gt; kactveptp_ang1 &lt;/ci&gt;
          &lt;ci&gt; S99 &lt;/ci&gt;
        &lt;/apply&gt;
      &lt;/math&gt;
    &lt;/kineticLaw&gt;
  &lt;/reaction&gt;
  &lt;reaction id="R290" reversible="false"&gt;
    &lt;listOfReactants&gt;
      &lt;speciesReference species="S101"/&gt;
    &lt;/listOfReactants&gt;
    &lt;listOfProducts&gt;
      &lt;speciesReference species="S6"/&gt;
      &lt;speciesReference species="S118"/&gt;
    &lt;/listOfProducts&gt;
    &lt;kineticLaw&gt;
      &lt;math xmlns="http://www.w3.org/1998/Math/MathML"&gt;
        &lt;apply&gt;
          &lt;times/&gt;
          &lt;cn&gt; 3 &lt;/cn&gt;
          &lt;ci&gt; kactveptp_ang1 &lt;/ci&gt;
          &lt;ci&gt; S101 &lt;/ci&gt;
        &lt;/apply&gt;
      &lt;/math&gt;
    &lt;/kineticLaw&gt;
  &lt;/reaction&gt;
  &lt;reaction id="R291" reversible="false"&gt;
    &lt;listOfReactants&gt;
      &lt;speciesReference species="S103"/&gt;
    &lt;/listOfReactants&gt;
    &lt;listOfProducts&gt;
      &lt;speciesReference species="S6"/&gt;
      &lt;speciesReference species="S116"/&gt;
    &lt;/listOfProducts&gt;
    &lt;kineticLaw&gt;
      &lt;math xmlns="http://www.w3.org/1998/Math/MathML"&gt;
        &lt;apply&gt;
          &lt;times/&gt;
          &lt;cn&gt; 4 &lt;/cn&gt;
          &lt;ci&gt; kactveptp_ang1 &lt;/ci&gt;
          &lt;ci&gt; S103 &lt;/ci&gt;
        &lt;/apply&gt;
      &lt;/math&gt;
    &lt;/kineticLaw&gt;
  &lt;/reaction&gt;
  &lt;reaction id="R292" reversible="false"&gt;
    &lt;listOfReactants&gt;
      &lt;speciesReference species="S105"/&gt;
    &lt;/listOfReactants&gt;
    &lt;listOfProducts&gt;
      &lt;speciesReference species="S6"/&gt;
      &lt;speciesReference species="S121"/&gt;
    &lt;/listOfProducts&gt;
    &lt;kineticLaw&gt;
      &lt;math xmlns="http://www.w3.org/1998/Math/MathML"&gt;
```

```
      <apply>
        <times/>
        <cn> 2 </cn>
        <ci> kactveptp_ang1 </ci>
        <ci> S105 </ci>
      </apply>
    </math>
  </kineticLaw>
</reaction>
<reaction id="R293" reversible="false">
  <listOfReactants>
    <speciesReference species="S107"/>
  </listOfReactants>
  <listOfProducts>
    <speciesReference species="S6"/>
    <speciesReference species="S124"/>
  </listOfProducts>
  <kineticLaw>
    <math xmlns="http://www.w3.org/1998/Math/MathML">
      <apply>
        <times/>
        <ci> kactveptp_ang1 </ci>
        <ci> S107 </ci>
      </apply>
    </math>
  </kineticLaw>
</reaction>
<reaction id="R294" reversible="false">
  <listOfReactants>
    <speciesReference species="S100"/>
  </listOfReactants>
  <listOfProducts>
    <speciesReference species="S6"/>
    <speciesReference species="S122"/>
  </listOfProducts>
  <kineticLaw>
    <math xmlns="http://www.w3.org/1998/Math/MathML">
      <apply>
        <times/>
        <cn> 2 </cn>
        <ci> kactveptp_ang2 </ci>
        <ci> S100 </ci>
      </apply>
    </math>
  </kineticLaw>
</reaction>
<reaction id="R295" reversible="false">
  <listOfReactants>
    <speciesReference species="S102"/>
  </listOfReactants>
  <listOfProducts>
    <speciesReference species="S6"/>
    <speciesReference species="S119"/>
  </listOfProducts>
  <kineticLaw>
    <math xmlns="http://www.w3.org/1998/Math/MathML">
      <apply>
```

```
        &lt;times/&gt;
        &lt;cn&gt; 3 &lt;/cn&gt;
        &lt;ci&gt; kactveptp_ang2 &lt;/ci&gt;
        &lt;ci&gt; S102 &lt;/ci&gt;
      &lt;/apply&gt;
    &lt;/math&gt;
  &lt;/kineticLaw&gt;
&lt;/reaction&gt;
&lt;reaction id="R296" reversible="false"&gt;
  &lt;listOfReactants&gt;
    &lt;speciesReference species="S104"/&gt;
  &lt;/listOfReactants&gt;
  &lt;listOfProducts&gt;
    &lt;speciesReference species="S6"/&gt;
    &lt;speciesReference species="S117"/&gt;
  &lt;/listOfProducts&gt;
  &lt;kineticLaw&gt;
    &lt;math xmlns="http://www.w3.org/1998/Math/MathML"&gt;
      &lt;apply&gt;
        &lt;times/&gt;
        &lt;cn&gt; 4 &lt;/cn&gt;
        &lt;ci&gt; kactveptp_ang2 &lt;/ci&gt;
        &lt;ci&gt; S104 &lt;/ci&gt;
      &lt;/apply&gt;
    &lt;/math&gt;
  &lt;/kineticLaw&gt;
&lt;/reaction&gt;
&lt;reaction id="R297" reversible="false"&gt;
  &lt;listOfReactants&gt;
    &lt;speciesReference species="S106"/&gt;
  &lt;/listOfReactants&gt;
  &lt;listOfProducts&gt;
    &lt;speciesReference species="S6"/&gt;
    &lt;speciesReference species="S123"/&gt;
  &lt;/listOfProducts&gt;
  &lt;kineticLaw&gt;
    &lt;math xmlns="http://www.w3.org/1998/Math/MathML"&gt;
      &lt;apply&gt;
        &lt;times/&gt;
        &lt;cn&gt; 2 &lt;/cn&gt;
        &lt;ci&gt; kactveptp_ang2 &lt;/ci&gt;
        &lt;ci&gt; S106 &lt;/ci&gt;
      &lt;/apply&gt;
    &lt;/math&gt;
  &lt;/kineticLaw&gt;
&lt;/reaction&gt;
&lt;reaction id="R298" reversible="false"&gt;
  &lt;listOfReactants&gt;
    &lt;speciesReference species="S108"/&gt;
  &lt;/listOfReactants&gt;
  &lt;listOfProducts&gt;
    &lt;speciesReference species="S6"/&gt;
    &lt;speciesReference species="S125"/&gt;
  &lt;/listOfProducts&gt;
  &lt;kineticLaw&gt;
    &lt;math xmlns="http://www.w3.org/1998/Math/MathML"&gt;
      &lt;apply&gt;
```

```
        <times/>
        <ci> kactveptp_ang2 </ci>
        <ci> S108 </ci>
      </apply>
    </math>
  </kineticLaw>
</reaction>
<reaction id="R299" reversible="false">
  <listOfReactants>
    <speciesReference species="S13"/>
    <speciesReference species="S99"/>
  </listOfReactants>
  <listOfProducts>
    <speciesReference species="S73"/>
    <speciesReference species="S99"/>
  </listOfProducts>
  <kineticLaw>
    <math xmlns="http://www.w3.org/1998/Math/MathML">
      <apply>
        <times/>
        <ci> kactPI3KTie2 </ci>
        <ci> S13 </ci>
        <ci> S99 </ci>
      </apply>
    </math>
  </kineticLaw>
</reaction>
<reaction id="R300" reversible="false">
  <listOfReactants>
    <speciesReference species="S13"/>
    <speciesReference species="S100"/>
  </listOfReactants>
  <listOfProducts>
    <speciesReference species="S73"/>
    <speciesReference species="S100"/>
  </listOfProducts>
  <kineticLaw>
    <math xmlns="http://www.w3.org/1998/Math/MathML">
      <apply>
        <times/>
        <ci> kactPI3KTie2 </ci>
        <ci> S13 </ci>
        <ci> S100 </ci>
      </apply>
    </math>
  </kineticLaw>
</reaction>
<reaction id="R301" reversible="false">
  <listOfReactants>
    <speciesReference species="S13"/>
    <speciesReference species="S101"/>
  </listOfReactants>
  <listOfProducts>
    <speciesReference species="S73"/>
    <speciesReference species="S101"/>
  </listOfProducts>
  <kineticLaw>
```

```
    <math xmlns="http://www.w3.org/1998/Math/MathML">
      <apply>
        <times/>
        <ci> kactPI3KTie2 </ci>
        <ci> S13 </ci>
        <ci> S101 </ci>
      </apply>
    </math>
  </kineticLaw>
</reaction>
<reaction id="R302" reversible="false">
  <listOfReactants>
    <speciesReference species="S13"/>
    <speciesReference species="S102"/>
  </listOfReactants>
  <listOfProducts>
    <speciesReference species="S73"/>
    <speciesReference species="S102"/>
  </listOfProducts>
  <kineticLaw>
    <math xmlns="http://www.w3.org/1998/Math/MathML">
      <apply>
        <times/>
        <ci> kactPI3KTie2 </ci>
        <ci> S13 </ci>
        <ci> S102 </ci>
      </apply>
    </math>
  </kineticLaw>
</reaction>
<reaction id="R303" reversible="false">
  <listOfReactants>
    <speciesReference species="S13"/>
    <speciesReference species="S103"/>
  </listOfReactants>
  <listOfProducts>
    <speciesReference species="S73"/>
    <speciesReference species="S103"/>
  </listOfProducts>
  <kineticLaw>
    <math xmlns="http://www.w3.org/1998/Math/MathML">
      <apply>
        <times/>
        <ci> kactPI3KTie2 </ci>
        <ci> S13 </ci>
        <ci> S103 </ci>
      </apply>
    </math>
  </kineticLaw>
</reaction>
<reaction id="R304" reversible="false">
  <listOfReactants>
    <speciesReference species="S13"/>
    <speciesReference species="S104"/>
  </listOfReactants>
  <listOfProducts>
    <speciesReference species="S73"/>
```

```
      &lt;speciesReference species="S104"/&gt;
    &lt;/listOfProducts&gt;
    &lt;kineticLaw&gt;
      &lt;math xmlns="http://www.w3.org/1998/Math/MathML"&gt;
        &lt;apply&gt;
          &lt;times/&gt;
          &lt;ci&gt; kactPI3KTie2 &lt;/ci&gt;
          &lt;ci&gt; S13 &lt;/ci&gt;
          &lt;ci&gt; S104 &lt;/ci&gt;
        &lt;/apply&gt;
      &lt;/math&gt;
    &lt;/kineticLaw&gt;
  &lt;/reaction&gt;
  &lt;reaction id="R305" reversible="false"&gt;
    &lt;listOfReactants&gt;
      &lt;speciesReference species="S13"/&gt;
      &lt;speciesReference species="S105"/&gt;
    &lt;/listOfReactants&gt;
    &lt;listOfProducts&gt;
      &lt;speciesReference species="S73"/&gt;
      &lt;speciesReference species="S105"/&gt;
    &lt;/listOfProducts&gt;
    &lt;kineticLaw&gt;
      &lt;math xmlns="http://www.w3.org/1998/Math/MathML"&gt;
        &lt;apply&gt;
          &lt;times/&gt;
          &lt;ci&gt; kactPI3KTie2 &lt;/ci&gt;
          &lt;ci&gt; S13 &lt;/ci&gt;
          &lt;ci&gt; S105 &lt;/ci&gt;
        &lt;/apply&gt;
      &lt;/math&gt;
    &lt;/kineticLaw&gt;
  &lt;/reaction&gt;
  &lt;reaction id="R306" reversible="false"&gt;
    &lt;listOfReactants&gt;
      &lt;speciesReference species="S13"/&gt;
      &lt;speciesReference species="S106"/&gt;
    &lt;/listOfReactants&gt;
    &lt;listOfProducts&gt;
      &lt;speciesReference species="S73"/&gt;
      &lt;speciesReference species="S106"/&gt;
    &lt;/listOfProducts&gt;
    &lt;kineticLaw&gt;
      &lt;math xmlns="http://www.w3.org/1998/Math/MathML"&gt;
        &lt;apply&gt;
          &lt;times/&gt;
          &lt;ci&gt; kactPI3KTie2 &lt;/ci&gt;
          &lt;ci&gt; S13 &lt;/ci&gt;
          &lt;ci&gt; S106 &lt;/ci&gt;
        &lt;/apply&gt;
      &lt;/math&gt;
    &lt;/kineticLaw&gt;
  &lt;/reaction&gt;
  &lt;reaction id="R307" reversible="false"&gt;
    &lt;listOfReactants&gt;
      &lt;speciesReference species="S13"/&gt;
      &lt;speciesReference species="S107"/&gt;
```

```xml
      &lt;/listOfReactants&gt;
      &lt;listOfProducts&gt;
        &lt;speciesReference species="S73"/&gt;
        &lt;speciesReference species="S107"/&gt;
      &lt;/listOfProducts&gt;
      &lt;kineticLaw&gt;
        &lt;math xmlns="http://www.w3.org/1998/Math/MathML"&gt;
          &lt;apply&gt;
            &lt;times/&gt;
            &lt;ci&gt; kactPI3KTie2 &lt;/ci&gt;
            &lt;ci&gt; S13 &lt;/ci&gt;
            &lt;ci&gt; S107 &lt;/ci&gt;
          &lt;/apply&gt;
        &lt;/math&gt;
      &lt;/kineticLaw&gt;
    &lt;/reaction&gt;
    &lt;reaction id="R308" reversible="false"&gt;
      &lt;listOfReactants&gt;
        &lt;speciesReference species="S13"/&gt;
        &lt;speciesReference species="S108"/&gt;
      &lt;/listOfReactants&gt;
      &lt;listOfProducts&gt;
        &lt;speciesReference species="S73"/&gt;
        &lt;speciesReference species="S108"/&gt;
      &lt;/listOfProducts&gt;
      &lt;kineticLaw&gt;
        &lt;math xmlns="http://www.w3.org/1998/Math/MathML"&gt;
          &lt;apply&gt;
            &lt;times/&gt;
            &lt;ci&gt; kactPI3KTie2 &lt;/ci&gt;
            &lt;ci&gt; S13 &lt;/ci&gt;
            &lt;ci&gt; S108 &lt;/ci&gt;
          &lt;/apply&gt;
        &lt;/math&gt;
      &lt;/kineticLaw&gt;
    &lt;/reaction&gt;
    &lt;reaction id="R309" reversible="false"&gt;
      &lt;listOfReactants&gt;
        &lt;speciesReference species="S13"/&gt;
        &lt;speciesReference species="S109"/&gt;
      &lt;/listOfReactants&gt;
      &lt;listOfProducts&gt;
        &lt;speciesReference species="S73"/&gt;
        &lt;speciesReference species="S109"/&gt;
      &lt;/listOfProducts&gt;
      &lt;kineticLaw&gt;
        &lt;math xmlns="http://www.w3.org/1998/Math/MathML"&gt;
          &lt;apply&gt;
            &lt;times/&gt;
            &lt;ci&gt; kactPI3KTie2 &lt;/ci&gt;
            &lt;ci&gt; S13 &lt;/ci&gt;
            &lt;ci&gt; S109 &lt;/ci&gt;
          &lt;/apply&gt;
        &lt;/math&gt;
      &lt;/kineticLaw&gt;
    &lt;/reaction&gt;
    &lt;reaction id="R310" reversible="false"&gt;
```

```
    <listOfReactants>
      <speciesReference species="S13"/>
      <speciesReference species="S110"/>
    </listOfReactants>
    <listOfProducts>
      <speciesReference species="S73"/>
      <speciesReference species="S110"/>
    </listOfProducts>
    <kineticLaw>
      <math xmlns="http://www.w3.org/1998/Math/MathML">
        <apply>
          <times/>
          <ci> kactPI3KTie2 </ci>
          <ci> S13 </ci>
          <ci> S110 </ci>
        </apply>
      </math>
    </kineticLaw>
  </reaction>
  <reaction id="R311" reversible="false">
    <listOfReactants>
      <speciesReference species="S13"/>
      <speciesReference species="S111"/>
    </listOfReactants>
    <listOfProducts>
      <speciesReference species="S73"/>
      <speciesReference species="S111"/>
    </listOfProducts>
    <kineticLaw>
      <math xmlns="http://www.w3.org/1998/Math/MathML">
        <apply>
          <times/>
          <ci> kactPI3KTie2 </ci>
          <ci> S13 </ci>
          <ci> S111 </ci>
        </apply>
      </math>
    </kineticLaw>
  </reaction>
  <reaction id="R312" reversible="false">
    <listOfReactants>
      <speciesReference species="S13"/>
      <speciesReference species="S112"/>
    </listOfReactants>
    <listOfProducts>
      <speciesReference species="S73"/>
      <speciesReference species="S112"/>
    </listOfProducts>
    <kineticLaw>
      <math xmlns="http://www.w3.org/1998/Math/MathML">
        <apply>
          <times/>
          <ci> kactPI3KTie2 </ci>
          <ci> S13 </ci>
          <ci> S112 </ci>
        </apply>
      </math>
```

```
    </kineticLaw>
  </reaction>
  <reaction id="R313" reversible="false">
    <listOfReactants>
      <speciesReference species="S113"/>
    </listOfReactants>
    <listOfProducts>
      <speciesReference species="S84"/>
      <speciesReference species="S126"/>
    </listOfProducts>
    <kineticLaw>
      <math xmlns="http://www.w3.org/1998/Math/MathML">
        <apply>
          <times/>
          <ci> koffAKTPIP3 </ci>
          <ci> S113 </ci>
        </apply>
      </math>
    </kineticLaw>
  </reaction>
  <reaction id="R314" reversible="false">
    <listOfReactants>
      <speciesReference species="S96"/>
      <speciesReference species="S113"/>
    </listOfReactants>
    <listOfProducts>
      <speciesReference species="S96"/>
      <speciesReference species="S127"/>
    </listOfProducts>
    <kineticLaw>
      <math xmlns="http://www.w3.org/1998/Math/MathML">
        <apply>
          <times/>
          <ci> kpAKTPDK1 </ci>
          <ci> S96 </ci>
          <ci> S113 </ci>
        </apply>
      </math>
    </kineticLaw>
  </reaction>
  <reaction id="R315" reversible="false">
    <listOfReactants>
      <speciesReference species="S113"/>
    </listOfReactants>
    <listOfProducts>
      <speciesReference species="S97"/>
    </listOfProducts>
    <kineticLaw>
      <math xmlns="http://www.w3.org/1998/Math/MathML">
        <apply>
          <times/>
          <ci> kdp473AKTPPase </ci>
          <ci> S113 </ci>
        </apply>
      </math>
    </kineticLaw>
  </reaction>
```

```
<reaction id="R316" reversible="false">
  <listOfReactants>
    <speciesReference species="S16"/>
    <speciesReference species="S99"/>
  </listOfReactants>
  <listOfProducts>
    <speciesReference species="S74"/>
    <speciesReference species="S99"/>
  </listOfProducts>
  <kineticLaw>
    <math xmlns="http://www.w3.org/1998/Math/MathML">
      <apply>
        <times/>
        <ci> kprhoa </ci>
        <ci> S16 </ci>
        <ci> S99 </ci>
      </apply>
    </math>
  </kineticLaw>
</reaction>
<reaction id="R317" reversible="false">
  <listOfReactants>
    <speciesReference species="S16"/>
    <speciesReference species="S100"/>
  </listOfReactants>
  <listOfProducts>
    <speciesReference species="S74"/>
    <speciesReference species="S100"/>
  </listOfProducts>
  <kineticLaw>
    <math xmlns="http://www.w3.org/1998/Math/MathML">
      <apply>
        <times/>
        <ci> kprhoa </ci>
        <ci> S16 </ci>
        <ci> S100 </ci>
      </apply>
    </math>
  </kineticLaw>
</reaction>
<reaction id="R318" reversible="false">
  <listOfReactants>
    <speciesReference species="S16"/>
    <speciesReference species="S101"/>
  </listOfReactants>
  <listOfProducts>
    <speciesReference species="S74"/>
    <speciesReference species="S101"/>
  </listOfProducts>
  <kineticLaw>
    <math xmlns="http://www.w3.org/1998/Math/MathML">
      <apply>
        <times/>
        <ci> kprhoa </ci>
        <ci> S16 </ci>
        <ci> S101 </ci>
      </apply>
```

```xml
      </math>
    </kineticLaw>
  </reaction>
  <reaction id="R319" reversible="false">
    <listOfReactants>
      <speciesReference species="S16"/>
      <speciesReference species="S102"/>
    </listOfReactants>
    <listOfProducts>
      <speciesReference species="S74"/>
      <speciesReference species="S102"/>
    </listOfProducts>
    <kineticLaw>
      <math xmlns="http://www.w3.org/1998/Math/MathML">
        <apply>
          <times/>
          <ci> kprhoa </ci>
          <ci> S16 </ci>
          <ci> S102 </ci>
        </apply>
      </math>
    </kineticLaw>
  </reaction>
  <reaction id="R320" reversible="false">
    <listOfReactants>
      <speciesReference species="S16"/>
      <speciesReference species="S103"/>
    </listOfReactants>
    <listOfProducts>
      <speciesReference species="S74"/>
      <speciesReference species="S103"/>
    </listOfProducts>
    <kineticLaw>
      <math xmlns="http://www.w3.org/1998/Math/MathML">
        <apply>
          <times/>
          <ci> kprhoa </ci>
          <ci> S16 </ci>
          <ci> S103 </ci>
        </apply>
      </math>
    </kineticLaw>
  </reaction>
  <reaction id="R321" reversible="false">
    <listOfReactants>
      <speciesReference species="S16"/>
      <speciesReference species="S104"/>
    </listOfReactants>
    <listOfProducts>
      <speciesReference species="S74"/>
      <speciesReference species="S104"/>
    </listOfProducts>
    <kineticLaw>
      <math xmlns="http://www.w3.org/1998/Math/MathML">
        <apply>
          <times/>
          <ci> kprhoa </ci>
```

```
        <ci> S16 </ci>
        <ci> S104 </ci>
      </apply>
    </math>
  </kineticLaw>
</reaction>
<reaction id="R322" reversible="false">
  <listOfReactants>
    <speciesReference species="S16"/>
    <speciesReference species="S105"/>
  </listOfReactants>
  <listOfProducts>
    <speciesReference species="S74"/>
    <speciesReference species="S105"/>
  </listOfProducts>
  <kineticLaw>
    <math xmlns="http://www.w3.org/1998/Math/MathML">
      <apply>
        <times/>
        <ci> kprhoa </ci>
        <ci> S16 </ci>
        <ci> S105 </ci>
      </apply>
    </math>
  </kineticLaw>
</reaction>
<reaction id="R323" reversible="false">
  <listOfReactants>
    <speciesReference species="S16"/>
    <speciesReference species="S106"/>
  </listOfReactants>
  <listOfProducts>
    <speciesReference species="S74"/>
    <speciesReference species="S106"/>
  </listOfProducts>
  <kineticLaw>
    <math xmlns="http://www.w3.org/1998/Math/MathML">
      <apply>
        <times/>
        <ci> kprhoa </ci>
        <ci> S16 </ci>
        <ci> S106 </ci>
      </apply>
    </math>
  </kineticLaw>
</reaction>
<reaction id="R324" reversible="false">
  <listOfReactants>
    <speciesReference species="S16"/>
    <speciesReference species="S107"/>
  </listOfReactants>
  <listOfProducts>
    <speciesReference species="S74"/>
    <speciesReference species="S107"/>
  </listOfProducts>
  <kineticLaw>
    <math xmlns="http://www.w3.org/1998/Math/MathML">
```

```
        &lt;apply&gt;
          &lt;times/&gt;
          &lt;ci&gt; kprhoa &lt;/ci&gt;
          &lt;ci&gt; S16 &lt;/ci&gt;
          &lt;ci&gt; S107 &lt;/ci&gt;
        &lt;/apply&gt;
      &lt;/math&gt;
    &lt;/kineticLaw&gt;
  &lt;/reaction&gt;
  &lt;reaction id="R325" reversible="false"&gt;
    &lt;listOfReactants&gt;
      &lt;speciesReference species="S16"/&gt;
      &lt;speciesReference species="S108"/&gt;
    &lt;/listOfReactants&gt;
    &lt;listOfProducts&gt;
      &lt;speciesReference species="S74"/&gt;
      &lt;speciesReference species="S108"/&gt;
    &lt;/listOfProducts&gt;
    &lt;kineticLaw&gt;
      &lt;math xmlns="http://www.w3.org/1998/Math/MathML"&gt;
        &lt;apply&gt;
          &lt;times/&gt;
          &lt;ci&gt; kprhoa &lt;/ci&gt;
          &lt;ci&gt; S16 &lt;/ci&gt;
          &lt;ci&gt; S108 &lt;/ci&gt;
        &lt;/apply&gt;
      &lt;/math&gt;
    &lt;/kineticLaw&gt;
  &lt;/reaction&gt;
  &lt;reaction id="R326" reversible="false"&gt;
    &lt;listOfReactants&gt;
      &lt;speciesReference species="S16"/&gt;
      &lt;speciesReference species="S109"/&gt;
    &lt;/listOfReactants&gt;
    &lt;listOfProducts&gt;
      &lt;speciesReference species="S74"/&gt;
      &lt;speciesReference species="S109"/&gt;
    &lt;/listOfProducts&gt;
    &lt;kineticLaw&gt;
      &lt;math xmlns="http://www.w3.org/1998/Math/MathML"&gt;
        &lt;apply&gt;
          &lt;times/&gt;
          &lt;ci&gt; kprhoa &lt;/ci&gt;
          &lt;ci&gt; S16 &lt;/ci&gt;
          &lt;ci&gt; S109 &lt;/ci&gt;
        &lt;/apply&gt;
      &lt;/math&gt;
    &lt;/kineticLaw&gt;
  &lt;/reaction&gt;
  &lt;reaction id="R327" reversible="false"&gt;
    &lt;listOfReactants&gt;
      &lt;speciesReference species="S16"/&gt;
      &lt;speciesReference species="S110"/&gt;
    &lt;/listOfReactants&gt;
    &lt;listOfProducts&gt;
      &lt;speciesReference species="S74"/&gt;
      &lt;speciesReference species="S110"/&gt;
```

```xml
    </listOfProducts>
    <kineticLaw>
      <math xmlns="http://www.w3.org/1998/Math/MathML">
        <apply>
          <times/>
          <ci> kprhoa </ci>
          <ci> S16 </ci>
          <ci> S110 </ci>
        </apply>
      </math>
    </kineticLaw>
  </reaction>
  <reaction id="R328" reversible="false">
    <listOfReactants>
      <speciesReference species="S16"/>
      <speciesReference species="S111"/>
    </listOfReactants>
    <listOfProducts>
      <speciesReference species="S74"/>
      <speciesReference species="S111"/>
    </listOfProducts>
    <kineticLaw>
      <math xmlns="http://www.w3.org/1998/Math/MathML">
        <apply>
          <times/>
          <ci> kprhoa </ci>
          <ci> S16 </ci>
          <ci> S111 </ci>
        </apply>
      </math>
    </kineticLaw>
  </reaction>
  <reaction id="R329" reversible="false">
    <listOfReactants>
      <speciesReference species="S16"/>
      <speciesReference species="S112"/>
    </listOfReactants>
    <listOfProducts>
      <speciesReference species="S74"/>
      <speciesReference species="S112"/>
    </listOfProducts>
    <kineticLaw>
      <math xmlns="http://www.w3.org/1998/Math/MathML">
        <apply>
          <times/>
          <ci> kprhoa </ci>
          <ci> S16 </ci>
          <ci> S112 </ci>
        </apply>
      </math>
    </kineticLaw>
  </reaction>
  <reaction id="R330" reversible="false">
    <listOfReactants>
      <speciesReference species="S20"/>
      <speciesReference species="S99"/>
    </listOfReactants>
```

```
      <listOfProducts>
        <speciesReference species="S75"/>
        <speciesReference species="S99"/>
      </listOfProducts>
      <kineticLaw>
        <math xmlns="http://www.w3.org/1998/Math/MathML">
          <apply>
            <times/>
            <ci> kactabin2 </ci>
            <ci> S20 </ci>
            <ci> S99 </ci>
          </apply>
        </math>
      </kineticLaw>
    </reaction>
    <reaction id="R331" reversible="false">
      <listOfReactants>
        <speciesReference species="S20"/>
        <speciesReference species="S100"/>
      </listOfReactants>
      <listOfProducts>
        <speciesReference species="S75"/>
        <speciesReference species="S100"/>
      </listOfProducts>
      <kineticLaw>
        <math xmlns="http://www.w3.org/1998/Math/MathML">
          <apply>
            <times/>
            <ci> kactabin2 </ci>
            <ci> S20 </ci>
            <ci> S100 </ci>
          </apply>
        </math>
      </kineticLaw>
    </reaction>
    <reaction id="R332" reversible="false">
      <listOfReactants>
        <speciesReference species="S20"/>
        <speciesReference species="S101"/>
      </listOfReactants>
      <listOfProducts>
        <speciesReference species="S75"/>
        <speciesReference species="S101"/>
      </listOfProducts>
      <kineticLaw>
        <math xmlns="http://www.w3.org/1998/Math/MathML">
          <apply>
            <times/>
            <ci> kactabin2 </ci>
            <ci> S20 </ci>
            <ci> S101 </ci>
          </apply>
        </math>
      </kineticLaw>
    </reaction>
    <reaction id="R333" reversible="false">
      <listOfReactants>
```

```
    <speciesReference species="S20"/>
    <speciesReference species="S102"/>
  </listOfReactants>
  <listOfProducts>
    <speciesReference species="S75"/>
    <speciesReference species="S102"/>
  </listOfProducts>
  <kineticLaw>
    <math xmlns="http://www.w3.org/1998/Math/MathML">
      <apply>
        <times/>
        <ci> kactabin2 </ci>
        <ci> S20 </ci>
        <ci> S102 </ci>
      </apply>
    </math>
  </kineticLaw>
</reaction>
<reaction id="R334" reversible="false">
  <listOfReactants>
    <speciesReference species="S20"/>
    <speciesReference species="S103"/>
  </listOfReactants>
  <listOfProducts>
    <speciesReference species="S75"/>
    <speciesReference species="S103"/>
  </listOfProducts>
  <kineticLaw>
    <math xmlns="http://www.w3.org/1998/Math/MathML">
      <apply>
        <times/>
        <ci> kactabin2 </ci>
        <ci> S20 </ci>
        <ci> S103 </ci>
      </apply>
    </math>
  </kineticLaw>
</reaction>
<reaction id="R335" reversible="false">
  <listOfReactants>
    <speciesReference species="S20"/>
    <speciesReference species="S104"/>
  </listOfReactants>
  <listOfProducts>
    <speciesReference species="S75"/>
    <speciesReference species="S104"/>
  </listOfProducts>
  <kineticLaw>
    <math xmlns="http://www.w3.org/1998/Math/MathML">
      <apply>
        <times/>
        <ci> kactabin2 </ci>
        <ci> S20 </ci>
        <ci> S104 </ci>
      </apply>
    </math>
  </kineticLaw>
```

```xml
</reaction>
<reaction id="R336" reversible="false">
  <listOfReactants>
    <speciesReference species="S20"/>
    <speciesReference species="S105"/>
  </listOfReactants>
  <listOfProducts>
    <speciesReference species="S75"/>
    <speciesReference species="S105"/>
  </listOfProducts>
  <kineticLaw>
    <math xmlns="http://www.w3.org/1998/Math/MathML">
      <apply>
        <times/>
        <ci> kactabin2 </ci>
        <ci> S20 </ci>
        <ci> S105 </ci>
      </apply>
    </math>
  </kineticLaw>
</reaction>
<reaction id="R337" reversible="false">
  <listOfReactants>
    <speciesReference species="S20"/>
    <speciesReference species="S106"/>
  </listOfReactants>
  <listOfProducts>
    <speciesReference species="S75"/>
    <speciesReference species="S106"/>
  </listOfProducts>
  <kineticLaw>
    <math xmlns="http://www.w3.org/1998/Math/MathML">
      <apply>
        <times/>
        <ci> kactabin2 </ci>
        <ci> S20 </ci>
        <ci> S106 </ci>
      </apply>
    </math>
  </kineticLaw>
</reaction>
<reaction id="R338" reversible="false">
  <listOfReactants>
    <speciesReference species="S20"/>
    <speciesReference species="S107"/>
  </listOfReactants>
  <listOfProducts>
    <speciesReference species="S75"/>
    <speciesReference species="S107"/>
  </listOfProducts>
  <kineticLaw>
    <math xmlns="http://www.w3.org/1998/Math/MathML">
      <apply>
        <times/>
        <ci> kactabin2 </ci>
        <ci> S20 </ci>
        <ci> S107 </ci>
```

```
      </apply>
    </math>
  </kineticLaw>
</reaction>
<reaction id="R339" reversible="false">
  <listOfReactants>
    <speciesReference species="S20"/>
    <speciesReference species="S108"/>
  </listOfReactants>
  <listOfProducts>
    <speciesReference species="S75"/>
    <speciesReference species="S108"/>
  </listOfProducts>
  <kineticLaw>
    <math xmlns="http://www.w3.org/1998/Math/MathML">
      <apply>
        <times/>
        <ci> kactabin2 </ci>
        <ci> S20 </ci>
        <ci> S108 </ci>
      </apply>
    </math>
  </kineticLaw>
</reaction>
<reaction id="R340" reversible="false">
  <listOfReactants>
    <speciesReference species="S20"/>
    <speciesReference species="S109"/>
  </listOfReactants>
  <listOfProducts>
    <speciesReference species="S75"/>
    <speciesReference species="S109"/>
  </listOfProducts>
  <kineticLaw>
    <math xmlns="http://www.w3.org/1998/Math/MathML">
      <apply>
        <times/>
        <ci> kactabin2 </ci>
        <ci> S20 </ci>
        <ci> S109 </ci>
      </apply>
    </math>
  </kineticLaw>
</reaction>
<reaction id="R341" reversible="false">
  <listOfReactants>
    <speciesReference species="S20"/>
    <speciesReference species="S110"/>
  </listOfReactants>
  <listOfProducts>
    <speciesReference species="S75"/>
    <speciesReference species="S110"/>
  </listOfProducts>
  <kineticLaw>
    <math xmlns="http://www.w3.org/1998/Math/MathML">
      <apply>
        <times/>
```

```
          <ci> kactabin2 </ci>
          <ci> S20 </ci>
          <ci> S110 </ci>
        </apply>
      </math>
    </kineticLaw>
  </reaction>
  <reaction id="R342" reversible="false">
    <listOfReactants>
      <speciesReference species="S20"/>
      <speciesReference species="S111"/>
    </listOfReactants>
    <listOfProducts>
      <speciesReference species="S75"/>
      <speciesReference species="S111"/>
    </listOfProducts>
    <kineticLaw>
      <math xmlns="http://www.w3.org/1998/Math/MathML">
        <apply>
          <times/>
          <ci> kactabin2 </ci>
          <ci> S20 </ci>
          <ci> S111 </ci>
        </apply>
      </math>
    </kineticLaw>
  </reaction>
  <reaction id="R343" reversible="false">
    <listOfReactants>
      <speciesReference species="S20"/>
      <speciesReference species="S112"/>
    </listOfReactants>
    <listOfProducts>
      <speciesReference species="S75"/>
      <speciesReference species="S112"/>
    </listOfProducts>
    <kineticLaw>
      <math xmlns="http://www.w3.org/1998/Math/MathML">
        <apply>
          <times/>
          <ci> kactabin2 </ci>
          <ci> S20 </ci>
          <ci> S112 </ci>
        </apply>
      </math>
    </kineticLaw>
  </reaction>
  <reaction id="R344" reversible="false">
    <listOfReactants>
      <speciesReference species="S6"/>
      <speciesReference species="S120"/>
    </listOfReactants>
    <listOfProducts>
      <speciesReference species="S128"/>
    </listOfProducts>
    <kineticLaw>
      <math xmlns="http://www.w3.org/1998/Math/MathML">
```

```
        <apply>
          <times/>
          <ci> konveptp </ci>
          <ci> S6 </ci>
          <ci> S120 </ci>
        </apply>
      </math>
    </kineticLaw>
  </reaction>
  <reaction id="R345" reversible="false">
    <listOfReactants>
      <speciesReference species="S6"/>
      <speciesReference species="S121"/>
    </listOfReactants>
    <listOfProducts>
      <speciesReference species="S129"/>
    </listOfProducts>
    <kineticLaw>
      <math xmlns="http://www.w3.org/1998/Math/MathML">
        <apply>
          <times/>
          <ci> konveptp </ci>
          <ci> S6 </ci>
          <ci> S121 </ci>
        </apply>
      </math>
    </kineticLaw>
  </reaction>
  <reaction id="R346" reversible="false">
    <listOfReactants>
      <speciesReference species="S6"/>
      <speciesReference species="S122"/>
    </listOfReactants>
    <listOfProducts>
      <speciesReference species="S130"/>
    </listOfProducts>
    <kineticLaw>
      <math xmlns="http://www.w3.org/1998/Math/MathML">
        <apply>
          <times/>
          <ci> konveptp </ci>
          <ci> S6 </ci>
          <ci> S122 </ci>
        </apply>
      </math>
    </kineticLaw>
  </reaction>
  <reaction id="R347" reversible="false">
    <listOfReactants>
      <speciesReference species="S6"/>
      <speciesReference species="S123"/>
    </listOfReactants>
    <listOfProducts>
      <speciesReference species="S131"/>
    </listOfProducts>
    <kineticLaw>
      <math xmlns="http://www.w3.org/1998/Math/MathML">
```

```
        <apply>
          <times/>
          <ci> konveptp </ci>
          <ci> S6 </ci>
          <ci> S123 </ci>
        </apply>
      </math>
    </kineticLaw>
  </reaction>
  <reaction id="R348" reversible="false">
    <listOfReactants>
      <speciesReference species="S6"/>
      <speciesReference species="S124"/>
    </listOfReactants>
    <listOfProducts>
      <speciesReference species="S132"/>
    </listOfProducts>
    <kineticLaw>
      <math xmlns="http://www.w3.org/1998/Math/MathML">
        <apply>
          <times/>
          <ci> konveptp </ci>
          <ci> S6 </ci>
          <ci> S124 </ci>
        </apply>
      </math>
    </kineticLaw>
  </reaction>
  <reaction id="R349" reversible="false">
    <listOfReactants>
      <speciesReference species="S6"/>
      <speciesReference species="S125"/>
    </listOfReactants>
    <listOfProducts>
      <speciesReference species="S133"/>
    </listOfProducts>
    <kineticLaw>
      <math xmlns="http://www.w3.org/1998/Math/MathML">
        <apply>
          <times/>
          <ci> konveptp </ci>
          <ci> S6 </ci>
          <ci> S125 </ci>
        </apply>
      </math>
    </kineticLaw>
  </reaction>
  <reaction id="R350" reversible="false">
    <listOfReactants>
      <speciesReference species="S114"/>
    </listOfReactants>
    <listOfProducts>
      <speciesReference species="S6"/>
      <speciesReference species="S99"/>
    </listOfProducts>
    <kineticLaw>
      <math xmlns="http://www.w3.org/1998/Math/MathML">
```

```
      <apply>
        <times/>
        <cn> 3 </cn>
        <ci> koffveptp </ci>
        <ci> S114 </ci>
      </apply>
    </math>
  </kineticLaw>
</reaction>
<reaction id="R351" reversible="false">
  <listOfReactants>
    <speciesReference species="S115"/>
  </listOfReactants>
  <listOfProducts>
    <speciesReference species="S6"/>
    <speciesReference species="S100"/>
  </listOfProducts>
  <kineticLaw>
    <math xmlns="http://www.w3.org/1998/Math/MathML">
      <apply>
        <times/>
        <cn> 3 </cn>
        <ci> koffveptp </ci>
        <ci> S115 </ci>
      </apply>
    </math>
  </kineticLaw>
</reaction>
<reaction id="R352" reversible="false">
  <listOfReactants>
    <speciesReference species="S116"/>
  </listOfReactants>
  <listOfProducts>
    <speciesReference species="S6"/>
    <speciesReference species="S105"/>
  </listOfProducts>
  <kineticLaw>
    <math xmlns="http://www.w3.org/1998/Math/MathML">
      <apply>
        <times/>
        <cn> 3 </cn>
        <ci> koffveptp </ci>
        <ci> S116 </ci>
      </apply>
    </math>
  </kineticLaw>
</reaction>
<reaction id="R353" reversible="false">
  <listOfReactants>
    <speciesReference species="S117"/>
  </listOfReactants>
  <listOfProducts>
    <speciesReference species="S6"/>
    <speciesReference species="S106"/>
  </listOfProducts>
  <kineticLaw>
    <math xmlns="http://www.w3.org/1998/Math/MathML">
```

```xml
        <apply>
          <times/>
          <cn> 3 </cn>
          <ci> koffveptp </ci>
          <ci> S117 </ci>
        </apply>
      </math>
    </kineticLaw>
  </reaction>
  <reaction id="R354" reversible="false">
    <listOfReactants>
      <speciesReference species="S118"/>
    </listOfReactants>
    <listOfProducts>
      <speciesReference species="S6"/>
      <speciesReference species="S107"/>
    </listOfProducts>
    <kineticLaw>
      <math xmlns="http://www.w3.org/1998/Math/MathML">
        <apply>
          <times/>
          <cn> 2 </cn>
          <ci> koffveptp </ci>
          <ci> S118 </ci>
        </apply>
      </math>
    </kineticLaw>
  </reaction>
  <reaction id="R355" reversible="false">
    <listOfReactants>
      <speciesReference species="S119"/>
    </listOfReactants>
    <listOfProducts>
      <speciesReference species="S6"/>
      <speciesReference species="S108"/>
    </listOfProducts>
    <kineticLaw>
      <math xmlns="http://www.w3.org/1998/Math/MathML">
        <apply>
          <times/>
          <cn> 2 </cn>
          <ci> koffveptp </ci>
          <ci> S119 </ci>
        </apply>
      </math>
    </kineticLaw>
  </reaction>
  <reaction id="R356" reversible="false">
    <listOfReactants>
      <speciesReference species="S120"/>
    </listOfReactants>
    <listOfProducts>
      <speciesReference species="S6"/>
      <speciesReference species="S109"/>
    </listOfProducts>
    <kineticLaw>
      <math xmlns="http://www.w3.org/1998/Math/MathML">
```

```
      <apply>
        <times/>
        <ci> koffveptp </ci>
        <ci> S120 </ci>
      </apply>
    </math>
  </kineticLaw>
</reaction>
<reaction id="R357" reversible="false">
  <listOfReactants>
    <speciesReference species="S121"/>
  </listOfReactants>
  <listOfProducts>
    <speciesReference species="S6"/>
    <speciesReference species="S110"/>
  </listOfProducts>
  <kineticLaw>
    <math xmlns="http://www.w3.org/1998/Math/MathML">
      <apply>
        <times/>
        <ci> koffveptp </ci>
        <ci> S121 </ci>
      </apply>
    </math>
  </kineticLaw>
</reaction>
<reaction id="R358" reversible="false">
  <listOfReactants>
    <speciesReference species="S122"/>
  </listOfReactants>
  <listOfProducts>
    <speciesReference species="S6"/>
    <speciesReference species="S111"/>
  </listOfProducts>
  <kineticLaw>
    <math xmlns="http://www.w3.org/1998/Math/MathML">
      <apply>
        <times/>
        <ci> koffveptp </ci>
        <ci> S122 </ci>
      </apply>
    </math>
  </kineticLaw>
</reaction>
<reaction id="R359" reversible="false">
  <listOfReactants>
    <speciesReference species="S123"/>
  </listOfReactants>
  <listOfProducts>
    <speciesReference species="S6"/>
    <speciesReference species="S112"/>
  </listOfProducts>
  <kineticLaw>
    <math xmlns="http://www.w3.org/1998/Math/MathML">
      <apply>
        <times/>
        <ci> koffveptp </ci>
```

```
        <ci> S123 </ci>
      </apply>
    </math>
  </kineticLaw>
</reaction>
<reaction id="R360" reversible="false">
  <listOfReactants>
    <speciesReference species="S114"/>
  </listOfReactants>
  <listOfProducts>
    <speciesReference species="S6"/>
    <speciesReference species="S128"/>
  </listOfProducts>
  <kineticLaw>
    <math xmlns="http://www.w3.org/1998/Math/MathML">
      <apply>
        <times/>
        <cn> 3 </cn>
        <ci> kactveptp_ang1 </ci>
        <ci> S114 </ci>
      </apply>
    </math>
  </kineticLaw>
</reaction>
<reaction id="R361" reversible="false">
  <listOfReactants>
    <speciesReference species="S116"/>
  </listOfReactants>
  <listOfProducts>
    <speciesReference species="S6"/>
    <speciesReference species="S129"/>
  </listOfProducts>
  <kineticLaw>
    <math xmlns="http://www.w3.org/1998/Math/MathML">
      <apply>
        <times/>
        <cn> 3 </cn>
        <ci> kactveptp_ang1 </ci>
        <ci> S116 </ci>
      </apply>
    </math>
  </kineticLaw>
</reaction>
<reaction id="R362" reversible="false">
  <listOfReactants>
    <speciesReference species="S118"/>
  </listOfReactants>
  <listOfProducts>
    <speciesReference species="S6"/>
    <speciesReference species="S132"/>
  </listOfProducts>
  <kineticLaw>
    <math xmlns="http://www.w3.org/1998/Math/MathML">
      <apply>
        <times/>
        <cn> 2 </cn>
        <ci> kactveptp_ang1 </ci>
```

```
        <ci> S118 </ci>
      </apply>
    </math>
  </kineticLaw>
</reaction>
<reaction id="R363" reversible="false">
  <listOfReactants>
    <speciesReference species="S120"/>
  </listOfReactants>
  <listOfProducts>
    <speciesReference species="S6"/>
    <speciesReference species="S134"/>
  </listOfProducts>
  <kineticLaw>
    <math xmlns="http://www.w3.org/1998/Math/MathML">
      <apply>
        <times/>
        <ci> kactveptp_ang1 </ci>
        <ci> S120 </ci>
      </apply>
    </math>
  </kineticLaw>
</reaction>
<reaction id="R364" reversible="false">
  <listOfReactants>
    <speciesReference species="S121"/>
  </listOfReactants>
  <listOfProducts>
    <speciesReference species="S6"/>
    <speciesReference species="S135"/>
  </listOfProducts>
  <kineticLaw>
    <math xmlns="http://www.w3.org/1998/Math/MathML">
      <apply>
        <times/>
        <ci> kactveptp_ang1 </ci>
        <ci> S121 </ci>
      </apply>
    </math>
  </kineticLaw>
</reaction>
<reaction id="R365" reversible="false">
  <listOfReactants>
    <speciesReference species="S115"/>
  </listOfReactants>
  <listOfProducts>
    <speciesReference species="S6"/>
    <speciesReference species="S130"/>
  </listOfProducts>
  <kineticLaw>
    <math xmlns="http://www.w3.org/1998/Math/MathML">
      <apply>
        <times/>
        <cn> 3 </cn>
        <ci> kactveptp_ang2 </ci>
        <ci> S115 </ci>
      </apply>
```

```
    </math>
  </kineticLaw>
</reaction>
<reaction id="R366" reversible="false">
  <listOfReactants>
    <speciesReference species="S117"/>
  </listOfReactants>
  <listOfProducts>
    <speciesReference species="S6"/>
    <speciesReference species="S131"/>
  </listOfProducts>
  <kineticLaw>
    <math xmlns="http://www.w3.org/1998/Math/MathML">
      <apply>
        <times/>
        <cn> 3 </cn>
        <ci> kactveptp_ang2 </ci>
        <ci> S117 </ci>
      </apply>
    </math>
  </kineticLaw>
</reaction>
<reaction id="R367" reversible="false">
  <listOfReactants>
    <speciesReference species="S119"/>
  </listOfReactants>
  <listOfProducts>
    <speciesReference species="S6"/>
    <speciesReference species="S133"/>
  </listOfProducts>
  <kineticLaw>
    <math xmlns="http://www.w3.org/1998/Math/MathML">
      <apply>
        <times/>
        <cn> 2 </cn>
        <ci> kactveptp_ang2 </ci>
        <ci> S119 </ci>
      </apply>
    </math>
  </kineticLaw>
</reaction>
<reaction id="R368" reversible="false">
  <listOfReactants>
    <speciesReference species="S122"/>
  </listOfReactants>
  <listOfProducts>
    <speciesReference species="S6"/>
    <speciesReference species="S136"/>
  </listOfProducts>
  <kineticLaw>
    <math xmlns="http://www.w3.org/1998/Math/MathML">
      <apply>
        <times/>
        <ci> kactveptp_ang2 </ci>
        <ci> S122 </ci>
      </apply>
    </math>
```

```
    </kineticLaw>
  </reaction>
  <reaction id="R369" reversible="false">
    <listOfReactants>
      <speciesReference species="S123"/>
    </listOfReactants>
    <listOfProducts>
      <speciesReference species="S6"/>
      <speciesReference species="S137"/>
    </listOfProducts>
    <kineticLaw>
      <math xmlns="http://www.w3.org/1998/Math/MathML">
        <apply>
          <times/>
          <ci> kactveptp_ang2 </ci>
          <ci> S123 </ci>
        </apply>
      </math>
    </kineticLaw>
  </reaction>
  <reaction id="R370" reversible="false">
    <listOfReactants>
      <speciesReference species="S13"/>
      <speciesReference species="S114"/>
    </listOfReactants>
    <listOfProducts>
      <speciesReference species="S73"/>
      <speciesReference species="S114"/>
    </listOfProducts>
    <kineticLaw>
      <math xmlns="http://www.w3.org/1998/Math/MathML">
        <apply>
          <times/>
          <ci> kactPI3KTie2 </ci>
          <ci> S13 </ci>
          <ci> S114 </ci>
        </apply>
      </math>
    </kineticLaw>
  </reaction>
  <reaction id="R371" reversible="false">
    <listOfReactants>
      <speciesReference species="S13"/>
      <speciesReference species="S115"/>
    </listOfReactants>
    <listOfProducts>
      <speciesReference species="S73"/>
      <speciesReference species="S115"/>
    </listOfProducts>
    <kineticLaw>
      <math xmlns="http://www.w3.org/1998/Math/MathML">
        <apply>
          <times/>
          <ci> kactPI3KTie2 </ci>
          <ci> S13 </ci>
          <ci> S115 </ci>
        </apply>
```

```
      </math>
    </kineticLaw>
  </reaction>
  <reaction id="R372" reversible="false">
    <listOfReactants>
      <speciesReference species="S13"/>
      <speciesReference species="S116"/>
    </listOfReactants>
    <listOfProducts>
      <speciesReference species="S73"/>
      <speciesReference species="S116"/>
    </listOfProducts>
    <kineticLaw>
      <math xmlns="http://www.w3.org/1998/Math/MathML">
        <apply>
          <times/>
          <ci> kactPI3KTie2 </ci>
          <ci> S13 </ci>
          <ci> S116 </ci>
        </apply>
      </math>
    </kineticLaw>
  </reaction>
  <reaction id="R373" reversible="false">
    <listOfReactants>
      <speciesReference species="S13"/>
      <speciesReference species="S117"/>
    </listOfReactants>
    <listOfProducts>
      <speciesReference species="S73"/>
      <speciesReference species="S117"/>
    </listOfProducts>
    <kineticLaw>
      <math xmlns="http://www.w3.org/1998/Math/MathML">
        <apply>
          <times/>
          <ci> kactPI3KTie2 </ci>
          <ci> S13 </ci>
          <ci> S117 </ci>
        </apply>
      </math>
    </kineticLaw>
  </reaction>
  <reaction id="R374" reversible="false">
    <listOfReactants>
      <speciesReference species="S13"/>
      <speciesReference species="S118"/>
    </listOfReactants>
    <listOfProducts>
      <speciesReference species="S73"/>
      <speciesReference species="S118"/>
    </listOfProducts>
    <kineticLaw>
      <math xmlns="http://www.w3.org/1998/Math/MathML">
        <apply>
          <times/>
          <ci> kactPI3KTie2 </ci>
```

```
        <ci> S13 </ci>
        <ci> S118 </ci>
      </apply>
    </math>
  </kineticLaw>
</reaction>
<reaction id="R375" reversible="false">
  <listOfReactants>
    <speciesReference species="S13"/>
    <speciesReference species="S119"/>
  </listOfReactants>
  <listOfProducts>
    <speciesReference species="S73"/>
    <speciesReference species="S119"/>
  </listOfProducts>
  <kineticLaw>
    <math xmlns="http://www.w3.org/1998/Math/MathML">
      <apply>
        <times/>
        <ci> kactPI3KTie2 </ci>
        <ci> S13 </ci>
        <ci> S119 </ci>
      </apply>
    </math>
  </kineticLaw>
</reaction>
<reaction id="R376" reversible="false">
  <listOfReactants>
    <speciesReference species="S13"/>
    <speciesReference species="S120"/>
  </listOfReactants>
  <listOfProducts>
    <speciesReference species="S73"/>
    <speciesReference species="S120"/>
  </listOfProducts>
  <kineticLaw>
    <math xmlns="http://www.w3.org/1998/Math/MathML">
      <apply>
        <times/>
        <ci> kactPI3KTie2 </ci>
        <ci> S13 </ci>
        <ci> S120 </ci>
      </apply>
    </math>
  </kineticLaw>
</reaction>
<reaction id="R377" reversible="false">
  <listOfReactants>
    <speciesReference species="S13"/>
    <speciesReference species="S121"/>
  </listOfReactants>
  <listOfProducts>
    <speciesReference species="S73"/>
    <speciesReference species="S121"/>
  </listOfProducts>
  <kineticLaw>
    <math xmlns="http://www.w3.org/1998/Math/MathML">
```

```
        <apply>
          <times/>
          <ci> kactPI3KTie2 </ci>
          <ci> S13 </ci>
          <ci> S121 </ci>
        </apply>
      </math>
    </kineticLaw>
  </reaction>
  <reaction id="R378" reversible="false">
    <listOfReactants>
      <speciesReference species="S13"/>
      <speciesReference species="S122"/>
    </listOfReactants>
    <listOfProducts>
      <speciesReference species="S73"/>
      <speciesReference species="S122"/>
    </listOfProducts>
    <kineticLaw>
      <math xmlns="http://www.w3.org/1998/Math/MathML">
        <apply>
          <times/>
          <ci> kactPI3KTie2 </ci>
          <ci> S13 </ci>
          <ci> S122 </ci>
        </apply>
      </math>
    </kineticLaw>
  </reaction>
  <reaction id="R379" reversible="false">
    <listOfReactants>
      <speciesReference species="S13"/>
      <speciesReference species="S123"/>
    </listOfReactants>
    <listOfProducts>
      <speciesReference species="S73"/>
      <speciesReference species="S123"/>
    </listOfProducts>
    <kineticLaw>
      <math xmlns="http://www.w3.org/1998/Math/MathML">
        <apply>
          <times/>
          <ci> kactPI3KTie2 </ci>
          <ci> S13 </ci>
          <ci> S123 </ci>
        </apply>
      </math>
    </kineticLaw>
  </reaction>
  <reaction id="R380" reversible="false">
    <listOfReactants>
      <speciesReference species="S13"/>
      <speciesReference species="S124"/>
    </listOfReactants>
    <listOfProducts>
      <speciesReference species="S73"/>
      <speciesReference species="S124"/>
```

```
    &lt;/listOfProducts&gt;
    &lt;kineticLaw&gt;
      &lt;math xmlns="http://www.w3.org/1998/Math/MathML"&gt;
        &lt;apply&gt;
          &lt;times/&gt;
          &lt;ci&gt; kactPI3KTie2 &lt;/ci&gt;
          &lt;ci&gt; S13 &lt;/ci&gt;
          &lt;ci&gt; S124 &lt;/ci&gt;
        &lt;/apply&gt;
      &lt;/math&gt;
    &lt;/kineticLaw&gt;
  &lt;/reaction&gt;
  &lt;reaction id="R381" reversible="false"&gt;
    &lt;listOfReactants&gt;
      &lt;speciesReference species="S13"/&gt;
      &lt;speciesReference species="S125"/&gt;
    &lt;/listOfReactants&gt;
    &lt;listOfProducts&gt;
      &lt;speciesReference species="S73"/&gt;
      &lt;speciesReference species="S125"/&gt;
    &lt;/listOfProducts&gt;
    &lt;kineticLaw&gt;
      &lt;math xmlns="http://www.w3.org/1998/Math/MathML"&gt;
        &lt;apply&gt;
          &lt;times/&gt;
          &lt;ci&gt; kactPI3KTie2 &lt;/ci&gt;
          &lt;ci&gt; S13 &lt;/ci&gt;
          &lt;ci&gt; S125 &lt;/ci&gt;
        &lt;/apply&gt;
      &lt;/math&gt;
    &lt;/kineticLaw&gt;
  &lt;/reaction&gt;
  &lt;reaction id="R382" reversible="false"&gt;
    &lt;listOfReactants&gt;
      &lt;speciesReference species="S84"/&gt;
      &lt;speciesReference species="S126"/&gt;
    &lt;/listOfReactants&gt;
    &lt;listOfProducts&gt;
      &lt;speciesReference species="S113"/&gt;
    &lt;/listOfProducts&gt;
    &lt;kineticLaw&gt;
      &lt;math xmlns="http://www.w3.org/1998/Math/MathML"&gt;
        &lt;apply&gt;
          &lt;times/&gt;
          &lt;ci&gt; konAKTPIP3 &lt;/ci&gt;
          &lt;ci&gt; S84 &lt;/ci&gt;
          &lt;ci&gt; S126 &lt;/ci&gt;
        &lt;/apply&gt;
      &lt;/math&gt;
    &lt;/kineticLaw&gt;
  &lt;/reaction&gt;
  &lt;reaction id="R383" reversible="false"&gt;
    &lt;listOfReactants&gt;
      &lt;speciesReference species="S127"/&gt;
    &lt;/listOfReactants&gt;
    &lt;listOfProducts&gt;
      &lt;speciesReference species="S84"/&gt;
```

```
      <speciesReference species="S138"/>
    </listOfProducts>
    <kineticLaw>
      <math xmlns="http://www.w3.org/1998/Math/MathML">
        <apply>
          <times/>
          <ci> koffAKTPIP3 </ci>
          <ci> S127 </ci>
        </apply>
      </math>
    </kineticLaw>
  </reaction>
  <reaction id="R384" reversible="false">
    <listOfReactants>
      <speciesReference species="S126"/>
    </listOfReactants>
    <listOfProducts>
      <speciesReference species="S14"/>
    </listOfProducts>
    <kineticLaw>
      <math xmlns="http://www.w3.org/1998/Math/MathML">
        <apply>
          <times/>
          <ci> kdp473AKTPPase </ci>
          <ci> S126 </ci>
        </apply>
      </math>
    </kineticLaw>
  </reaction>
  <reaction id="R385" reversible="false">
    <listOfReactants>
      <speciesReference species="S127"/>
    </listOfReactants>
    <listOfProducts>
      <speciesReference species="S139"/>
    </listOfProducts>
    <kineticLaw>
      <math xmlns="http://www.w3.org/1998/Math/MathML">
        <apply>
          <times/>
          <ci> kdp473AKTPPase </ci>
          <ci> S127 </ci>
        </apply>
      </math>
    </kineticLaw>
  </reaction>
  <reaction id="R386" reversible="false">
    <listOfReactants>
      <speciesReference species="S127"/>
    </listOfReactants>
    <listOfProducts>
      <speciesReference species="S113"/>
    </listOfProducts>
    <kineticLaw>
      <math xmlns="http://www.w3.org/1998/Math/MathML">
        <apply>
          <times/>
```

```
        <ci> kdp308AKTPPase </ci>
        <ci> S127 </ci>
      </apply>
    </math>
  </kineticLaw>
</reaction>
<reaction id="R387" reversible="false">
  <listOfReactants>
    <speciesReference species="S16"/>
    <speciesReference species="S114"/>
  </listOfReactants>
  <listOfProducts>
    <speciesReference species="S74"/>
    <speciesReference species="S114"/>
  </listOfProducts>
  <kineticLaw>
    <math xmlns="http://www.w3.org/1998/Math/MathML">
      <apply>
        <times/>
        <ci> kprhoa </ci>
        <ci> S16 </ci>
        <ci> S114 </ci>
      </apply>
    </math>
  </kineticLaw>
</reaction>
<reaction id="R388" reversible="false">
  <listOfReactants>
    <speciesReference species="S16"/>
    <speciesReference species="S115"/>
  </listOfReactants>
  <listOfProducts>
    <speciesReference species="S74"/>
    <speciesReference species="S115"/>
  </listOfProducts>
  <kineticLaw>
    <math xmlns="http://www.w3.org/1998/Math/MathML">
      <apply>
        <times/>
        <ci> kprhoa </ci>
        <ci> S16 </ci>
        <ci> S115 </ci>
      </apply>
    </math>
  </kineticLaw>
</reaction>
<reaction id="R389" reversible="false">
  <listOfReactants>
    <speciesReference species="S16"/>
    <speciesReference species="S116"/>
  </listOfReactants>
  <listOfProducts>
    <speciesReference species="S74"/>
    <speciesReference species="S116"/>
  </listOfProducts>
  <kineticLaw>
    <math xmlns="http://www.w3.org/1998/Math/MathML">
```

```
        <apply>
          <times/>
          <ci> kprhoa </ci>
          <ci> S16 </ci>
          <ci> S116 </ci>
        </apply>
      </math>
    </kineticLaw>
  </reaction>
  <reaction id="R390" reversible="false">
    <listOfReactants>
      <speciesReference species="S16"/>
      <speciesReference species="S117"/>
    </listOfReactants>
    <listOfProducts>
      <speciesReference species="S74"/>
      <speciesReference species="S117"/>
    </listOfProducts>
    <kineticLaw>
      <math xmlns="http://www.w3.org/1998/Math/MathML">
        <apply>
          <times/>
          <ci> kprhoa </ci>
          <ci> S16 </ci>
          <ci> S117 </ci>
        </apply>
      </math>
    </kineticLaw>
  </reaction>
  <reaction id="R391" reversible="false">
    <listOfReactants>
      <speciesReference species="S16"/>
      <speciesReference species="S118"/>
    </listOfReactants>
    <listOfProducts>
      <speciesReference species="S74"/>
      <speciesReference species="S118"/>
    </listOfProducts>
    <kineticLaw>
      <math xmlns="http://www.w3.org/1998/Math/MathML">
        <apply>
          <times/>
          <ci> kprhoa </ci>
          <ci> S16 </ci>
          <ci> S118 </ci>
        </apply>
      </math>
    </kineticLaw>
  </reaction>
  <reaction id="R392" reversible="false">
    <listOfReactants>
      <speciesReference species="S16"/>
      <speciesReference species="S119"/>
    </listOfReactants>
    <listOfProducts>
      <speciesReference species="S74"/>
      <speciesReference species="S119"/>
```

```
    &lt;/listOfProducts&gt;
    &lt;kineticLaw&gt;
      &lt;math xmlns="http://www.w3.org/1998/Math/MathML"&gt;
        &lt;apply&gt;
          &lt;times/&gt;
          &lt;ci&gt; kprhoa &lt;/ci&gt;
          &lt;ci&gt; S16 &lt;/ci&gt;
          &lt;ci&gt; S119 &lt;/ci&gt;
        &lt;/apply&gt;
      &lt;/math&gt;
    &lt;/kineticLaw&gt;
  &lt;/reaction&gt;
  &lt;reaction id="R393" reversible="false"&gt;
    &lt;listOfReactants&gt;
      &lt;speciesReference species="S16"/&gt;
      &lt;speciesReference species="S120"/&gt;
    &lt;/listOfReactants&gt;
    &lt;listOfProducts&gt;
      &lt;speciesReference species="S74"/&gt;
      &lt;speciesReference species="S120"/&gt;
    &lt;/listOfProducts&gt;
    &lt;kineticLaw&gt;
      &lt;math xmlns="http://www.w3.org/1998/Math/MathML"&gt;
        &lt;apply&gt;
          &lt;times/&gt;
          &lt;ci&gt; kprhoa &lt;/ci&gt;
          &lt;ci&gt; S16 &lt;/ci&gt;
          &lt;ci&gt; S120 &lt;/ci&gt;
        &lt;/apply&gt;
      &lt;/math&gt;
    &lt;/kineticLaw&gt;
  &lt;/reaction&gt;
  &lt;reaction id="R394" reversible="false"&gt;
    &lt;listOfReactants&gt;
      &lt;speciesReference species="S16"/&gt;
      &lt;speciesReference species="S121"/&gt;
    &lt;/listOfReactants&gt;
    &lt;listOfProducts&gt;
      &lt;speciesReference species="S74"/&gt;
      &lt;speciesReference species="S121"/&gt;
    &lt;/listOfProducts&gt;
    &lt;kineticLaw&gt;
      &lt;math xmlns="http://www.w3.org/1998/Math/MathML"&gt;
        &lt;apply&gt;
          &lt;times/&gt;
          &lt;ci&gt; kprhoa &lt;/ci&gt;
          &lt;ci&gt; S16 &lt;/ci&gt;
          &lt;ci&gt; S121 &lt;/ci&gt;
        &lt;/apply&gt;
      &lt;/math&gt;
    &lt;/kineticLaw&gt;
  &lt;/reaction&gt;
  &lt;reaction id="R395" reversible="false"&gt;
    &lt;listOfReactants&gt;
      &lt;speciesReference species="S16"/&gt;
      &lt;speciesReference species="S122"/&gt;
    &lt;/listOfReactants&gt;
```

```xml
    <listOfProducts>
      <speciesReference species="S74"/>
      <speciesReference species="S122"/>
    </listOfProducts>
    <kineticLaw>
      <math xmlns="http://www.w3.org/1998/Math/MathML">
        <apply>
          <times/>
          <ci> kprhoa </ci>
          <ci> S16 </ci>
          <ci> S122 </ci>
        </apply>
      </math>
    </kineticLaw>
  </reaction>
  <reaction id="R396" reversible="false">
    <listOfReactants>
      <speciesReference species="S16"/>
      <speciesReference species="S123"/>
    </listOfReactants>
    <listOfProducts>
      <speciesReference species="S74"/>
      <speciesReference species="S123"/>
    </listOfProducts>
    <kineticLaw>
      <math xmlns="http://www.w3.org/1998/Math/MathML">
        <apply>
          <times/>
          <ci> kprhoa </ci>
          <ci> S16 </ci>
          <ci> S123 </ci>
        </apply>
      </math>
    </kineticLaw>
  </reaction>
  <reaction id="R397" reversible="false">
    <listOfReactants>
      <speciesReference species="S16"/>
      <speciesReference species="S124"/>
    </listOfReactants>
    <listOfProducts>
      <speciesReference species="S74"/>
      <speciesReference species="S124"/>
    </listOfProducts>
    <kineticLaw>
      <math xmlns="http://www.w3.org/1998/Math/MathML">
        <apply>
          <times/>
          <ci> kprhoa </ci>
          <ci> S16 </ci>
          <ci> S124 </ci>
        </apply>
      </math>
    </kineticLaw>
  </reaction>
  <reaction id="R398" reversible="false">
    <listOfReactants>
```

```
      <speciesReference species="S16"/>
      <speciesReference species="S125"/>
    </listOfReactants>
    <listOfProducts>
      <speciesReference species="S74"/>
      <speciesReference species="S125"/>
    </listOfProducts>
    <kineticLaw>
      <math xmlns="http://www.w3.org/1998/Math/MathML">
        <apply>
          <times/>
          <ci> kprhoa </ci>
          <ci> S16 </ci>
          <ci> S125 </ci>
        </apply>
      </math>
    </kineticLaw>
  </reaction>
  <reaction id="R399" reversible="false">
    <listOfReactants>
      <speciesReference species="S20"/>
      <speciesReference species="S114"/>
    </listOfReactants>
    <listOfProducts>
      <speciesReference species="S75"/>
      <speciesReference species="S114"/>
    </listOfProducts>
    <kineticLaw>
      <math xmlns="http://www.w3.org/1998/Math/MathML">
        <apply>
          <times/>
          <ci> kactabin2 </ci>
          <ci> S20 </ci>
          <ci> S114 </ci>
        </apply>
      </math>
    </kineticLaw>
  </reaction>
  <reaction id="R400" reversible="false">
    <listOfReactants>
      <speciesReference species="S20"/>
      <speciesReference species="S115"/>
    </listOfReactants>
    <listOfProducts>
      <speciesReference species="S75"/>
      <speciesReference species="S115"/>
    </listOfProducts>
    <kineticLaw>
      <math xmlns="http://www.w3.org/1998/Math/MathML">
        <apply>
          <times/>
          <ci> kactabin2 </ci>
          <ci> S20 </ci>
          <ci> S115 </ci>
        </apply>
      </math>
    </kineticLaw>
```

```
</reaction>
<reaction id="R401" reversible="false">
  <listOfReactants>
    <speciesReference species="S20"/>
    <speciesReference species="S116"/>
  </listOfReactants>
  <listOfProducts>
    <speciesReference species="S75"/>
    <speciesReference species="S116"/>
  </listOfProducts>
  <kineticLaw>
    <math xmlns="http://www.w3.org/1998/Math/MathML">
      <apply>
        <times/>
        <ci> kactabin2 </ci>
        <ci> S20 </ci>
        <ci> S116 </ci>
      </apply>
    </math>
  </kineticLaw>
</reaction>
<reaction id="R402" reversible="false">
  <listOfReactants>
    <speciesReference species="S20"/>
    <speciesReference species="S117"/>
  </listOfReactants>
  <listOfProducts>
    <speciesReference species="S75"/>
    <speciesReference species="S117"/>
  </listOfProducts>
  <kineticLaw>
    <math xmlns="http://www.w3.org/1998/Math/MathML">
      <apply>
        <times/>
        <ci> kactabin2 </ci>
        <ci> S20 </ci>
        <ci> S117 </ci>
      </apply>
    </math>
  </kineticLaw>
</reaction>
<reaction id="R403" reversible="false">
  <listOfReactants>
    <speciesReference species="S20"/>
    <speciesReference species="S118"/>
  </listOfReactants>
  <listOfProducts>
    <speciesReference species="S75"/>
    <speciesReference species="S118"/>
  </listOfProducts>
  <kineticLaw>
    <math xmlns="http://www.w3.org/1998/Math/MathML">
      <apply>
        <times/>
        <ci> kactabin2 </ci>
        <ci> S20 </ci>
        <ci> S118 </ci>
```

```
      </apply>
    </math>
  </kineticLaw>
</reaction>
<reaction id="R404" reversible="false">
  <listOfReactants>
    <speciesReference species="S20"/>
    <speciesReference species="S119"/>
  </listOfReactants>
  <listOfProducts>
    <speciesReference species="S75"/>
    <speciesReference species="S119"/>
  </listOfProducts>
  <kineticLaw>
    <math xmlns="http://www.w3.org/1998/Math/MathML">
      <apply>
        <times/>
        <ci> kactabin2 </ci>
        <ci> S20 </ci>
        <ci> S119 </ci>
      </apply>
    </math>
  </kineticLaw>
</reaction>
<reaction id="R405" reversible="false">
  <listOfReactants>
    <speciesReference species="S20"/>
    <speciesReference species="S120"/>
  </listOfReactants>
  <listOfProducts>
    <speciesReference species="S75"/>
    <speciesReference species="S120"/>
  </listOfProducts>
  <kineticLaw>
    <math xmlns="http://www.w3.org/1998/Math/MathML">
      <apply>
        <times/>
        <ci> kactabin2 </ci>
        <ci> S20 </ci>
        <ci> S120 </ci>
      </apply>
    </math>
  </kineticLaw>
</reaction>
<reaction id="R406" reversible="false">
  <listOfReactants>
    <speciesReference species="S20"/>
    <speciesReference species="S121"/>
  </listOfReactants>
  <listOfProducts>
    <speciesReference species="S75"/>
    <speciesReference species="S121"/>
  </listOfProducts>
  <kineticLaw>
    <math xmlns="http://www.w3.org/1998/Math/MathML">
      <apply>
        <times/>
```

```
        &lt;ci&gt; kactabin2 &lt;/ci&gt;
        &lt;ci&gt; S20 &lt;/ci&gt;
        &lt;ci&gt; S121 &lt;/ci&gt;
      &lt;/apply&gt;
    &lt;/math&gt;
  &lt;/kineticLaw&gt;
&lt;/reaction&gt;
&lt;reaction id="R407" reversible="false"&gt;
  &lt;listOfReactants&gt;
    &lt;speciesReference species="S20"/&gt;
    &lt;speciesReference species="S122"/&gt;
  &lt;/listOfReactants&gt;
  &lt;listOfProducts&gt;
    &lt;speciesReference species="S75"/&gt;
    &lt;speciesReference species="S122"/&gt;
  &lt;/listOfProducts&gt;
  &lt;kineticLaw&gt;
    &lt;math xmlns="http://www.w3.org/1998/Math/MathML"&gt;
      &lt;apply&gt;
        &lt;times/&gt;
        &lt;ci&gt; kactabin2 &lt;/ci&gt;
        &lt;ci&gt; S20 &lt;/ci&gt;
        &lt;ci&gt; S122 &lt;/ci&gt;
      &lt;/apply&gt;
    &lt;/math&gt;
  &lt;/kineticLaw&gt;
&lt;/reaction&gt;
&lt;reaction id="R408" reversible="false"&gt;
  &lt;listOfReactants&gt;
    &lt;speciesReference species="S20"/&gt;
    &lt;speciesReference species="S123"/&gt;
  &lt;/listOfReactants&gt;
  &lt;listOfProducts&gt;
    &lt;speciesReference species="S75"/&gt;
    &lt;speciesReference species="S123"/&gt;
  &lt;/listOfProducts&gt;
  &lt;kineticLaw&gt;
    &lt;math xmlns="http://www.w3.org/1998/Math/MathML"&gt;
      &lt;apply&gt;
        &lt;times/&gt;
        &lt;ci&gt; kactabin2 &lt;/ci&gt;
        &lt;ci&gt; S20 &lt;/ci&gt;
        &lt;ci&gt; S123 &lt;/ci&gt;
      &lt;/apply&gt;
    &lt;/math&gt;
  &lt;/kineticLaw&gt;
&lt;/reaction&gt;
&lt;reaction id="R409" reversible="false"&gt;
  &lt;listOfReactants&gt;
    &lt;speciesReference species="S20"/&gt;
    &lt;speciesReference species="S124"/&gt;
  &lt;/listOfReactants&gt;
  &lt;listOfProducts&gt;
    &lt;speciesReference species="S75"/&gt;
    &lt;speciesReference species="S124"/&gt;
  &lt;/listOfProducts&gt;
  &lt;kineticLaw&gt;
```

```
    <math xmlns="http://www.w3.org/1998/Math/MathML">
      <apply>
        <times/>
        <ci> kactabin2 </ci>
        <ci> S20 </ci>
        <ci> S124 </ci>
      </apply>
    </math>
  </kineticLaw>
</reaction>
<reaction id="R410" reversible="false">
  <listOfReactants>
    <speciesReference species="S20"/>
    <speciesReference species="S125"/>
  </listOfReactants>
  <listOfProducts>
    <speciesReference species="S75"/>
    <speciesReference species="S125"/>
  </listOfProducts>
  <kineticLaw>
    <math xmlns="http://www.w3.org/1998/Math/MathML">
      <apply>
        <times/>
        <ci> kactabin2 </ci>
        <ci> S20 </ci>
        <ci> S125 </ci>
      </apply>
    </math>
  </kineticLaw>
</reaction>
<reaction id="R411" reversible="false">
  <listOfReactants>
    <speciesReference species="S6"/>
    <speciesReference species="S134"/>
  </listOfReactants>
  <listOfProducts>
    <speciesReference species="S140"/>
  </listOfProducts>
  <kineticLaw>
    <math xmlns="http://www.w3.org/1998/Math/MathML">
      <apply>
        <times/>
        <ci> konveptp </ci>
        <ci> S6 </ci>
        <ci> S134 </ci>
      </apply>
    </math>
  </kineticLaw>
</reaction>
<reaction id="R412" reversible="false">
  <listOfReactants>
    <speciesReference species="S6"/>
    <speciesReference species="S135"/>
  </listOfReactants>
  <listOfProducts>
    <speciesReference species="S141"/>
  </listOfProducts>
```

```xml
      <kineticLaw>
        <math xmlns="http://www.w3.org/1998/Math/MathML">
          <apply>
            <times/>
            <ci> konveptp </ci>
            <ci> S6 </ci>
            <ci> S135 </ci>
          </apply>
        </math>
      </kineticLaw>
    </reaction>
    <reaction id="R413" reversible="false">
      <listOfReactants>
        <speciesReference species="S6"/>
        <speciesReference species="S136"/>
      </listOfReactants>
      <listOfProducts>
        <speciesReference species="S142"/>
      </listOfProducts>
      <kineticLaw>
        <math xmlns="http://www.w3.org/1998/Math/MathML">
          <apply>
            <times/>
            <ci> konveptp </ci>
            <ci> S6 </ci>
            <ci> S136 </ci>
          </apply>
        </math>
      </kineticLaw>
    </reaction>
    <reaction id="R414" reversible="false">
      <listOfReactants>
        <speciesReference species="S6"/>
        <speciesReference species="S137"/>
      </listOfReactants>
      <listOfProducts>
        <speciesReference species="S143"/>
      </listOfProducts>
      <kineticLaw>
        <math xmlns="http://www.w3.org/1998/Math/MathML">
          <apply>
            <times/>
            <ci> konveptp </ci>
            <ci> S6 </ci>
            <ci> S137 </ci>
          </apply>
        </math>
      </kineticLaw>
    </reaction>
    <reaction id="R415" reversible="false">
      <listOfReactants>
        <speciesReference species="S128"/>
      </listOfReactants>
      <listOfProducts>
        <speciesReference species="S6"/>
        <speciesReference species="S120"/>
      </listOfProducts>
```

```
    <kineticLaw>
      <math xmlns="http://www.w3.org/1998/Math/MathML">
        <apply>
          <times/>
          <cn> 2 </cn>
          <ci> koffveptp </ci>
          <ci> S128 </ci>
        </apply>
      </math>
    </kineticLaw>
  </reaction>
  <reaction id="R416" reversible="false">
    <listOfReactants>
      <speciesReference species="S129"/>
    </listOfReactants>
    <listOfProducts>
      <speciesReference species="S6"/>
      <speciesReference species="S121"/>
    </listOfProducts>
    <kineticLaw>
      <math xmlns="http://www.w3.org/1998/Math/MathML">
        <apply>
          <times/>
          <cn> 2 </cn>
          <ci> koffveptp </ci>
          <ci> S129 </ci>
        </apply>
      </math>
    </kineticLaw>
  </reaction>
  <reaction id="R417" reversible="false">
    <listOfReactants>
      <speciesReference species="S130"/>
    </listOfReactants>
    <listOfProducts>
      <speciesReference species="S6"/>
      <speciesReference species="S122"/>
    </listOfProducts>
    <kineticLaw>
      <math xmlns="http://www.w3.org/1998/Math/MathML">
        <apply>
          <times/>
          <cn> 2 </cn>
          <ci> koffveptp </ci>
          <ci> S130 </ci>
        </apply>
      </math>
    </kineticLaw>
  </reaction>
  <reaction id="R418" reversible="false">
    <listOfReactants>
      <speciesReference species="S131"/>
    </listOfReactants>
    <listOfProducts>
      <speciesReference species="S6"/>
      <speciesReference species="S123"/>
    </listOfProducts>
```

```
    <kineticLaw>
      <math xmlns="http://www.w3.org/1998/Math/MathML">
        <apply>
          <times/>
          <cn> 2 </cn>
          <ci> koffveptp </ci>
          <ci> S131 </ci>
        </apply>
      </math>
    </kineticLaw>
  </reaction>
  <reaction id="R419" reversible="false">
    <listOfReactants>
      <speciesReference species="S132"/>
    </listOfReactants>
    <listOfProducts>
      <speciesReference species="S6"/>
      <speciesReference species="S124"/>
    </listOfProducts>
    <kineticLaw>
      <math xmlns="http://www.w3.org/1998/Math/MathML">
        <apply>
          <times/>
          <ci> koffveptp </ci>
          <ci> S132 </ci>
        </apply>
      </math>
    </kineticLaw>
  </reaction>
  <reaction id="R420" reversible="false">
    <listOfReactants>
      <speciesReference species="S133"/>
    </listOfReactants>
    <listOfProducts>
      <speciesReference species="S6"/>
      <speciesReference species="S125"/>
    </listOfProducts>
    <kineticLaw>
      <math xmlns="http://www.w3.org/1998/Math/MathML">
        <apply>
          <times/>
          <ci> koffveptp </ci>
          <ci> S133 </ci>
        </apply>
      </math>
    </kineticLaw>
  </reaction>
  <reaction id="R421" reversible="false">
    <listOfReactants>
      <speciesReference species="S128"/>
    </listOfReactants>
    <listOfProducts>
      <speciesReference species="S6"/>
      <speciesReference species="S140"/>
    </listOfProducts>
    <kineticLaw>
      <math xmlns="http://www.w3.org/1998/Math/MathML">
```

```
        &lt;apply&gt;
          &lt;times/&gt;
          &lt;cn&gt; 2 &lt;/cn&gt;
          &lt;ci&gt; kactveptp_ang1 &lt;/ci&gt;
          &lt;ci&gt; S128 &lt;/ci&gt;
        &lt;/apply&gt;
      &lt;/math&gt;
    &lt;/kineticLaw&gt;
  &lt;/reaction&gt;
  &lt;reaction id="R422" reversible="false"&gt;
    &lt;listOfReactants&gt;
      &lt;speciesReference species="S129"/&gt;
    &lt;/listOfReactants&gt;
    &lt;listOfProducts&gt;
      &lt;speciesReference species="S6"/&gt;
      &lt;speciesReference species="S141"/&gt;
    &lt;/listOfProducts&gt;
    &lt;kineticLaw&gt;
      &lt;math xmlns="http://www.w3.org/1998/Math/MathML"&gt;
        &lt;apply&gt;
          &lt;times/&gt;
          &lt;cn&gt; 2 &lt;/cn&gt;
          &lt;ci&gt; kactveptp_ang1 &lt;/ci&gt;
          &lt;ci&gt; S129 &lt;/ci&gt;
        &lt;/apply&gt;
      &lt;/math&gt;
    &lt;/kineticLaw&gt;
  &lt;/reaction&gt;
  &lt;reaction id="R423" reversible="false"&gt;
    &lt;listOfReactants&gt;
      &lt;speciesReference species="S132"/&gt;
    &lt;/listOfReactants&gt;
    &lt;listOfProducts&gt;
      &lt;speciesReference species="S6"/&gt;
      &lt;speciesReference species="S144"/&gt;
    &lt;/listOfProducts&gt;
    &lt;kineticLaw&gt;
      &lt;math xmlns="http://www.w3.org/1998/Math/MathML"&gt;
        &lt;apply&gt;
          &lt;times/&gt;
          &lt;ci&gt; kactveptp_ang1 &lt;/ci&gt;
          &lt;ci&gt; S132 &lt;/ci&gt;
        &lt;/apply&gt;
      &lt;/math&gt;
    &lt;/kineticLaw&gt;
  &lt;/reaction&gt;
  &lt;reaction id="R424" reversible="false"&gt;
    &lt;listOfReactants&gt;
      &lt;speciesReference species="S130"/&gt;
    &lt;/listOfReactants&gt;
    &lt;listOfProducts&gt;
      &lt;speciesReference species="S6"/&gt;
      &lt;speciesReference species="S142"/&gt;
    &lt;/listOfProducts&gt;
    &lt;kineticLaw&gt;
      &lt;math xmlns="http://www.w3.org/1998/Math/MathML"&gt;
        &lt;apply&gt;
```

```
          <times/>
          <cn> 2 </cn>
          <ci> kactveptp_ang2 </ci>
          <ci> S130 </ci>
        </apply>
      </math>
    </kineticLaw>
  </reaction>
  <reaction id="R425" reversible="false">
    <listOfReactants>
      <speciesReference species="S131"/>
    </listOfReactants>
    <listOfProducts>
      <speciesReference species="S6"/>
      <speciesReference species="S143"/>
    </listOfProducts>
    <kineticLaw>
      <math xmlns="http://www.w3.org/1998/Math/MathML">
        <apply>
          <times/>
          <cn> 2 </cn>
          <ci> kactveptp_ang2 </ci>
          <ci> S131 </ci>
        </apply>
      </math>
    </kineticLaw>
  </reaction>
  <reaction id="R426" reversible="false">
    <listOfReactants>
      <speciesReference species="S133"/>
    </listOfReactants>
    <listOfProducts>
      <speciesReference species="S6"/>
      <speciesReference species="S145"/>
    </listOfProducts>
    <kineticLaw>
      <math xmlns="http://www.w3.org/1998/Math/MathML">
        <apply>
          <times/>
          <ci> kactveptp_ang2 </ci>
          <ci> S133 </ci>
        </apply>
      </math>
    </kineticLaw>
  </reaction>
  <reaction id="R427" reversible="false">
    <listOfReactants>
      <speciesReference species="S13"/>
      <speciesReference species="S128"/>
    </listOfReactants>
    <listOfProducts>
      <speciesReference species="S73"/>
      <speciesReference species="S128"/>
    </listOfProducts>
    <kineticLaw>
      <math xmlns="http://www.w3.org/1998/Math/MathML">
        <apply>
```

```xml
          &lt;times/&gt;
          &lt;ci&gt; kactPI3KTie2 &lt;/ci&gt;
          &lt;ci&gt; S13 &lt;/ci&gt;
          &lt;ci&gt; S128 &lt;/ci&gt;
        &lt;/apply&gt;
      &lt;/math&gt;
    &lt;/kineticLaw&gt;
  &lt;/reaction&gt;
  &lt;reaction id="R428" reversible="false"&gt;
    &lt;listOfReactants&gt;
      &lt;speciesReference species="S13"/&gt;
      &lt;speciesReference species="S129"/&gt;
    &lt;/listOfReactants&gt;
    &lt;listOfProducts&gt;
      &lt;speciesReference species="S73"/&gt;
      &lt;speciesReference species="S129"/&gt;
    &lt;/listOfProducts&gt;
    &lt;kineticLaw&gt;
      &lt;math xmlns="http://www.w3.org/1998/Math/MathML"&gt;
        &lt;apply&gt;
          &lt;times/&gt;
          &lt;ci&gt; kactPI3KTie2 &lt;/ci&gt;
          &lt;ci&gt; S13 &lt;/ci&gt;
          &lt;ci&gt; S129 &lt;/ci&gt;
        &lt;/apply&gt;
      &lt;/math&gt;
    &lt;/kineticLaw&gt;
  &lt;/reaction&gt;
  &lt;reaction id="R429" reversible="false"&gt;
    &lt;listOfReactants&gt;
      &lt;speciesReference species="S13"/&gt;
      &lt;speciesReference species="S130"/&gt;
    &lt;/listOfReactants&gt;
    &lt;listOfProducts&gt;
      &lt;speciesReference species="S73"/&gt;
      &lt;speciesReference species="S130"/&gt;
    &lt;/listOfProducts&gt;
    &lt;kineticLaw&gt;
      &lt;math xmlns="http://www.w3.org/1998/Math/MathML"&gt;
        &lt;apply&gt;
          &lt;times/&gt;
          &lt;ci&gt; kactPI3KTie2 &lt;/ci&gt;
          &lt;ci&gt; S13 &lt;/ci&gt;
          &lt;ci&gt; S130 &lt;/ci&gt;
        &lt;/apply&gt;
      &lt;/math&gt;
    &lt;/kineticLaw&gt;
  &lt;/reaction&gt;
  &lt;reaction id="R430" reversible="false"&gt;
    &lt;listOfReactants&gt;
      &lt;speciesReference species="S13"/&gt;
      &lt;speciesReference species="S131"/&gt;
    &lt;/listOfReactants&gt;
    &lt;listOfProducts&gt;
      &lt;speciesReference species="S73"/&gt;
      &lt;speciesReference species="S131"/&gt;
    &lt;/listOfProducts&gt;
```

```
    <kineticLaw>
      <math xmlns="http://www.w3.org/1998/Math/MathML">
        <apply>
          <times/>
          <ci> kactPI3KTie2 </ci>
          <ci> S13 </ci>
          <ci> S131 </ci>
        </apply>
      </math>
    </kineticLaw>
  </reaction>
  <reaction id="R431" reversible="false">
    <listOfReactants>
      <speciesReference species="S13"/>
      <speciesReference species="S132"/>
    </listOfReactants>
    <listOfProducts>
      <speciesReference species="S73"/>
      <speciesReference species="S132"/>
    </listOfProducts>
    <kineticLaw>
      <math xmlns="http://www.w3.org/1998/Math/MathML">
        <apply>
          <times/>
          <ci> kactPI3KTie2 </ci>
          <ci> S13 </ci>
          <ci> S132 </ci>
        </apply>
      </math>
    </kineticLaw>
  </reaction>
  <reaction id="R432" reversible="false">
    <listOfReactants>
      <speciesReference species="S13"/>
      <speciesReference species="S133"/>
    </listOfReactants>
    <listOfProducts>
      <speciesReference species="S73"/>
      <speciesReference species="S133"/>
    </listOfProducts>
    <kineticLaw>
      <math xmlns="http://www.w3.org/1998/Math/MathML">
        <apply>
          <times/>
          <ci> kactPI3KTie2 </ci>
          <ci> S13 </ci>
          <ci> S133 </ci>
        </apply>
      </math>
    </kineticLaw>
  </reaction>
  <reaction id="R433" reversible="false">
    <listOfReactants>
      <speciesReference species="S13"/>
      <speciesReference species="S134"/>
    </listOfReactants>
    <listOfProducts>
```

```
    <speciesReference species="S73"/>
    <speciesReference species="S134"/>
  </listOfProducts>
  <kineticLaw>
    <math xmlns="http://www.w3.org/1998/Math/MathML">
      <apply>
        <times/>
        <ci> kactPI3KTie2 </ci>
        <ci> S13 </ci>
        <ci> S134 </ci>
      </apply>
    </math>
  </kineticLaw>
</reaction>
<reaction id="R434" reversible="false">
  <listOfReactants>
    <speciesReference species="S13"/>
    <speciesReference species="S135"/>
  </listOfReactants>
  <listOfProducts>
    <speciesReference species="S73"/>
    <speciesReference species="S135"/>
  </listOfProducts>
  <kineticLaw>
    <math xmlns="http://www.w3.org/1998/Math/MathML">
      <apply>
        <times/>
        <ci> kactPI3KTie2 </ci>
        <ci> S13 </ci>
        <ci> S135 </ci>
      </apply>
    </math>
  </kineticLaw>
</reaction>
<reaction id="R435" reversible="false">
  <listOfReactants>
    <speciesReference species="S13"/>
    <speciesReference species="S136"/>
  </listOfReactants>
  <listOfProducts>
    <speciesReference species="S73"/>
    <speciesReference species="S136"/>
  </listOfProducts>
  <kineticLaw>
    <math xmlns="http://www.w3.org/1998/Math/MathML">
      <apply>
        <times/>
        <ci> kactPI3KTie2 </ci>
        <ci> S13 </ci>
        <ci> S136 </ci>
      </apply>
    </math>
  </kineticLaw>
</reaction>
<reaction id="R436" reversible="false">
  <listOfReactants>
    <speciesReference species="S13"/>
```

```
      <speciesReference species="S137"/>
    </listOfReactants>
    <listOfProducts>
      <speciesReference species="S73"/>
      <speciesReference species="S137"/>
    </listOfProducts>
    <kineticLaw>
      <math xmlns="http://www.w3.org/1998/Math/MathML">
        <apply>
          <times/>
          <ci> kactPI3KTie2 </ci>
          <ci> S13 </ci>
          <ci> S137 </ci>
        </apply>
      </math>
    </kineticLaw>
  </reaction>
  <reaction id="R437" reversible="false">
    <listOfReactants>
      <speciesReference species="S84"/>
      <speciesReference species="S138"/>
    </listOfReactants>
    <listOfProducts>
      <speciesReference species="S127"/>
    </listOfProducts>
    <kineticLaw>
      <math xmlns="http://www.w3.org/1998/Math/MathML">
        <apply>
          <times/>
          <ci> konAKTPIP3 </ci>
          <ci> S84 </ci>
          <ci> S138 </ci>
        </apply>
      </math>
    </kineticLaw>
  </reaction>
  <reaction id="R438" reversible="false">
    <listOfReactants>
      <speciesReference species="S139"/>
    </listOfReactants>
    <listOfProducts>
      <speciesReference species="S84"/>
      <speciesReference species="S146"/>
    </listOfProducts>
    <kineticLaw>
      <math xmlns="http://www.w3.org/1998/Math/MathML">
        <apply>
          <times/>
          <ci> koffAKTPIP3 </ci>
          <ci> S139 </ci>
        </apply>
      </math>
    </kineticLaw>
  </reaction>
  <reaction id="R439" reversible="false">
    <listOfReactants>
      <speciesReference species="S139"/>
```

```
    &lt;/listOfReactants&gt;
    &lt;listOfProducts&gt;
      &lt;speciesReference species="S127"/&gt;
    &lt;/listOfProducts&gt;
    &lt;kineticLaw&gt;
      &lt;math xmlns="http://www.w3.org/1998/Math/MathML"&gt;
        &lt;apply&gt;
          &lt;times/&gt;
          &lt;ci&gt; kpmTORAKT &lt;/ci&gt;
          &lt;ci&gt; S139 &lt;/ci&gt;
        &lt;/apply&gt;
      &lt;/math&gt;
    &lt;/kineticLaw&gt;
  &lt;/reaction&gt;
  &lt;reaction id="R440" reversible="false"&gt;
    &lt;listOfReactants&gt;
      &lt;speciesReference species="S138"/&gt;
    &lt;/listOfReactants&gt;
    &lt;listOfProducts&gt;
      &lt;speciesReference species="S146"/&gt;
    &lt;/listOfProducts&gt;
    &lt;kineticLaw&gt;
      &lt;math xmlns="http://www.w3.org/1998/Math/MathML"&gt;
        &lt;apply&gt;
          &lt;times/&gt;
          &lt;ci&gt; kdp473AKTPPase &lt;/ci&gt;
          &lt;ci&gt; S138 &lt;/ci&gt;
        &lt;/apply&gt;
      &lt;/math&gt;
    &lt;/kineticLaw&gt;
  &lt;/reaction&gt;
  &lt;reaction id="R441" reversible="false"&gt;
    &lt;listOfReactants&gt;
      &lt;speciesReference species="S138"/&gt;
    &lt;/listOfReactants&gt;
    &lt;listOfProducts&gt;
      &lt;speciesReference species="S126"/&gt;
    &lt;/listOfProducts&gt;
    &lt;kineticLaw&gt;
      &lt;math xmlns="http://www.w3.org/1998/Math/MathML"&gt;
        &lt;apply&gt;
          &lt;times/&gt;
          &lt;ci&gt; kdp308AKTPPase &lt;/ci&gt;
          &lt;ci&gt; S138 &lt;/ci&gt;
        &lt;/apply&gt;
      &lt;/math&gt;
    &lt;/kineticLaw&gt;
  &lt;/reaction&gt;
  &lt;reaction id="R442" reversible="false"&gt;
    &lt;listOfReactants&gt;
      &lt;speciesReference species="S139"/&gt;
    &lt;/listOfReactants&gt;
    &lt;listOfProducts&gt;
      &lt;speciesReference species="S97"/&gt;
    &lt;/listOfProducts&gt;
    &lt;kineticLaw&gt;
      &lt;math xmlns="http://www.w3.org/1998/Math/MathML"&gt;
```

```
      <apply>
        <times/>
        <ci> kdp308AKTPPase </ci>
        <ci> S139 </ci>
      </apply>
    </math>
  </kineticLaw>
</reaction>
<reaction id="R443" reversible="false">
  <listOfReactants>
    <speciesReference species="S16"/>
    <speciesReference species="S128"/>
  </listOfReactants>
  <listOfProducts>
    <speciesReference species="S74"/>
    <speciesReference species="S128"/>
  </listOfProducts>
  <kineticLaw>
    <math xmlns="http://www.w3.org/1998/Math/MathML">
      <apply>
        <times/>
        <ci> kprhoa </ci>
        <ci> S16 </ci>
        <ci> S128 </ci>
      </apply>
    </math>
  </kineticLaw>
</reaction>
<reaction id="R444" reversible="false">
  <listOfReactants>
    <speciesReference species="S16"/>
    <speciesReference species="S129"/>
  </listOfReactants>
  <listOfProducts>
    <speciesReference species="S74"/>
    <speciesReference species="S129"/>
  </listOfProducts>
  <kineticLaw>
    <math xmlns="http://www.w3.org/1998/Math/MathML">
      <apply>
        <times/>
        <ci> kprhoa </ci>
        <ci> S16 </ci>
        <ci> S129 </ci>
      </apply>
    </math>
  </kineticLaw>
</reaction>
<reaction id="R445" reversible="false">
  <listOfReactants>
    <speciesReference species="S16"/>
    <speciesReference species="S130"/>
  </listOfReactants>
  <listOfProducts>
    <speciesReference species="S74"/>
    <speciesReference species="S130"/>
  </listOfProducts>
```

```
    &lt;kineticLaw&gt;
      &lt;math xmlns="http://www.w3.org/1998/Math/MathML"&gt;
        &lt;apply&gt;
          &lt;times/&gt;
          &lt;ci&gt; kprhoa &lt;/ci&gt;
          &lt;ci&gt; S16 &lt;/ci&gt;
          &lt;ci&gt; S130 &lt;/ci&gt;
        &lt;/apply&gt;
      &lt;/math&gt;
    &lt;/kineticLaw&gt;
  &lt;/reaction&gt;
  &lt;reaction id="R446" reversible="false"&gt;
    &lt;listOfReactants&gt;
      &lt;speciesReference species="S16"/&gt;
      &lt;speciesReference species="S131"/&gt;
    &lt;/listOfReactants&gt;
    &lt;listOfProducts&gt;
      &lt;speciesReference species="S74"/&gt;
      &lt;speciesReference species="S131"/&gt;
    &lt;/listOfProducts&gt;
    &lt;kineticLaw&gt;
      &lt;math xmlns="http://www.w3.org/1998/Math/MathML"&gt;
        &lt;apply&gt;
          &lt;times/&gt;
          &lt;ci&gt; kprhoa &lt;/ci&gt;
          &lt;ci&gt; S16 &lt;/ci&gt;
          &lt;ci&gt; S131 &lt;/ci&gt;
        &lt;/apply&gt;
      &lt;/math&gt;
    &lt;/kineticLaw&gt;
  &lt;/reaction&gt;
  &lt;reaction id="R447" reversible="false"&gt;
    &lt;listOfReactants&gt;
      &lt;speciesReference species="S16"/&gt;
      &lt;speciesReference species="S132"/&gt;
    &lt;/listOfReactants&gt;
    &lt;listOfProducts&gt;
      &lt;speciesReference species="S74"/&gt;
      &lt;speciesReference species="S132"/&gt;
    &lt;/listOfProducts&gt;
    &lt;kineticLaw&gt;
      &lt;math xmlns="http://www.w3.org/1998/Math/MathML"&gt;
        &lt;apply&gt;
          &lt;times/&gt;
          &lt;ci&gt; kprhoa &lt;/ci&gt;
          &lt;ci&gt; S16 &lt;/ci&gt;
          &lt;ci&gt; S132 &lt;/ci&gt;
        &lt;/apply&gt;
      &lt;/math&gt;
    &lt;/kineticLaw&gt;
  &lt;/reaction&gt;
  &lt;reaction id="R448" reversible="false"&gt;
    &lt;listOfReactants&gt;
      &lt;speciesReference species="S16"/&gt;
      &lt;speciesReference species="S133"/&gt;
    &lt;/listOfReactants&gt;
    &lt;listOfProducts&gt;
```

```
      <speciesReference species="S74"/>
      <speciesReference species="S133"/>
    </listOfProducts>
    <kineticLaw>
      <math xmlns="http://www.w3.org/1998/Math/MathML">
        <apply>
          <times/>
          <ci> kprhoa </ci>
          <ci> S16 </ci>
          <ci> S133 </ci>
        </apply>
      </math>
    </kineticLaw>
  </reaction>
  <reaction id="R449" reversible="false">
    <listOfReactants>
      <speciesReference species="S16"/>
      <speciesReference species="S134"/>
    </listOfReactants>
    <listOfProducts>
      <speciesReference species="S74"/>
      <speciesReference species="S134"/>
    </listOfProducts>
    <kineticLaw>
      <math xmlns="http://www.w3.org/1998/Math/MathML">
        <apply>
          <times/>
          <ci> kprhoa </ci>
          <ci> S16 </ci>
          <ci> S134 </ci>
        </apply>
      </math>
    </kineticLaw>
  </reaction>
  <reaction id="R450" reversible="false">
    <listOfReactants>
      <speciesReference species="S16"/>
      <speciesReference species="S135"/>
    </listOfReactants>
    <listOfProducts>
      <speciesReference species="S74"/>
      <speciesReference species="S135"/>
    </listOfProducts>
    <kineticLaw>
      <math xmlns="http://www.w3.org/1998/Math/MathML">
        <apply>
          <times/>
          <ci> kprhoa </ci>
          <ci> S16 </ci>
          <ci> S135 </ci>
        </apply>
      </math>
    </kineticLaw>
  </reaction>
  <reaction id="R451" reversible="false">
    <listOfReactants>
      <speciesReference species="S16"/>
```

```
    <speciesReference species="S136"/>
  </listOfReactants>
  <listOfProducts>
    <speciesReference species="S74"/>
    <speciesReference species="S136"/>
  </listOfProducts>
  <kineticLaw>
    <math xmlns="http://www.w3.org/1998/Math/MathML">
      <apply>
        <times/>
        <ci> kprhoa </ci>
        <ci> S16 </ci>
        <ci> S136 </ci>
      </apply>
    </math>
  </kineticLaw>
</reaction>
<reaction id="R452" reversible="false">
  <listOfReactants>
    <speciesReference species="S16"/>
    <speciesReference species="S137"/>
  </listOfReactants>
  <listOfProducts>
    <speciesReference species="S74"/>
    <speciesReference species="S137"/>
  </listOfProducts>
  <kineticLaw>
    <math xmlns="http://www.w3.org/1998/Math/MathML">
      <apply>
        <times/>
        <ci> kprhoa </ci>
        <ci> S16 </ci>
        <ci> S137 </ci>
      </apply>
    </math>
  </kineticLaw>
</reaction>
<reaction id="R453" reversible="false">
  <listOfReactants>
    <speciesReference species="S20"/>
    <speciesReference species="S128"/>
  </listOfReactants>
  <listOfProducts>
    <speciesReference species="S75"/>
    <speciesReference species="S128"/>
  </listOfProducts>
  <kineticLaw>
    <math xmlns="http://www.w3.org/1998/Math/MathML">
      <apply>
        <times/>
        <ci> kactabin2 </ci>
        <ci> S20 </ci>
        <ci> S128 </ci>
      </apply>
    </math>
  </kineticLaw>
</reaction>
```

```xml
<reaction id="R454" reversible="false">
  <listOfReactants>
    <speciesReference species="S20"/>
    <speciesReference species="S129"/>
  </listOfReactants>
  <listOfProducts>
    <speciesReference species="S75"/>
    <speciesReference species="S129"/>
  </listOfProducts>
  <kineticLaw>
    <math xmlns="http://www.w3.org/1998/Math/MathML">
      <apply>
        <times/>
        <ci> kactabin2 </ci>
        <ci> S20 </ci>
        <ci> S129 </ci>
      </apply>
    </math>
  </kineticLaw>
</reaction>
<reaction id="R455" reversible="false">
  <listOfReactants>
    <speciesReference species="S20"/>
    <speciesReference species="S130"/>
  </listOfReactants>
  <listOfProducts>
    <speciesReference species="S75"/>
    <speciesReference species="S130"/>
  </listOfProducts>
  <kineticLaw>
    <math xmlns="http://www.w3.org/1998/Math/MathML">
      <apply>
        <times/>
        <ci> kactabin2 </ci>
        <ci> S20 </ci>
        <ci> S130 </ci>
      </apply>
    </math>
  </kineticLaw>
</reaction>
<reaction id="R456" reversible="false">
  <listOfReactants>
    <speciesReference species="S20"/>
    <speciesReference species="S131"/>
  </listOfReactants>
  <listOfProducts>
    <speciesReference species="S75"/>
    <speciesReference species="S131"/>
  </listOfProducts>
  <kineticLaw>
    <math xmlns="http://www.w3.org/1998/Math/MathML">
      <apply>
        <times/>
        <ci> kactabin2 </ci>
        <ci> S20 </ci>
        <ci> S131 </ci>
      </apply>
```

```
    </math>
  </kineticLaw>
</reaction>
<reaction id="R457" reversible="false">
  <listOfReactants>
    <speciesReference species="S20"/>
    <speciesReference species="S132"/>
  </listOfReactants>
  <listOfProducts>
    <speciesReference species="S75"/>
    <speciesReference species="S132"/>
  </listOfProducts>
  <kineticLaw>
    <math xmlns="http://www.w3.org/1998/Math/MathML">
      <apply>
        <times/>
        <ci> kactabin2 </ci>
        <ci> S20 </ci>
        <ci> S132 </ci>
      </apply>
    </math>
  </kineticLaw>
</reaction>
<reaction id="R458" reversible="false">
  <listOfReactants>
    <speciesReference species="S20"/>
    <speciesReference species="S133"/>
  </listOfReactants>
  <listOfProducts>
    <speciesReference species="S75"/>
    <speciesReference species="S133"/>
  </listOfProducts>
  <kineticLaw>
    <math xmlns="http://www.w3.org/1998/Math/MathML">
      <apply>
        <times/>
        <ci> kactabin2 </ci>
        <ci> S20 </ci>
        <ci> S133 </ci>
      </apply>
    </math>
  </kineticLaw>
</reaction>
<reaction id="R459" reversible="false">
  <listOfReactants>
    <speciesReference species="S20"/>
    <speciesReference species="S134"/>
  </listOfReactants>
  <listOfProducts>
    <speciesReference species="S75"/>
    <speciesReference species="S134"/>
  </listOfProducts>
  <kineticLaw>
    <math xmlns="http://www.w3.org/1998/Math/MathML">
      <apply>
        <times/>
        <ci> kactabin2 </ci>
```

```
        <ci> S20 </ci>
        <ci> S134 </ci>
      </apply>
    </math>
  </kineticLaw>
</reaction>
<reaction id="R460" reversible="false">
  <listOfReactants>
    <speciesReference species="S20"/>
    <speciesReference species="S135"/>
  </listOfReactants>
  <listOfProducts>
    <speciesReference species="S75"/>
    <speciesReference species="S135"/>
  </listOfProducts>
  <kineticLaw>
    <math xmlns="http://www.w3.org/1998/Math/MathML">
      <apply>
        <times/>
        <ci> kactabin2 </ci>
        <ci> S20 </ci>
        <ci> S135 </ci>
      </apply>
    </math>
  </kineticLaw>
</reaction>
<reaction id="R461" reversible="false">
  <listOfReactants>
    <speciesReference species="S20"/>
    <speciesReference species="S136"/>
  </listOfReactants>
  <listOfProducts>
    <speciesReference species="S75"/>
    <speciesReference species="S136"/>
  </listOfProducts>
  <kineticLaw>
    <math xmlns="http://www.w3.org/1998/Math/MathML">
      <apply>
        <times/>
        <ci> kactabin2 </ci>
        <ci> S20 </ci>
        <ci> S136 </ci>
      </apply>
    </math>
  </kineticLaw>
</reaction>
<reaction id="R462" reversible="false">
  <listOfReactants>
    <speciesReference species="S20"/>
    <speciesReference species="S137"/>
  </listOfReactants>
  <listOfProducts>
    <speciesReference species="S75"/>
    <speciesReference species="S137"/>
  </listOfProducts>
  <kineticLaw>
    <math xmlns="http://www.w3.org/1998/Math/MathML">
```

```
      <apply>
        <times/>
        <ci> kactabin2 </ci>
        <ci> S20 </ci>
        <ci> S137 </ci>
      </apply>
    </math>
  </kineticLaw>
</reaction>
<reaction id="R463" reversible="false">
  <listOfReactants>
    <speciesReference species="S140"/>
  </listOfReactants>
  <listOfProducts>
    <speciesReference species="S6"/>
    <speciesReference species="S134"/>
  </listOfProducts>
  <kineticLaw>
    <math xmlns="http://www.w3.org/1998/Math/MathML">
      <apply>
        <times/>
        <ci> koffveptp </ci>
        <ci> S140 </ci>
      </apply>
    </math>
  </kineticLaw>
</reaction>
<reaction id="R464" reversible="false">
  <listOfReactants>
    <speciesReference species="S141"/>
  </listOfReactants>
  <listOfProducts>
    <speciesReference species="S6"/>
    <speciesReference species="S135"/>
  </listOfProducts>
  <kineticLaw>
    <math xmlns="http://www.w3.org/1998/Math/MathML">
      <apply>
        <times/>
        <ci> koffveptp </ci>
        <ci> S141 </ci>
      </apply>
    </math>
  </kineticLaw>
</reaction>
<reaction id="R465" reversible="false">
  <listOfReactants>
    <speciesReference species="S142"/>
  </listOfReactants>
  <listOfProducts>
    <speciesReference species="S6"/>
    <speciesReference species="S136"/>
  </listOfProducts>
  <kineticLaw>
    <math xmlns="http://www.w3.org/1998/Math/MathML">
      <apply>
        <times/>
```

```
        &lt;ci&gt; koffveptp &lt;/ci&gt;
        &lt;ci&gt; S142 &lt;/ci&gt;
      &lt;/apply&gt;
    &lt;/math&gt;
  &lt;/kineticLaw&gt;
&lt;/reaction&gt;
&lt;reaction id="R466" reversible="false"&gt;
  &lt;listOfReactants&gt;
    &lt;speciesReference species="S143"/&gt;
  &lt;/listOfReactants&gt;
  &lt;listOfProducts&gt;
    &lt;speciesReference species="S6"/&gt;
    &lt;speciesReference species="S137"/&gt;
  &lt;/listOfProducts&gt;
  &lt;kineticLaw&gt;
    &lt;math xmlns="http://www.w3.org/1998/Math/MathML"&gt;
      &lt;apply&gt;
        &lt;times/&gt;
        &lt;ci&gt; koffveptp &lt;/ci&gt;
        &lt;ci&gt; S143 &lt;/ci&gt;
      &lt;/apply&gt;
    &lt;/math&gt;
  &lt;/kineticLaw&gt;
&lt;/reaction&gt;
&lt;reaction id="R467" reversible="false"&gt;
  &lt;listOfReactants&gt;
    &lt;speciesReference species="S140"/&gt;
  &lt;/listOfReactants&gt;
  &lt;listOfProducts&gt;
    &lt;speciesReference species="S6"/&gt;
    &lt;speciesReference species="S147"/&gt;
  &lt;/listOfProducts&gt;
  &lt;kineticLaw&gt;
    &lt;math xmlns="http://www.w3.org/1998/Math/MathML"&gt;
      &lt;apply&gt;
        &lt;times/&gt;
        &lt;ci&gt; kactveptp_ang1 &lt;/ci&gt;
        &lt;ci&gt; S140 &lt;/ci&gt;
      &lt;/apply&gt;
    &lt;/math&gt;
  &lt;/kineticLaw&gt;
&lt;/reaction&gt;
&lt;reaction id="R468" reversible="false"&gt;
  &lt;listOfReactants&gt;
    &lt;speciesReference species="S141"/&gt;
  &lt;/listOfReactants&gt;
  &lt;listOfProducts&gt;
    &lt;speciesReference species="S6"/&gt;
    &lt;speciesReference species="S148"/&gt;
  &lt;/listOfProducts&gt;
  &lt;kineticLaw&gt;
    &lt;math xmlns="http://www.w3.org/1998/Math/MathML"&gt;
      &lt;apply&gt;
        &lt;times/&gt;
        &lt;ci&gt; kactveptp_ang1 &lt;/ci&gt;
        &lt;ci&gt; S141 &lt;/ci&gt;
      &lt;/apply&gt;
```

```
      </math>
    </kineticLaw>
  </reaction>
  <reaction id="R469" reversible="false">
    <listOfReactants>
      <speciesReference species="S142"/>
    </listOfReactants>
    <listOfProducts>
      <speciesReference species="S6"/>
      <speciesReference species="S149"/>
    </listOfProducts>
    <kineticLaw>
      <math xmlns="http://www.w3.org/1998/Math/MathML">
        <apply>
          <times/>
          <ci> kactveptp_ang2 </ci>
          <ci> S142 </ci>
        </apply>
      </math>
    </kineticLaw>
  </reaction>
  <reaction id="R470" reversible="false">
    <listOfReactants>
      <speciesReference species="S143"/>
    </listOfReactants>
    <listOfProducts>
      <speciesReference species="S6"/>
      <speciesReference species="S150"/>
    </listOfProducts>
    <kineticLaw>
      <math xmlns="http://www.w3.org/1998/Math/MathML">
        <apply>
          <times/>
          <ci> kactveptp_ang2 </ci>
          <ci> S143 </ci>
        </apply>
      </math>
    </kineticLaw>
  </reaction>
  <reaction id="R471" reversible="false">
    <listOfReactants>
      <speciesReference species="S13"/>
      <speciesReference species="S140"/>
    </listOfReactants>
    <listOfProducts>
      <speciesReference species="S73"/>
      <speciesReference species="S140"/>
    </listOfProducts>
    <kineticLaw>
      <math xmlns="http://www.w3.org/1998/Math/MathML">
        <apply>
          <times/>
          <ci> kactPI3KTie2 </ci>
          <ci> S13 </ci>
          <ci> S140 </ci>
        </apply>
      </math>
```

```
    &lt;/kineticLaw&gt;
  &lt;/reaction&gt;
  &lt;reaction id="R472" reversible="false"&gt;
    &lt;listOfReactants&gt;
      &lt;speciesReference species="S13"/&gt;
      &lt;speciesReference species="S141"/&gt;
    &lt;/listOfReactants&gt;
    &lt;listOfProducts&gt;
      &lt;speciesReference species="S73"/&gt;
      &lt;speciesReference species="S141"/&gt;
    &lt;/listOfProducts&gt;
    &lt;kineticLaw&gt;
      &lt;math xmlns="http://www.w3.org/1998/Math/MathML"&gt;
        &lt;apply&gt;
          &lt;times/&gt;
          &lt;ci&gt; kactPI3KTie2 &lt;/ci&gt;
          &lt;ci&gt; S13 &lt;/ci&gt;
          &lt;ci&gt; S141 &lt;/ci&gt;
        &lt;/apply&gt;
      &lt;/math&gt;
    &lt;/kineticLaw&gt;
  &lt;/reaction&gt;
  &lt;reaction id="R473" reversible="false"&gt;
    &lt;listOfReactants&gt;
      &lt;speciesReference species="S13"/&gt;
      &lt;speciesReference species="S142"/&gt;
    &lt;/listOfReactants&gt;
    &lt;listOfProducts&gt;
      &lt;speciesReference species="S73"/&gt;
      &lt;speciesReference species="S142"/&gt;
    &lt;/listOfProducts&gt;
    &lt;kineticLaw&gt;
      &lt;math xmlns="http://www.w3.org/1998/Math/MathML"&gt;
        &lt;apply&gt;
          &lt;times/&gt;
          &lt;ci&gt; kactPI3KTie2 &lt;/ci&gt;
          &lt;ci&gt; S13 &lt;/ci&gt;
          &lt;ci&gt; S142 &lt;/ci&gt;
        &lt;/apply&gt;
      &lt;/math&gt;
    &lt;/kineticLaw&gt;
  &lt;/reaction&gt;
  &lt;reaction id="R474" reversible="false"&gt;
    &lt;listOfReactants&gt;
      &lt;speciesReference species="S13"/&gt;
      &lt;speciesReference species="S143"/&gt;
    &lt;/listOfReactants&gt;
    &lt;listOfProducts&gt;
      &lt;speciesReference species="S73"/&gt;
      &lt;speciesReference species="S143"/&gt;
    &lt;/listOfProducts&gt;
    &lt;kineticLaw&gt;
      &lt;math xmlns="http://www.w3.org/1998/Math/MathML"&gt;
        &lt;apply&gt;
          &lt;times/&gt;
          &lt;ci&gt; kactPI3KTie2 &lt;/ci&gt;
          &lt;ci&gt; S13 &lt;/ci&gt;
```

```
        <ci> S143 </ci>
      </apply>
    </math>
  </kineticLaw>
</reaction>
<reaction id="R475" reversible="false">
  <listOfReactants>
    <speciesReference species="S13"/>
    <speciesReference species="S144"/>
  </listOfReactants>
  <listOfProducts>
    <speciesReference species="S73"/>
    <speciesReference species="S144"/>
  </listOfProducts>
  <kineticLaw>
    <math xmlns="http://www.w3.org/1998/Math/MathML">
      <apply>
        <times/>
        <ci> kactPI3KTie2 </ci>
        <ci> S13 </ci>
        <ci> S144 </ci>
      </apply>
    </math>
  </kineticLaw>
</reaction>
<reaction id="R476" reversible="false">
  <listOfReactants>
    <speciesReference species="S13"/>
    <speciesReference species="S145"/>
  </listOfReactants>
  <listOfProducts>
    <speciesReference species="S73"/>
    <speciesReference species="S145"/>
  </listOfProducts>
  <kineticLaw>
    <math xmlns="http://www.w3.org/1998/Math/MathML">
      <apply>
        <times/>
        <ci> kactPI3KTie2 </ci>
        <ci> S13 </ci>
        <ci> S145 </ci>
      </apply>
    </math>
  </kineticLaw>
</reaction>
<reaction id="R477" reversible="false">
  <listOfReactants>
    <speciesReference species="S84"/>
    <speciesReference species="S146"/>
  </listOfReactants>
  <listOfProducts>
    <speciesReference species="S139"/>
  </listOfProducts>
  <kineticLaw>
    <math xmlns="http://www.w3.org/1998/Math/MathML">
      <apply>
        <times/>
```

```
        <ci> konAKTPIP3 </ci>
        <ci> S84 </ci>
        <ci> S146 </ci>
      </apply>
    </math>
  </kineticLaw>
</reaction>
<reaction id="R478" reversible="false">
  <listOfReactants>
    <speciesReference species="S146"/>
  </listOfReactants>
  <listOfProducts>
    <speciesReference species="S14"/>
  </listOfProducts>
  <kineticLaw>
    <math xmlns="http://www.w3.org/1998/Math/MathML">
      <apply>
        <times/>
        <ci> kdp308AKTPPase </ci>
        <ci> S146 </ci>
      </apply>
    </math>
  </kineticLaw>
</reaction>
<reaction id="R479" reversible="false">
  <listOfReactants>
    <speciesReference species="S16"/>
    <speciesReference species="S140"/>
  </listOfReactants>
  <listOfProducts>
    <speciesReference species="S74"/>
    <speciesReference species="S140"/>
  </listOfProducts>
  <kineticLaw>
    <math xmlns="http://www.w3.org/1998/Math/MathML">
      <apply>
        <times/>
        <ci> kprhoa </ci>
        <ci> S16 </ci>
        <ci> S140 </ci>
      </apply>
    </math>
  </kineticLaw>
</reaction>
<reaction id="R480" reversible="false">
  <listOfReactants>
    <speciesReference species="S16"/>
    <speciesReference species="S141"/>
  </listOfReactants>
  <listOfProducts>
    <speciesReference species="S74"/>
    <speciesReference species="S141"/>
  </listOfProducts>
  <kineticLaw>
    <math xmlns="http://www.w3.org/1998/Math/MathML">
      <apply>
        <times/>
```

```
          <ci> kprhoa </ci>
          <ci> S16 </ci>
          <ci> S141 </ci>
        </apply>
      </math>
    </kineticLaw>
  </reaction>
  <reaction id="R481" reversible="false">
    <listOfReactants>
      <speciesReference species="S16"/>
      <speciesReference species="S142"/>
    </listOfReactants>
    <listOfProducts>
      <speciesReference species="S74"/>
      <speciesReference species="S142"/>
    </listOfProducts>
    <kineticLaw>
      <math xmlns="http://www.w3.org/1998/Math/MathML">
        <apply>
          <times/>
          <ci> kprhoa </ci>
          <ci> S16 </ci>
          <ci> S142 </ci>
        </apply>
      </math>
    </kineticLaw>
  </reaction>
  <reaction id="R482" reversible="false">
    <listOfReactants>
      <speciesReference species="S16"/>
      <speciesReference species="S143"/>
    </listOfReactants>
    <listOfProducts>
      <speciesReference species="S74"/>
      <speciesReference species="S143"/>
    </listOfProducts>
    <kineticLaw>
      <math xmlns="http://www.w3.org/1998/Math/MathML">
        <apply>
          <times/>
          <ci> kprhoa </ci>
          <ci> S16 </ci>
          <ci> S143 </ci>
        </apply>
      </math>
    </kineticLaw>
  </reaction>
  <reaction id="R483" reversible="false">
    <listOfReactants>
      <speciesReference species="S16"/>
      <speciesReference species="S144"/>
    </listOfReactants>
    <listOfProducts>
      <speciesReference species="S74"/>
      <speciesReference species="S144"/>
    </listOfProducts>
    <kineticLaw>
```

```
        <math xmlns="http://www.w3.org/1998/Math/MathML">
          <apply>
            <times/>
            <ci> kprhoa </ci>
            <ci> S16 </ci>
            <ci> S144 </ci>
          </apply>
        </math>
      </kineticLaw>
    </reaction>
    <reaction id="R484" reversible="false">
      <listOfReactants>
        <speciesReference species="S16"/>
        <speciesReference species="S145"/>
      </listOfReactants>
      <listOfProducts>
        <speciesReference species="S74"/>
        <speciesReference species="S145"/>
      </listOfProducts>
      <kineticLaw>
        <math xmlns="http://www.w3.org/1998/Math/MathML">
          <apply>
            <times/>
            <ci> kprhoa </ci>
            <ci> S16 </ci>
            <ci> S145 </ci>
          </apply>
        </math>
      </kineticLaw>
    </reaction>
    <reaction id="R485" reversible="false">
      <listOfReactants>
        <speciesReference species="S20"/>
        <speciesReference species="S140"/>
      </listOfReactants>
      <listOfProducts>
        <speciesReference species="S75"/>
        <speciesReference species="S140"/>
      </listOfProducts>
      <kineticLaw>
        <math xmlns="http://www.w3.org/1998/Math/MathML">
          <apply>
            <times/>
            <ci> kactabin2 </ci>
            <ci> S20 </ci>
            <ci> S140 </ci>
          </apply>
        </math>
      </kineticLaw>
    </reaction>
    <reaction id="R486" reversible="false">
      <listOfReactants>
        <speciesReference species="S20"/>
        <speciesReference species="S141"/>
      </listOfReactants>
      <listOfProducts>
        <speciesReference species="S75"/>
```

```
      <speciesReference species="S141"/>
    </listOfProducts>
    <kineticLaw>
      <math xmlns="http://www.w3.org/1998/Math/MathML">
        <apply>
          <times/>
          <ci> kactabin2 </ci>
          <ci> S20 </ci>
          <ci> S141 </ci>
        </apply>
      </math>
    </kineticLaw>
  </reaction>
  <reaction id="R487" reversible="false">
    <listOfReactants>
      <speciesReference species="S20"/>
      <speciesReference species="S142"/>
    </listOfReactants>
    <listOfProducts>
      <speciesReference species="S75"/>
      <speciesReference species="S142"/>
    </listOfProducts>
    <kineticLaw>
      <math xmlns="http://www.w3.org/1998/Math/MathML">
        <apply>
          <times/>
          <ci> kactabin2 </ci>
          <ci> S20 </ci>
          <ci> S142 </ci>
        </apply>
      </math>
    </kineticLaw>
  </reaction>
  <reaction id="R488" reversible="false">
    <listOfReactants>
      <speciesReference species="S20"/>
      <speciesReference species="S143"/>
    </listOfReactants>
    <listOfProducts>
      <speciesReference species="S75"/>
      <speciesReference species="S143"/>
    </listOfProducts>
    <kineticLaw>
      <math xmlns="http://www.w3.org/1998/Math/MathML">
        <apply>
          <times/>
          <ci> kactabin2 </ci>
          <ci> S20 </ci>
          <ci> S143 </ci>
        </apply>
      </math>
    </kineticLaw>
  </reaction>
  <reaction id="R489" reversible="false">
    <listOfReactants>
      <speciesReference species="S20"/>
      <speciesReference species="S144"/>
```

```
    &lt;/listOfReactants&gt;
    &lt;listOfProducts&gt;
      &lt;speciesReference species="S75"/&gt;
      &lt;speciesReference species="S144"/&gt;
    &lt;/listOfProducts&gt;
    &lt;kineticLaw&gt;
      &lt;math xmlns="http://www.w3.org/1998/Math/MathML"&gt;
        &lt;apply&gt;
          &lt;times/&gt;
          &lt;ci&gt; kactabin2 &lt;/ci&gt;
          &lt;ci&gt; S20 &lt;/ci&gt;
          &lt;ci&gt; S144 &lt;/ci&gt;
        &lt;/apply&gt;
      &lt;/math&gt;
    &lt;/kineticLaw&gt;
  &lt;/reaction&gt;
  &lt;reaction id="R490" reversible="false"&gt;
    &lt;listOfReactants&gt;
      &lt;speciesReference species="S20"/&gt;
      &lt;speciesReference species="S145"/&gt;
    &lt;/listOfReactants&gt;
    &lt;listOfProducts&gt;
      &lt;speciesReference species="S75"/&gt;
      &lt;speciesReference species="S145"/&gt;
    &lt;/listOfProducts&gt;
    &lt;kineticLaw&gt;
      &lt;math xmlns="http://www.w3.org/1998/Math/MathML"&gt;
        &lt;apply&gt;
          &lt;times/&gt;
          &lt;ci&gt; kactabin2 &lt;/ci&gt;
          &lt;ci&gt; S20 &lt;/ci&gt;
          &lt;ci&gt; S145 &lt;/ci&gt;
        &lt;/apply&gt;
      &lt;/math&gt;
    &lt;/kineticLaw&gt;
  &lt;/reaction&gt;
  &lt;reaction id="R491" reversible="false"&gt;
    &lt;listOfReactants&gt;
      &lt;speciesReference species="S13"/&gt;
      &lt;speciesReference species="S147"/&gt;
    &lt;/listOfReactants&gt;
    &lt;listOfProducts&gt;
      &lt;speciesReference species="S73"/&gt;
      &lt;speciesReference species="S147"/&gt;
    &lt;/listOfProducts&gt;
    &lt;kineticLaw&gt;
      &lt;math xmlns="http://www.w3.org/1998/Math/MathML"&gt;
        &lt;apply&gt;
          &lt;times/&gt;
          &lt;ci&gt; kactPI3KTie2 &lt;/ci&gt;
          &lt;ci&gt; S13 &lt;/ci&gt;
          &lt;ci&gt; S147 &lt;/ci&gt;
        &lt;/apply&gt;
      &lt;/math&gt;
    &lt;/kineticLaw&gt;
  &lt;/reaction&gt;
  &lt;reaction id="R492" reversible="false"&gt;
```

```
    &lt;listOfReactants&gt;
      &lt;speciesReference species="S13"/&gt;
      &lt;speciesReference species="S149"/&gt;
    &lt;/listOfReactants&gt;
    &lt;listOfProducts&gt;
      &lt;speciesReference species="S73"/&gt;
      &lt;speciesReference species="S149"/&gt;
    &lt;/listOfProducts&gt;
    &lt;kineticLaw&gt;
      &lt;math xmlns="http://www.w3.org/1998/Math/MathML"&gt;
        &lt;apply&gt;
          &lt;times/&gt;
          &lt;ci&gt; kactPI3KTie2 &lt;/ci&gt;
          &lt;ci&gt; S13 &lt;/ci&gt;
          &lt;ci&gt; S149 &lt;/ci&gt;
        &lt;/apply&gt;
      &lt;/math&gt;
    &lt;/kineticLaw&gt;
  &lt;/reaction&gt;
  &lt;reaction id="R493" reversible="false"&gt;
    &lt;listOfReactants&gt;
      &lt;speciesReference species="S16"/&gt;
      &lt;speciesReference species="S147"/&gt;
    &lt;/listOfReactants&gt;
    &lt;listOfProducts&gt;
      &lt;speciesReference species="S74"/&gt;
      &lt;speciesReference species="S147"/&gt;
    &lt;/listOfProducts&gt;
    &lt;kineticLaw&gt;
      &lt;math xmlns="http://www.w3.org/1998/Math/MathML"&gt;
        &lt;apply&gt;
          &lt;times/&gt;
          &lt;ci&gt; kprhoa &lt;/ci&gt;
          &lt;ci&gt; S16 &lt;/ci&gt;
          &lt;ci&gt; S147 &lt;/ci&gt;
        &lt;/apply&gt;
      &lt;/math&gt;
    &lt;/kineticLaw&gt;
  &lt;/reaction&gt;
  &lt;reaction id="R494" reversible="false"&gt;
    &lt;listOfReactants&gt;
      &lt;speciesReference species="S16"/&gt;
      &lt;speciesReference species="S149"/&gt;
    &lt;/listOfReactants&gt;
    &lt;listOfProducts&gt;
      &lt;speciesReference species="S74"/&gt;
      &lt;speciesReference species="S149"/&gt;
    &lt;/listOfProducts&gt;
    &lt;kineticLaw&gt;
      &lt;math xmlns="http://www.w3.org/1998/Math/MathML"&gt;
        &lt;apply&gt;
          &lt;times/&gt;
          &lt;ci&gt; kprhoa &lt;/ci&gt;
          &lt;ci&gt; S16 &lt;/ci&gt;
          &lt;ci&gt; S149 &lt;/ci&gt;
        &lt;/apply&gt;
      &lt;/math&gt;
```

```xml
        &lt;/kineticLaw&gt;
      &lt;/reaction&gt;
      &lt;reaction id="R495" reversible="false"&gt;
        &lt;listOfReactants&gt;
          &lt;speciesReference species="S20"/&gt;
          &lt;speciesReference species="S147"/&gt;
        &lt;/listOfReactants&gt;
        &lt;listOfProducts&gt;
          &lt;speciesReference species="S75"/&gt;
          &lt;speciesReference species="S147"/&gt;
        &lt;/listOfProducts&gt;
        &lt;kineticLaw&gt;
          &lt;math xmlns="http://www.w3.org/1998/Math/MathML"&gt;
            &lt;apply&gt;
              &lt;times/&gt;
              &lt;ci&gt; kactabin2 &lt;/ci&gt;
              &lt;ci&gt; S20 &lt;/ci&gt;
              &lt;ci&gt; S147 &lt;/ci&gt;
            &lt;/apply&gt;
          &lt;/math&gt;
        &lt;/kineticLaw&gt;
      &lt;/reaction&gt;
      &lt;reaction id="R496" reversible="false"&gt;
        &lt;listOfReactants&gt;
          &lt;speciesReference species="S20"/&gt;
          &lt;speciesReference species="S149"/&gt;
        &lt;/listOfReactants&gt;
        &lt;listOfProducts&gt;
          &lt;speciesReference species="S75"/&gt;
          &lt;speciesReference species="S149"/&gt;
        &lt;/listOfProducts&gt;
        &lt;kineticLaw&gt;
          &lt;math xmlns="http://www.w3.org/1998/Math/MathML"&gt;
            &lt;apply&gt;
              &lt;times/&gt;
              &lt;ci&gt; kactabin2 &lt;/ci&gt;
              &lt;ci&gt; S20 &lt;/ci&gt;
              &lt;ci&gt; S149 &lt;/ci&gt;
            &lt;/apply&gt;
          &lt;/math&gt;
        &lt;/kineticLaw&gt;
      &lt;/reaction&gt;
    &lt;/listOfReactions&gt;
  &lt;/model&gt;
&lt;/sbml&gt;
```
